# Singlet Fission in Push–Pull *Para*‐Azaquinodimethane Films under the Gaze of Time Resolved Optical and Magnetic Spectroscopy

**DOI:** 10.1002/anie.202520838

**Published:** 2025-12-26

**Authors:** Martina Alebardi, Angelo Carella, Alessandro Grasso, Enrico Sorbelli, Francesco Lazzarin, Cristina Munzone, Cosimo Gianluca Fortuna, Fausto Elisei, Anna Spalletti, Carmela Bonaccorso, Marilena Di Valentin, Benedetta Carlotti

**Affiliations:** ^1^ Department of Chemistry, Biology and Biotechnology University of Perugia via elce di sotto n.8 Perugia 06123 Italy; ^2^ Department of Chemical Sciences University of Padova via Francesco Marzolo n.1 Padova 35131 Italy; ^3^ Department of Chemical Sciences University of Catania V.le A. Doria n.6 Catania 95125 Italy

**Keywords:** Femtosecond and nanosecond transient absorption, Push–pull quinoidal derivatives, Singlet fission, TD‐DFT calculations, Time resolved electron paramagnetic resonance

## Abstract

In this study, a totally green synthetic protocol was employed to obtain four new donor–acceptor–donor *para*‐azaquinodimethane (pAQM) derivatives to uncover the effect of tuning the push–pull character on their photobehaviour in solution as well as in thin film and in particular on their capability of undergoing singlet fission (SF). The bisthiophene and substituted benzene were used as electron‐donor groups at opposite sides of the pAQM electron‐acceptor core in asymmetric structures (**AsOMe** and **AsNMe_2_**), whereas different 5‐phenyl thiophene moieties were symmetrically linked to the pAQM core in **TPh** and **TPhOMe**. The photoinduced excited state dynamics was investigated in a synergic effort by employing both time resolved optical and electron paramagnetic resonance (EPR) spectroscopies, with the aid of TD‐DFT calculations. Our femtosecond transient absorption results showed that intermolecular SF is enabled in the solid state aggregates of these compounds: the fastest and most efficient SF was revealed for the structure with the strongest push–pull degree (**AsNMe_2_**). The other asymmetric molecule (**AsOMe**) interestingly exhibited higher energetic SF‐generated triplet excitons (1.3 eV) than literature SF‐materials, as demonstrated by the nanosecond transient absorption sensitization experiments, with possible intriguing implications toward silicon matched SF photovoltaics. Time resolved EPR measurements unveiled the spectral signature of the quintet multiexciton intermediate ^5^(TT) in the pAQM thin films for the first time, to the best of our knowledge, for such unconventional SF materials. The high‐spin photoinduced ^5^(TT) appears to be a promising candidate for Quantum Information Science and technologies as a viable molecular spin qudit.

## Graphical abstract

New push–pull para‐azaquinodimethane derivatives were synthetized through a sustainable protocol. Transient absorption results showed ultrafast intermolecular singlet fission (SF) in film and demonstrated for **AsOMe** higher triplet energy than for literature SF materials, with intriguing perspectives for photovoltaics toward silicon matched SF. The high‐spin quintet observed via time resolved Electron Paramagnetic Resonance appears to be a viable molecular spin qudit for quantum information science applications.

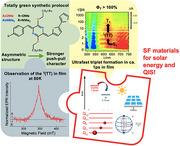

## Introduction

Singlet Fission (SF) is a peculiar photophysical process through which a high energy singlet excited state down converts to give two triplets of roughly half the energy.^[^ ^1^, ^2^ ^]^ For SF to take place, the energy of the S_1_ state should be at least equal to twice the energy of the T_1_ state: E(S_1_) ≥ 2E(T_1_). Through SF, by absorption of a single photon two triplet excitons are produced. As a result, in SF materials the triplet quantum yield may exceed 100% and approach 200%. The intermediate species between the S_1_ and T_1_ excited states is the correlated triplet pair or double triplet ^1^(TT), where the two triplets are coupled together into a biexciton characterized by an overall singlet spin multiplicity.^[^ ^3^, ^4^ ^]^ Such biexcitonic state generally exhibits a double nature: the lifetime of a singlet and the absorption spectrum of a triplet excited state.^[^ ^5^, ^6^, ^7^ ^]^ Passing through the ^1^(TT) intermediate, SF is a spin allowed production of the triplet and it is thus generally ultrafast. If the ^1^(TT) lifetime is long enough, spin evolution may occur to produce the quintet double triplet state ^5^(TT) before the spins decorrelate to give the two independent triplets.^[^ ^8^, ^9^ ^]^

SF is of utmost interest in many different fields of applications, first of all in photovoltaics.^[^ ^10^, ^11^, ^12^ ^]^ SF chromophores may be employed in all‐organic solar cells, as their multiple triplet exciton generation ability allows multiple charge extraction to electron acceptors.^[^ ^13^ ^]^ Also, SF may be exploited to increase the Shockley–Queisser limit by reducing the thermalization losses in single junction silicon solar cells. The SF down conversion may be used to allow absorption of the solar high energy photons and transform them into excitons that better match the silicon band gap to give charge injection into the semiconductor in hybrid SF material/silicon devices. Additionally, due to the large triplet production yields, SF appears a more efficient and competitive strategy over intersystem crossing to design new effective photosensitizers for Reactive Oxygen Species generation in Photodynamic Therapy (PDT).^[^ ^14^, ^15^ ^]^ Both photovoltaics and PDT use the SF‐generated triplets. However, for practical implementation in photovoltaic devices and in PDT, SF systems should show high triplet energy for use as photosensitizers to lower band gap semiconductors (silicon 1.12 eV) or to singlet oxygen (0.98 eV).^[^ ^5^, ^7^, ^16^ ^]^ Following SF, triplet states are populated according to spin conservation, resulting in selective population of the T_0_ spin sublevel in the triplet manifold: such efficient initialization of the system in a pure spin state allows for applications in quantum information science as it fulfills the DiVincenzo criteria for a viable molecular spin qutrit.^[^ ^17^, ^18^, ^19^ ^]^ It is noteworthy that not only the SF‐generated triplets show wide interest for practical use, but also the high‐spin quintet multiexciton. This ^5^(TT) transient, which could be readily observed in purely organic systems and at temperatures higher than units of kelvin,^[^ ^20^, ^21^ ^]^ is also a promising candidate for quantum information science (QIS).^[^ ^21^, ^22^, ^23^ ^]^

SF has been generally observed in acene derivatives (pentacene and tetracene), which fulfill the rare energetic requirement for SF. However, acenes show major drawbacks such as limited light absorption as well as poor air and light stability, which limit their potential for applications. Therefore, in the last years, the search for new unconventional SF materials has become a hot topic.^[^ ^24^ ^]^ Intramolecular donor–acceptor interaction were highlighted in the literature as key design features for organic SF candidates, as from the mechanistic point of view virtual charge transfer excited state are believed to aid the SF process.^[^ ^25^, ^26^, ^27^, ^28^, ^29^, ^30^ ^]^ Among the most promising classes of SF chromophores alternative to acenes, quinoidal derivatives have recently emerged for their excellent stability and optimal features as organic semiconductors^[^ ^31^, ^32^, ^33^, ^34^, ^35^, ^36^, ^37^, ^38^ ^]^ and as they meet both the energetic and materials criteria for SF. However, none of the quinoidal materials proposed to date possess the appropriate triplet energy levels for direct energy transfer to silicon based solar cells (*T*_1_ *> *1.1 eV) so that efficient sensitization can occur. A recent theoretical analysis of *π*‐conjugated chromophores^[^ ^39^ ^]^ revealed that the energy gap between the first excited singlet (S_1_) and triplet (T_1_) states values can be tuned by varying structural motifs, namely minimizing the number of *π*‐electrons, reducing delocalization (through spatial separation and/or deplanarization) and optimizing interactions (inserting pro‐aromatic motifs). Some of these molecular strategies have found experimental validation through the synthesis of diketopyrrolopyrrole based^[^ ^40^ ^]^ and *p*‐azaquinodimethane (*p*‐AQM) based chromophores,^[^ ^41^ ^]^ whose estimated T_1_ energy levels are located at around 1.1 eV.

In a previous work from our groups,^[^ ^42^ ^]^ we have designed and synthetized *p*‐AQM derivatives with a symmetrical donor–acceptor–donor structure where 2,2′‐Bithiophene and 4‐Methoxybenzene were used as electron‐donor moieties bound at the opposite sites of a π‐conjugated *p*AQM electron‐acceptor core. Solubility and processability of these samples were optimized by introducing t‐butyl‐acetate (COOtBu) side chain substituents. We found evidence for ultrafast and quantitative SF in thin films of the bithiophene pAQM derivative while the anisole derivative was used to sensitize SF via FRET in mixed thin films of the two chromophores showing panchromatic absorption in the entire visible range.

**Scheme 1:**
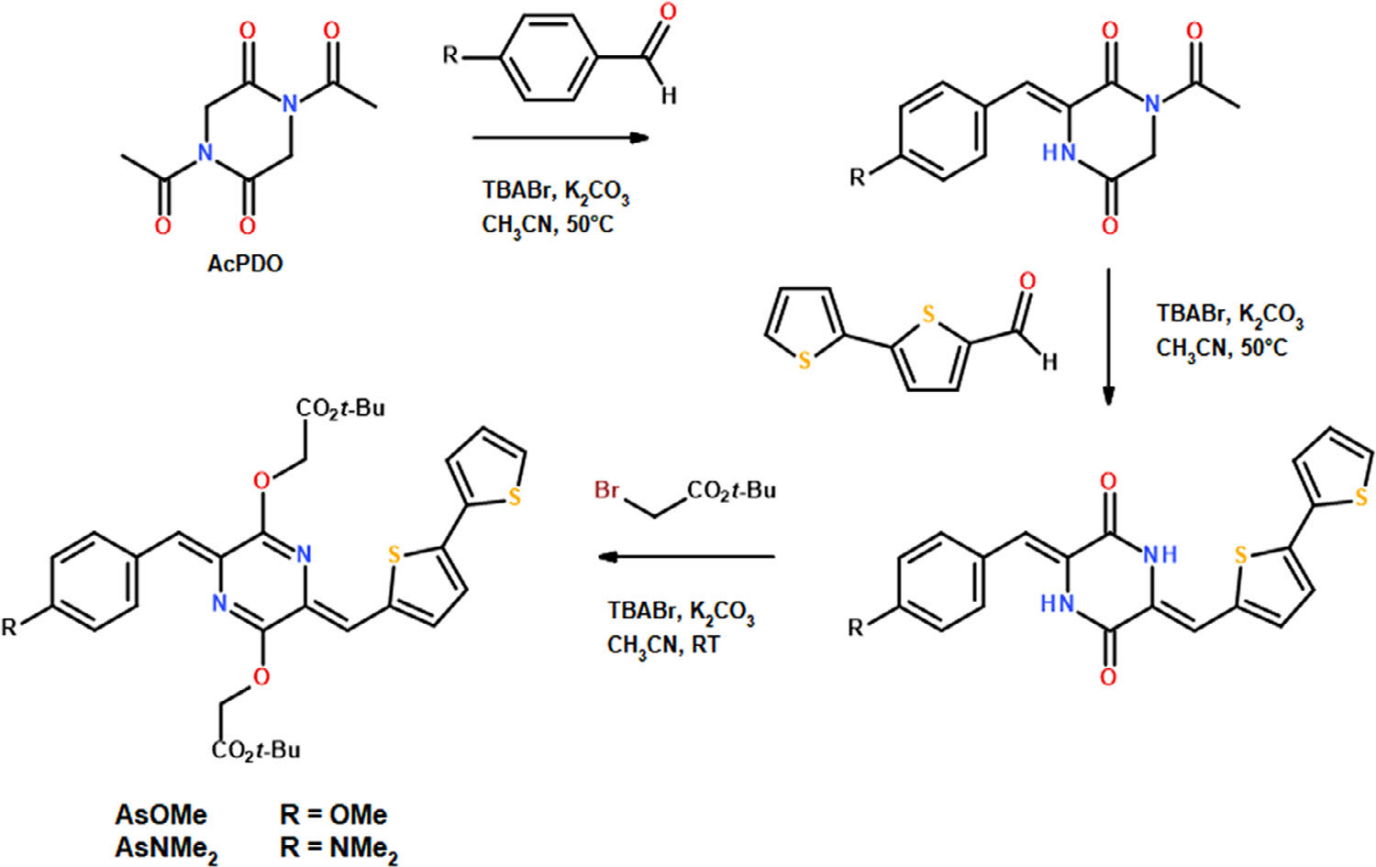
Synthesis of asymmetric pAQM derivatives, **AsOMe** and **AsNMe_2_**.

**Scheme 2:**
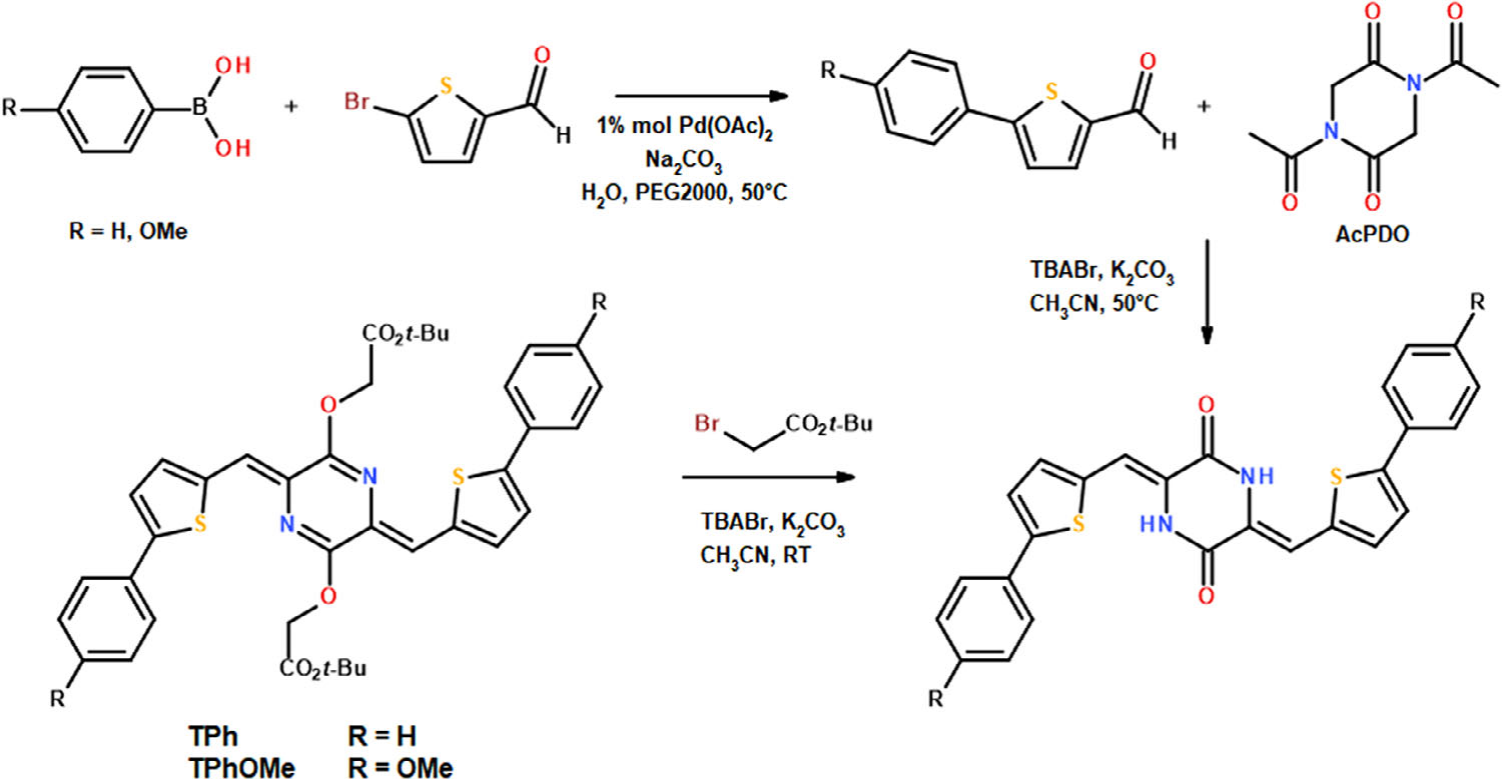
Synthesis of symmetric pAQM derivatives, **TPh** and **TPhOMe**.

In this study, we improve our sustainable protocol for the synthesis of new pAQM systems to unveil how structural modification of the aromatic/heteroaromatic moieties linked to the central pAQM core affects T_1_ (and S_1_) energy values. In order to minimize the overall number of *π*‐electrons, reduce delocalization, and optimize geometric interactions we mixed the 2,2′‐Bisthiophene and 4‐Methoxybenzene units in both asymmetric (**AsOMe** in Scheme 1) and symmetric (**TPhOMe** in Scheme 2) pAQM derivatives. Two analogous structures were also synthetized to uncover the effect of tuning more the push–pull character in these compounds: **AsNMe_2_** where the stronger electron donor NMe_2_ group relative to OMe was introduced in the asymmetrical backbone and **TPh** where the OMe electron donor substituents were removed from the symmetrical backbone (Schemes 1 and 2). The photobehaviour of these new materials was experimentally investigated both in solution and in thin film to disclose the impact of aggregation. Valuable information about the intramolecular charge transfer and the excited state energies was obtained through a joint TD‐DFT study. Moreover, the photoinduced excited state dynamics was studied in a synergic effort by employing both time resolved optical and magnetic spectroscopy for the first time for such unconventional SF materials.

## Results and Discussion

### Synthesis and Characterization

The structural modification to deal with, at first, was the reduction of the overall number of *π*‐electrons. Looking at the results previously obtained with the pAQM derivatives,^[^ ^42^ ^]^ we decided to replace one of the bisthiophene groups with a phenyl moiety; the insertion of electron donating groups on the phenyl ring, namely methoxy and dimethylamino, increases also the push–pull character of our pAQM derivatives. The new compounds (**AsOMe** and **AsNMe_2_**) were obtained through stepwise introduction of the different aromatic/heteroaromatic units on the central diketopiperazine core (AcPDO), while the sterically demanding t‐butylacetate group was chosen for the final alkylation step (Scheme 1); the overall synthetic pathway can be carried out in a safe solvent, such as Acetonitrile, and take advantage of the addition of both tetrabutyl‐ammonium bromide (TBABr), for phase transfer catalysis, and simple inorganic base (K_2_CO_3_).

Symmetric systems are the most common class of pAQM chromophores due to their straightforward synthetic pathway and, also in this case, we resorted to our green protocol to obtain the **TPh** and **TPhOMe** derivatives (Scheme 2), functionalized with 5‐phenyl‐thiophene moieties.

Phosphine‐free Suzuki reaction in a mixture of water and poly(ethylene glycol) (PEG) was a highly efficient procedure^[^ ^43^, ^44^ ^]^ for the coupling of 5‐Bromo‐2‐thiophenecarboxaldehyde with substituted phenylboronic acid in short times under mild conditions. These intermediate aldehydes undergo Knoevenagel condensation and insertion of the t‐butylacetate groups, with phase transfer catalysis.

The tailored functionalization of the p‐azaquinodimethane scaffold gave us two novel symmetrical pAQM derivatives and we expect these systems to have T_1_ energy level higher than the bisthiophene symmetrical derivatives^[^ ^42^ ^]^ due to reduced delocalization (the low steric hindrance between the hydrogen atoms in the *ortho* position does not hamper the rotation about the C_Thiophene_─C_Phenyl_ bond but the system moves away from planarity/flatness) and higher aromatic character given by the phenyl ring functionalized with electron donating group.

### Absorption and Fluorescence Properties

**Figure 1:**
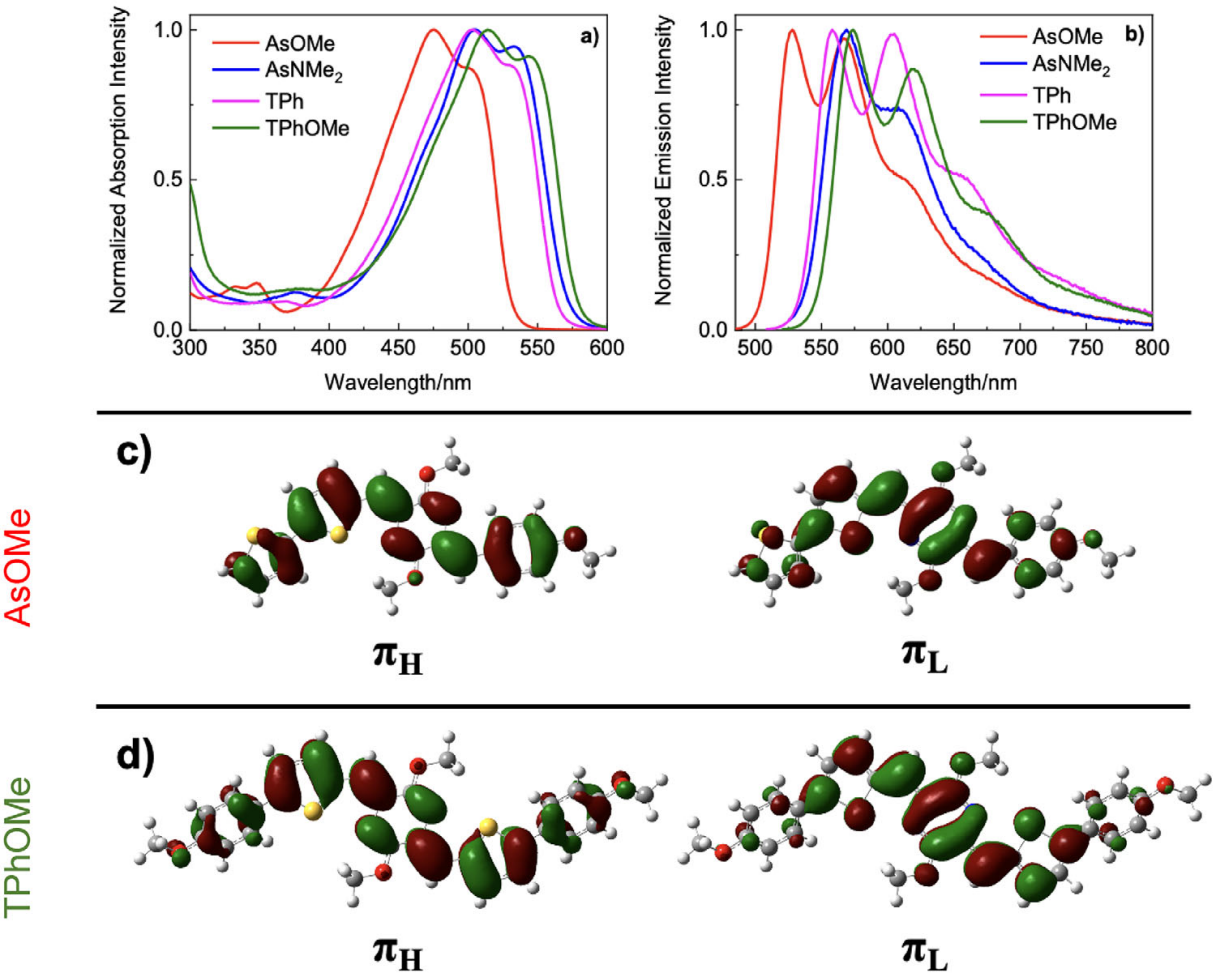
Absorption (a) and emission (b) spectra of all the compounds in toluene. Frontier molecular orbitals describing the S_0_→S_1_ absorption transition for **AsOMe** (c) and **TPhOMe** (d).

Figure 1 shows the absorption and emission spectra of the four investigated compounds in toluene (details in section 2 of Supporting Information). Both the absorption and the fluorescence spectra of **AsOMe** are significantly blue‐shifted relative to the other compounds, whose spectra appear to be more similar to each other. However, looking in more detail, the spectra of **TPhOMe** are slightly red‐shifted with respect to the spectra of **TPh**, in agreement with a more significant push–pull character when the methoxy substituents are introduced in the molecular structure. More importantly, the spectra of **AsNMe_2_** are largely red‐shifted relative to those of **AsOMe**, consistently with NMe_2_ being a stronger electron donor than the OMe group. The quantum chemical calculations predicted fairly well the experimental absorption and fluorescence spectra. The lowest excited singlet state S_1_ is mainly described by the HOMO–LUMO transition of π,π* nature in all cases (section 4 of the Supporting Information). When considering the ground state optimized geometry, the HOMO → LUMO implies some charge displacement from the lateral electron rich groups toward the central electron deficient pAQM, with the branch bearing the stronger OMe or NMe_2_ groups being more involved in the charge transfer than the bithiophene in the asymmetric structures (Figure 1). The molar extinction coefficients were found to be relevant for the investigated samples: 20 000–30 000 M^−1^cm^−1^ for the asymmetric and 45 000–60 000 M^−1^cm^−1^ for the symmetric derivatives (Table 1). This suggests that the investigated compounds may be efficient solar light harvester. The fluorescence quantum yields in toluene were found to be between 24% and 28% for **AsOMe**, **TPh**, and **TPhOMe**, while being only 2% for the **AsNMe_2_** compound likely due to its strongest intramolecular charge transfer character. Similarly, the fluorescence lifetimes measured by time correlated single photon counting were found to be 1–1.5 ns for **AsOMe**, **TPh**, and **TPhOMe**, while being shorter than the instrumental response function for **AsNMe_2_**. As a result, the fluorescence rate constant (*k*_F_ = *ɸ*_F_/*τ*_F_) was found to be ca. 2 × 10^8^ s^−1^ for all the molecules, suggesting that fluorescence is a fully allowed transition in toluene solution.

**Table 1:** Absorption and fluorescence properties of the compounds in toluene. ɛ: molar absorption coefficient of the ground state (in DMSO). *Φ*_F_: fluorescence quantum yield; *τ*_F_: fluorescence lifetime; *k*_F_: fluorescence rate constant.

	*Ɛ*_max_ / M^−1^cm^−1^	*ɸ*_F_	*τ*_F_ / ps	*k*_F_ / 10^8^ s^−1^
**AsOMe**	28 000	0.24	1090	2.2
**AsNMe_2_**	24 100	0.02	87*	2.3
**TPh**	45 800	0.26	1520	1.7
**TPhOMe**	60 500	0.28	1160	2.4

*From femtosecond transient absorption measurements.

The solvent polarity effect on the absorption and fluorescence properties was probed to obtain a further insight into the push–pull character of the investigated molecules. The results are reported in detail in the Supporting Information (section 3). The solvent effect on the absorption spectra was small in all cases. The observed solvatochromism of the emission spectra was also small for **AsOMe**, **TPh**, and **TPhOMe**, with the only apparent red shift having been observed in the more polar dimethylsulphoxide solvent. Differently, for **AsNMe_2_**, a significant fluorosolvatochromism was revealed, with the emission spectrum losing its vibrational structure and red‐shifting upon increasing the solvent dielectric constant. The solvatochromic results were analyzed in detail considering the dependence of the Stokes shift upon a function of the solvent properties (dielectric constant and refractive index). While no significant correlation was found in the case of **TPh**, a linear plot resulted from analyzing the other three compounds with slopes of 290 cm^−1^ for **AsOMe**, 1960 cm^−1^ for **AsNMe_2_**, and 440 cm^−1^ for **TPhOMe** (Figure S33). Considering Equation S1 (for the asymmetric structures) and Equation S2 (for the symmetric structure) described in the experimental section of the Supporting Information, the dipole moment difference between the excited and the ground state (Δ*µ*_CT_) was obtained (see Table S7). Δ*µ*_CT_ was found to be 7.4 D for **AsOMe**, 19 D for **AsNMe_2_**, and 9.0 D for **TPhOMe**. These data gave quantitative evidence for the strongest push–pull character of **AsNMe_2_**. An apparent solvent polarity effect was observed on the fluorescence quantum yields and lifetimes of **AsOMe**, **TPh**, and **TPhOMe**, with the yields and lifetimes being quenched in the more polar media. Among them, the most significant quenching effect (from 26% in toluene to 7% in dimethylsulphoxide) was observed for the asymmetric **AsOMe** derivative.

**Figure 2:**
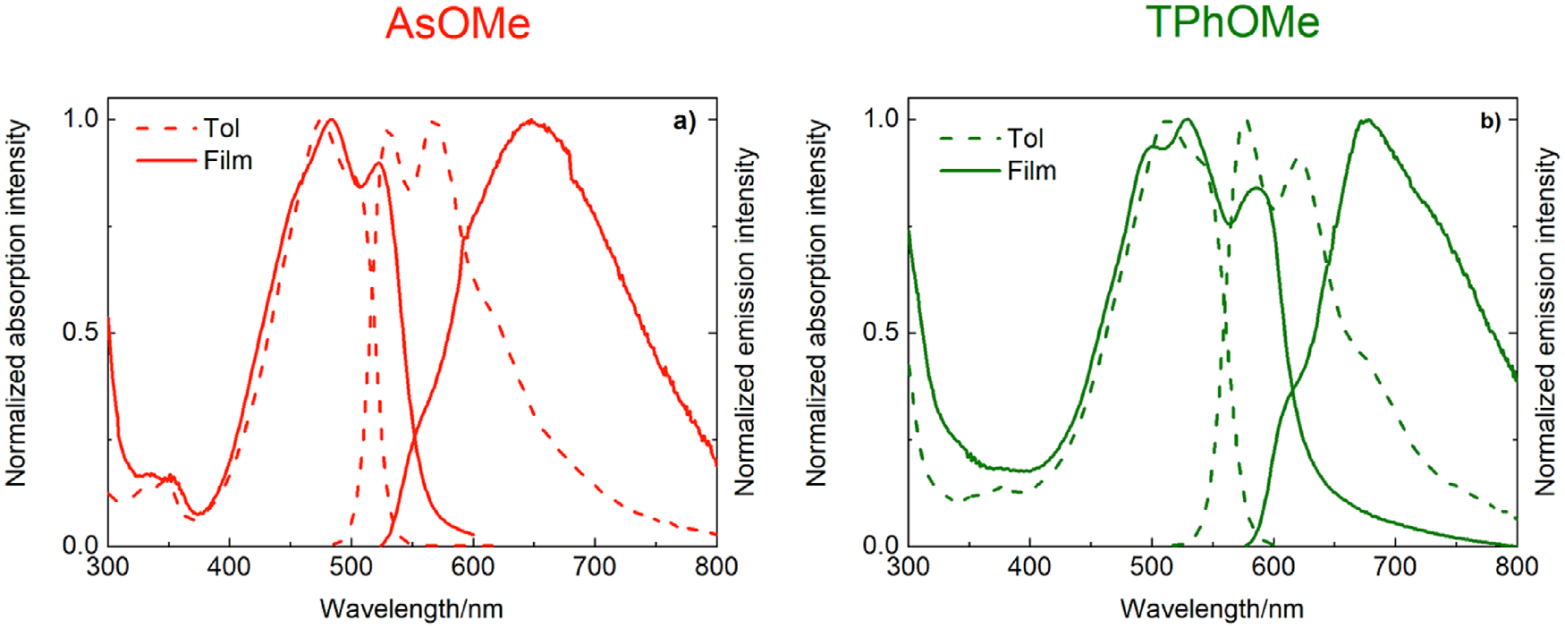
Comparison between absorption and emission spectra of **AsOMe** (a) and **TPhOMe** (b) in toluene (dashed) and thin film (solid).

The absorption and fluorescence properties were also investigated in thin films produced via spin coating (section 5 of the Supporting Information). When the spectra recorded in film were compared with those obtained in solution (Figure 2), the most significant changes that can be noted are the appearance of an absorption band due to the formation of J‐aggregates as well as the red‐shift of the emission spectrum on going from the solution to the solid state. The fluorescence kinetics, providing lifetimes of 1–1.5 ns for **AsOMe**, **TPh**, and **TPhOMe** in solution, were revealed to be overlapped to the instrumental response function for all the compounds in thin film (Figure S54). This shortening of the fluorescence lifetime points to the opening of a deactivation pathway competitive to the fluorescence in the solid state aggregates. Field‐emission scanning electron microscopy (FE‐SEM) images (Figure S118) indicate that the morphology of the spin coated films is related to the structure of the deposited chromophores, both in terms of the uniformity of the substrate coverage and general aspect of the layers. More regular spherical particles were evidenced for the asymmetric derivatives: **AsOMe** structures exhibit diameters on the order of a few micrometers, while **AsNMe**_2_ structures are smaller and sometimes have a donut/toroidal shape. On the other hand, the morphology observed for the symmetric **TPh** and **TPhOMe** was found to be generally more irregular, characterized by the presence of lamellar structures, thinner and more elongated for **TPhOMe** than for **TPh**.

### Femtosecond and Nanosecond Transient Absorption

**Figure 3:**
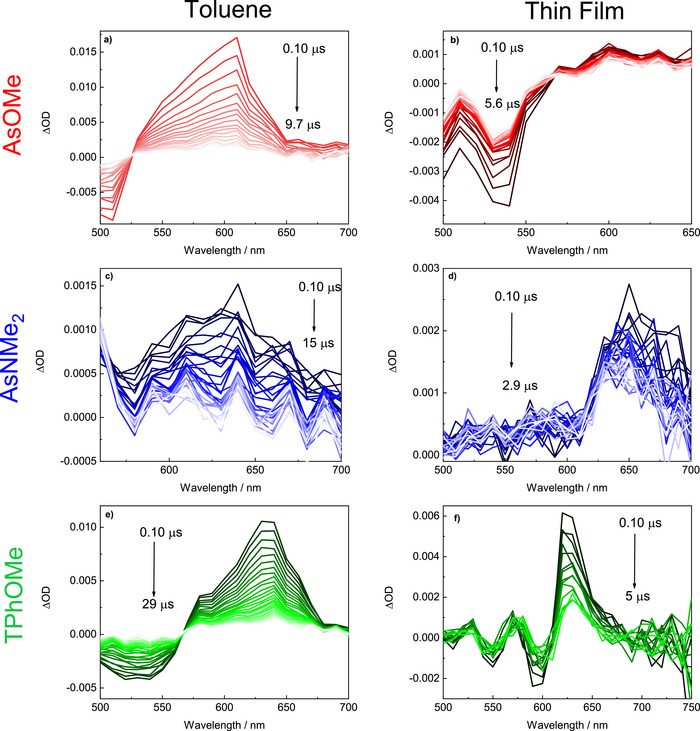
Nanosecond laser flash photolysis transient absorption spectra of **AsOMe**, **AsNMe_2_**, and **TPhOMe** in toluene (a, c, e) and thin film (b, d, f).

The involvement of non‐radiative deactivation pathways, such as the triplet production, was investigated via nanosecond transient absorption experiments. In toluene solution, a significant transient absorption signal was detected for all the investigated compounds (Figure 3 and section 6 of the Supporting Information). Such transient species exhibited spectra characterized by negative ground state bleaching (GSB) at shorter wavelengths and positive excited state absorption (ESA) at longer wavelengths peaked at 610 nm for **AsOMe**, 640 nm for **AsNMe_2_**, 620 nm for **TPh**, and 640 nm for **TPhOMe**. The triplet maximum absorption is slightly red‐shifted upon increasing the push–pull character (from **AsOMe** to **AsNMe_2_** and from **TPh** to **TPhOMe**). The computationally predicted triplet excited state absorption reproduced well the experimentally observed trend among the molecules in the series. This transient exhibited lifetimes of ca. 100 ns in air equilibrated solution and 7–11 µs in nitrogen purged solution (Table 2). Moreover, it could be produced via energy transfer by using a high triplet energy donor, such as benzophenone (*E*_T _= 2.99 eV), in sensitization experiments. It was thus assigned to the lowest triplet excited state T_1_. The sensitization experiments allowed an accurate evaluation of the triplet extinction coefficient (*ε*_T _= 15 000–40 000 M^−1^cm^−1^) and, coupled to relative actinometry measurements, of the triplet quantum yield. The triplet yield was found to be 30 ± 4% for **AsOMe**, 3.6 ± 0.5% for **AsNMe_2_**, 18 ± 3% for both **TPh** and **TPhOMe** in toluene (Table 2). The lower triplet and fluorescence yield of **AsNMe_2_** in solution are likely due to its largest intramolecular charge transfer degree. The nanosecond transient absorption experiments were also performed in thin film and these measurements interestingly showed large triplet excited state absorption upon laser excitation also in the solid state (Figure 3 and section 7 of the Supporting Information). The transient absorption spectra exhibit an additional negative GSB band at longer wavelengths compared to the solution spectra due to the J‐aggregate absorption and ESA signals peaked between 610 and 650 nm similarly to the solution results (Figure 3). It is noteworthy that the triplet lifetimes measured in the solid state showed values ranging between 1 and 11 µs. A shorter component was generally also necessary to achieve best fitting of the decay kinetics in the thin films, probably due to the inhomogeneous nature of the solid state aggregates.

**Table 2:** Triplet properties of the investigated compounds in toluene solution and thin film. *λ*_T_: maximum wavelength of triplet absorption spectrum; *τ*_T_: triplet lifetime in air equilibrated and nitrogen purged solutions; *ε*_T_: triplet molar absorption coefficient; *Φ*_T_: triplet quantum yield.

	Solution					Thin film		
Compound	*λ*_T_/nm	*τ*_air_/ns	*τ*_N2_/ns	*ε*_T_/M^−1^ cm^−1^	*Φ*_T_	*λ*_T_/nm	*τ*_T_/ns	*Φ*_T_ ^a)^
**AsOMe**	610	120	6900	42 100	0.30 ± 0.04	610	11 000	1.1 ± 0.2
**AsNMe_2_**	640	130	6500	12 900	0.036 ± 0.005	650	3900	2.1 ± 0.4
**TPh**	620	150	8000	39 800	0.18 ± 0.03	620	1800	1.2 ± 0.2
**TPhOMe**	640	130	11 400	30 700	0.18 ± 0.03	650	1100	1.0 ± 0.2

^a)^ From femtosecond transient absorption

**Figure 4:**
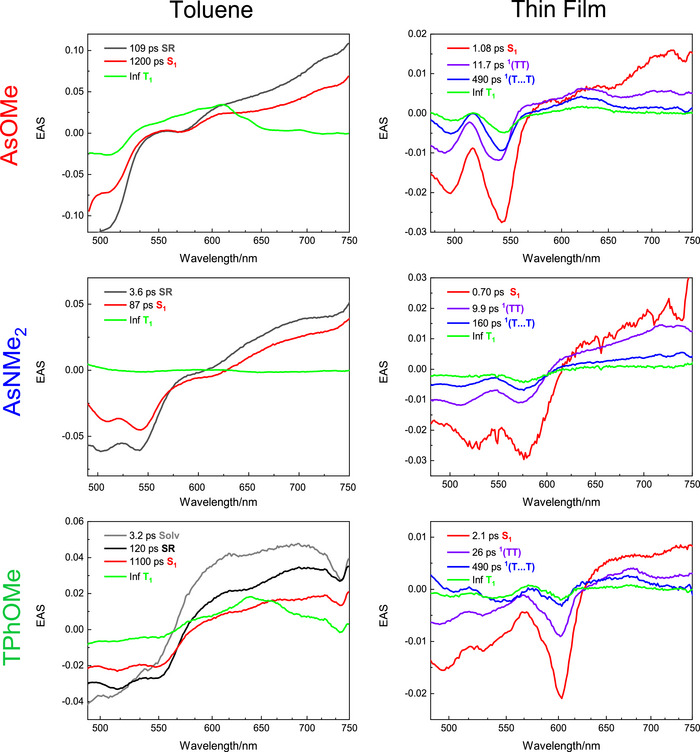
Evolution Associated Spectra (EAS) for **AsOMe**, **AsNMe_2_**, and **TPhOMe** in toluene and thin film from femtosecond transient absorption measurements.

Femtosecond transient absorption measurements were carried out to unveil the mechanism of triplet production both in solution and in the solid state (sections 9 and 11 of the Supporting Information). The femtosecond transient absorption spectra in solution evolve in time from a spectral shape characterized by a broad ESA extending above 700 nm and a negative GSB in the region 500–550 nm to a spectrum exhibiting a narrow ESA peaked at 610–640 nm matching the triplet absorption (Figure 4). Global fitting of the data resulted in 3–4 exponential components with lifetimes of few picoseconds assigned to solvation (Solv),^[^ ^45^ ^]^ of ca. 100 ps assigned to structural relaxation (SR), of ca. 1 ns assigned to S_1_ (87 ps for **AsNMe_2_**) and an infinite lifetime in the investigated time window of ca. 3 ns (Inf) assigned to T_1_ (see Table 3). The mechanism of triplet production in solution is therefore intersystem crossing occurring in ca. 1 ns for all compounds but **AsNMe_2_** for which the larger push–pull character may trigger a faster spin orbit charge transfer induced intersystem crossing (SOCT‐ISC).^[^ ^46^ ^]^ The femtosecond fluorescence up conversion experiments in toluene solution gave results in good agreement with the ultrafast transient absorption, as for the transient species in the singlet manifold (section 10 of the Supporting Information). When the ultrafast excited state dynamics was investigated in dimethylsulphoxide, a similar temporal evolution of the spectra was observed (sections 13 of the Supporting Information). However, the lifetime of the S_1_ state was generally shorter in this polar solvent than in toluene as expected for electronic states with charge‐transfer character. When measured in thin film, the femtosecond transient absorption spectra exhibited the sharp GSB band due to the J‐aggregates at 540 nm for **AsOMe** and ca. 600 nm for the other compounds. Moreover, the ESA portion of the spectra was initially a broad band in the region 700–750 nm (singlet ESA) but quickly evolved to give a narrow ESA peaked at 620–670 nm (triplet ESA). Indeed, the global fit revealed in all cases the presence of four exponential components. The first component exhibited a singlet‐like absorption spectrum and a lifetime of 0.70 ps for **AsNMe_2_** and 1.1 ps for **AsOMe**, while being longer for the symmetric derivatives (1.7 ps for **TPh** and 2.1 ps for **TPhOMe**). The second, third and fourth components all showed a triplet‐like Evolution Associated Spectrum and lifetimes of tens of ps, hundreds of ps and infinite in the investigated time window, respectively (Figure 4 and Table 3). The ultrafast triplet production is attributed to intermolecular singlet fission (SF) enabled in the thin film aggregates.^[^ ^42^, ^47^ ^]^ While the Inf component is assigned to the T_1_ excited state, the tens and hundreds of ps components are associated to the correlated triplet pair intermediates between S_1_ and T_1_: the spin entangled spatially correlated ^1^(TT) and the spin entangled but spatially decorrelated ^1^(T…T).^[^ ^48^ ^]^ Indeed, these transient species show the lifetime typical of a singlet and the absorption spectrum typical of a triplet excited state, disclosing its double nature.^[^ ^5^, ^6^, ^7^, ^49^ ^]^ Not only the correlated triplet pair formation but also its separation into independent triplets is faster for **AsNMe_2_**, the strongest push–pull system in the series (for which the largest Δ*µ*_CT_ was obtained from analysis of the solvatochromism). It is likely that the more significant intramolecular charge transfer degree in **AsNMe_2_** may play an important role in favouring SF, thus accelerating the dynamics of triplet production via virtual intramolecular charge transfer states.^[^ ^5^, ^27^, ^28^ ^]^ Analysis of the femtosecond transient absorption data allowed an estimate of the triplet quantum yield in thin film, according to a procedure reported in the literature^[^ ^27^ ^]^ and detailed in the Supporting Information (section 12). Even though this procedure contains many approximations, the estimated triplet quantum yield was found to be remarkable in all cases, even higher than 100% in some cases pointing to multiple triplet exciton generation phenomena. In particular, while the triplet yield was around 100% for **AsOMe**, **TPh**, and **TPhOMe** (110 ± 20%, 120 ± 20%, and 100 ± 20%, respectively), it resulted to be roughly 200% for the **AsNMe_2_** films. Our results suggest that the strongest push–pull character in **AsNMe_2_** triggers its fastest and most efficient SF among the molecules in this series.

**Table 3:** Femtosecond transient absorption results for all the investigated compounds in toluene and thin films: *τ*_TA_ lifetimes resulting from Global Analysis and relative assignment.

	*τ*_TA_ /ps				
	AsOMe	AsNMe_2_	TPh	TPhOMe	Assignment
Toluene	–	3.6	–	3.2	Solv.
	110	–	88	120	SR
	1200	87	1110	1100	S_1_
	Inf	Inf	Inf	Inf	T_1_
Film	1.1	0.70	1.7	2.1	S_1_
	12	9.9	25	26	^1^(TT)
	490	160	500	490	^1^(T…T)
	Inf	Inf	Inf	Inf	T_1_

**Figure 5:**
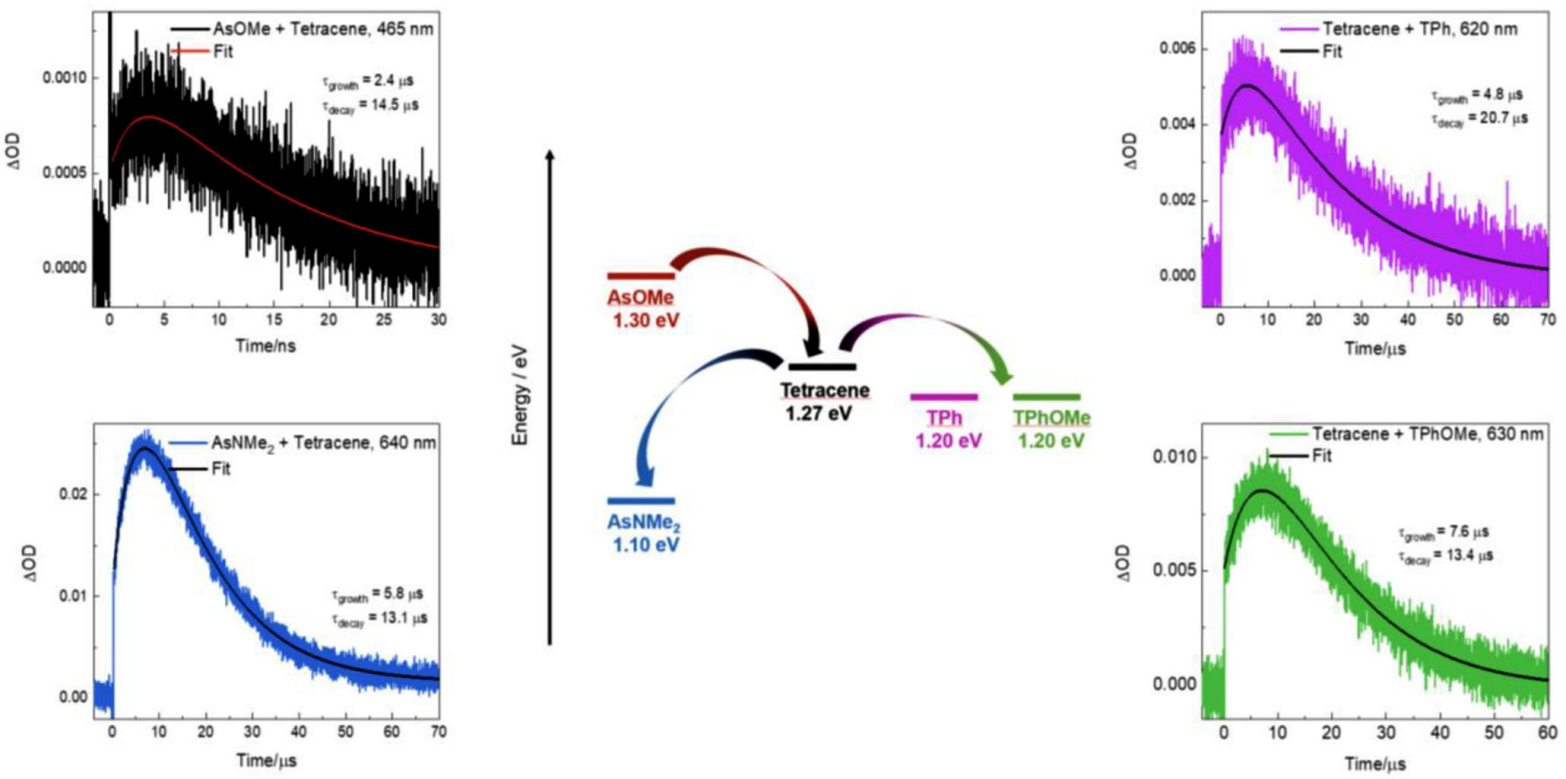
Sensitization experiment with the nanosecond laser flash photolysis by employing Tetracene together with asymmetric (**AsOMe** and **AsNMe_2_**, on the left graphs) and symmetric (**TPh** and **TPhOMe**, on the right graphs) compounds.

Quantum chemical calculations were performed to obtain information about the singlet (*E*_S_) and triplet (*E*_T_) excited state energies (Table 4). The obtained results in terms of Δ*E*_TT‐S_ = 2*E*_T_−*E*_S_ indicate that SF is actually thermodynamically feasible for all the investigated compounds. Sensitization experiments were also carried out through the nanosecond transient absorption (section 8 of the Supporting Information) to get an experimental insight into the triplet energy by using several sensitizers characterized by different triplet energies (Anthracene, 1.85 eV; Tetracene, 1.27 eV; Rubrene, 1.14 eV).^[^ ^50^ ^]^ It is noteworthy that while tetracene acted as triplet energy donor to **AsNMe_2_**, **TPh**, and **TPhOMe**, it functioned as triplet energy acceptor when employed together with **AsOMe** (Figure 5). This finding suggests that the triplet energy is above 1.27 eV for **AsOMe**, while being lower than 1.27 eV for all the other compounds. Anthracene was used as triplet energy donor to **AsOMe**, while all the other molecules could also be sensitized by Rubrene as energy donor. When the quenching rate constant derived considering the triplet donor lifetime in the absence and in the presence of the acceptor was lower than the diffusional rate constant, the triplet energy was estimated through the Sandros equation.^[^ ^7^, ^51^ ^]^ When possible (**TPh** and **TPhOMe**), the triplet energy was obtained as the average of two distinct sensitization experiments. These triplet energy values should be compared to the Franck–Condon computationally predicted triplets, in the sensitization experimental conditions. The experimental **AsOMe** triplet energy (1.3 eV) was found to be larger than for the other compounds (1.1 eV for **AsNMe_2_**, 1.2 eV for **TPh** and **TPhOMe**). By estimating also, the singlet excited state energy as the crossing between the absorption and fluorescence spectra, it was possible to evaluate, also experimentally, the thermodinamic feasibility of SF for these compounds (Δ*E*_TT‐S,exp_ = 2*E*_T,exp_−*E*_S,exp_ in Table 4). The SF was experimentally predicted to be slightly exothermic for **AsNMe_2_** (Δ*E*_TT‐S,exp_ = −0.08 eV), while being still thermodynamically favorable but slightly energetically up‐hill for the other compounds, particularly for **AsOMe** (Δ*E*_TT‐S,exp_ = +0.20 eV). Given that the femtosecond transient absorption measurements have demonstrated the occurrence of SF for all the investigated compounds in thin film, we believe that the ultraviolet (355 nm) or blue (400 nm) laser excitation in our transient experiments may allow to overcome the activation barrier for the process even in the case of **AsOMe**. The SF‐generated highly energetic triplet excitons are really appealing in view of photovoltaic applications as they appear to be slightly above the silicon band‐gap.^[^ ^40^, ^52^ ^]^ Very interestingly, our experimental results for **AsOMe** suggest higher energetic triplet excitons (1.3 eV) relative to most literature SF‐materials (e.g., pentacene and indeed tetracene).^[^ ^53^, ^54^ ^]^

**Table 4:** Experimental (exp) and computationally predicted (th) energies for the excited states (*E*_S_ singlet and *E*_T_ triplet).

Compound	*E*_S,exp_ / eV	*E*_T,exp_ / eV	Δ*E*_TT‐S,exp_ /eV	*E*_Srel,th_/ eV	*E*_T(rel/FC),th_/ eV	Δ*E*_TT‐S,th_ /eV
**AsOMe**	2.40	1.3	+0.20	2.10	1.11/1.26	+0.12
**AsNMe_2_**	2.25	1.1	−0.05	2.03	1.10/1.23	+0.17
**TPh**	2.27	1.2	+0.13	1.98	1.03/1.14	+0.08
**TPhOMe**	2.21	1.2	+0.19	1.97	1.03/1.14	+0.09

*Δ*E*_TT‐S,exp_ = 2*E*_T,exp_−*E*_S,exp_; Δ*E*_TT‐S,th_ = 2*E*_Trel,th_−*E*_Srel,th_ were *rel* stands for relaxed and *FC* for Frank–Condon excited states

### Time Resolved Electron Paramagnetic Resonance

Further evidence that these systems can work as SF active materials, with different activation energy barriers, was gained using Time Resolved Electron Paramagnetic Resonance (TR‐EPR). TR‐EPR spectroscopy is a powerful technique to study paramagnetic states populated after photoexcitation of the systems.^[^ ^55^ ^]^ TR‐EPR can be used to determine the Zero Field Splitting (ZFS) parameters, *D* and *E* as reported in the Supporting Information, related to the spin distribution in the system,^[^ ^56^ ^]^ while the spin polarization of the TR‐EPR spectrum sheds light into the population mechanism of such states; finally, the time evolution of the TR‐EPR signal gives information on spin dynamics and sublevel population decay.^[^ ^57^, ^58^, ^59^ ^]^

**Figure 6:**
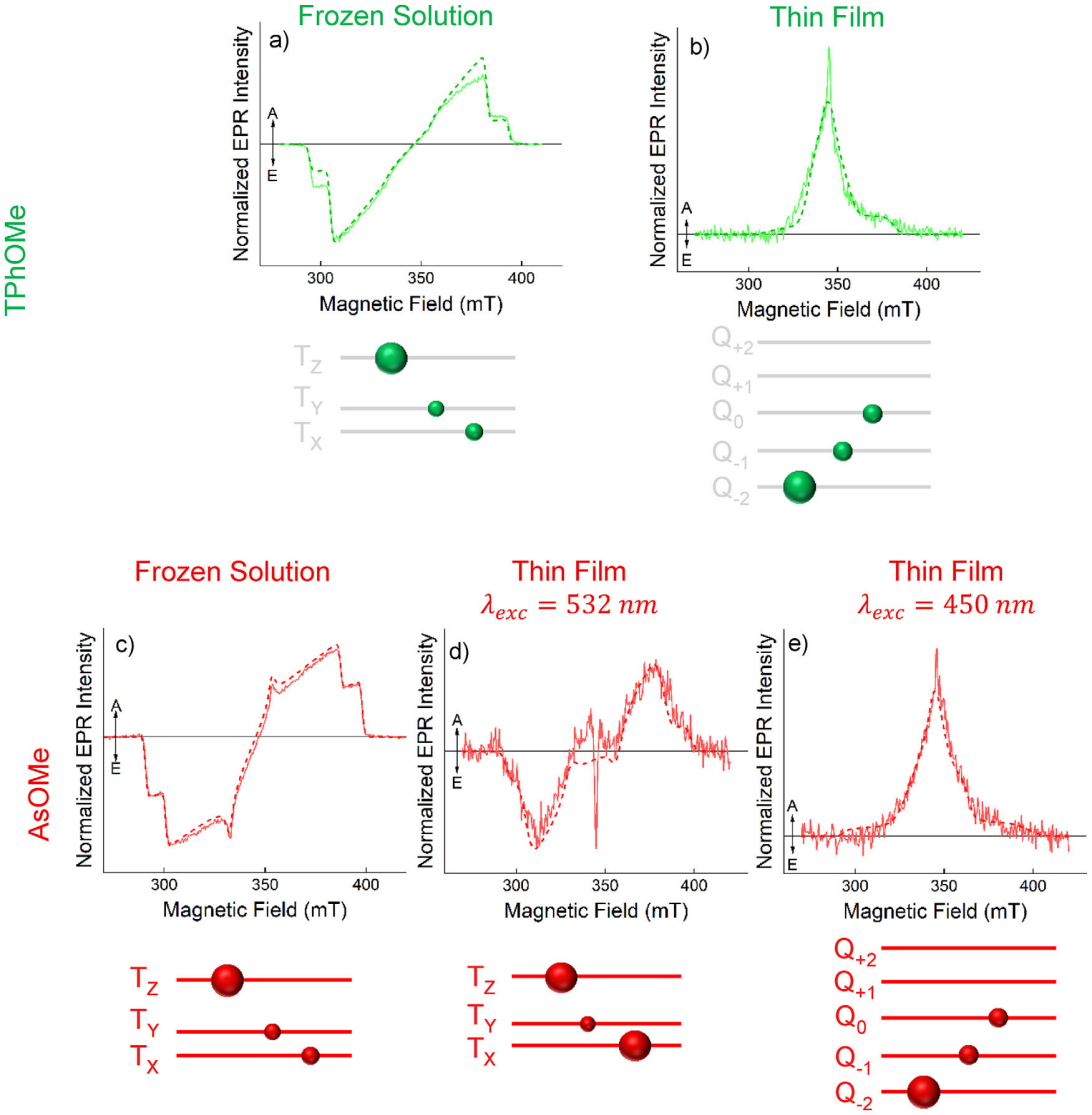
X‐band TR‐EPR spectra (solid line) and their simulations (dashed line) in solution a), c) and in film b), d), e) at 80 K for **TPhOMe** a), b) and **AsOMe** c), d), e) after photoexcitation with 2.5 mJ/shot (solution) and 5 mJ/shot (film) at 532 nm. In the case of **AsOMe** also the TR‐EPR spectrum in film following excitation with 2 mJ/shot at 450 nm is shown (d). A schematic representation of the sublevel populations is also reported.

TR‐EPR characterization in frozen solution of the four pAQM derivatives shows that similar triplet state spectra are detected for all the compounds (Figure 6a,c, section 14.1 of the Supporting Information and Figures S103–S106). In all cases, an initial *eeeaaa* spin polarization pattern was registered (*a* = enhanced absorption and *e *= emission) at early delay after flash (DAF) values, which later evolves to *aaaeee*. The initial spin polarization is due to selective population of the triplet sublevels at ISC from the excited singlet state. The inversion of the spin polarization pattern at later times has already been observed in other systems, e.g. carotenoids and photoconductive polymers,^[^ ^57^, ^59^ ^]^ and it can be ascribed to anisotropic depopulation of the triplet state sublevels. The time constants obtained from the simulation of the TR‐EPR data (Table S27) show a faster decay of the *Z* sublevel, which is also preferentially populated at ISC, followed by a slower decay of the population of the *X* and *Y* sublevels.

Simulations, based on the spin Hamiltonian of a triplet state reported in section 1.3.2 of the Supporting Information, allow relative triplet sublevels population parameters, decay kinetics and ZFS parameters to be derived (see Table S27). ZFS parameters for the different compounds are very similar and the *D < 0* assignment is supported by the simulation of the magnetophotoselection (MPS) effect (Figure S107), which allows to determine the orientation of the optical transition dipole moment (TDM) in the ZFS frame by using linearly polarized light parallel and perpendicular to the external magnetic field.^[^ ^60^ ^]^ TD‐DFT calculations (Figures S47–S50) show that the S_0_→S_1_ TDM lies in the molecular plane, directed toward the donor moieties, similarly the triplet spin density which is mainly localized on the donors, see Figure S51. Experimentally, an increase in the intensity of the transition along the *Z* ZFS canonical axis is observed when light is polarized parallel to the external magnetic field, and a decrease when it is polarized perpendicular to the external magnetic field. This suggests, and it is confirmed by spectral simulations, that the *Z* axis of the ZFS tensor is aligned with the TDM in the molecular plane, resulting in a negative *D* value (prolate electron distribution). Quantitatively, the spherical angles describing the orientation of the TDM in the ZFS frame, obtained from simulation of the MPS effect, are listed in Table S27.

For **AsOMe** and **AsNMe_2_**, the absolute value of *D* is higher by ∼100 MHz, which might suggest an additional charge transfer (CT) contribution in the triplet state. TR‐EPR results in a polar glass, (ethanol/methanol 3:2 mixture) where CT contributions are expected to be enhanced, show instead a lower absolute value of *D* (Figures S108 and S109), indicating that the triplet state of the isolated molecules has little to no CT character. Thus, the lower *D* ZFS parameters for the asymmetric compounds probably arise from lower delocalization of the spin density than in **TPh** and **TPhOMe** as shown in Figure S51.

TR‐EPR experiments on thin films gave different results for the pAQM derivatives. The TR‐EPR spectrum for a thin film of **TPhOMe** is reported in Figure 6b; this spectrum is very different from the triplet TR‐EPR spectrum of **TPhOMe** obtained in frozen solution, Figure 6a. The spectrum in thin film shows a narrow, ∼2 mT wide, absorptive signal at ∼345 mT from a photogenerated radical (not simulated), as observed in other SF materials,^[^ ^61^, ^62^ ^]^ and a purely absorptive feature with a width of ∼60 mT, with a shoulder at ∼370 mT, showing a reduced spectral range compared to the triplet state spectrum obtained in frozen solution, which is ∼100 mT wide. This reduction of the spectrum width can be explained considering the population of a quintet state, deriving from two coupled triplet states generated by SF, for additional details on the spin Hamiltonian of a pair of interacting triplets can be found in section 1.3.2 of the Supporting Information. Given two identical triplet states with *D_A_* = *D_B_*  = *D_T_*  as the ZFS parameter, the coupled homo‐triplet pair ^5^(TT), in the strong exchange coupling regime (*J* ≫ *D*), will have a ZFS parameter $D=\frac{D_{T}}{3}\;$,^[^ ^63^ ^]^ which is the maximum ZFS reduction observed when the two triplets are collinear. The lower ZFS interaction, responsible for the spectral feature, agrees with the observed narrower spectrum obtained in film.

We can assign the TR‐EPR spectrum of **TPhOMe** in thin film to the ^5^(TT) triplet pair, showing that SF becomes active for **TPhOMe** when aggregates are present. Interestingly, the simulation of the TR‐EPR spectrum shows that there is selective population of the Q_‐2_, Q_‐1_, and Q_0_ sublevels. Evidence for SQ_‐1_ and SQ_‐2_ mixing in thin films of SF active materials was already observed by Nagashima et al,^[^ ^64^ ^]^ we expect a similar mechanism to be active in the **TPhOMe** film. It is also worth noting that Nagashima et al. observed an additional triplet state spectrum attributed to uncorrelated triplet states in which the T_0_ and T_‐1_ sublevels are populated once the quintet state has decayed. In our case, this contribution was not observed probably because of the fast decay of the ^5^(TT) and the contribution from ISC populated triplet states which becomes visible once the ^5^(TT) has disappeared, vide infra.

The simulation of the TR‐EPR spectrum of the strongly coupled triplets was performed using a *J* = 20 GHz, which was found to be the lower limit for the strong exchange coupling condition; higher *J* values do not change the outcome of the simulation. We also used the ZFS parameters of the isolated triplets, derived from simulations of the TR‐EPR spectra in solution, with the ZFS frames of the two triplets deviating from collinearity. A dipolar interaction of 150 MHz between the triplets, (${\hat{\vec{S}}}_{A}\underline{D_{AB}}{\hat{\vec{S}}}_{B}$ in Equation S2) was employed for the simulation of the thin film spectra; this choice for the dipolar interaction was based on $D_{AB}\propto \frac{52.041\;(\frac{MHz}{nm^{3}})}{r^{3}}$, where *r* is the distance between the two triplets in the pair; the distance was estimated from the *J* = 20 GHz lower limit on the exchange interaction and its dependence on *r* as reported for aliphatic systems where no direct conjugation is expected.^[^ ^65^, ^66^ ^]^ Examples of how the simulations of the ^5^(TT) spectrum change using collinear and non‐collinear ZFS frames with different (α, β, γ) Euler angles and their effect on the sublevel transitions are reported in Figure S110. In the case of γ, after a rotation around the Y ZFS axis given by β  =  60°, we observe that the simulated spectrum becomes slightly narrower and does not properly reproduce the experimental spectrum for γ > 20°, an example of the simulation obtained for (α, β, γ)  =  (0, 60°, 30°) is shown in Figure S110e, along with simulations in which (α, β, γ)  =  (20, 60°, 0°) and (α, β, γ)  =  (20°, 60°, 20°) Euler angles were used. The simulations show that the optimal Euler angles configuration (α,  β,  γ) =  (0°,  62°,  0°) is necessary to correctly position the Q_‐2_→Q_‐1_ transition and to properly reproduce the signals at 350 mT and 336 mT arising from shoulders in the Q_0_→Q_+1_ and Q_‐1_→Q_0_ transitions respectively. Further details on the simulation parameters employed for the ^5^(TT) spectra can be found in Section 14.2 of the Supporting Information.

An EPR spectrum with a similar spectral range, and therefore ZFS parameters, population of the Q_‐2_, Q_‐1_, and Q_0_ sublevels of ^5^(TT) and non‐collinear ZFS frames, has also been observed and simulated for the **AsNMe_2_** film, Figure S111a, showing that SF is active in **AsNMe_2_** aggregates as well and the quintet state due to correlated triplets is also populated. Similarly, to **TPhOMe**, we also detected an ISC populated triplet state spectrum at later times, vide infra.

On the other hand, in the case of **AsOMe** film, an ISC populated triplet state was observed following photoexcitation at 532 nm. The TR‐EPR spectrum is reported in Figure 6d, it shows lower absolute values of the ZFS parameters with respect to the spectrum in frozen solution, Figure 6c. This reduction of the ZFS parameters (∼70 MHz for *D* and ∼90 MHz for *E*) might be due to delocalization of the triplet exciton in the aggregate.^[^ ^67^ ^]^ In film we also observe an *eeeaaa* spin polarization pattern at early DAFs which evolves to *aaaeee* in time, similarly to what occurs in frozen solution, Figures 6c and S103, suggesting that anisotropic decay of the triplet sublevels is still active even in the film. While these results would lead to interpretation that SF is not occurring in this system, evidence for SF in **AsOMe** aggregates can be obtained when the same film is photoexcited with higher energy photons. A TR‐EPR spectrum with quintet state features similar to the ones obtained for **TPhOMe** and **AsNMe_2_** is observed when photoexcitation is performed at 450 nm, Figure 6e.

Lastly, a triplet state is observed in the TR‐EPR spectrum of the **TPh** film, Figure S111b. In this case, SF is still active, but an uncoupled triplet is observed: the polarization pattern *eaaeea* is not compatible with ISC population of the triplet state but arises from selective population of the T_0_ triplet sublevel, deriving from the spin‐conservation condition in SF. The asymmetric shape of the TR‐EPR spectrum, resulting in net integrated absorption intensity, demonstrates that additional population of the T_‐1_ sublevel is occurring, as reported in the simulation parameters (Table 5). Population of the T_‐1_ state after SF is possible when SQ_‐1_ and SQ_‐2_ mixing is active as described by Tayebjee et al.^[^ ^8^ ^]^ and Nagashima et al.^[^ ^64^ ^]^ The TR‐EPR spectrum of the quintet state might not be visible because of fast decay in **TPh** films. Furthermore, the polarization pattern observed for **TPh** suggests that *D < 0*; thus, a similar triplet state distribution is observed both in solution and in film.

**Table 5:** ZFS (*D* ± 8 MHz, *E* ± 5 MHz) and population of the sublevels (*p_i_* ± 0.02) for the simulation of the X‐band TR‐EPR spectra in frozen solution and film. ***p_X_:p_Y_:p_Z_*** refers to triplet states populated via ISC, ***p_‐1_:p_0_:p_+1_*** to free triplets populated after SF and ***p_‐2_:p_‐1_:p_0_:p_+1_:p_+2_*** to the populations of the ^5^(TT). Further details can be found in the Supporting Information.

	Compound	*D* (MHz)	*E* (MHz)	*p_X_*: *p_Y_*: *p_Z_*	*p*_−1_: *p*_0_: *p*_+1_	*p*_−2_: *p*_−1_: *p*_0_: *p*_+1_: *p*_+2_
Frozen solution	**AsOMe**	−1495	−304	0.29:0.26:0.45	–	–
	**AsNMe_2_**	−1504	−306	0.32:0.14:0.54	–	–
	**TPh**	−1392	−262	0.31:0.15:0.54	–	–
	**TPhOMe**	−1384	−260	0.34:0.12:0.54	–	–
Thin film	**AsOMe (532 nm)**	−1424	−214	0.39:0.2:0.41	–	–
	**AsOMe (450 nm)**	−1495	−304	–	–	0.50:0.26:0.24:0:0
	**AsNMe_2_**	−1504	−306	–	–	0.48:0.28:0.24:0:0
	**TPh**	−1284	−238	–	0.26:0.74:0	–
	**TPhOMe**	−1384	−260	–	–	0.53:0.25:0.22:0:0

For the all the four pAQM derivatives SF features are accompanied by the observation of the spectra of ISC populated triplet states, thus showing that different deactivation pathways occur in the films starting from the singlet excited state.^[^ ^68^ ^]^ For **TPhOMe**, **AsNMe_2_**, and **TPh**, these ISC triplet state spectra can be observed at later DAFs and they are all characterized by an *aaaeee* spin polarization pattern, Figures S112–S114. In the case of the **AsOMe** film, where only the ISC triplet state can be observed when exciting at 532 nm, the initial *eeeaaa* polarization pattern evolves to *aaaeee* in time, we expect a similar mechanism to be active in the other aggregates. In fact, transients extracted at field positions in which no ^5^(TT) contributions are present, e.g. at 310 mT for **TPhOMe** (see Figure S115d), show the presence of a weak emissive signal which evolves to absorptive at later DAFs as expected due to polarization inversion. Furthermore, these triplet states exhibit a lower absolute value of *D* compared to the triplets observed in frozen solutions. This reduction is probably due to partial delocalization of the triplet exciton in the aggregate resulting in a lower ZFS interaction.^[^ ^67^ ^]^ For those systems in which correlated triplets are observed, while ISC polarization patterns are always visible, the presence of spectral features from uncorrelated triplet states deriving from SF was not discernible.

Another possible contribution to TR‐EPR spectra at later times could come from triplet‐triplet annihilation (TTA), as also suggested by the nanosecond transient absorption results. TTA can occur if SF is exergonic and contributes to the TR‐EPR spectrum with a delayed net polarization, which is emissive if the triplets are antiferromagnetically coupled and absorptive if they are ferromagnetically coupled, because of the spin selectivity of TTA.^[^ ^69^, ^70^, ^71^ ^]^ Yet, no net polarization is observed even in the case of the ISC populated triplets; thus, we rule out contributions arising from TTA.

Since SF was observed at room temperature via transient absorption, we also conducted TR‐EPR measurements on films at higher temperature (*T* = 250 K, Figure S116) on **AsNMe_2_** and **TPh** films. The results resemble those obtained at 80 K, confirming SF is still occurring and can be detected via TR‐EPR spectroscopy at temperatures approaching room temperature.

Finally, it is worth noting that TR‐EPR spectroscopy also confirms the sensitization experiments. For the **AsOMe** film photoexcitation at 532 nm results in population of a triplet state via ISC, while photoexcitation at 450 nm yields a spectrum showing both the features of the ^5^(TT) and the ISC contribution: SF is endergonic for this molecule, in agreement with the optical and computational results listed in Table 4. On the other hand, the invariance of the TR‐EPR spectrum for the **TPh** film, after photoexcitation at 532 nm and at the maximum absorption of the J‐aggregate band (at 600 nm), as shown in Figure S117b, indicates that SF is exergonic or only slightly endergonic. In the case of **AsNMe_2_**, when photoexcitation is performed at 600 nm a triplet state spectrum is observed instead of the spectrum with the features of the ^5^(TT), Figure S117a. The *eaaeea* spin polarization of this triplet state spectrum due to the uncorrelated triplet states deriving from SF, found also for **TPh**, proves that SF is still active and, thus, it is also in this case exergonic or only slightly endergonic. The observation of an uncorrelated triplet state at 600 nm and a correlated triplet pair at 532 nm is probably due to the superexchange mechanism in SF.^[^ ^72^, ^73^ ^]^ That is, the population of the high energy ^5^(TT) is possible at 532 nm because SF proceeds from higher CT states in the aggregate which are accessible at this wavelength, whereas at higher wavelengths (lower energies) this state is not accessible; thus, the initial ^1^(TT) undergoes spin dephasing from the coupled triplet pair to form uncoupled non‐interacting triplet states which are free to diffuse in the film. In fact, this behavior in **AsNMe_2_** film is consistent with the faster and more efficient SF observed via transient absorption.

## Conclusion

In this work, we synthetized new donor‐acceptor‐donor para‐azaquinodimethane (pAQM) derivatives through an optimized sustainable protocol and investigated how tuning the intramolecular push–pull character affects their capability of undergoing SF in solution as well as in thin film. The bithiophene and methoxybenzene/dimethylaminobenzene were used as electron‐donors at opposite sides of the pAQM electron‐acceptor in asymmetric structures (**AsOMe** and **AsNMe_2_**), whereas two 5‐phenylthiophene moieties were symmetrically bound to the pAQM core in **TPh** and **TPhOMe**.

The excited state dynamics was investigated in a joint effort through time resolved optical and magnetic spectroscopies with the help of TD‐DFT simulations. Our femtosecond transient absorption results showed that intermolecular SF is activated in the solid state aggregates of these organic materials, where a peculiar intermediate species was observed characterized by a triplet‐like absorption spectrum and by a lifetime typical of a singlet excited state and thus associated to the correlated triplet pair ^1^(TT). The fastest (700 fs) and most efficient (ca. 200% triplet yield) SF was revealed for the compound with the largest push–pull degree (**AsNMe_2_**). TR‐EPR measurements undisclosed the spectral signatures of ISC‐generated triplets for all the isolated molecules in frozen solution, confirming the nanosecond ISC dynamics observed in transient absorption measurements. By contrast, TR‐EPR gave evidence for the occurrence of SF upon aggregation in film. Indeed, the spectrum of the uncorrelated triplet state generated via SF was detected in the solid state for the compound characterized by the lowest push–pull degree (**TPh**). Spectral signatures assigned to the quintet multiexciton SF intermediate were obtained for the **AsOMe**, **AsNMe_2_**, and **TPhOMe** films. The high‐spin photoinduced ^5^(TT), observed in this study for the first time, to the best of our knowledge, for unconventional SF materials, appears to be a viable molecular spin qudit for quantum information science and technology applications.

SF was found to occur in ca. 1 ps and with ca. 100% triplet yield in the films of **AsOMe**, for which the nanosecond transient absorption sensitization experiments interestingly demonstrated the highest triplet energy (1.3 eV) among the molecules in the series, higher than the energy of SF‐generated triplets for literature conventional materials (e.g., tetracene). It is noteworthy that a wavelength effect was uncovered through the EPR study for **AsOMe** in the solid state: SF was actually enabled only upon a high‐energy blue laser excitation, while being not triggered by green pulses, in agreement with a slightly endergonic SF for this compound. This finding opens up intriguing perspectives for photovoltaic applications of these novel stable chromophores toward silicon matched SF.

## Supporting Information

The Supporting Information reports details about the synthesis and characterization, the sample preparation and the employed experimental techniques and computational methods; additional data about the absorption and emission properties in solution and in thin film; results of the quantum mechanical calculations; additional nanosecond transient absorption results in solution and in thin film; results of the sensitization experiments; additional femtosecond transient absorption and fluorescence up conversion results in solution and in thin film; detailed procedure employed for the triplet yield calculation; additional time resolved electron paramagnetic resonance results in solution and in thin film; scanning electron microscopy results.

## Conflict of Interests

The authors declare no conflict of interest.

## Supporting information



Supporting Information

## Data Availability

The data that support the findings of this study are available in the supplementary material of this article.
