# Supplementary material for: Singlet Fission in Push–Pull Para‐Azaquinodimethane Films under the Gaze of Time Resolved Optical and Magnetic Spectroscopy
Source: Angew Chem Int Ed Engl. 2025 Dec 26;65(6):e20838. doi: 10.1002/anie.202520838 (PMC12865250; doi:10.1002/anie.202520838)
Supplement: Supplementary file 1 — Supporting Information [file ANIE-65-e20838-s001.docx]

**Supporting Information**

Singlet Fission in Push-Pull para-Azaquinodimethane Films Under the Gaze of Time Resolved Optical and Magnetic Spectroscopy

Martina Alebardi,^[a],‡^ Angelo Carella, ^[b],‡^ Alessandro Grasso,^[c],‡^ Enrico Sorbelli,^[a]^ Francesco Lazzarin,^[b]^ Cristina Munzone,^[c]^ Cosimo Gianluca Fortuna,^[c]^ Fausto Elisei,^[a]^ Anna Spalletti,^[a]^ Carmela Bonaccorso,^[c],^* Marilena Di Valentin,^[b],^* Benedetta Carlotti^[a],^*

**Table of Contents**

[1. Experimental section: 2](#_Toc209286075)

[1.1 Synthesis, NMR and MS Characterization 2](#_Toc209286076)

[1.2 Photophysical characterization: materials and methods 21](#_Toc209286077)

[1.3 EPR characterization 22](#_Toc209286078)

[**1.3.1 Materials and Methods** 22](#_Toc209286079)

[**1.3.2 The Spin Hamiltonian** 23](#_Toc209286080)

[1.4 Thin film preparation 24](#_Toc209286081)

[1.5 Quantum mechanical calculations 24](#_Toc209286082)

[2. Absorption and emission in solution: structure effect 25](#_Toc209286083)

[3. Absorption and emission in solution: solvent effect 26](#_Toc209286084)

[4. Quantum mechanical calculation results 30](#_Toc209286085)

[4.1 AsOMe 30](#_Toc209286086)

[4.2 AsNMe_2_ 34](#_Toc209286087)

[4.3 TPh 38](#_Toc209286088)

[4.4 TPhOMe 42](#_Toc209286089)

[5. Absorption and emission in thin film 49](#_Toc209286090)

[6. Nanosecond Transient Absorption in solution 52](#_Toc209286091)

[7. Nanosecond Transient Absorption in thin film 56](#_Toc209286092)

[8. Triplet energies sensitizations 57](#_Toc209286093)

[9. Femtosecond Transient Absorption in solution 63](#_Toc209286094)

[10. Femtosecond Fluorescence Up conversion in solution 65](#_Toc209286095)

[11. Femtosecond Transient Absorption in thin film 67](#_Toc209286096)

[12. Triplet Yield Calculation 71](#_Toc209286097)

[13. Ultrafast Spectroscopy experiments summary 81](#_Toc209286098)

[14. TR-EPR Results 80](#_Toc209286099)

[14.1 TR-EPR in frozen solution 80](#_Toc209286100)

[14.2 TR-EPR in film 85](#_Toc209286101)

***15. Scanning Electron Microscopy of Thin Films………………………………………….91***

# **Experimental section:**

## **Synthesis, NMR and MS Characterization**

All reagents and solvents were purchased from Sigma-Aldrich (Merck Group, Italy) or Alfa Aesar (Johnson Matthey Group, United Kingdom) and used as received. All reactions were carried out under nitrogen atmosphere unless otherwise stated. Thin layer chromatography (TLC) was carried out on silica gel plates (Merck 60, F254); column chromatography was carried out on silica gel 60 (Merck, 0.063 – 0.200 mm).

NMR spectra were recorded using a Varian Inova 500 spectrometer and a Bruker Avance 400 spectrometer. Chemical shifts (*δ*) are expressed in ppm and referenced to residual undeuterated solvent. NMR data were processed using MestReC software (http://www.mestrec.com). Two-dimensional (2D) NMR experiments (gCOSY; gHSQCAD) were carried out on all new compounds using the pulse sequences from the Varian user library.

MS analyses were carried out using an API 2000™ LC/MS/MS (AB SCIEX, USA) triple quadrupole mass spectrometer equipped with TurboIonSpray® and APCI sources and a Finnigan LCQ Deca XP Max LC/MSⁿ system (Thermo Electron Corporation, USA), equipped with an electrospray ionization (ESI) source and a three- dimensional ion trap mass analyzer. The instruments operated in positive ion mode and were configured for full scan acquisition over an m/z range of 100–2000.

**Synthesis of Asymmetric pAQM derivatives**

General procedure for step1:

AcPDO (0.5 mmol) were mixed with the proper aldehyde (0.75 mmol), tetrabutylammonium bromide (TBA) (0.15 mmol) and potassium carbonate (K_2_CO_3_) (0.75 mmol) in acetonitrile (3 mL). The mixture was sonicated for 5 min and then stirred at room temperature for 24-48 h. After this period, the mixture was poured into water (40 mL). The solid formed was separated through filtration, rinsed with diethyl ether and, finally, dried in oven (50 °C, 4 h). The solid was employed for the next step without further purification.

**(3*Z*)-1-acetyl-3-[[4-(dimethylamino)phenyl]methylene]piperazine-2,5-dione**: 75 mg, orange solid, 52% yield. ^1^H NMR (500 MHz, DMSO-*d*_6_, 27°C, ppm) δ= 10.10 (s, 1H, N*H*), 7.49 (d, *J* = 8.7 Hz, 2H, C*H_Ar_*), 6.91 (s, 1H, =C*H*), 6.75 (d, *J* = 8.6 Hz, 2H, C*H_Ph_*), 4.35 (s, 2H, C*H_2_*), 2.98 (s, 6H, NC*H_3_*).

**(3*Z*)-1-acetyl-3-[(4-methoxyphenyl)methylene]piperazine-2,5-dione**: 98 mg, orange solid, 71% yield. ^1^H NMR (500 MHz, DMSO-*d*_6_, 27°C, ppm) δ= 10.18 (s, 1H, N*H*), 7.57 (d, *J* = 8.6 Hz, 2H, C*H_Ar_*), 7.00 (d, *J* = 8.6 Hz, 2H, C*H_Ph_*), 6.94 (s, 1H, =C*H*), 4.36 (s, 2H, C*H_2_*), 3.81 (s, 3H, OC*H_3_*).

General procedure for Step 2:

Monoaryl pAQM derivative (0.2 mmol) was mixed with the proper aldehyde (0.2 mmol), tetrabutylammonium bromide (TBA) (0.06 mmol) and potassium carbonate (K_2_CO_3_) (0.3 mmol) in acetonitrile (3 mL). The mixture was sonicated for 5 min and heated at 50 °C for 24-48 h. After cooling to room temperature, the mixture was poured into water (30 mL). The solid formed was separated through filtration, rinsed with diethyl ether and, finally, dried in oven (50 °C, 4 h). The solid was employed for the next step without further purification.

**(Z)-3-([2,2'-bithiophen]-5-ylmethylene)-6-((Z)-4-(dimethylamino)benzylidene)piperazine-2,5-dione**: 71.0 mg, dark orange solid, 86.6% yield. ^1^H NMR (500 MHz, DMSO-*d*_6_, 27°C, ppm) δ= 9.97 (s, 1H, N*H*), 9.66 (s, 1H, N*H*), 7.56 (d, *J* = 4.9 Hz, 1H, C*H_Th1_*), 7.47 (d, J=3.7 Hz, 1H, C*H_Th1_*), 7.45 (d, *J* = 8.7 Hz, 2H, C*H_Ph_*), 7.40 (d, *J* = 3.4 Hz, 1H, C*H_Th2_*), 7.36 (d, *J* = 3.8 Hz, 1H, C*H_Th2_*), 7.12 (dd, J_1_=4.9 Hz, J_2_=3.7Hz, 1H, C*H_Th1_*), 6.88 (s, 1H, =C*H*), 6.75 (d, J=6.8 Hz, 2H, C*H_ar_*), 6.74 (s, 1H, =C*H*), 2.97 (s, 6H, NC*H_3_*). The product, also at 70°C, was not soluble enough to yield a ^13^C spectrum with a suitably high signal-to-noise ratio after overnight acquisition.

**(Z)-3-([2,2'-bithiophen]-5-ylmethylene)-6-((Z)-4-methoxybenzylidene)piperazine-2,5-dione**: 70.7 mg, dark orange solid, 89% yield. ^1^H NMR (500 MHz, DMSO-*d*_6_, 27°C, ppm) δ= 10.01 (s, 1H, N*H*), 9.69 (s, 1H, N*H*), 7.55 (d, *J* = 4.9 Hz, 1H, C*H_Th1_*), 7.52 (d, J=8.1 Hz, 2H, CH*_Ph_*), 7.48 (d, *J* = 3.6 Hz, 1H, C*H_Th2_*), 7.40 (d, J=3.6 Hz, 1H, C*H_Th2_*), 7.35 (d, *J* = 3.8 Hz, 1H, C*H_Th1_*), 7.13 (dd, J_1_=4.9 Hz, J_2_=3.4 Hz, 1H, C*H_Th1_*), 7.00 (d, *J* = 8.1 Hz, 2H, C*H_Ph_*), 6.91 (s, 1H, =C*H*), 6.78 (s, 1H, =C*H*), 3.81 (s, 3H, OC*H_3_*). The product, also at 70°C, was not soluble enough to yield a ^13^C spectrum with a suitably high signal-to-noise ratio after overnight acquisition.

General procedure for Step 3:

Asymmetric derivative (0.1 mmol) was mixed with tert-Butyl bromoacetate (0,4 mmol, 4 eq.), 13.8 mg of tetrabutylammonium bromide (TBA) (0.04 mmol, 0.4 eq.), and 69 mg of potassium carbonate (K_2_CO_3_) (0.5 mmol, 5 eq.) in 2 mL of acetonitrile. The mixture was sonicated for 10 min and stirred at room temperature overnight. The mixture was poured into water (30 mL). The solid formed was separated through filtration, rinsed with diethyl ether and, finally, dried in oven (50 °C, 4 h).

**AsNMe_2_: *tert*-butyl 2-[(3*Z*,6*Z*)-5-(2-*tert*-butoxy-2-oxo-ethoxy)-3-[[4-(dimethylamino)phenyl]-methylene]-6-[[5-(2-thienyl)-2-thienyl]methylene]pyrazin-2-yl]oxyacetate**: 53 mg, light orange solid, 82.2% yield.

^1^H NMR (400 MHz, Acetone-*d*_6_, 25°C, ppm) δ= 8.04 (d, *J* = 9.0 Hz, 2H, C*H_Ph_*), 7.47 (dd, J_1_ = 5.1 Hz, J_2_ = 1.0 Hz, 1H, C*H_Th1_*), 7.42 (dd, J_1_ = 3.6 Hz, J_2_ = 1.0 Hz, 1H, C*H_Th1_*), 7.33 (d, *J* = 3.9 Hz, 1H, C*H_Th2_*), 7.26 (d, *J* = 3.9 Hz, 1H, C*H_Th2_*), 7.15 (s, 1H, =C*H*), 7.13 (dd, J_1_=5.1 Hz, J_2_=3.6 Hz, 1H, C*H_Th1_*), 6.78 (d, J=9.0 Hz, 2H, C*H_ar_*), 7.00 (s, 1H, =C*H*), 5.16 (s, 2H, OC*H_2_*), 4.92 (s, 2H, OC*H_2_*), 3.06 (s, 6H, N(C*H_3_*)_2_). 1.44 (s, 9H, C(C*H_3_*)_3_), 1.39 (s, 9H, C(C*H_3_*)_3_). ^13^C NMR (100 MHz, Acetone-*d*_6_, 25°C, ppm) δ= 192.44, 192.32, 166.85, 160.31, 157.49, 142.67, 141.90, 137.57, 137.51, 133.24, 132.27, 128.14, 125.38, 124.14, 123.70, 123.29, 116.68, 113.91, 113.21, 110.27, 81.46, 81.39, 63.67, 63.26, 54.79, 27.36, 27.31. MS (ESI) m/z (%): 650.33 (95) [M+H]^+^, 672.33 (100) [M+Na]^+^.

**AsOMe: *tert*-butyl 2-[(3*Z*,6*Z*)-5-(2-*tert*-butoxy-2-oxo-ethoxy)-3-[(4-methoxyphenyl)methylene]-6-[[5-(2-thienyl)-2-thienyl]methylene]pyrazin-2-yl]oxyacetate**: 46.2 mg, orange solid, 72.5% yield.

^1^H NMR (500 MHz, Acetone-*d*_6_, 25°C, ppm) δ= 8.12 (d, *J* = 8.9 Hz, 2H, C*H_Ph_*), 7.49 (dd, J_1_ = 5.1 Hz, J_2_ = 1.0 Hz, 1H, C*H_Th1_*), 7.44 (dd, J_1_ = 3.6 Hz, J_2_ = 1.0 Hz, 1H, C*H_Th1_*), 7.38 (d, *J* = 3.9 Hz, 1H, C*H_Th2_*), 7.28 (d, *J* = 3.9 Hz, 1H, C*H_Th2_*), 7.24 (s, 1H, =C*H*), 7.14 (dd, J_1_=5.1 Hz, J_2_=3.6 Hz, 1H, C*H_Th1_*), 7.02 (s, 1H, =C*H*), 6.99 (d, J=8.9 Hz, 2H, C*H_ar_*), 5.17 (s, 2H, OC*H_2_*), 4.94 (s, 2H, OC*H_2_*), 3.87 (s, 6H, OC*H_3_*). 1.42 (s, 9H, C(C*H_3_*)_3_), 1.39 (s, 9H, C(C*H_3_*)_3_). ^13^C NMR (100 MHz, Acetone-*d*_6_, 25°C, ppm) δ= 167.12, 166.99, 157.82, 155.20, 153.97, 150.82, 141.04, 137.99, 133.37, 131.34, 128.09, 127.28, 125.98, 125.33, 125.11, 123.89, 123.70, 123.20, 114.94, 111.69, 81.34, 81.30, 63.54, 63.13, 39.26, 27.37, 27.35. MS (ESI) m/z (%): 637.33 (55) [M+H]^+^, 659.40 (100) [M+Na]^+^, 1321.07 (30) [2M+Na]^+^

**General procedure for Suzuki reaction**

A mixture of Pd(OAc)_2_ (4 mg, 2 mol%), PEG 2000 (3.5 g), Na_2_CO_3_ (0.212 g, 2 mmol) and water (3 mL) was heated to 50 °C with stirring. Thus, 5-Bromo-2-thiophenecarboxaldehyde (1 mmol) and the proper phenylboronic acid (1.5 mmol) were added and the reaction carried out at 50 °C for 45-60 min. The reaction mixture was cooled to room temperature and extracted four times with diethyl ether (4×15 ml). The combined organic phases were rotavaporated under vacuum. The desired product was purified by column chromatography on a silica gel column (eluent cyclohexane\ethylacetate).

**5-Phenylthiophene-2-carboxaldehyde**: Using phenylboronic acid, 157 mg, white solid, 83% yield.^[1]^

^1^H NMR (500 MHz, Cholroform-*d*) δ ppm 9.90 (s, 1H, CO*H*), 7.75 (d, *J* = 3.9 Hz, 1H, C*H_Th_*), 7.68 (dd, J_1_ = 8.1 Hz, J_2_ = 1.4 Hz, 2H, C*H_Ph_*), 7.52 (t, J = 8.1 Hz, 1H, C*H_Ph_*), 7.43 (m, 2H, C*H_Ph_*), 7.41 (d, J = 3.9 Hz, 1H, C*H_Th_*).

**5-(4-methoxyphenyl)thiophene-2-carboxaldehyde**: Using 4-Methoxyphenylboronic acid, 193 mg, pastel yellow solid, 88% yield.^[1]^

^1^H NMR (500 MHz, Cholroform-*d*) δ ppm 9.88 (s, 1H, CO*H*), 7.98 (d, *J =* 3.93 Hz, 1H, C*H_Th_*), 7.74 (dd, J_2_ *=* 8.8 Hz, J_2_ *=* 1.8 Hz, 2H. C*H_Ph_*), 7.60 (d, *J =* 3.93 Hz, 1H, C*H_Th_*), 7.04 (dd, J_2_ *=* 8.8 Hz, J_2_ *=* 1.8 Hz, 2H. C*H_Ph_*), 3.83 (s, 3H, OC*H_3_*).

**Synthesis of Asymmetric pAQM derivatives**

General procedure for step1:

AcPDO (58 mg, 0.3 mmol) was mixed with the proper aldehyde (0.69 mmol), tetrabutylammonium bromide (TBA) (38 mg, 0.12 mmol) and potassium carbonate (K_2_CO_3_) (160 mg, 1.2 mmol) in acetonitrile (2 mL). The mixture was sonicated for 5 min and heated at 60 °C for 12-18 h. After cooling to room temperature, the mixture was poured into water (25 mL). The solid formed was separated through filtration, rinsed with diethyl ether and, finally, dried in oven (50 °C, 4 h). The solid was employed for the next step without further purification.

**(3*Z*,6*Z*)-3,6-bis[(5-phenyl-2-thienyl)methylene]piperazine-2,5-dione**: Using 5-phenylthiophene-2-carboxyaldehyde, 126 mg, orange solid, 92.3% yield.

^1^H NMR (500 MHz, DMSO-*d*_6_, 27°C, ppm) δ= 10.78 (s, 2H, N*H*), 7.70 (d, *J =* 7.6 Hz, 4H. C*H_Ph_*), 7.55 (d, *J =* 3.2 Hz, 2H), 7.44 (m, 4H, C*H_Ph_*), 7.34 (m, 2H, C*H_Ph_*), 6.86 (s, 2H, =C*H*). The product, also at 70°C, was not soluble enough to yield a ^13^C spectrum with a suitably high signal-to-noise ratio after overnight acquisition.

**(3*Z*,6*Z*)-3,6-bis[[5-(4-methoxyphenyl)-2-thienyl]methylene]piperazine-2,5-dione**: Using 5-(4methoxyphenyl)thiophene-2-carboxyaldehyde, 133 mg, orange solid, 86.2 % yield.

^1^H NMR (500 MHz, DMSO-*d*_6_, 27°C, ppm) δ 9.88 (s, 2H, N*H*), 7.64 (d, *J =* 8.4 Hz, 4H C*H_Ph_*), 7.53 (d, *J =* 3.6 Hz, 2H, C*H_Th_*), 7.46 (d, *J =* 3.6 Hz, 2H, C*H_Th_*), 7.17 (s, 2H, =C*H*), 7.02 (d, *J =* 8.4 Hz, 4H, C*H_Ph_*), 3.81 (s, 6H, OC*H_3_*. The product, also at 70°C, was not soluble enough to yield a ^13^C spectrum with a suitably high signal-to-noise ratio after overnight acquisition.

General procedure for step 2

0,1 mmol of pAQM-Ar were mixed with tert-Butyl bromoacetate (58µL, 0.4 mmol), tetrabutylammonium bromide (TBA) (12.9 mg, 0.04 mmol), and potassium carbonate (K_2_CO_3_) (55 mg, 0.4 mmol) in 2 mL of acetonitrile. The mixture was sonicated for 10 min and heated at 50 °C for 12-18 h. After cooling to room temperature, the mixture was poured into water (50 mL). The solid formed was separated through filtration, rinsed with diethyl ether and, finally, dried in oven (50 °C, 4 h).

**TPh *tert*-butyl 2-[(3*Z*,6*Z*)-5-(2-*tert*-butoxy-2-oxo-ethoxy)-3,6-bis[(5-phenyl-2-thienyl)methylene]-pyrazin-2-yl]oxyacetate**: 52 mg, purple sangria solid, 76.2 % yield.

^1^H NMR (500 MHz, DMSO-*d*_6_, 27°C, ppm) δ= 7.70 (d, *J =* 7.4 Hz, 2H, C*H_Ph_*), 7.56 (d, *J =* 3.8 Hz, 2H, C*H_Th_*), 7.52 (d, *J =* 3.8 Hz, 2H, C*H_Th_*), 7.45 (t, *J =* 7.6 Hz, 4H, C*H_Ph_*), 7.35 (t, *J =* 7.4 Hz, 2H, C*H_Ph_*), 7.52 (dd, J=3.9 Hz, 2H, C*H_Th_*), 7.30 (s, 2H, =C*H*), 5.18 (s, 4H, OC*H_2_*), 1.33 (s, 18H, C(*CH_3_)_3_*); (400 MHz, Chloroform-*d*, 25°C, ppm) δ 10.78 (s, 2H, N*H*), 7.70 (d, *J =* 7.6 Hz, 2H. C*H_Ph_*), 7.55 (d, *J =* 3.2 Hz, 1H), 7.44 (m, 2H, C*H_Ph_*), 7.34 (m, 1H, C*H_Ph_*), 6.86 (s, 1H, =C*H*). ^13^C NMR (100 MHz, Chloroform-*d*, 25°C, ppm) δ=167.38, 156.62, 148.85, 138.46, 134.62, 132.27, 128.90, 127.75, 127.18, 125.69, 122.84, 117.98, 82.33, 63.84, 28.11.

MS (APCI) m/z (%): 570.2 (100) [M-2tBu+2H]^+^, 627.0 (45) [M-tBu+2H]^+^, 682.1 (40) [M^⦁^]^+^.

**TPhOMe *tert*-butyl 2-[(3*Z*,6*Z*)-5-(2-*tert*-butoxy-2-oxo-ethoxy)-3,6-bis[[5-(4-methoxyphenyl)-2-thienyl]methylene]pyrazin-2-yl]oxyacetate**: 65 mg, purple wine solid, 87.5% yield.

^1^H NMR (500 MHz, DMSO-*d*_6_, 27°C, ppm) δ= 7.71 (d, *J =* 8.7 Hz, 2H, C*H_Ph_*), 7.47 (d, *J =* 3.9 Hz, 2H, C*H_Th_*), 7.42 (d, *J =* 3.9 Hz, 2H, C*H_Th_*), 7.26 (s, 2H, =CH-), 7.01 (d, *J =* 8.7 Hz, 2H, C*H_Ph_*), 7.01 (d, *J =* 8.8 Hz, 2H, PhH), 5.16 (s, 2H, OC*H_2_*), 3.82 (s. 6H, OC*H_3_*) 1.33 (s, 18H, C(*CH_3_)_3_*); (400 MHz, Chloroform-*d*, 25°C, ppm) δ 10.78 (s, 2H, N*H*), 7.70 (d, *J =* 7.6 Hz, 2H. C*H_Ph_*), 7.55 (d, *J =* 3.2 Hz, 1H), 7.44 (m, 2H, C*H_Ph_*), 7.34 (m, 1H, C*H_Ph_*), 6.86 (s, 1H, =C*H*). ^13^C NMR (100 MHz, Chloroform-*d*, 25°C, ppm) δ= 167.53, 159.48, 156.46, 148.86, 137.54, 132.31, 127.53, 126.96, 126.79, 121.79, 117.87, 114.37, 82.34, 63.79, 55.40, 28.13.

MS (APCI) m/z (%): 629.8 (15) [M-2tBu+2H]^+^, 685.9 (15) [M-tBu+H]^+^, 741.7 (45) [M^⦁^]^+^.


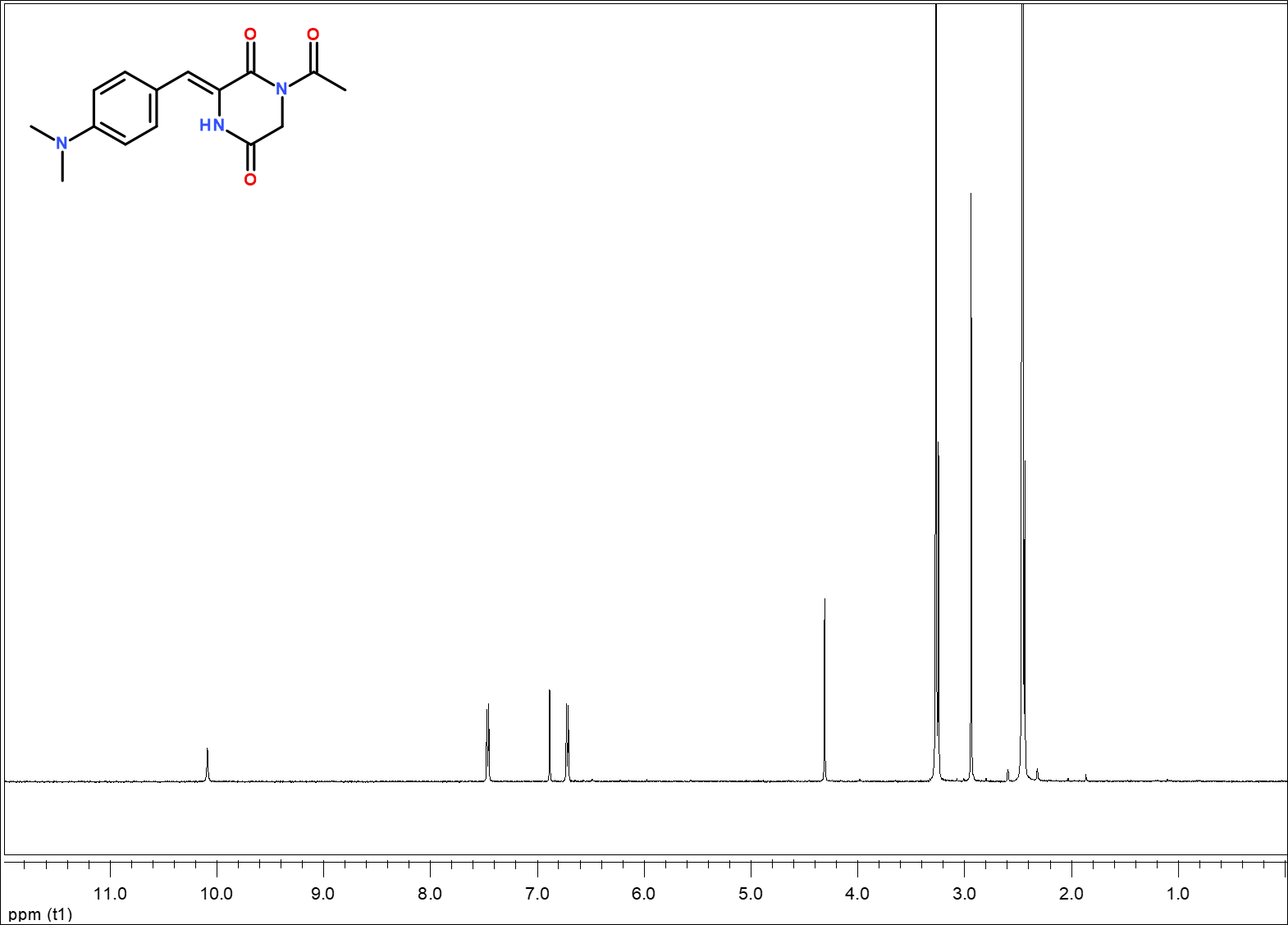


**Figure S1** ^1^H NMR spectrum of (3Z)-1-acetyl-3-[[4-(dimethylamino)phenyl]methylene]piperazine-2,5-dione (500 MHz, 27°C, DMSO *d_6_*)


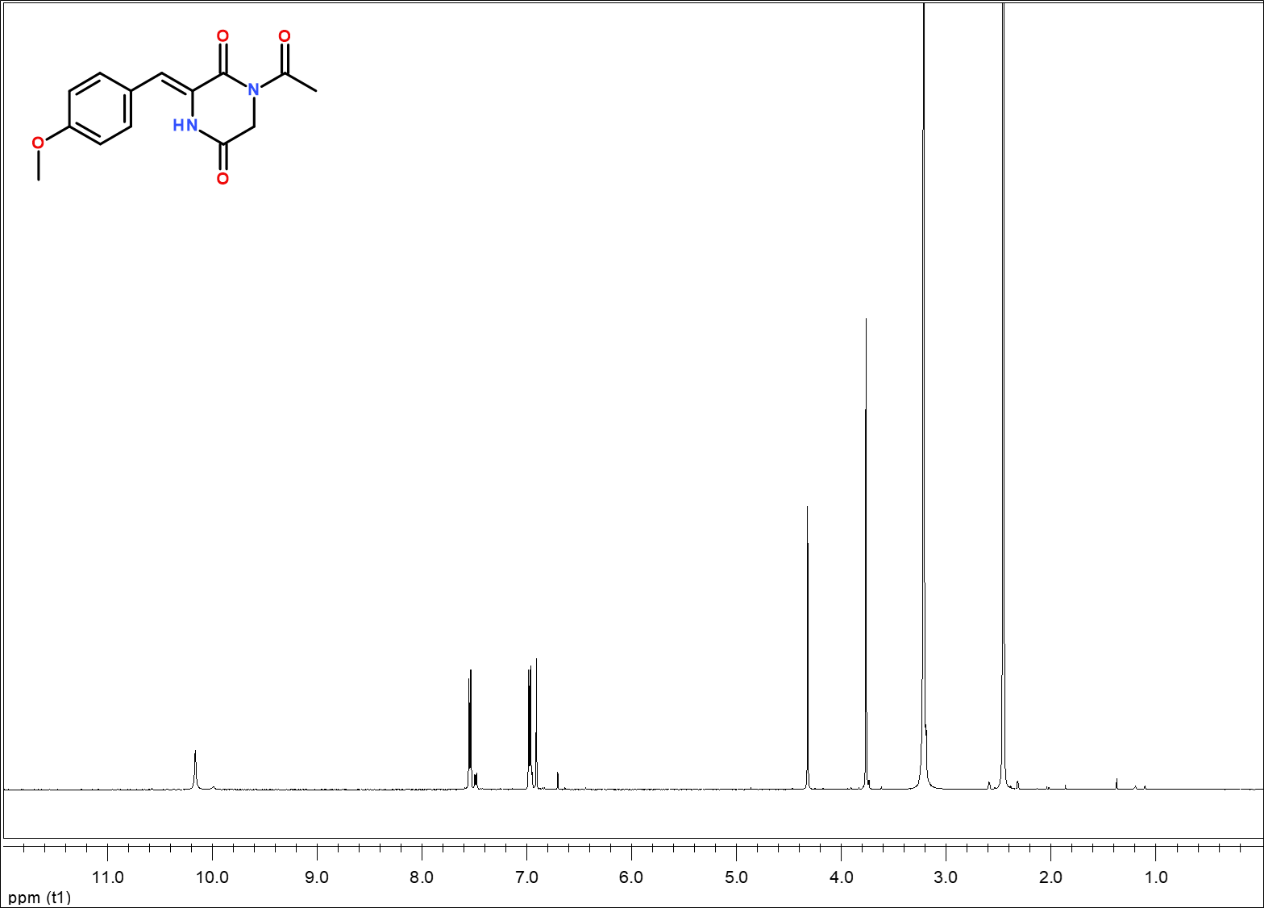


**Figure S2** ^1^H NMR spectrum of (3Z)-1-acetyl-3-[[4-methoxyphenyl]methylene]piperazine-2,5-dione (500 MHz, 27°C, DMSO *d_6_*)


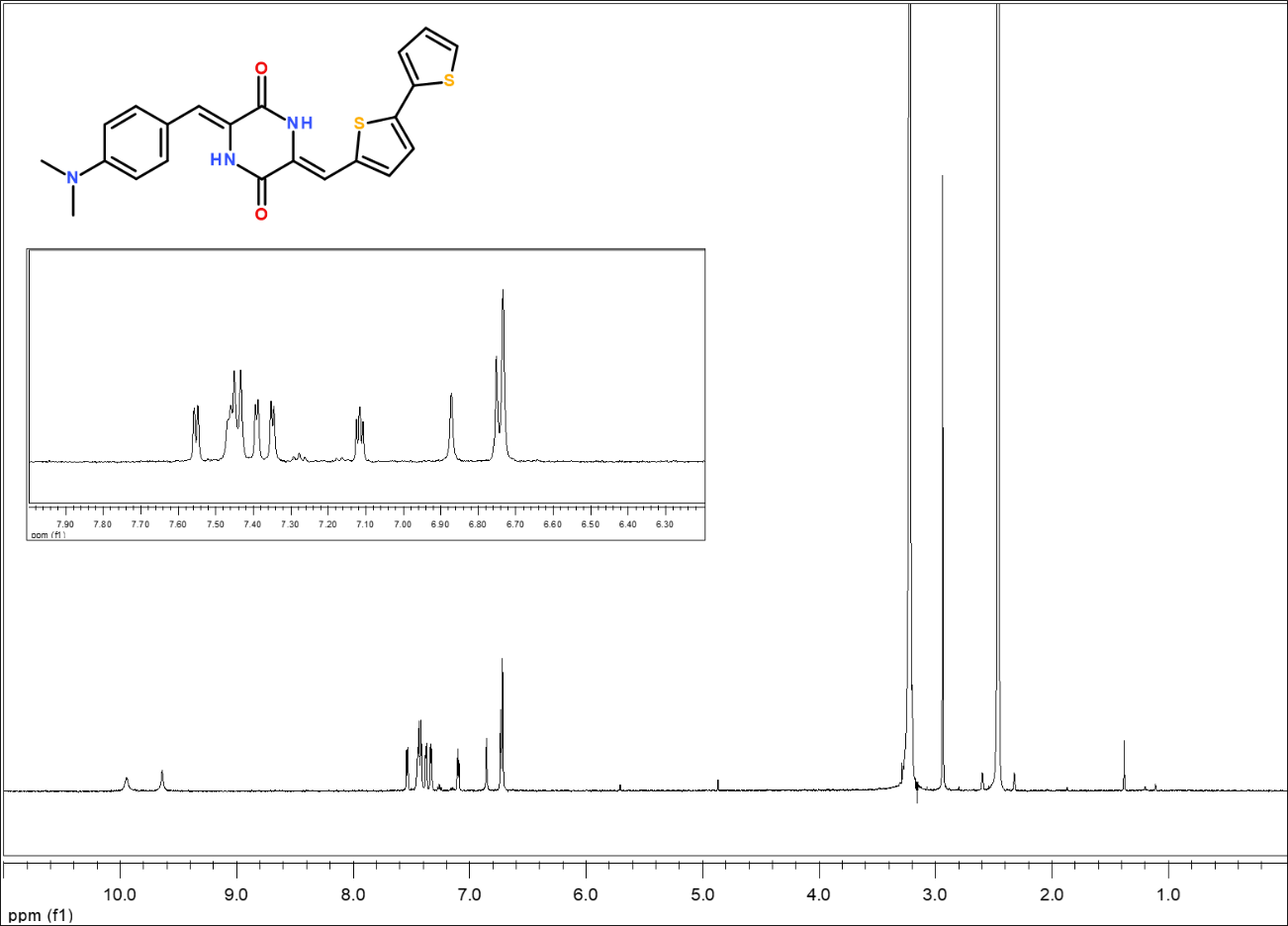


**Figure S3** ^1^H NMR spectrum of ((Z)-3-([2,2'-bithiophen]-5-ylmethylene)-6-((Z)-4-(dimethylamino)-benzylidene)piperazine-2,5-dione (500 MHz, 27°C, DMSO *d_6_*)


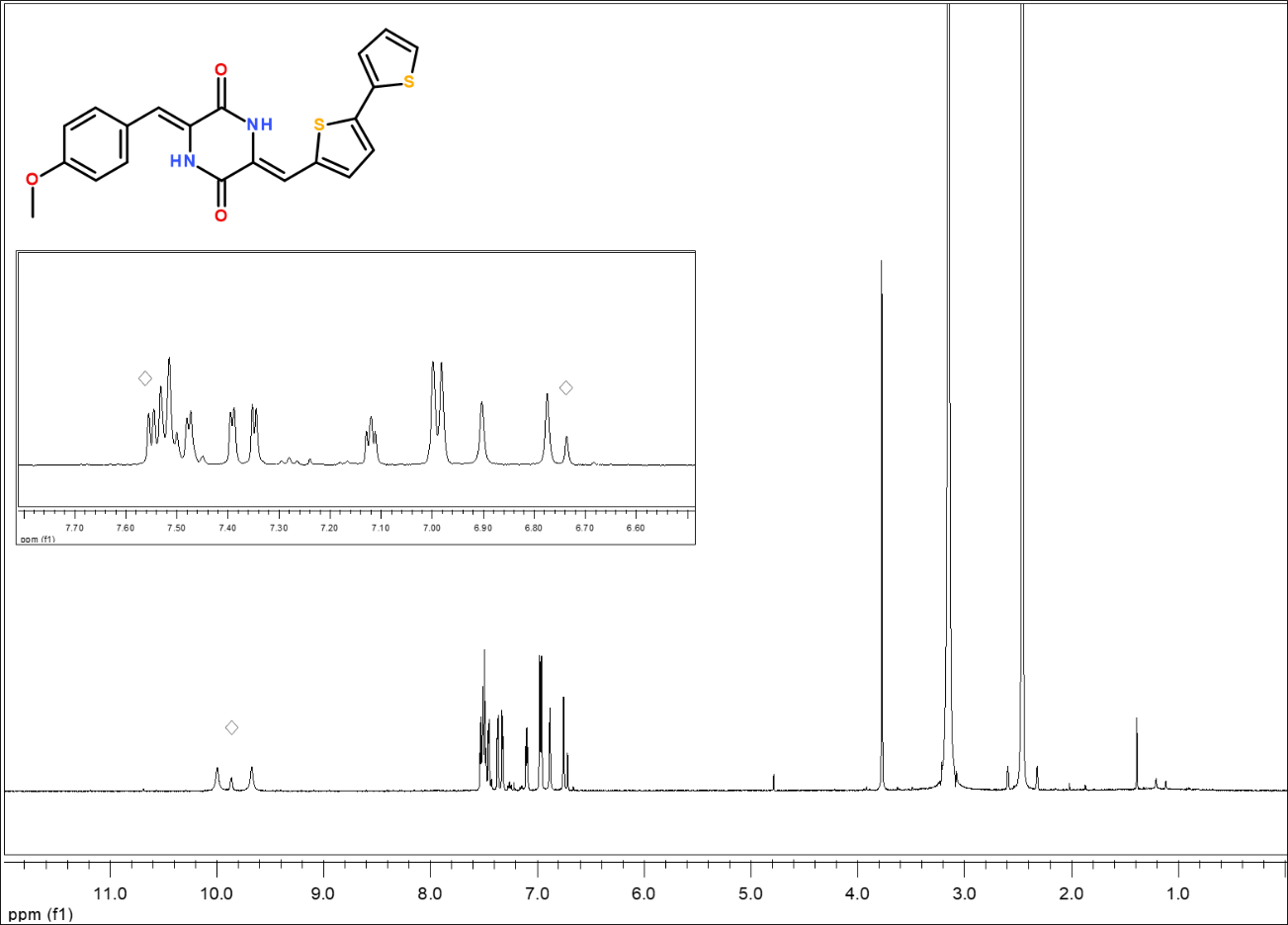


**Figure S4** ^1^H NMR spectrum of ((Z)-3-([2,2'-bithiophen]-5-ylmethylene)-6-((Z)-4-methoxy
benzylidene)piperazine-2,5-dione (500 MHz, 27°C, DMSO *d_6_*), ◊ Signals from residual impurities.


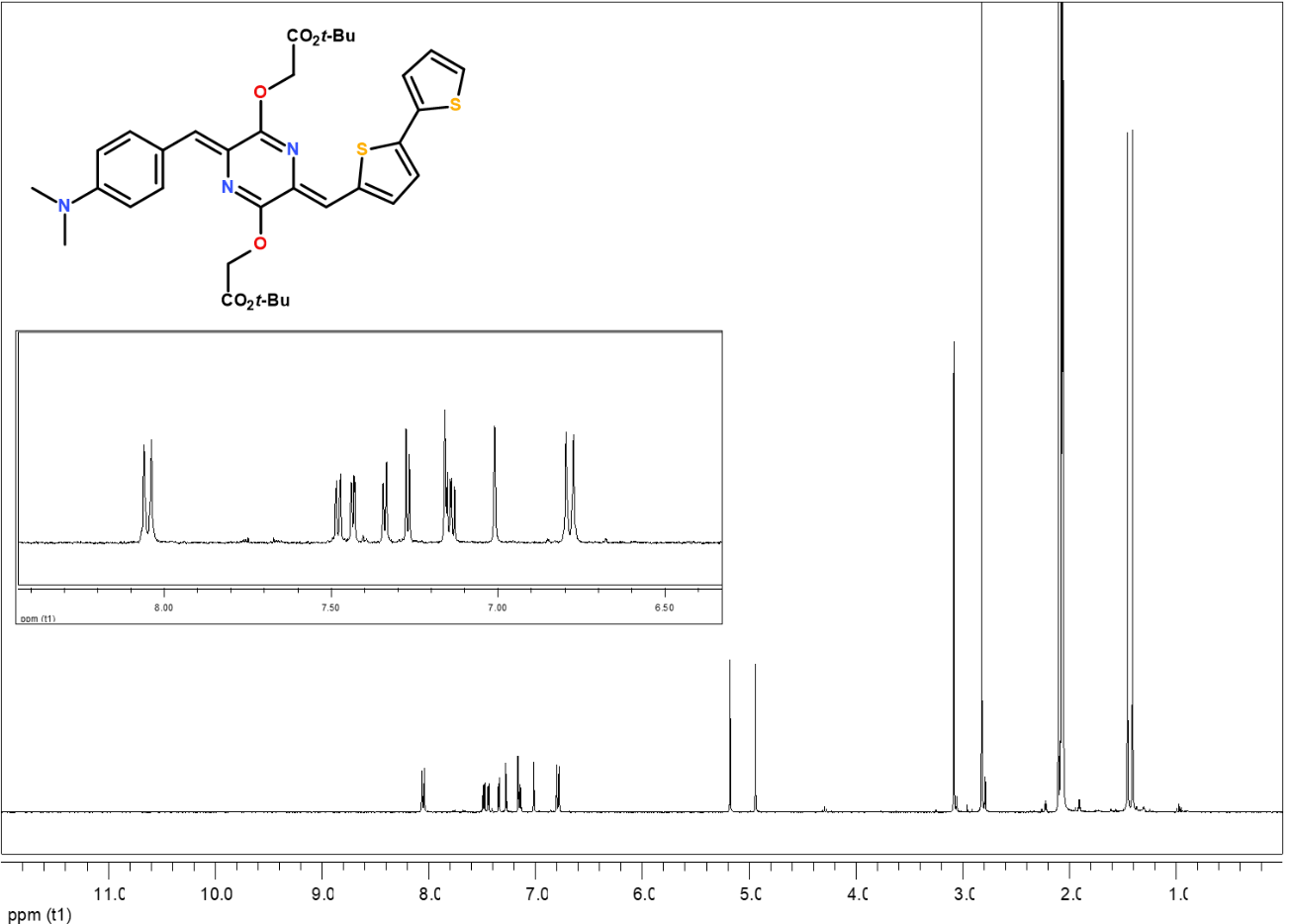


**Figure S5** ^1^H NMR spectrum of **AsNMe_2_**: tert-butyl 2-[(3Z,6Z)-5-(2-tert-butoxy-2-oxo-ethoxy)-3-[[4-(dimethylamino)phenyl]-methylene]-6-[[5-(2-thienyl)-2-thienyl]methylene]pyrazin-2-yl]oxyacetate (400 MHz, 25°C, Acetone *d_6_*)


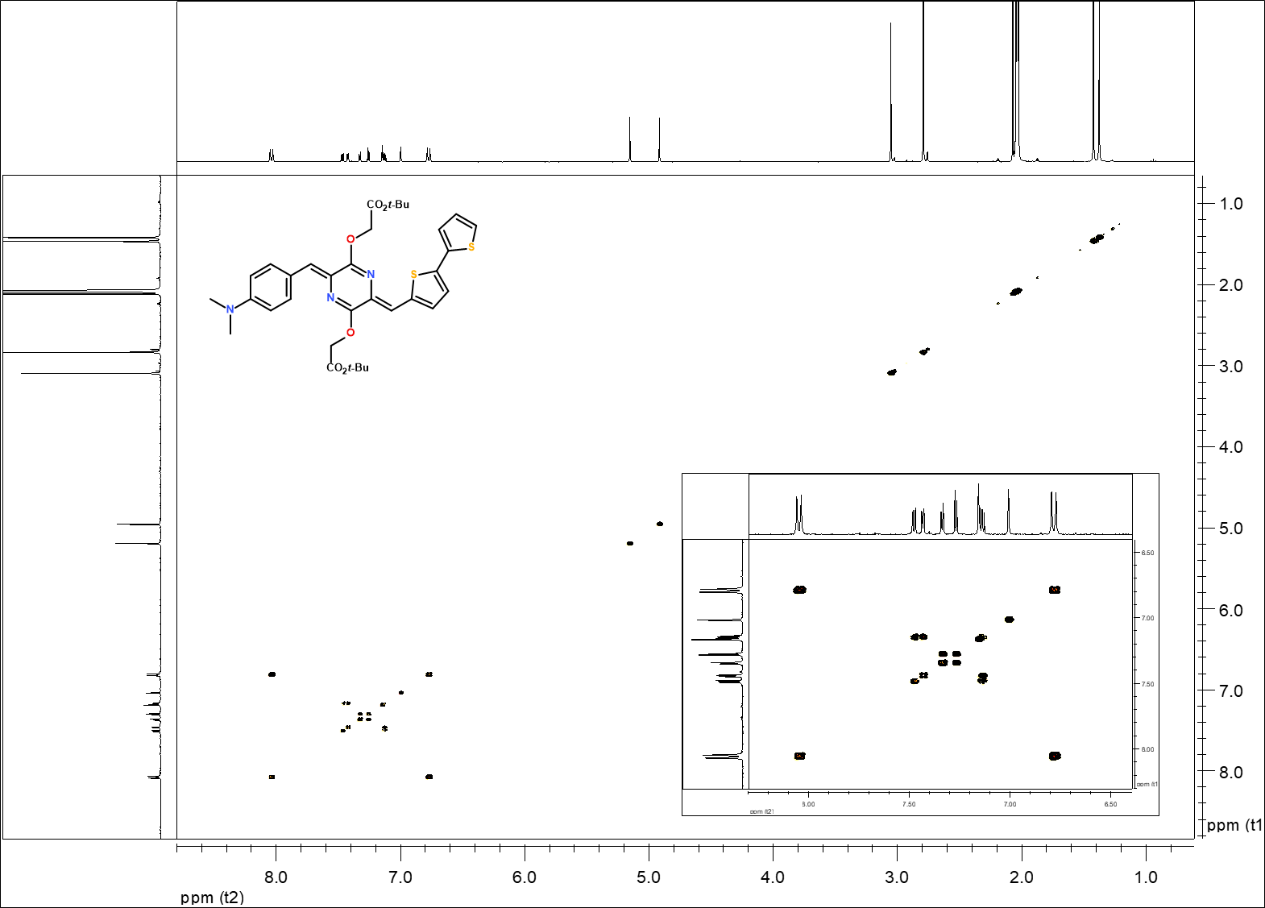


**Figure S6** ^1^H-^1^H gCOSY NMR spectrum of **AsNMe_2_**: tert-butyl 2-[(3Z,6Z)-5-(2-tert-butoxy-2-oxo-ethoxy)-3-[[4-(dimethylamino)phenyl]-methylene]-6-[[5-(2-thienyl)-2-thienyl]methylene]pyrazin-2-yl]oxyacetate (400 MHz, 25°C, Acetone *d_6_*)


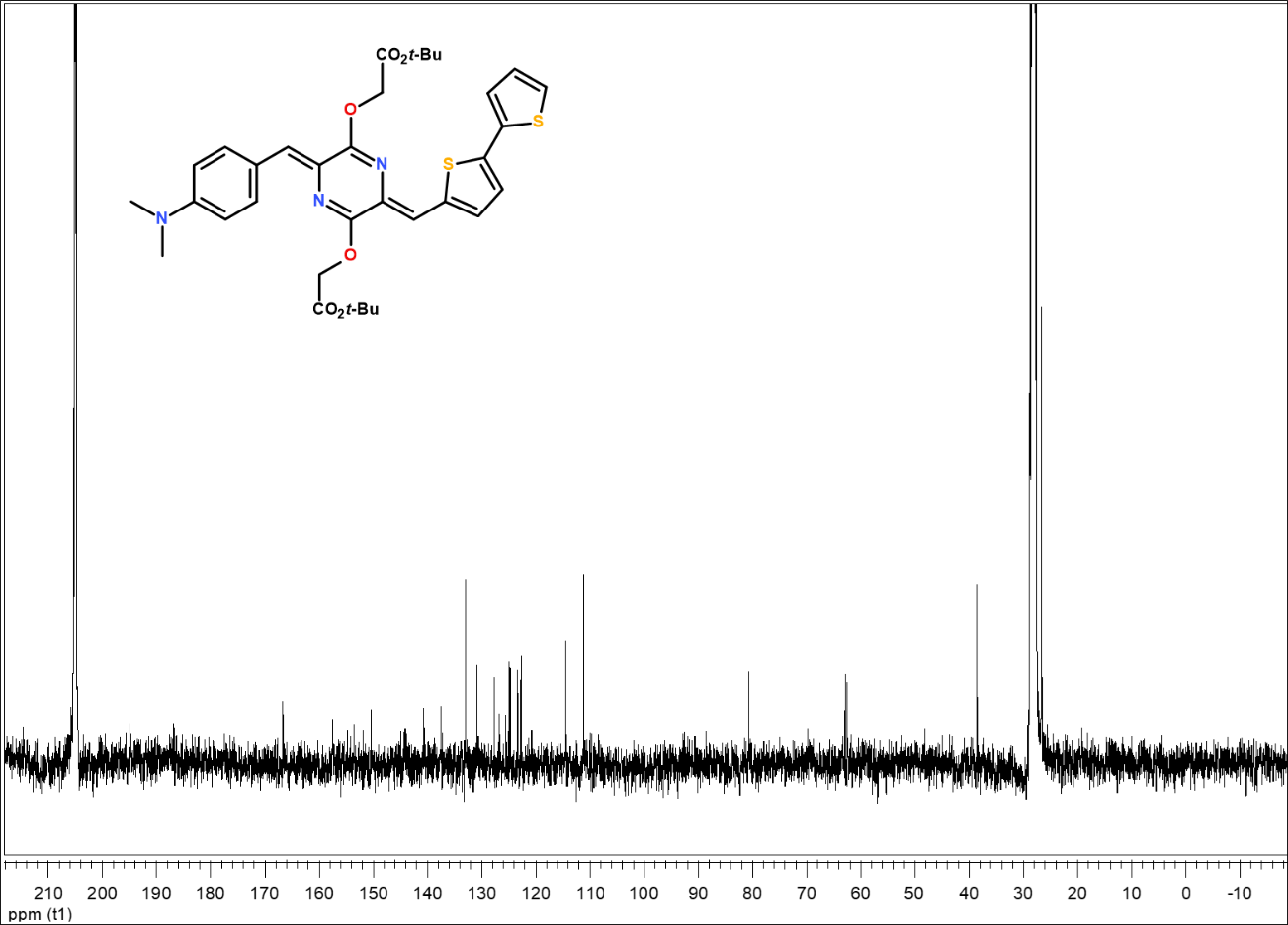


**Figure S7** ^13^C NMR spectrum of **AsNMe_2_**: tert-butyl 2-[(3Z,6Z)-5-(2-tert-butoxy-2-oxo-ethoxy)-3-[[4-(dimethylamino)phenyl]-methylene]-6-[[5-(2-thienyl)-2-thienyl]methylene]pyrazin-2-yl]oxyacetate (400 MHz, 25°C, Acetone *d_6_*)


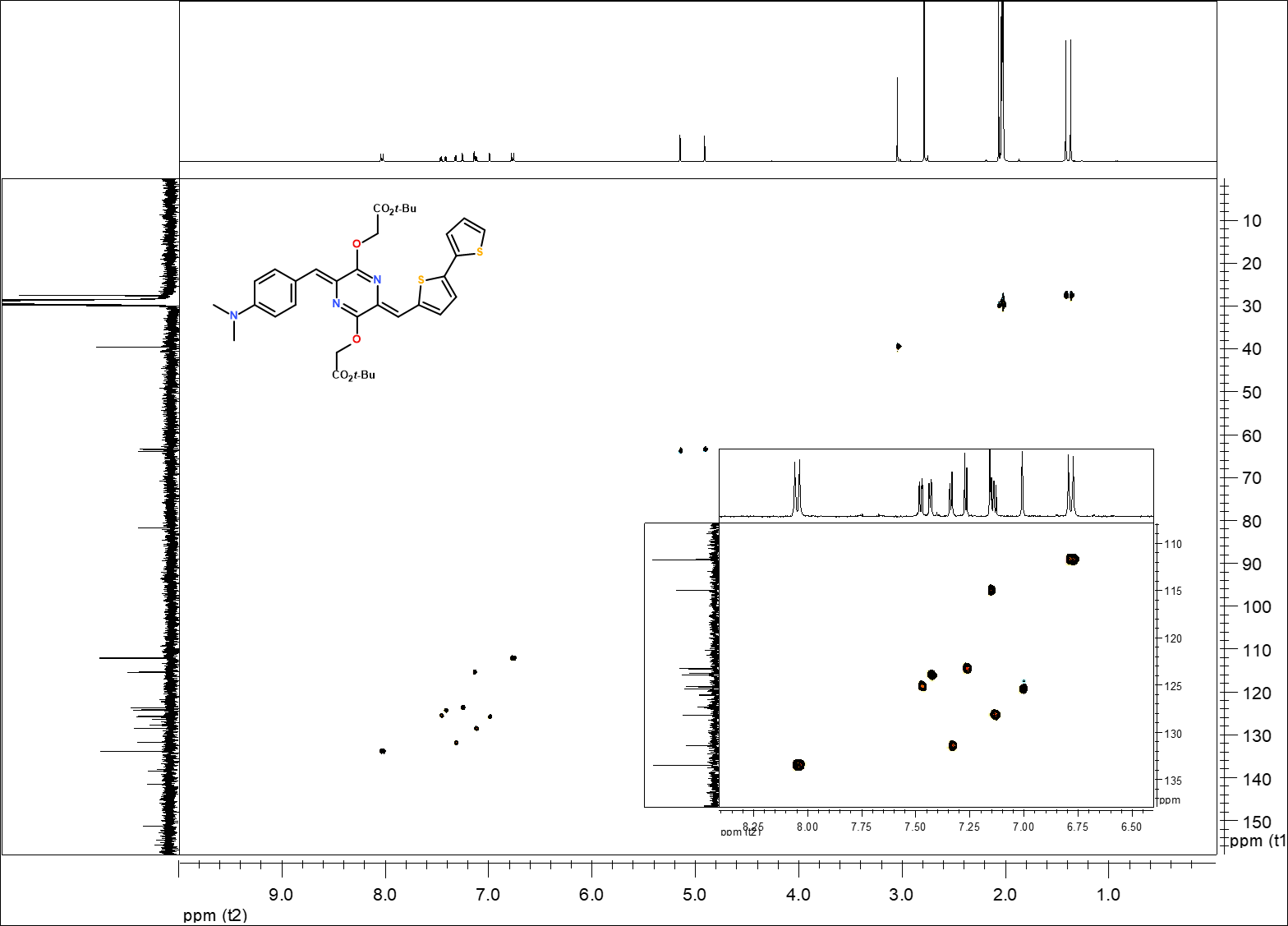


**Figure S8** ^1^H-^13^C gHSQCAD NMR spectrum of **AsNMe_2_**: tert-butyl 2-[(3Z,6Z)-5-(2-tert-butoxy-2-oxo-ethoxy)-3-[[4-(dimethylamino)phenyl]-methylene]-6-[[5-(2-thienyl)-2-thienyl]methylene]-pyrazin-2-yl]oxyacetate (400 MHz, 25°C, Acetone *d_6_*)


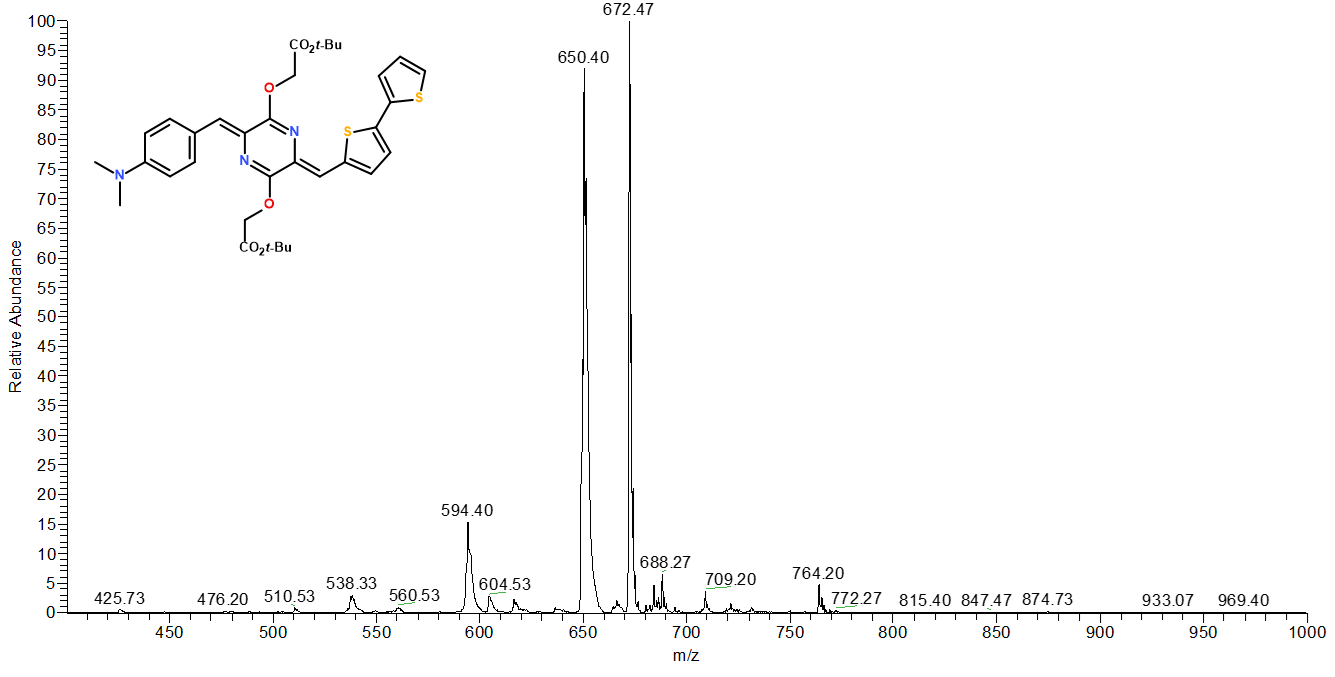


**Figure S9** MS (ESI) spectrum of **AsNMe_2_**: tert-butyl 2-[(3Z,6Z)-5-(2-tert-butoxy-2-oxo-ethoxy)-3-[[4-(dimethylamino)phenyl]-methylene]-6-[[5-(2-thienyl)-2-thienyl]methylene]pyrazin-2-yl]oxyacetate


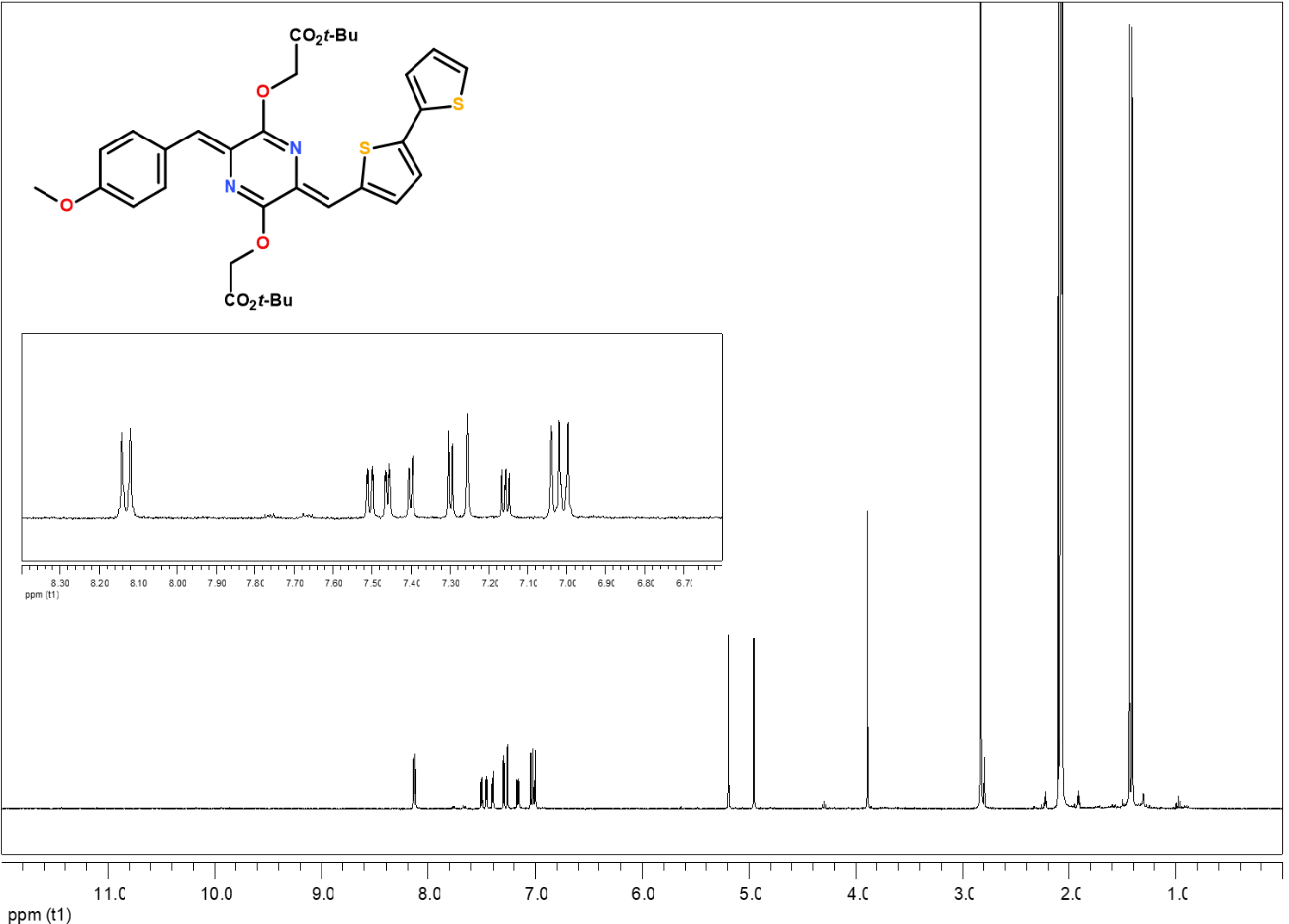


**Figure S10** ^1^H NMR spectrum of **AsOMe**: tert-butyl 2-[(3Z,6Z)-5-(2-tert-butoxy-2-oxo-ethoxy)-3-[[4-methoxyphenyl]-methylene]-6-[[5-(2-thienyl)-2-thienyl]methylene]pyrazin-2-yl]oxyacetate (400 MHz, 25°C, Acetone *d_6_*)


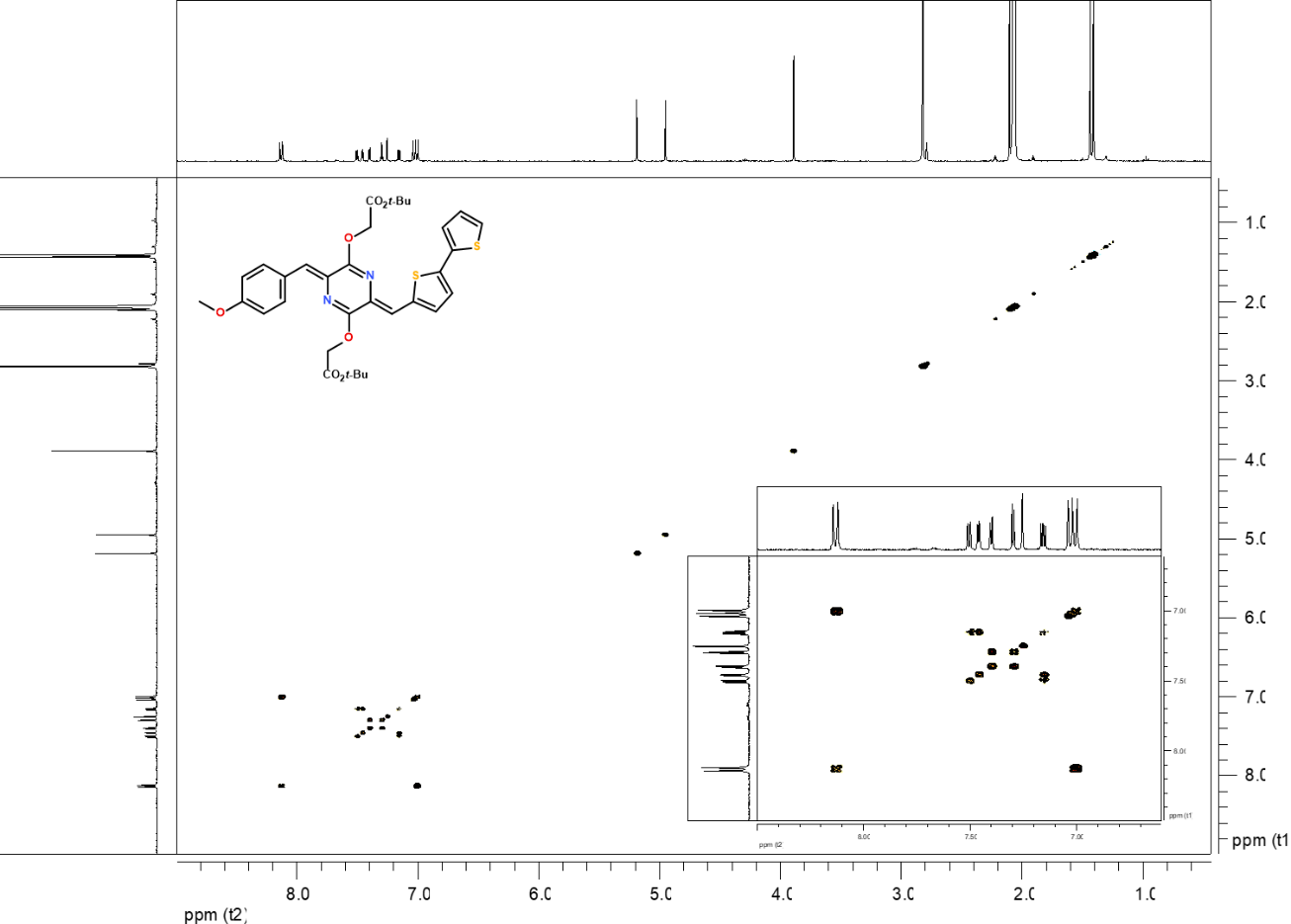


**Figure S11** ^1^H-^1^H gCOSY NMR spectrum of **AsOMe**: tert-butyl 2-[(3Z,6Z)-5-(2-tert-butoxy-2-oxo-ethoxy)-3-[[4-methoxyphenyl]-methylene]-6-[[5-(2-thienyl)-2-thienyl]methylene]pyrazin-2-yl]oxyacetate (400 MHz, 25°C, Acetone *d_6_*)


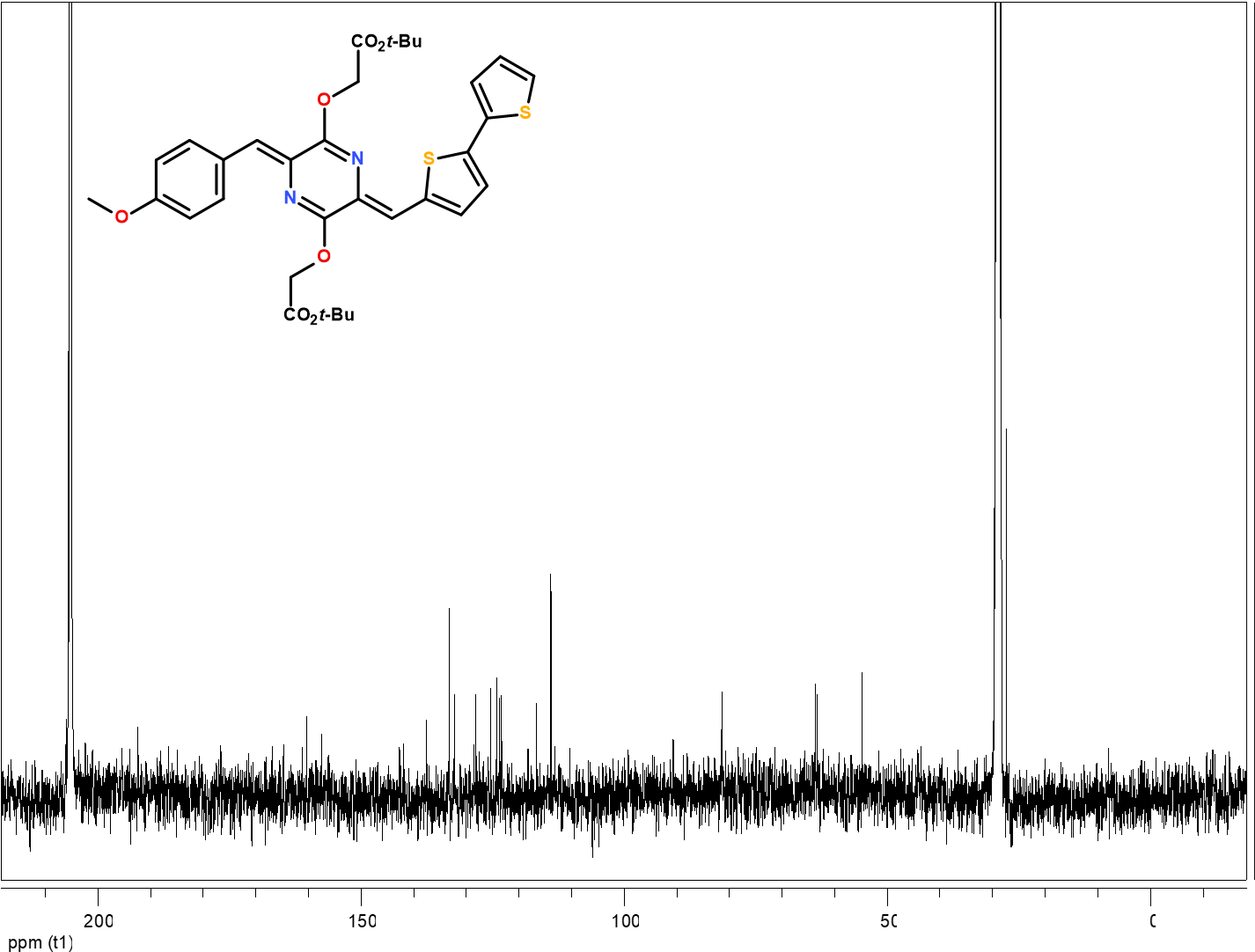


**Figure S12** ^13^C NMR spectrum of **AsOMe**: tert-butyl 2-[(3Z,6Z)-5-(2-tert-butoxy-2-oxo-ethoxy)-3-[[4-methoxyphenyl]-methylene]-6-[[5-(2-thienyl)-2-thienyl]methylene]pyrazin-2-yl]oxyacetate (400 MHz, 25°C, Acetone *d_6_*)


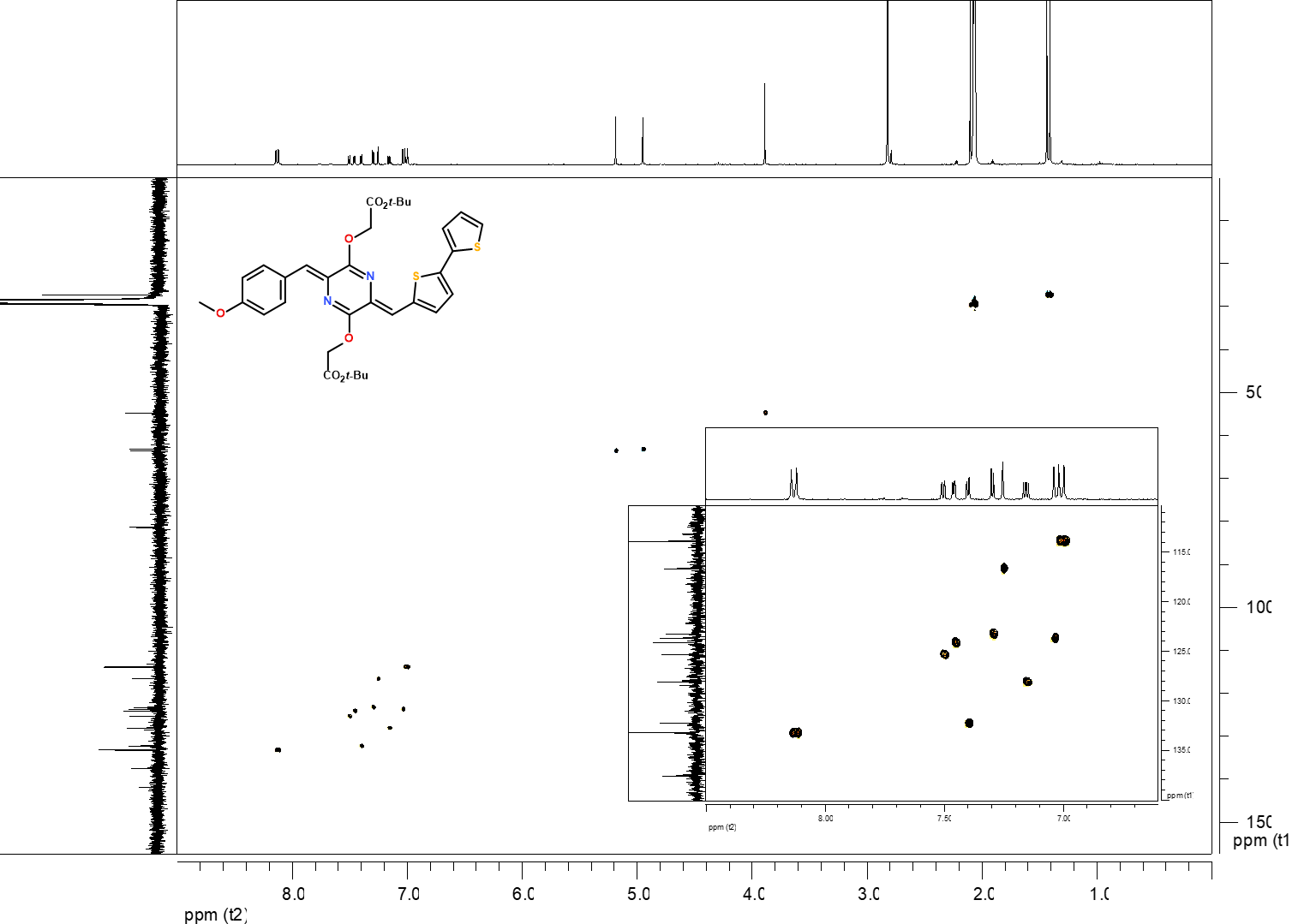


**Figure S13** ^1^H-^13^C gHSQCAD NMR spectrum of **AsOMe**: tert-butyl 2-[(3Z,6Z)-5-(2-tert-butoxy-2-oxo-ethoxy)-3-[[4-methoxyphenyl]-methylene]-6-[[5-(2-thienyl)-2-thienyl]methylene]pyrazin-2-yl]oxyacetate (400 MHz, 25°C, Acetone *d_6_*)


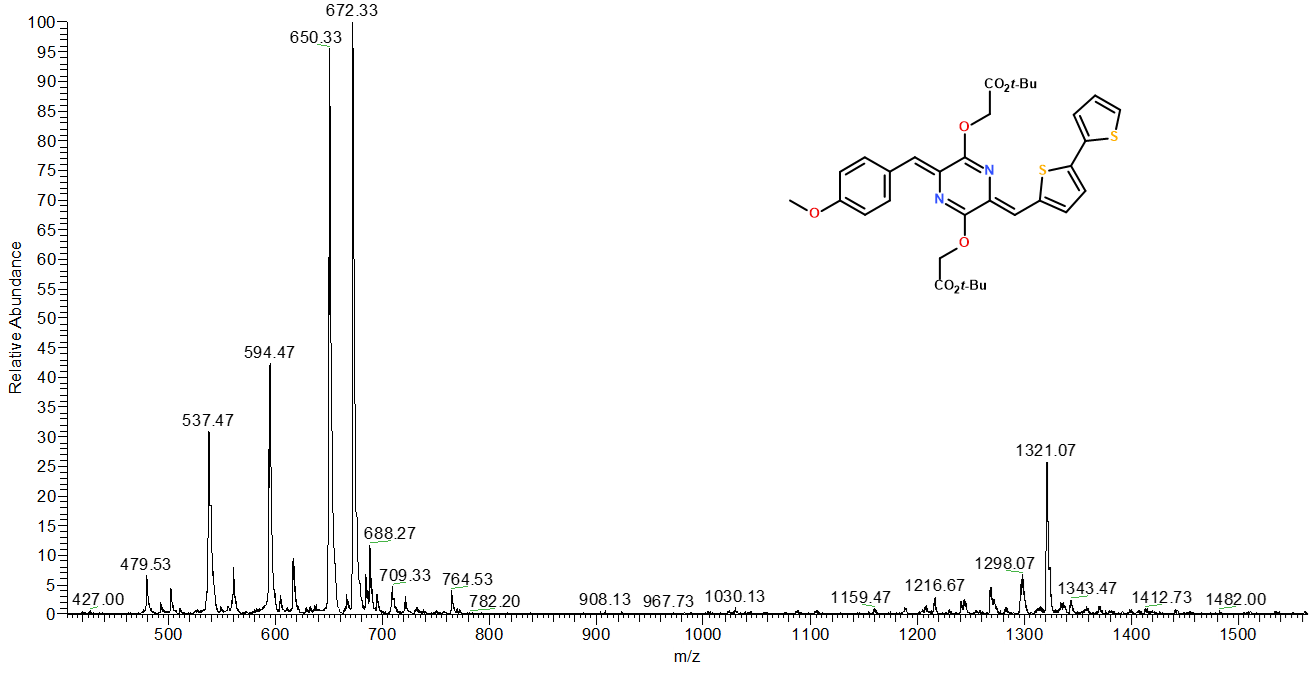


**Figure S14** MS (ESI) spectrum of **AsOMe**: tert-butyl 2-[(3Z,6Z)-5-(2-tert-butoxy-2-oxo-ethoxy)-3-[[4-methoxyphenyl]-methylene]-6-[[5-(2-thienyl)-2-thienyl]methylene]pyrazin-2-yl]oxyacetate


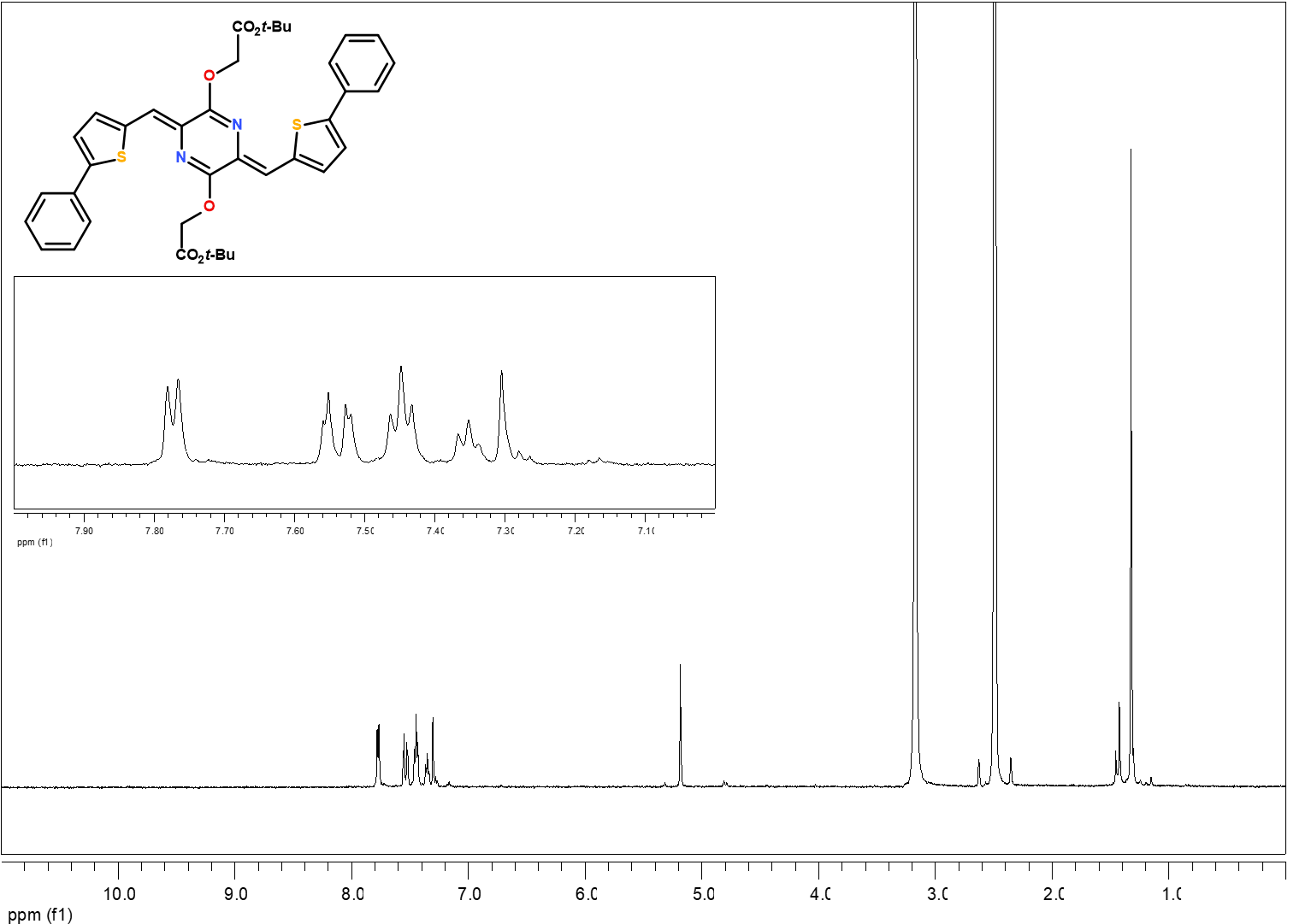


**Figure S15** ^1^H NMR spectrum of **TPh:** tert-butyl 2-[(3Z,6Z)-5-(2-tert-butoxy-2-oxo-ethoxy)-3,6-bis[(5-phenyl-2-thienyl)methylene]-pyrazin-2-yl]oxyacetate (500 MHz, 25 °C, DMSO *d_6_*)


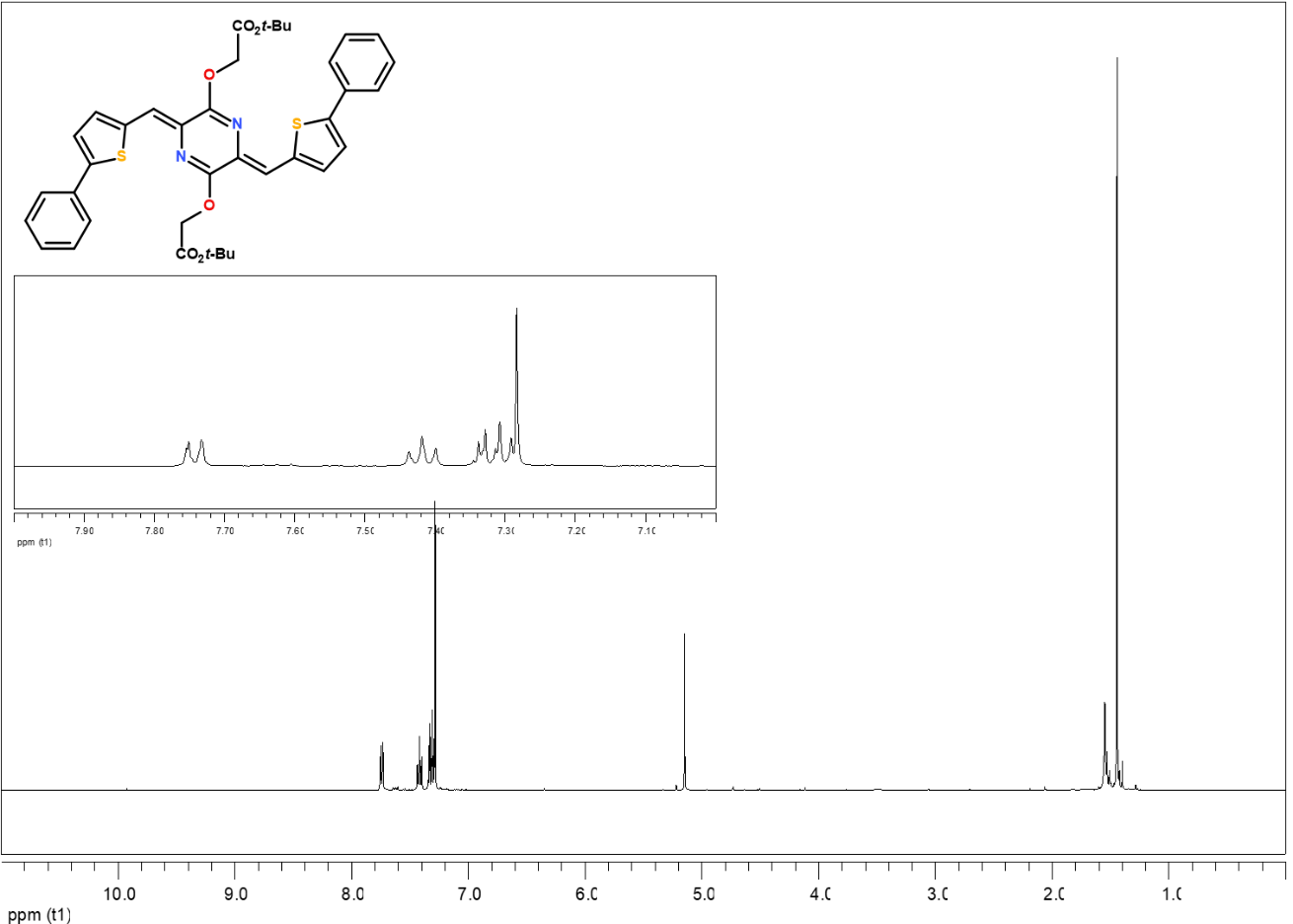


**Figure S16** ^1^H NMR spectrum of **TPh:** tert-butyl 2-[(3Z,6Z)-5-(2-tert-butoxy-2-oxo-ethoxy)-3,6-bis[(5-phenyl-2-thienyl)methylene]-pyrazin-2-yl]oxyacetate (400 MHz, 25°C, Chloroform *d*)


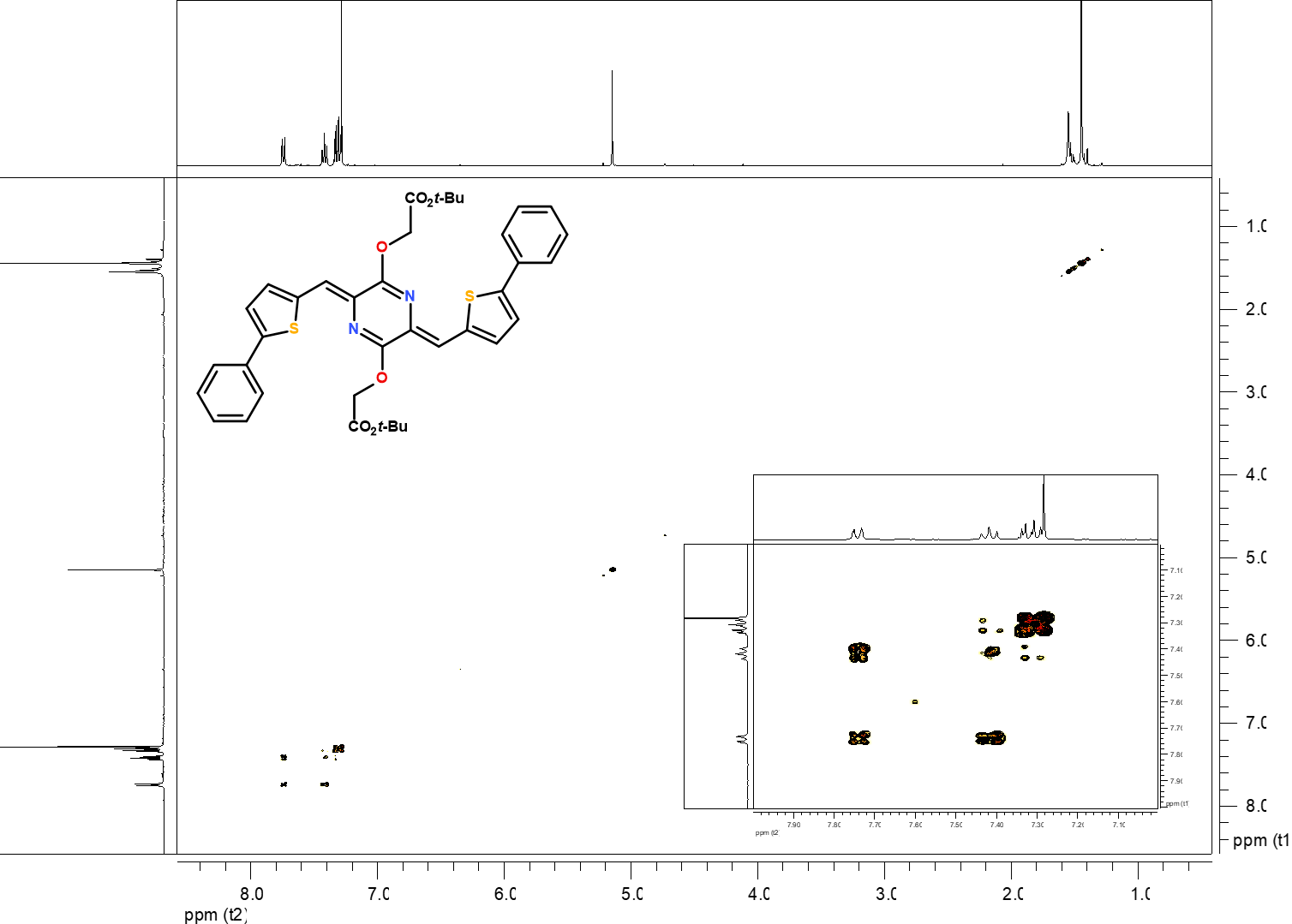


**Figure S17** ^1^H-^1^H gCOSY NMR spectrum of **TPh:** tert-butyl 2-[(3Z,6Z)-5-(2-tert-butoxy-2-oxo-ethoxy)-3,6-bis[(5-phenyl-2-thienyl)methylene]-pyrazin-2-yl]oxyacetate (400 MHz, 25 °C, Chloroform *d*)


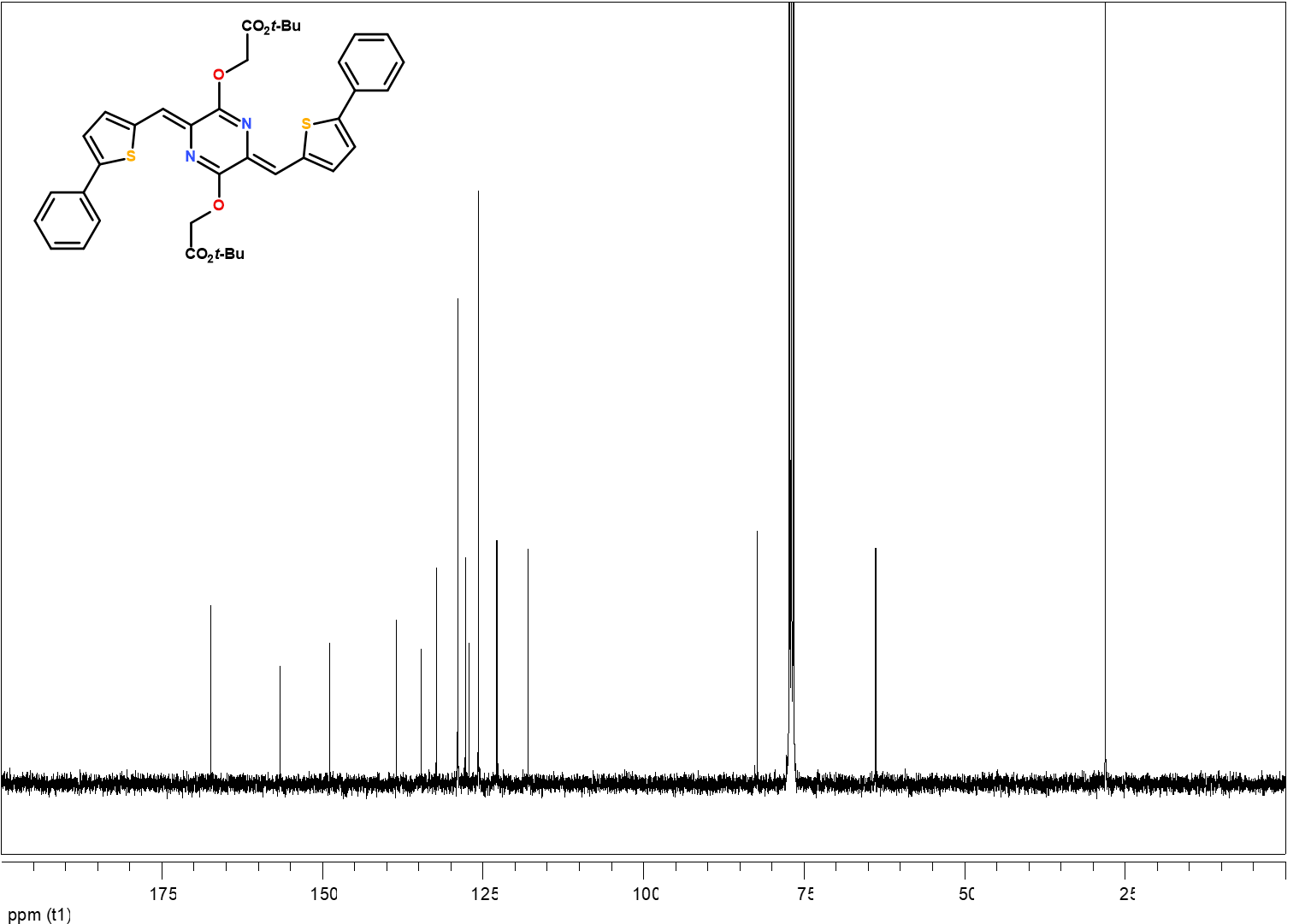


**Figure S18** ^13^C NMR spectrum of **TPh:** tert-butyl 2-[(3Z,6Z)-5-(2-tert-butoxy-2-oxo-ethoxy)-3,6-bis[(5-phenyl-2-thienyl)methylene]-pyrazin-2-yl]oxyacetate (100 MHz, 25 °C, Chloroform *d*)


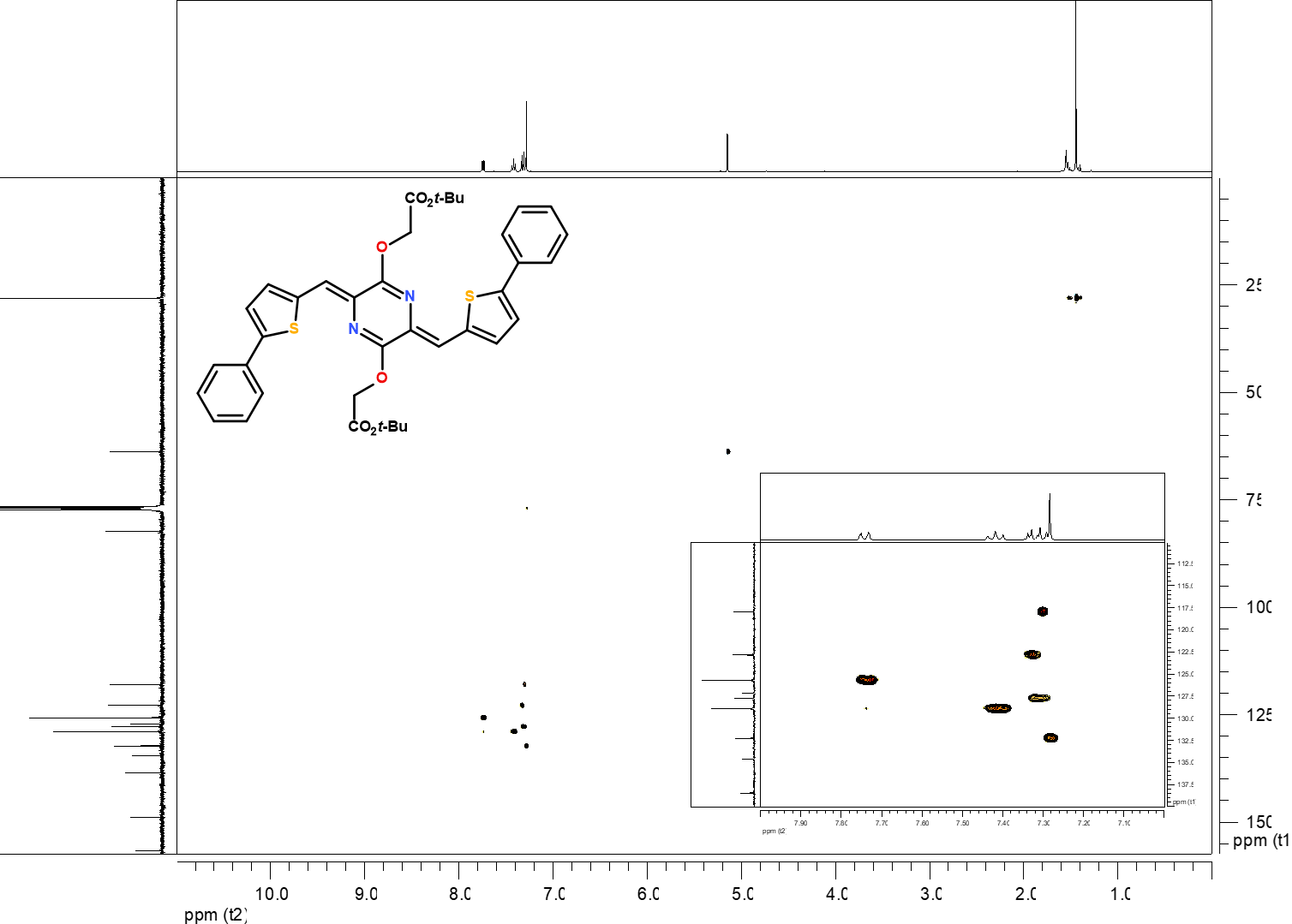


**Figure S19** ^1^H-^13^C gHSQCAD NMR spectrum of **TPh:** tert-butyl 2-[(3Z,6Z)-5-(2-tert-butoxy-2-oxo-ethoxy)-3,6-bis[(5-phenyl-2-thienyl)methylene]-pyrazin-2-yl]oxyacetate (400 MHz, 25°C, Chloroform *d*)


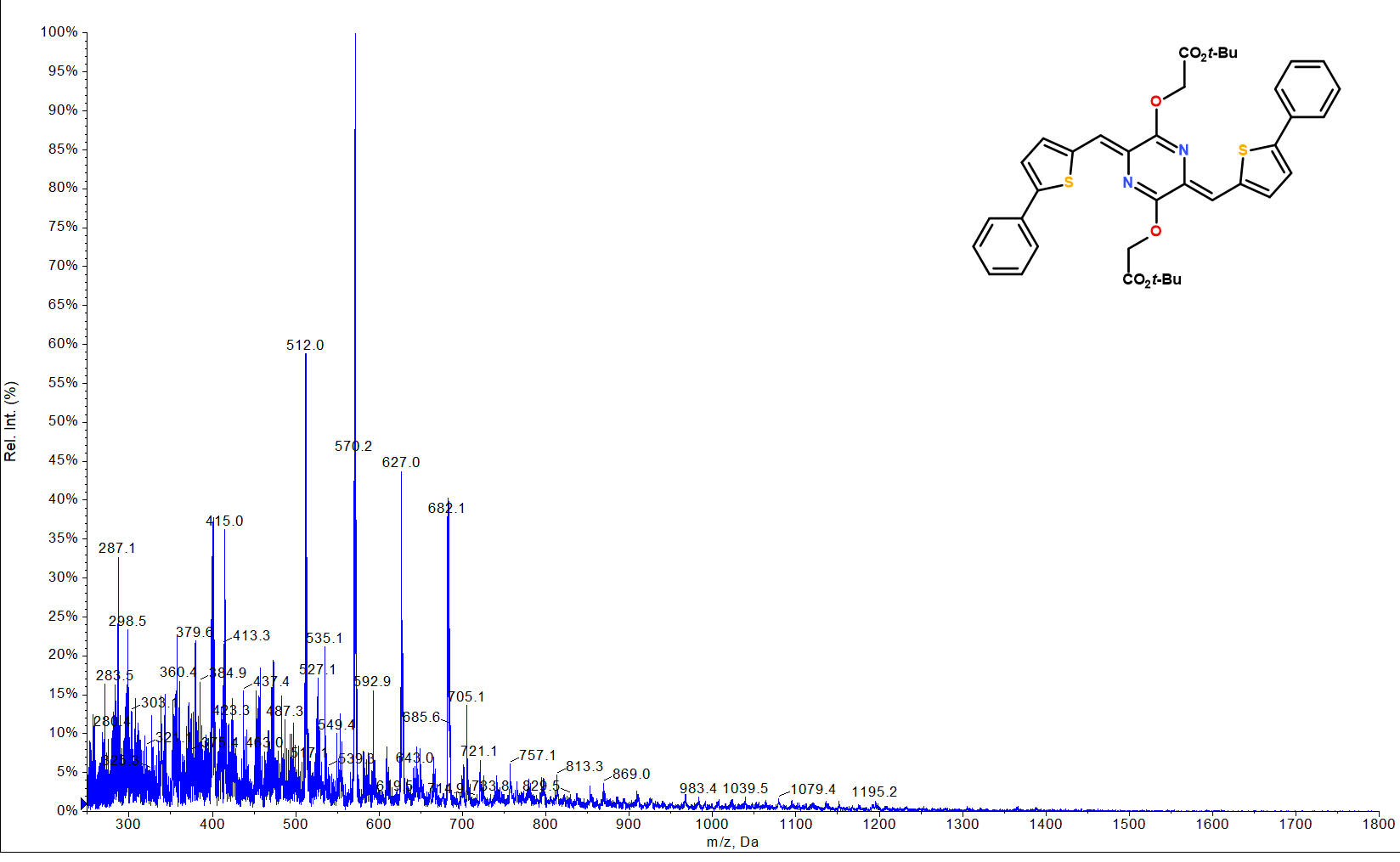


**Figure S20** MS (APCI) spectrum of **TPh:** tert-butyl 2-[(3Z,6Z)-5-(2-tert-butoxy-2-oxo-ethoxy)-3,6-bis[(5-phenyl-2-thienyl)methylene]-pyrazin-2-yl]oxyacetate


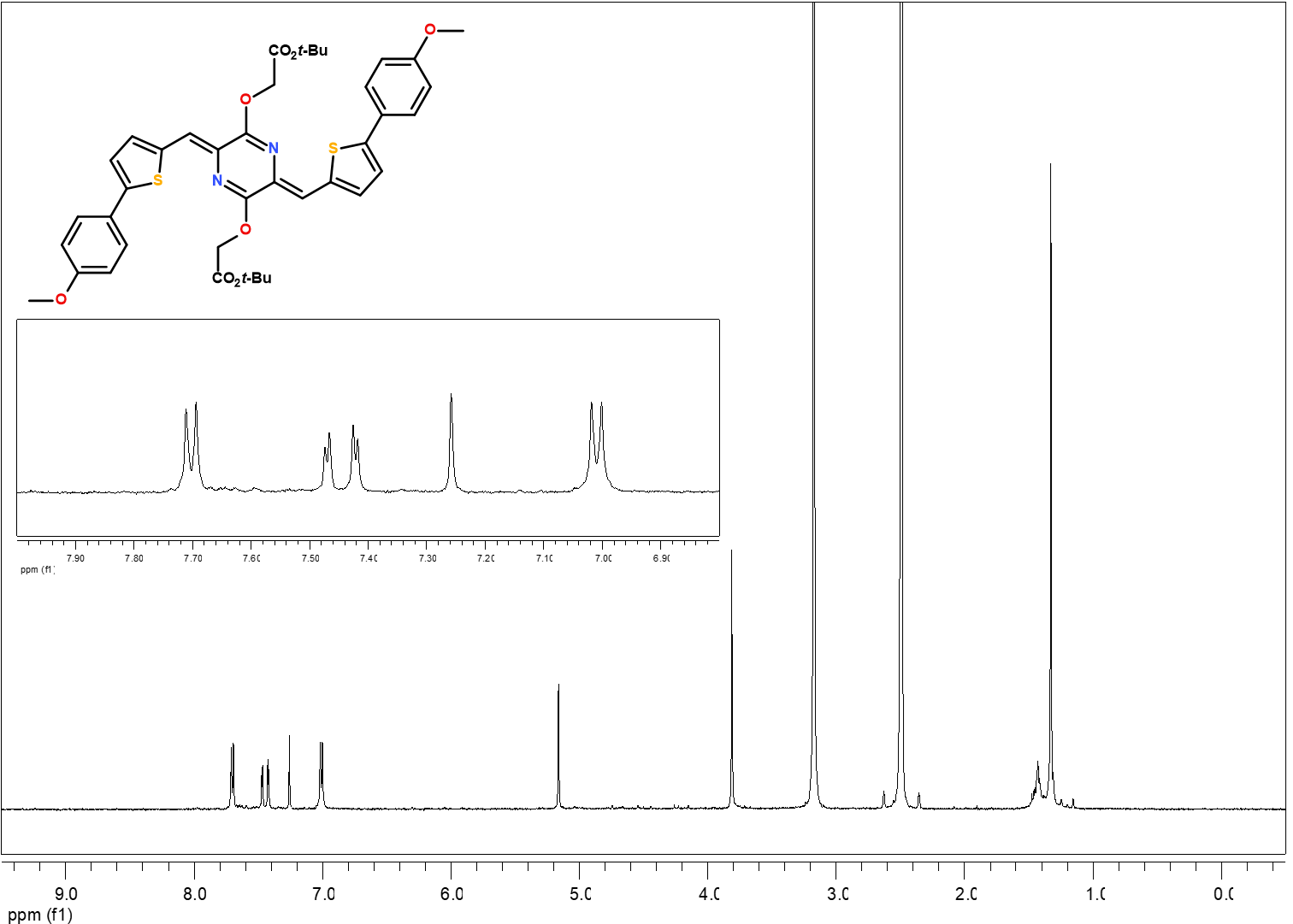


**Figure S21** ^1^H NMR spectrum of **TPhOMe:** tert-butyl 2-[(3Z,6Z)-5-(2-tert-butoxy-2-oxo-ethoxy)-3,6-bis[(5-(4-methoxyphenyl)-2-thienyl)methylene]-pyrazin-2-yl]oxyacetate (500 MHz, 25 °C, DMSO *d_6_*)


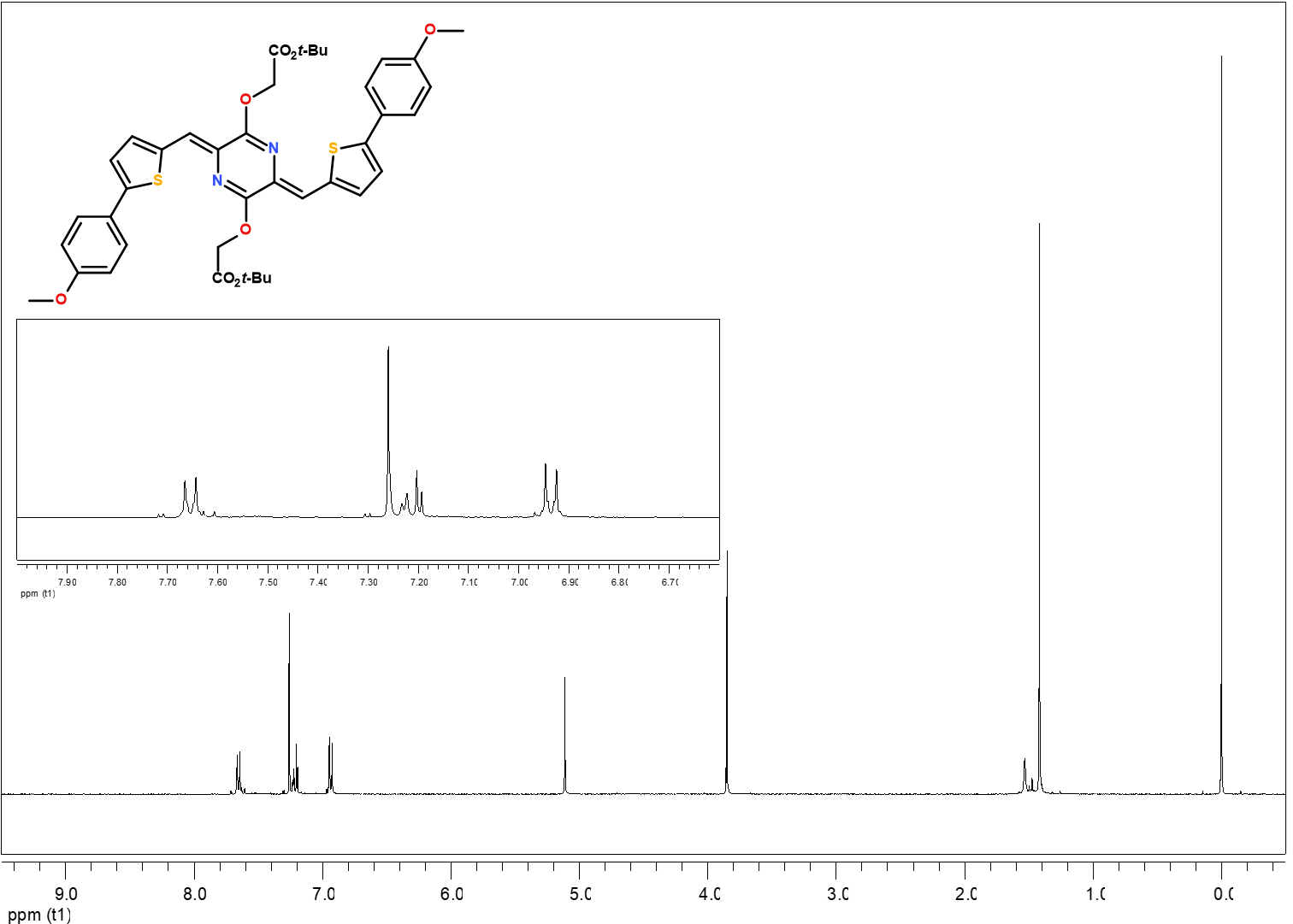


**Figure S22** ^1^H NMR spectrum of **TPhOMe:** tert-butyl 2-[(3Z,6Z)-5-(2-tert-butoxy-2-oxo-ethoxy)-3,6-bis[(5-(4-methoxyphenyl)-2-thienyl)methylene]-pyrazin-2-yl]oxyacetate (400 MHz, 25 °C, Chloroform *d*)


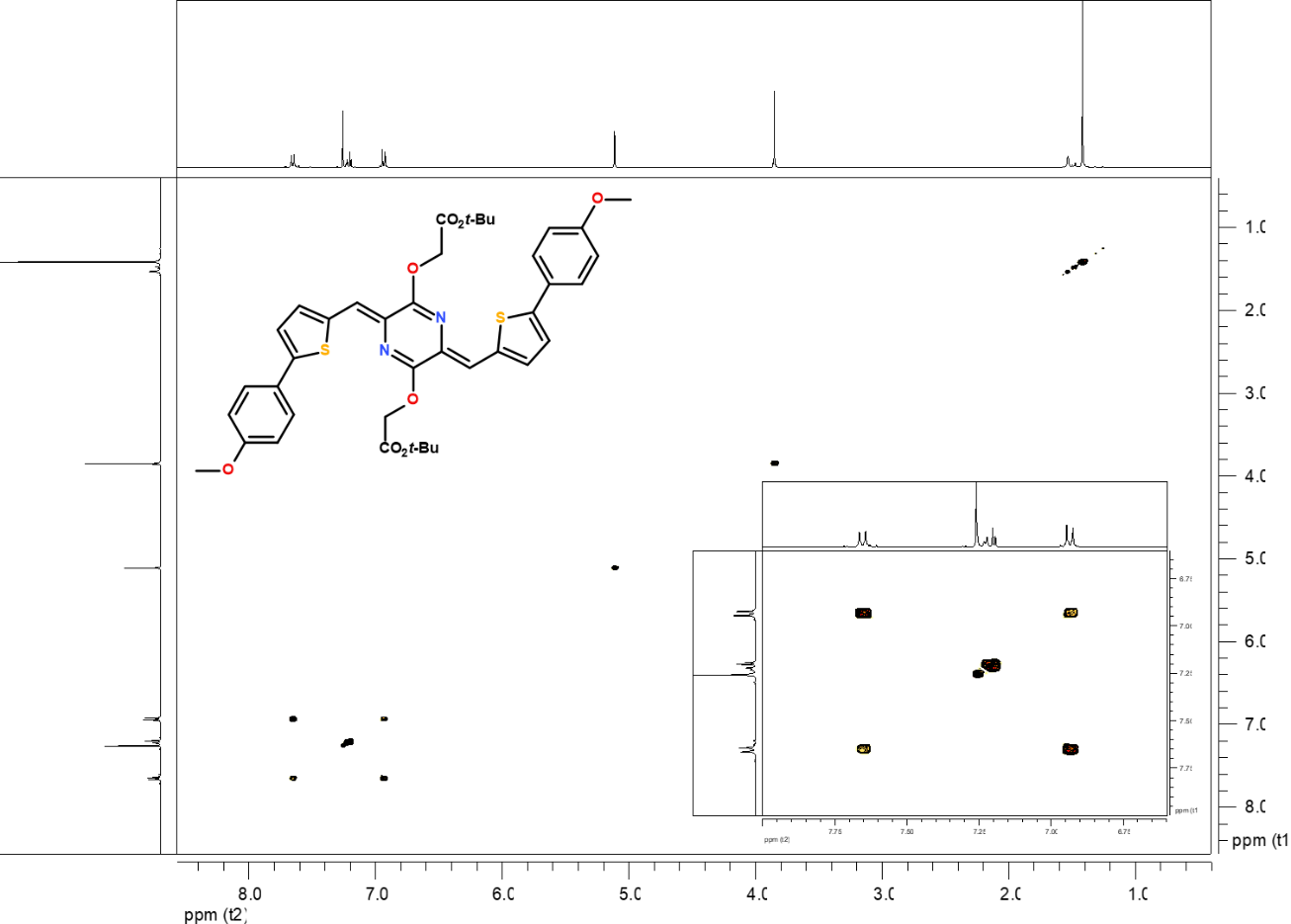


**Figure S23** ^1^H ^1^H gCOSY NMR spectrum of **TPhOMe:** tert-butyl 2-[(3Z,6Z)-5-(2-tert-butoxy-2-oxo-ethoxy)-3,6-bis[(5-(4-methoxyphenyl)-2-thienyl)methylene]-pyrazin-2-yl]oxyacetate (400 MHz, 25°C, Chloroform *d*)


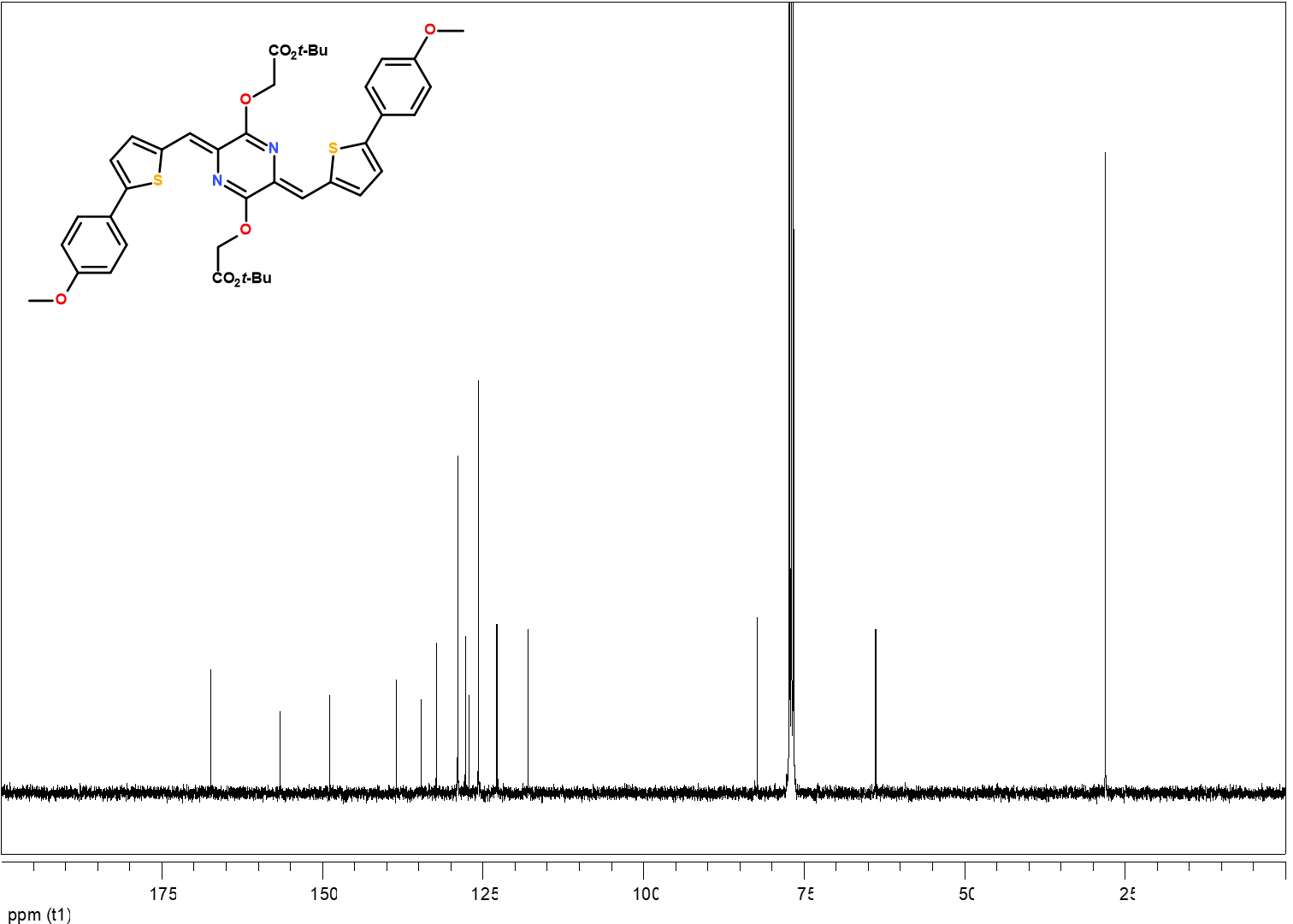


**Figure S24** ^13^C NMR spectrum of **TPhOMe:** tert-butyl 2-[(3Z,6Z)-5-(2-tert-butoxy-2-oxo-ethoxy)-3,6-bis[(5-(4-methoxyphenyl)-2-thienyl)methylene]-pyrazin-2-yl]oxyacetate (100 MHz, 25°C, Chloroform *d*)


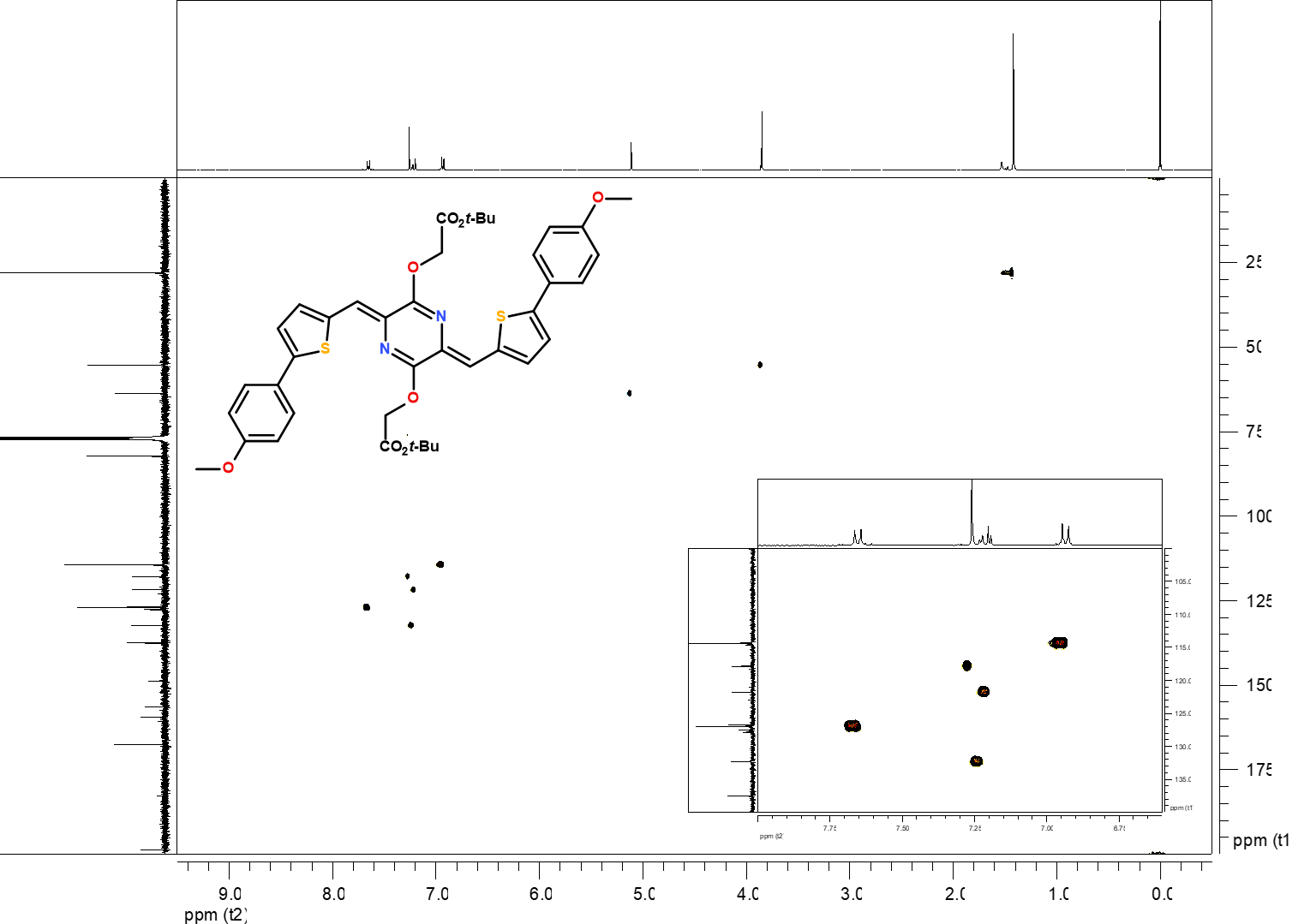


**Figure S25** ^1^H-^13^C gHSQCAD NMR spectrum of **TPhOMe:** tert-butyl 2-[(3Z,6Z)-5-(2-tert-butoxy-2-oxo-ethoxy)-3,6-bis[(5-(4-methoxyphenyl)-2-thienyl)methylene]-pyrazin-2-yl]oxyacetate (400 MHz, 25°C, Chloroform *d*)


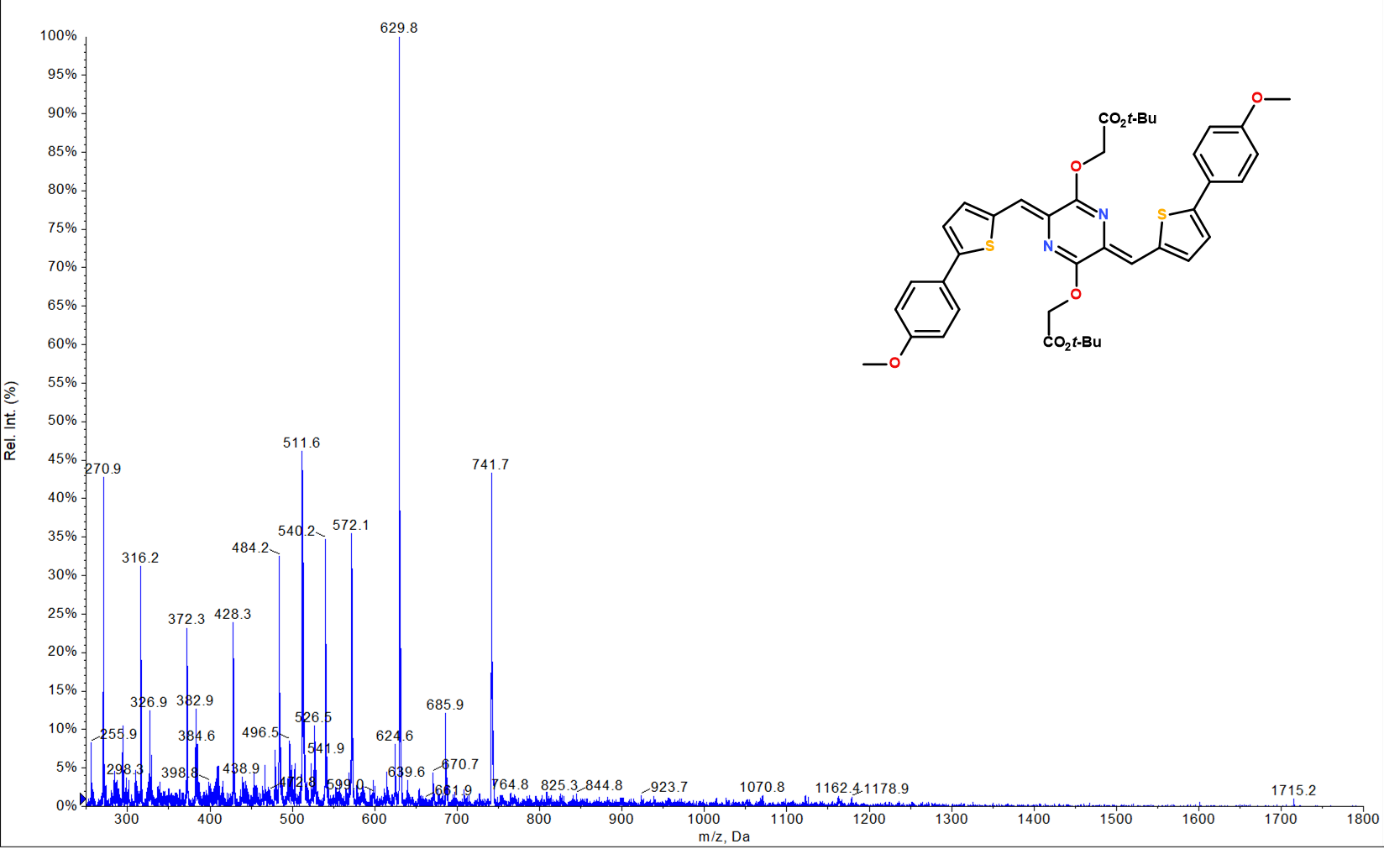


**Figure S26** MS (APCI) spectrum of **TPhOMe:** tert-butyl 2-[(3Z,6Z)-5-(2-tert-butoxy-2-oxo-ethoxy)-3,6-bis[(5-(4-methoxyphenyl)-2-thienyl)methylene]-pyrazin-2-yl]oxyacetate.

## **Photophysical characterization: materials and methods**

Spectral and photophysical properties were recorded in the following solvents. All of them have been purchased from Sigma-Aldrich. Solvents and relative properties. ε: dielectric constant at 298 K, n: refractive index at 298 K. Toluene (Tol) 2.4 1.4968; Anisole (An) 4.33 1.51791; Chloroform (CHCl_3_) 4.8 1.4476; Ethyl Acetate (EtAc) 6.02 1.3720; Tetrahydrofuran (THF) 7.6 1.407; Acetonitrile (MeCN) 35.9 1.3441; Dimethylformammide (DMF) 36.7 1.4310; Dimethylsulphoxide (DMSO) 46.5 1.4793. Stationary absorption measurements were carried out by a Varian Cary 1 UV-Visible spectrophotometer. A FS5 Spectrofluorimeter by Edinburgh Instruments operated by Fluoracle software was used for excitation/emission fluorescence and phosphorescence spectra at room temperature and at 77K, respectively, for the samples in solution as well as in thin film. For the phosphorescence measurements a micropulsed lamp source coupled with a InGaAs detector were employed. Air-equilibrated dilute solutions (absorbance < 0.1 at the excitation wavelength) were used for the fluorimetric measurements. The fluorescence quantum yields (Φ_F_, experimental error ± 10%) were obtained by employing tetracene (Φ_F_ = 0.17 in air-equilibrated cyclohexane) and Rhodamine 6G (Φ_F_ = 0.95 in air-equilibrated ethanol) as reference compounds,^[2]^ taking also into account the different refractive indexes of the used solvents. Fluorescence lifetimes were measured through an Edinburgh Instrument 199S Spectrofluorimeter equipped with the single photon counting technique. Several LEDs centered at 370, 460 and 640 nm were used as light sources (temporal resolution = 0.5 ns). Triplet properties were measured by laser flash photolysis (Edinburgh LP980) upon excitation at 355 nm (third harmonic of a Quanta-Ray/ INDI Nd:YAG laser, Spectra Physics) with nanosecond timeresolution (pulse width 7 ns and laser energy < 1 mJ per pulse) coupled with a PMT for signal detection. The excitation at 355 nm was the pump-pulse while a pulsed xenon lamp was used to probe the absorption properties of the produced excited states. The experimental setup was calibrated by an optically matched solution of benzophenone (Bz) in MeCN (Φ_T_ = 1 and ε_T_ = 6500 M^−1^ cm^−1^ at 520 nm).^[3]^ Triplet–triplet absorption coefficients (ε_T_) were evaluated by energy transfer experiment from benzophenone to the studied compounds in MeCN. Sensitization experiments were also carried out by employing the triplet energy donor 2,2’-dithienylketone (DTK),^[4]^ tetracene and rubrene.^[2]^ The triplet quantum yields, Φ_T_, were determined (estimated uncertainty of ±15%) by an actinometry approach considering Bz in MeCN as the reference. All measurements were performed by purging the sample with pure molecular nitrogen. The detailed methodologies concerning the determination of the triplet extinction coefficient and quantum yield have been extensively discussed in the Supporting Information of a previous work published in our research group.^[5]^ The experimental setup for ultrafast transient absorption and broadband fluorescence up conversion experiments have been previously reported.^[6]^ The 400 nm excitation pulses (ca. 60 fs) were generated as the second harmonic of an amplified Ti:Sapphire laser system (Spectra Physics). The Helios transient absorption spectrometer (Ultrafast Systems) is characterized by a temporal resolution of about 150 fs and a spectral resolution of 1.5 nm. A small portion of the 800 nm light passes through an optical delay line (time window of 3200 ps) and is focused onto a Sapphire crystal (2 mm thick) to generate a white-light in the 450-800 nm spectral range (probe pulse). In the Up-Conversion set up (Halcyone, Ultrafast System), the 400-nm pulse excites the sample whereas the fundamental laser beam acts as the “gate” light, after passing through a delay line, which is then summed to the sample emission promoting the up-conversion process. The time resolution is about 200 fs while the spectra resolution is 1.5 nm. All measurements were carried out under the magic angle relative polarization condition in 2 mm cuvettes at an absorbance of about 0.5 at 400 nm (concentration ≈ 2×10^−4^ M). The solution was stirred during the experiments to avoid photoproduct interferences. Photodegradation was checked recording the absorption spectra before and after the time-resolved measurements, where no significant change was observed. The experimental 3D data matrices were firstly analyzed performing the Global Analysis by Surface Xplorer PRO (Ultrafast Systems) software, and successively through GloTarAn software in order to obtain the Evolution-Associated Spectra (EAS) considering a consecutive kinetic model.

Further analysis of the fluorosolvatochromic behavior was carried out by considering the dependence of the Stokes shift ($\Delta\tilde{\nu}$) on the solvent properties (dielectric constant, ε, and refractive index, n) expressed as $f\left( \varepsilon,n \right)=\left( \frac{\varepsilon-1}{\varepsilon+2}-\frac{n^{2}-1}{n^{2}+2} \right)$, to obtain information about the dipole moment difference between the excited state and the ground state (Δμ_CT_ = μ_E_ – μ_G_) for the asymmetric structures according to Equation S1:

| $\Delta\tilde{\nu}=\tilde{\nu_{abs}}-\tilde{\nu_{em}}=\left( \delta_{abs}-\delta_{em} \right)+\frac{{2\Delta\mu_{CT}}^{2}}{hca^{3}}\left( \frac{\varepsilon-1}{\varepsilon+2}-\frac{n^{2}-1}{n^{2}+2} \right)$ | (S1) |
| --- | --- |

where $\Delta\tilde{\nu}=\tilde{\nu_{abs}}-\tilde{\nu_{em}}$is the Stokes shift (in cm^-1^), *a* is the cavity radius within Onsager’s model, taken as 60% of the calculated diameter along the CT direction resulting from the optimized geometry in cm, *h* is Planck constant and *c* is the speed of light in a vacuum.

In the case of the symmetric structures, another equation was applied which relates the Stokes shift to the quadrupole moment difference (ΔQ_CT_ = Q_E_ – Q_G_) as follows:

| $\Delta\tilde{\nu}=\tilde{\nu_{abs}}-\tilde{\nu_{em}}=\left( \delta_{abs}-\delta_{em} \right)+\frac{{2{\Delta Q}_{CT}}^{2}}{hca^{5}}\left( \frac{\varepsilon-1}{\varepsilon+2}-\frac{n^{2}-1}{n^{2}+2} \right)$ | (S2) |
| --- | --- |

being Q = 2µ*d* and thus ΔQ_CT_^2^ = 4d^2^(Δμ_CT_)^2^. In this case, the quadrupole is considered as two opposite dipoles, sharing the central pAQM, separated by a distance *d* between their barycenter.

## **EPR characterization**

### **1.3.1 Materials and Methods**

Samples in solution were prepared by dissolving the molecule in a mixture of toluene (Tol)/dichloromethane (DCM) 1:1 to obtain a high-quality glass upon freezing; due to photostability issues in DCM, **AsOMe** was dissolved in a mixture of Tol/2-methyltetrahydrofuran (MeTHF) 1:1. All samples were ~200 µM in concentration, determined via absorption spectroscopy, and were put in 4 mm o.d. by 3 mm i.d. quartz EPR tubes, subjected to several freeze-pump-thaw cycles and flame sealed under vacuum.

Experiments were performed at X-band on a Bruker Elexsys E580 spectrometer, equipped with an ER047PS 1 MHz preamplifier, using a Bruker ER4117X-MD5-W1 cavity in a CF935 Oxford Instruments cryostat. The TR-EPR spectra were recorded in direct detection mode with the time traces sampled using the SpecJet I module of the console after amplification by a FEMTO DHPVA wideband voltage amplifier with a 20 MHz low-pass filter. The temperature was set to 80 K, unless otherwise stated, and controlled with a MercuryiTC Oxford Instrument temperature controller. Experiments photoexciting at 450 nm and 600 nm were performed on a Bruker Elexsys E580 X/Q-band spectrometer, with an ER047PH 20 Hz–6.5 MHz preamplifier, using a Bruker ER4118X-MD5-W1 cavity in a CF900 Oxford Instruments cryostat and an Oxford Instrument ITC503 temperature control unit.

The microwave power was set at about 0.15 mW for the samples in solution and 1.5 mW for the samples in film.

Photoexcitation at 532 nm was performed using the second harmonic module of a Quantel Rainbow Nd:YAG laser working at a 50 Hz repetition rate. Photoexcitation at 450 nm was provided by an optical parametric oscillator (OPO, Opotek MagicPRISM) pumped by the third harmonic module of a Quantel Rainbow Nd:YAG laser operating with a 10 Hz repetition rate.

Laser pulses were 5 ns long, with energies of 2 mJ/shot for samples in solution and 5 mJ/shot for samples in film, except for the measurements on **AsOMe** at 450 nm (2 mJ/shot) and at 600 nm for **TPh** and **AsNMe_2_** (0.6 mJ/shot) were used. MPS experiments were performed exciting the sample with linearly polarized light using a half-wave plate followed by a Glan-Taylor linear polarizer. The spectra corresponding to isotropic excitation were reconstructed by taking the sum $I_{par}$+${2I}_{perp}$, where $I_{par}$ and $I_{perp}$ are the TR-EPR signal intensities with the linear polarization direction of the light parallel and perpendicular to the external magnetic field, respectively.

TR-EPR spectra were processed via baseline correction using off-resonance transients to remove the background signal and the spectra were extracted at a single delay after the laser flash. Spectral simulations were performed with a home-written Matlab code based on EasySpin version 6.0.^[7,8]^

### **1.3.2 The Spin Hamiltonian**

In the case of a photoexcited triplet states for organic chromophores in an external magnetic field, $\vec{B}_{0}$, the spin Hamiltonian of the system is given by:

| $\hat{H}=\mu_{B}\vec{B}_{0}\underline{g}\hat{\vec{S}}+\hat{\vec{S}}\underline{D}\hat{\vec{S}}+J{\hat{\vec{S}}}_{1}\cdot{\hat{\vec{S}}}_{2}$ | S3 |
| --- | --- |

where, $\hat{\vec{S}}$ is the total spin operator $\hat{\vec{S}}={\hat{\vec{S}}}_{1}+{\hat{\vec{S}}}_{2}$, $\underline{g}$ is the g-tensor of the triplet, $\underline{D}$ is the Zero Field Splitting (ZFS) tensor and $J$ is the exchange interaction parameter. The first term in the Hamiltonian is the Zeeman interaction, the second term is the ZFS interaction a dipolar interaction between the two unpaired electrons, and the last term is the exchange interaction, which accounts for electrostatic interactions, and is responsible for the separation between the singlet and triplet state. Given the high energy gap between singlet and triplet states the exchange interaction can be considered as a constant which does not contribute to the EPR spectrum.^[9]^ Because of the ZFS interaction, the degeneracy of the three triplet spin sublevels is lifted even at zero field, which are defined as the *X*, *Y* and *Z* sublevels in the ZFS eigenbasis or the T_-1_, T_0_ and T_+1_ states in the high-field eigenbasis. The ZFS interaction is described by two parameters *D* and *E*, the former is related to the electron spin distribution in the triplet state which can be either oblate (*D*>0) or prolate (*D*<0), whereas *E* is related to the symmetry of the spin distribution.^[9]^

In the case of a pair of interacting triplets, both intramolecular and intermolecular contributions need to be considered for the Hamiltonian:

| $\hat{H}=\mu_{B}\vec{B}_{0}\underline{g_{A}}{\hat{\vec{S}}}_{A}+\mu_{B}\vec{B}_{0}\underline{g_{B}}{\hat{\vec{S}}}_{B}+{\hat{\vec{S}}}_{A}\underline{D_{A}}{\hat{\vec{S}}}_{A}+{\hat{\vec{S}}}_{B}\underline{D_{B}}{\hat{\vec{S}}}_{B}+{\hat{\vec{S}}}_{A}\underline{D_{AB}}{\hat{\vec{S}}}_{B}+J{\hat{\vec{S}}}_{A}\cdot{\hat{\vec{S}}}_{B}$ | S4 |
| --- | --- |

where the first two terms are the Zeeman interaction for triplet A and triplet B in the pair, the third and fourth terms are the ZFS interaction for triplet A and triplet B respectively, the fifth term is the intermolecular dipolar interaction between the two triplets, and the last term is the exchange interaction between the two triplets.^[8,10]^ The exchange interaction, $J$, separates the energy levels with different spin multiplicities, thus in the strong exchange coupling regime, it will not contribute to the TR-EPR spectrum, which will only be affected by the ZFS, Zeeman and intermolecular dipolar interactions.

## **1.4 Thin film preparation**

Microscope glasses were cleaned using KOH pellets dissolved in absolute ethanol as follows. Saturated KOH solution was prepared by mixing 30 g of KOH in 120 ml of absolute ethanol. For the treatment in the saturated KOH solution, the microscope glasses were immersed in an ultrasonic bath for 3 min. Then they were rinsed with MilliQ water using a squirt bottle and transferred into a beaker to be sonicated in distilled deionized water for an additional 3 min twice, with rinsing in-between. Finally, the glasses were rinsed with 95% ethanol, dried using nitrogen gas, and stored in a desiccator. Thin films have been prepared by deposition of 50÷100 μL of a solution 2÷5 ×10^−3^ M in CHCl_3_ on a clean microscope glass and spin coating for 10 seconds at 500 rpm, then for 40 sec at 2000 rpm.

Film samples for TR-EPR measurements were prepared by dissolving the molecules in DCM and the solution was transferred in the EPR tube. The film was formed by deposition on the internal walls of the EPR tube via solvent evaporation; multiple depositions were performed to increase the optical density of the film, finally the tubes were flame sealed under vacuum.

The film morphologies were analyzed via field emission scanning electron microscopy (FE-SEM) using a ZEISS SUPRA 55 VP field emission microscope (ZEISS, Jena, Germany); the films deposited onto Si following general procedures were analyzed as prepared.

## **1.5 Quantum mechanical calculations**

Quantum-mechanical calculations were carried out on the pAQM derivatives using the Gaussian 09 package Revision D.01.^[11]^ Density functional theory (DFT) based on the wB97XD^[12]^ /6-31G*^[13]^ method was used to optimize the geometry and to obtain the properties of the substrates in the ground state. Furthermore, the relaxed excited states S_1_ and T_1_ were optimized and characterized by the time dependent (TD) wB97XD/6-31G*//wB97XD/6-31G* method. The Frank-Condon excited singlet and triplet states were characterized by TD-DFT WB97X/D6-31G* excited-state calculations, too. Solvation effects induced by toluene were included in the calculations by means of the Conductor-like Polarizable Continuum Model (CPCM).^[14]^

# **Absorption and emission in solution: structure effect**

**Figure S27** Absorption (**a**) and emission (**b**) spectra of the compounds in toluene.

**Table S1** Spectral properties of the compounds in DMSO. λ_abs_: maximum wavelengths of absorption and emission spectra; ɛ: molar absorption coefficients of the ground state.

|  | **λ_abs_ / nm** | **Ɛ_max_/ M^-1^cm^-1^** |
| --- | --- | --- |
| AsOMe | 476 | 28000 |
| AsNMe_2_ | 505 | 24100 |
| TPh | 503 | 45800 |
| TPhOMe | 514 | 60500 |

**Table S2** Fluorescence properties of the compounds in toluene. λ_em_: maximum wavelengths of emission spectra; Φ_F_: fluorescence quantum yield; τ_F_: fluorescence lifetime.

|  | **λ_em_**/nm | **ɸ_F_** | **τ_F_ /** ps | **k_F_** / 10^8^ s^-1^ |
| --- | --- | --- | --- | --- |
| AsOMe | 528 | 0.24 | 1090 | 2.2 |
| AsNMe_2_ | 569 | 0.020 | 87* | 2.3 |
| TPh | 558 | 0.26 | 1520 | 1.7 |
| TPhOMe | 574 | 0.28 | 1160 | 2.4 |

*From fs Transient absorption measurements.

**Figure S28** Absorption (solid line), excitation (dashed line) and emission of the compounds in toluene.

# **Absorption and emission in solution: solvent effect**

**Figure S29** Absorption (**a**) and emission (**b**) of **AsOMe** in solvents at different polarity.

**Table S3** Spectral and fluorescence properties of **AsOMe** in different solvents. λ_abs_, λ_em_: maximumwavelengths ofabsorption and emission spectra; Δυ: Stokes shift; Φ_F_: fluorescence quantum yield; τ_F_: fluorescence lifetime; k_F_: fluorescence rate, Φ_F_/ τ_F_.

| **AsOMe** | **f(ε,n)** | **λ_abs_/nm** | **λ_em_/nm** | **Δυ/cm^-1^** | **ɸ_F_** | **τ_F_ / ps** | **k_F_ / 10^8^ s^-1^** |
| --- | --- | --- | --- | --- | --- | --- | --- |
| MeCN | 0.712 | 470 | 566 | 3610 |  |  |  |
| DMSO | 0.655 | 478 | 577 | 3590 | 0.07 | 310* | 2.3 |
| EtAc/MeCN 1:1 | 0.556 | 470 | 565 | 3580 |  |  |  |
| THF | 0.441 | 472 | 566 | 3520 | 0.12 | - | - |
| CHCl_3_ | 0.293 | 473 | 567 | 3505 | 0.14 | 790 | 1.8 |
| An | 0.224 | 477 | 571 | 3450 |  |  |  |
| Tol | 0.0242 | 475 | 567 | 3420 | 0.24 | 1100 | 2.2 |

*From fs Transient Absorption measurements.

**Figure S30** Absorption (a) and emission (b) of **AsNMe_2_** in solvents at different polarity.

**Table S4** Spectral and fluorescence properties of **AsNMe_2_** in different solvents. λ_abs_, λ_em_: maximum wavelengths of absorption and emission spectra; Δυ: Stokes shift; Φ_F_: fluorescence quantum yield; τ_F_: fluorescence lifetime; k_F_: fluorescence rate, Φ_F_/ τ_F_.

| **AsNMe_2_** | **f(ε,n)** | **λ_abs_/nm** | **λ_em_/nm** | **Δυ/cm^-1^** | **ɸ_F_** | **τ_F_ /ps** | **k_F_ / 10^8^ s^-1^** |
| --- | --- | --- | --- | --- | --- | --- | --- |
| MeCN | 0.712 | 506 | 617 | 3560 |  |  |  |
| DMSO | 0.655 | 520 | 631 | 3380 | 0.014 | 99* | 1.4 |
| EtAc/MeCN 1:1 | 0.556 | 506 | 606 | 3260 |  |  |  |
| THF | 0.441 | 503 | 591 | 2960 | 0.010 | - | - |
| CHCl_3_ | 0.293 | 505 | 577 | 2471 | 0.012 | - | - |
| An | 0.224 | 511 | 586 | 2505 |  |  |  |
| Tol | 0.0242 | 505 | 569 | 2230 | 0.020 | 87* | 2.3 |

*From fs Transient absorption measurements.

**Figure S31** Absorption (left) and emission (right) of **TPh** in solvents at different polarity.

**Table S5** Spectral and fluorescence properties of **TPh** in different solvents. λ_abs_, λ_em_: maximum wavelengths of absorption and emission spectra; Δυ: Stokes shift; Φ_F_: fluorescence quantum yield; τ_F_: fluorescence lifetime; k_F_: fluorescence rate, Φ_F_/ τ_F_.

| **TPh** | **f(ε,n)** | **λ_abs_/nm** | **λ_em_/nm** | **Δυ/cm^-1^** | **ɸ_F_** | **τ_F_ /ps** | **k_F_ / 10^8^ s^-1^** |
| --- | --- | --- | --- | --- | --- | --- | --- |
| DMSO | 0.655 | 508 | 612 | 3345 | 0.16 | 1030 | 1.5 |
| THF | 0.441 | 501 | 601 | 3320 | 0.18 | 1470 | 1.2 |
| CHCl_3_ | 0.293 | 501 | 603 | 3376 | 0.22 | 1460 | 1.5 |
| Tol | 0.0242 | 503 | 605 | 3350 | 0.26 | 1520 | 1.7 |

**Figure S32** Absorption (left) and emission (right) of **TPhOMe** in solvents at different polarity.

**Table S6** Spectral and fluorescence properties of **TPhOMe** in different solvents. λ_abs_, λ_em_: maximum wavelengths of absorption and emission spectra; Δυ: Stokes shift; Φ_F_: fluorescence quantum yield; τ_F_: fluorescence lifetime; k_F_: fluorescence rate, Φ_F_/ τ_F_.

| **TPhOMe** | **f(ε,n)** | **λ_abs_/nm** | **λ_em_/nm** | **Δυ/cm^-1^** | **ɸ_F_** | **τ_F_ /ps** | **k_F_ / 10^8^ s^-1^** |
| --- | --- | --- | --- | --- | --- | --- | --- |
| MeCN | 0.712 | 511 | 581 | 2360 |  |  |  |
| DMSO | 0.655 | 520 | 591 | 2310 | 0.16 | 770 | 2.0 |
| EtAc/MeCN 1:1 | 0.556 | 511 | 576 | 2210 |  |  |  |
| THF | 0.441 | 514 | 574 | 2034 | 0.18 | 1030 | 1.8 |
| CHCl_3_ | 0.293 | 514 | 578 | 2154 | 0.22 | 1060 | 2.1 |
| An | 0.224 | 517 | 580 | 2100 |  |  |  |
| Tol | 0.0242 | 514 | 574 | 2034 | 0.28 | 1170 | 2.4 |

**Figure S33** Linear plots of the Stokes shift *vs* solvent properties (dieletric constant, ε, and refractive index, *n*) for the investigated compounds.

**Table S7.** Predicted and experimental data required for the determination of dipole moment difference (Δμ_CT_ = μ_ES_− μ_GS_) of the investigated compounds from the solvatochromic method: Onsager’s cavity ray (*a*), distance between the barycenter of the two poles (*d*) used as the double dipole model is considered, slope of the linear plot of the Stokes shift vs solvent properties (Figure S33).

| Compound | *a* / Å | *d* / Å | Slope/cm^-1^ | Δμ_CT_ / D |
| --- | --- | --- | --- | --- |
| **AsOMe** | 12.4 | - | 290 | 7.4 |
| **AsNMe_2_** | 12.5 | - | 1960 | 19 |
| **TPhOMe** | 16.4 | 12.6 | 440 | 9.0 |

**Figure S34** Time Resolved Single Photon Counting kinetics of the compounds **AsOMe** (a), **AsNMe_2_** (b), **TPh** (c) and **TPhOMe** (d) in toluene.

# **Quantum mechanical calculation results**

## **AsOMe**

**
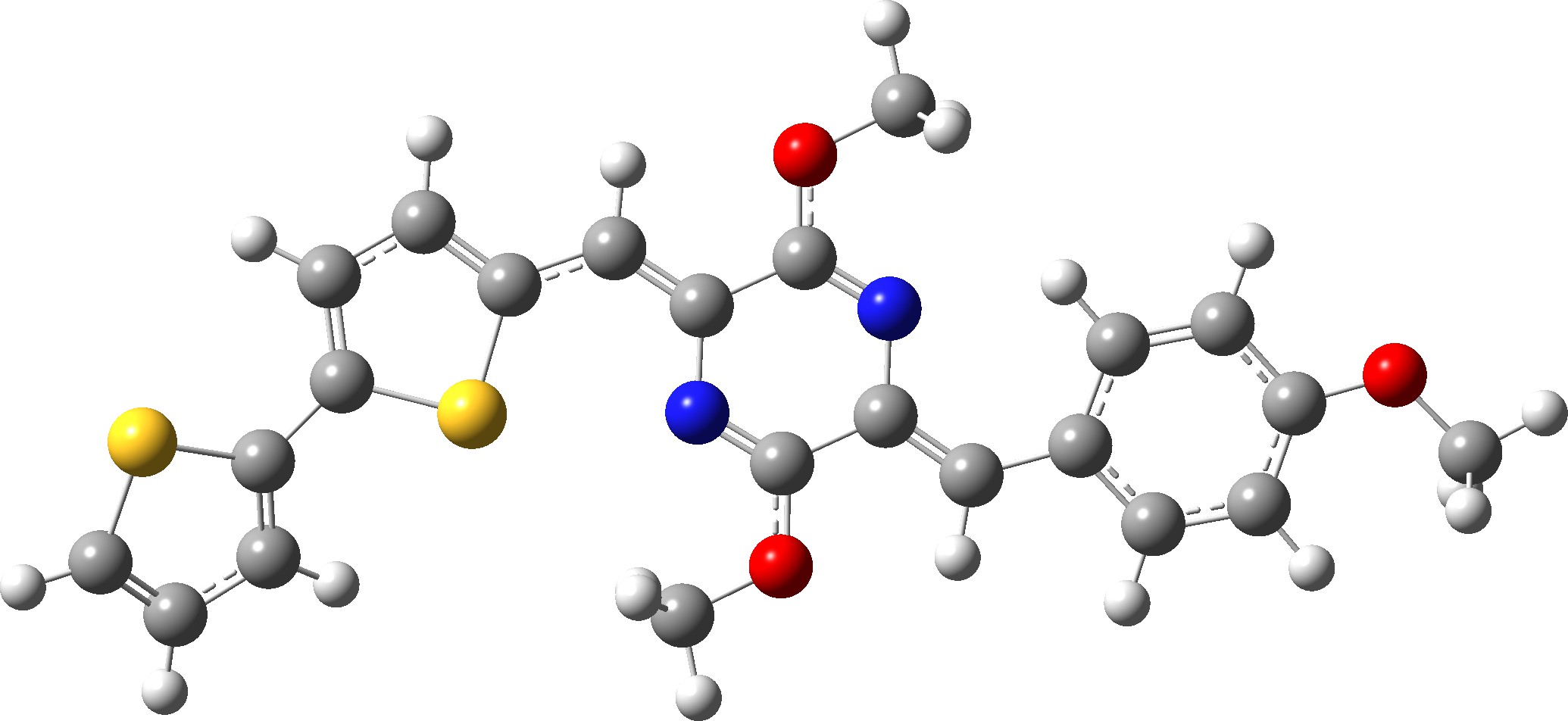
**

**Figure S35** Ground state optimized geometry of **AsOMe** by wB97XD/6-311+G(d) @ S_0_ in toluene

**Table S8** Absorption wavelengths (λ), oscillator strength (f) and molecular orbitals of **AsOMe** in toluene (CPCM) calculated by the wB97XD/6-311+G(d)//wB97XD/6-311+G(d) model, together with the experimental absorption and emission maxima.

| **Transition** | **λ_th_/nm** | **f** | **MO** | **%** | **λ_exp_/nm** |
| --- | --- | --- | --- | --- | --- |
| **S_0_**→**T_1_** | 982 | 0.0000 | π_H_→π_L_^*^ | 94 |  |
| **S_0_**→**T_2_** | 515 | 0.0000 | π_H-1_→π_L_^*^ π_H_→π_L+1*_ | 46  34 |  |
| **S_0_**→**S_1_** | 444 | 1.9820 | π_H_→π_L_^*^ | 93 | 475 |
| **S_0_**→**T_3_** | 404 | 0.0000 | π_H-2_→π_L_^*^ π_H-1_→π_L+1_^*^ | 25  26 |  |
| **S_0_**→**T_4_** | 333 | 0.0000 | π_H-7_→π_L_^*^ π_H-1_→π_L+2_^*^ | 20  15 |  |
| **S_0_**→**T_5_** | 328 | 0.0000 | π_H-8_→π_L_^*^ π_H-2_→π_L+1_^*^ | 14  12 |  |
| **S_0_**→**S_2_** | 322 | 0.1792 | π_H-1_→π_L_^*^ | 70 |  |
| **S_0_**→**T_6_** | 313 | 0.0000 | π_H-3_→π_L_^*^ | 55 |  |
| **S_0_**→**T_7_** | 305 | 0.0000 | n_H-6_→π_L_^*^ | 85 |  |
| **S_0_**→**T_8_** | 301 | 0.0000 | π_H_→π_L+3_^*^ π_H_→π_L+9_^*^ | 16  15 |  |
| **S_0_**→**T_9_** | 299 | 0.0000 | π_H_→π_L+2_^*^ π_H_→π_L+3_^*^ | 16  14 |  |
| **S_0_**→**S_3_** | 293 | 0.0013 | n_H-6_→π_L_^*^ | 86 |  |
| **S_0_**→**T_10_** | 285 | 0.0000 | π_H-4_→π_L+1_^*^ | 38 |  |
| **S_0_**→**S_4_** | 281 | 0.0871 | π_H_→π_L+1_^*^ | 53 |  |
| **S_0_**→**S_5_** | 268 | 0.0496 | π_H_→π_L+2_^*^ | 47 |  |
| **S_0_**→**S_6_** | 262 | 0.0192 | π_H_→π_L+3_^*^ | 29 |  |
| **S_0_**→**S_7_** | 259 | 0.1728 | π_H-3_→π_L_^*^ | 39 |  |
| **S_0_**→**S_8_** | 250 | 0.0109 | π_H-2_→π_L_^*^ | 20 |  |
| **S_0_**→**S_9_** | 235 | 0.0993 | π_H-7_→π_L_^*^ | 43 |  |
| **S_0_**→**S_10_** | 233 | 0.1130 | π_H-4_→π_L_^*^ π_H-4_→π_L+1_^*^ | 38  30  25 |  |
| **S_1_**→**S_0_** | 590 | 1.9357 | π_H_→π_L_^*^ | 96 | 528 |


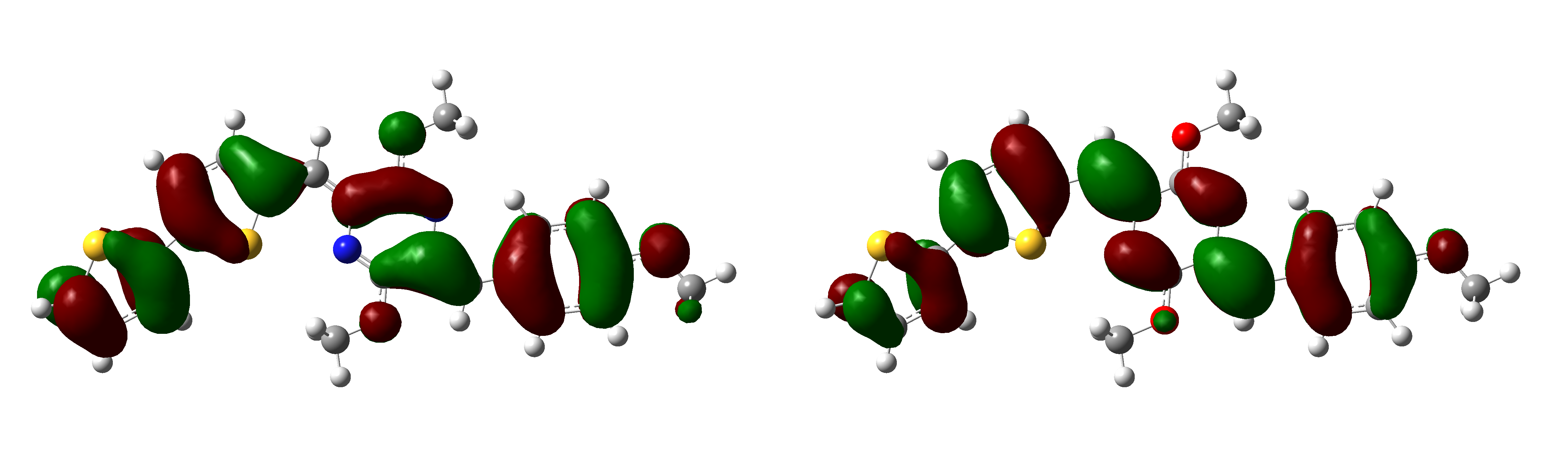


**π_H-1_ π_H_**

**
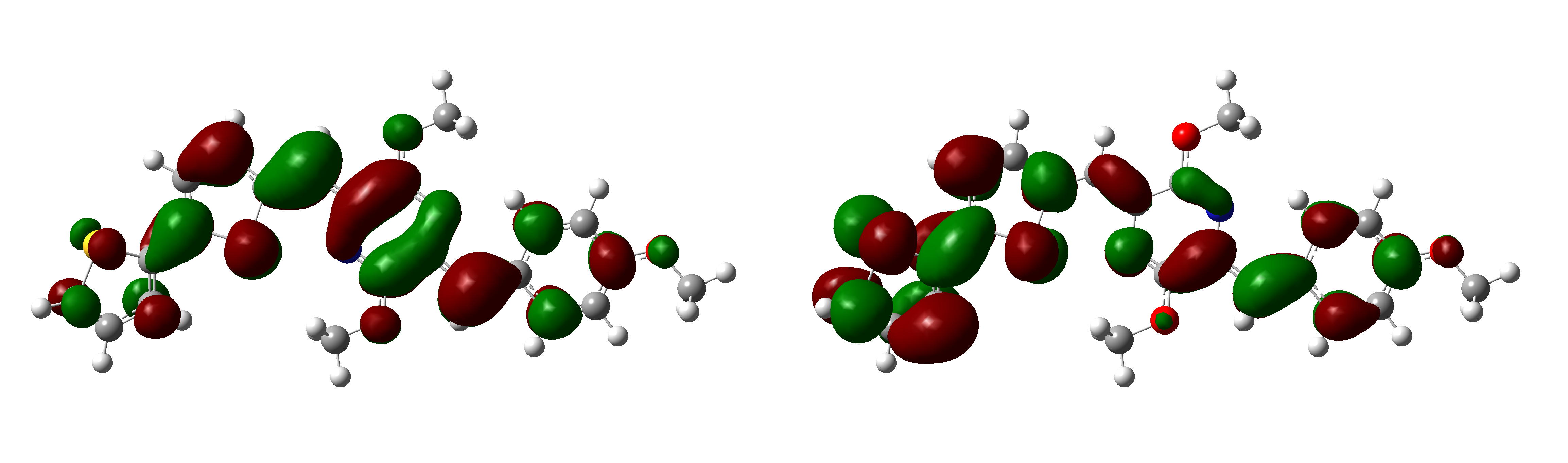
**

**π_L_ π_L+1_**

**Figure S36** Frontier molecular orbitals of **AsOMe**.


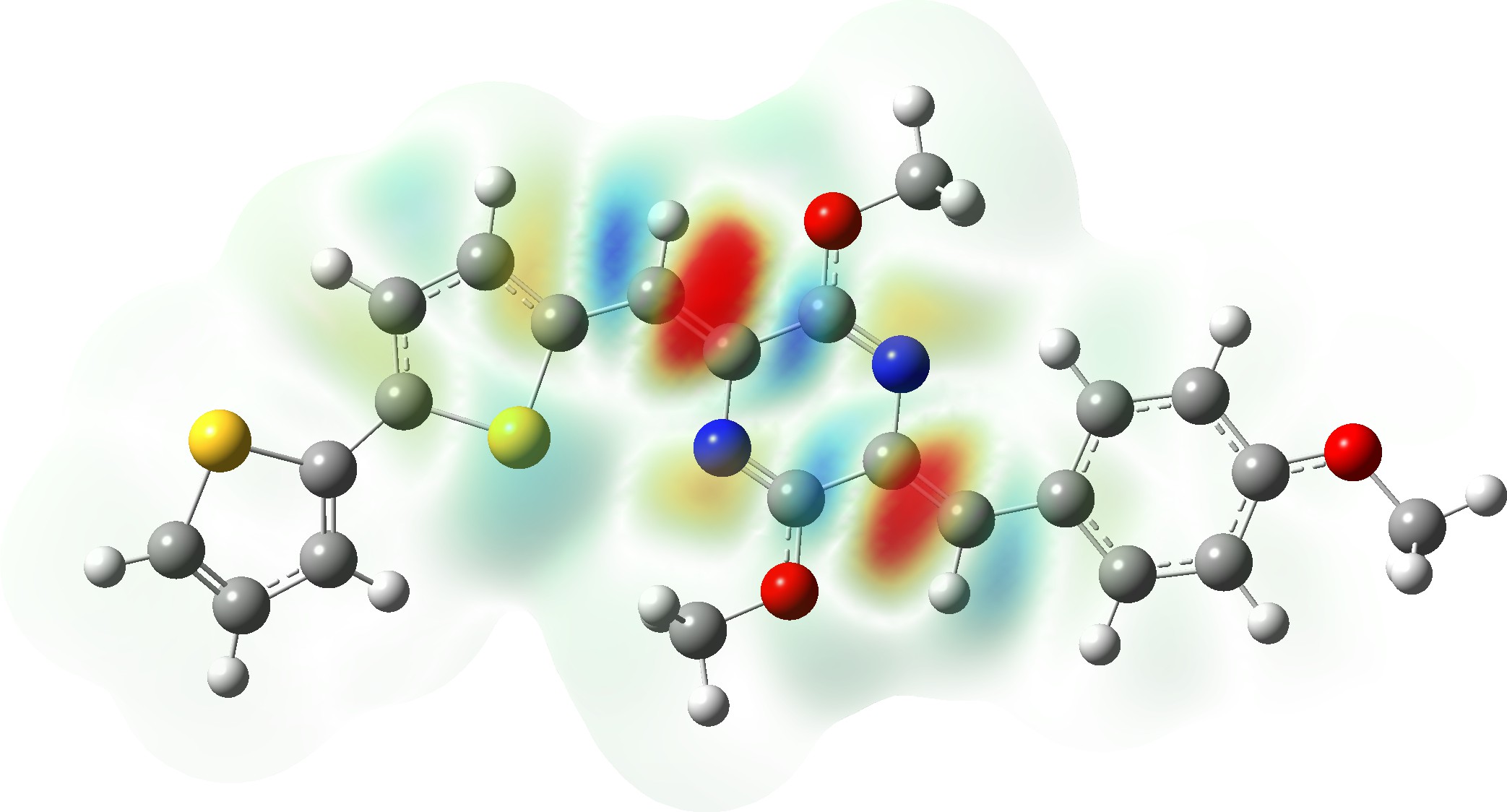


**Figure S37** Effect of the S_0_→S_1_ transition on the electron density of **AsOMe**; increase and decrease of electron densities are represented by blue (+0.0001 and red (-0.0001), respectively.

**Table S9** Absorption wavelengths (λ), oscillator strength (f) and molecular orbitals of the T_1_ → Tn transitions of **AsOMe** in toluene (CPCM) calculated by the wB97XD/6-31G*//wB97XD/6-31G* model, together with the experimental absorption maxima. SOMO and SOMO-1 are the highest and the second highest Singly Occupied Molecular Orbitals in the T_1_ state, respectively.

| **Transition** | **λ_th_/nm** | **f** | **MO** | **%** | **λ_exp_/nm** |
| --- | --- | --- | --- | --- | --- |
| **T_1_**→**T_2_** | 755 | 0.0396 | π_SOMO-2_→π_SOMO-1_ | 59 |  |
| **T_1_**→**T_3_** | 549 | 1.1156 | π_SOMO_→π_SOMO+1_ | 51 | 610 |
| **T_1_**→**T_4_** | 512 | 0.1633 | π_SOMO_→π_SOMO+2_ π_SOMO-3_→π_SOMO-1_ | 30  25 |  |
| **T_1_**→**T_5_** | 418 | 0.2744 | π_SOMO_→π_SOMO+2_ | 28 |  |
| **T_1_**→**T_6_** | 411 | 0.0005 | π_SOMO-7_→π_SOMO-1_ | 86 |  |
| **T_1_**→**T_7_** | 391 | 0.1862 | π_SOMO-3_→π_SOMO-1_ | 23 |  |
| **T_1_**→**T_8_** | 388 | 0.0184 | π_SOMO-4_→π_SOMO-1_ | 16 |  |
| **T_1_**→**T_9_** | 382 | 0.0394 | π_SOMO-4_→π_SOMO-1_ | 14 |  |
| **T_1_**→**T_10_** | 364 | 0.0107 | π_SOMO-1_→π_SOMO+3_ π_SOMO_→π_SOMO+3_ | 21  36 |  |
| **T_1_**→**T_11_** | 340 | 0.2772 | π_SOMO-1_→π_SOMO+1_ π_SOMO_→π_SOMO+5_ | 15  15 |  |

**Table S10** Predicted energies for the **AsOMe** excited states in toluene (CPCM) calculated by TD wB97XD/6- 31G*//wB97XD/6-31G*; the lowest triplet energies were obtained by calculation on the T_1_ state optimised as ground state. ∆ = [E(S_1_)-2E(T_1_)] and Ω = [E(T_2_) - 2E(T_1_)] are the SF driving force and the triplet-triplet annihilation (TTA) possibility, respectively, thus the compounds should match the SF energy criteria (∆ ≥ 0) and TTA criteria (Ω ≥ 0).

| **BF** |  |  |  |  |  |  |  |  |
| --- | --- | --- | --- | --- | --- | --- | --- | --- |
| **Transition** | **λ/nm** | **ΔE/eV** | **E(wB97XD)/**  **Ha** | **ΔE(wB97XD)**  **/Ha** | **ΔE(wB97XD)/**  **eV** | **λ/ nm** | **State** | **E/eV** |
| S0 |  |  | -2019.877600 | 0.000000 | 0.000 |  | S0 | 0.000 |
| S0←T1,rel |  |  | -2019.836500 | 0.041100 | 1.118 | 1109 |  |  |
| S0←S1,rel |  |  | -2019.788700 | 0.088900 | 2.419 | 513 | T1,rel | **1.118** |
| S0→T1,FC | 982 | 1.263 |  |  |  |  | T1,FC | 1.263 |
| S1,rel→S0 | 590 | 2.103 |  |  |  |  | S1,rel | **2.103** |
| S0→S1,FC | 444 | 2.794 |  |  |  |  | S1,FC | 2.794 |
| S0→T2,FC | 515 | 2.409 |  |  |  |  | T2,FC | 2.409 |
| T1,rel→T2 | 755 | 1.643 |  |  |  |  | T2@T1,rel | **2.762** |
| ∆ | 0.558 |  |  |  |  |  |  |  |
| ∆adiab | -0.134 |  |  |  |  |  |  |  |
| ∆FC | 0.267 |  |  |  |  |  |  |  |
| Ω | 0.525 |  |  |  |  |  |  |  |

## **4.2 AsNMe_2_**

**
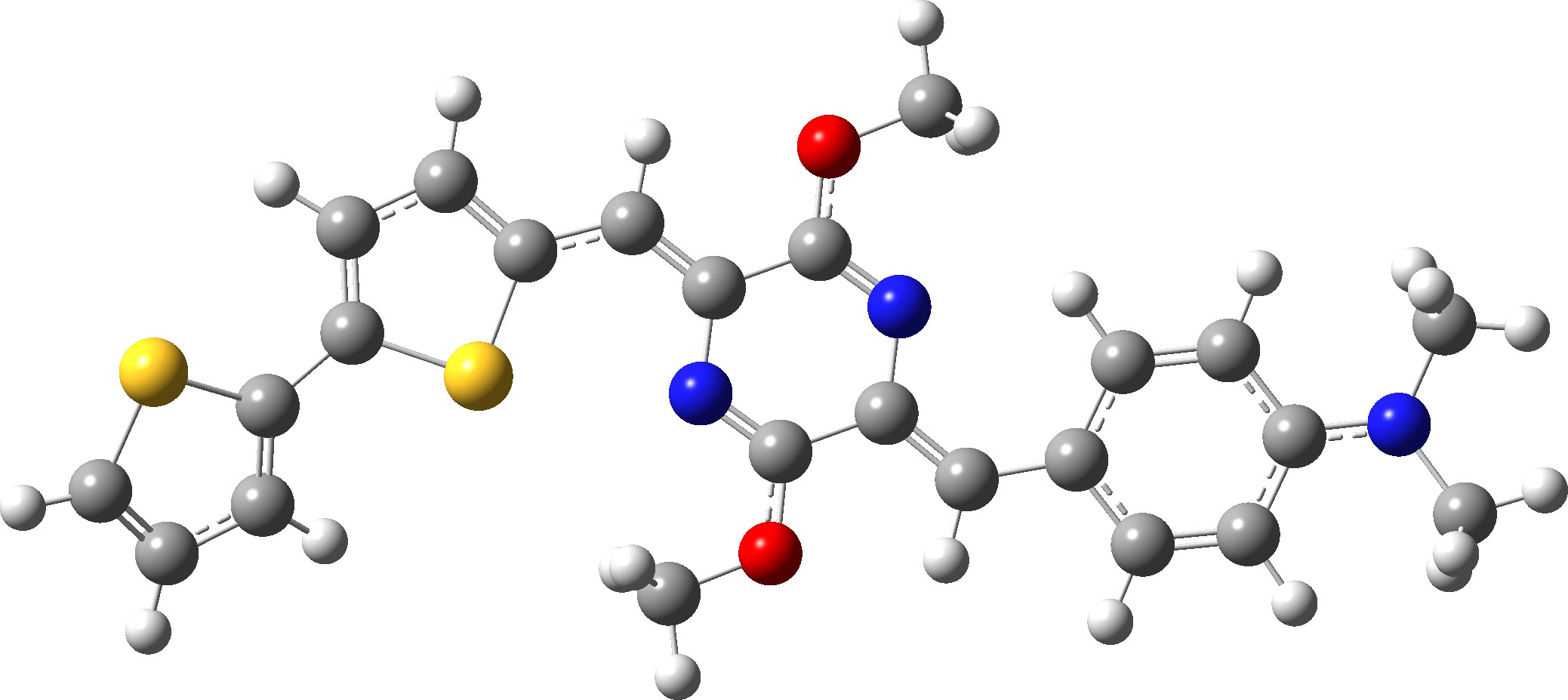
**

**Figure S38** Ground state optimized geometry of **AsNMe_2_** by wB97XD/6-311+G(d) @ S_0_ in toluene.

**Table S11** Absorption wavelengths (λ), oscillator strength (f) and molecular orbitals of **AsNMe_2_** in toluene (CPCM) calculated by the wB97XD/6-311+G(d)//wB97XD/6-311+G(d) model, together with the experimental absorption and emission maxima.

| **Transition** | **λth/nm** | **f** | **MO** | **%** | **λexp/nm** |
| --- | --- | --- | --- | --- | --- |
| **S0**→**T1** | 1008 | 0.0000 | πH→πL* | 87 |  |
| **S0**→**T2** | 526 | 0.0000 | πH-1→πL* | 42 |  |
| **S0**→**S1** | 461 | 2.1457 | πH→πL* | 91 | 504 |
| **S0**→**T3** | 410 | 0.0000 | πH-1→πL+1* | 27 |  |
| **S0**→**T4** | 343 | 0.0000 | πH-1→πL+2* | 13 |  |
| **S0**→**S2** | 337 | 0.0639 | πH-1→πL* | 70 |  |
| **S0**→**T5** | 332 | 0.0000 | πH→πL+2* | 26 |  |
| **S0**→**T6** | 323 | 0.0000 | πH→πL+4* | 46 |  |
| **S0**→**T7** | 311 | 0.0000 | πH-3→πL* | 28 |  |
| **S0**→**T8** | 306 | 0.0000 | πH→πL+10* | 12 |  |
| **S0**→**T9** | 303 | 0.0000 | nH-7→πL* | 84 |  |
| **S0**→**S3** | 291 | 0.0006 | nH-7→πL* | 84 |  |
| **S0**→**T10** | 288 | 0.0000 | πH-5→πL+4* | 31 |  |
| **S0**→**S4** | 285 | 0.1051 | πH→πL+1* | 50 |  |
| **S0**→**S5** | 279 | 0.0462 | πH→πL+4* | 43 |  |
| **S0**→**S6** | 269 | 0.1583 | πH→πL+2* | 31 |  |
| **S0**→**S7** | 258 | 0.1923 | πH-3→πL* | 52 |  |
| **S0**→**S8** | 253 | 0.0340 | πH-2→πL* | 27 |  |
| **S0**→**S9** | 249 | 0.0615 | πH-4→πL* | 25 |  |
| **S0**→**S10** | 240 | 0.0174 | πH→πL+3* | 47 |  |
| **S1**→**S0** | 612 | 2.1300 | πH→πL* | 95 | 569 |


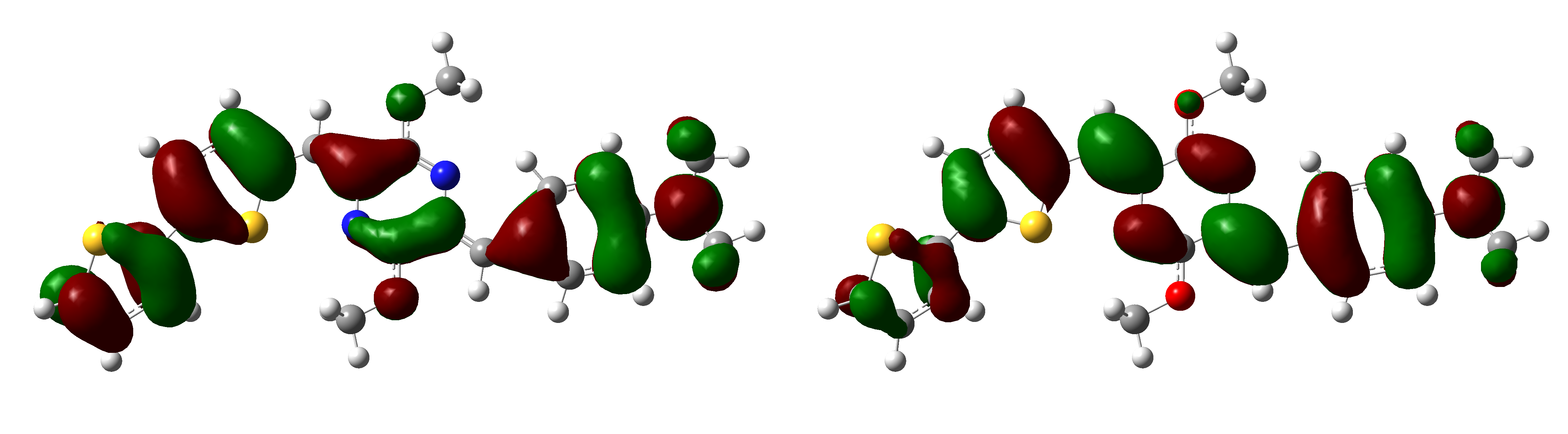


**πH-1 πH**

**
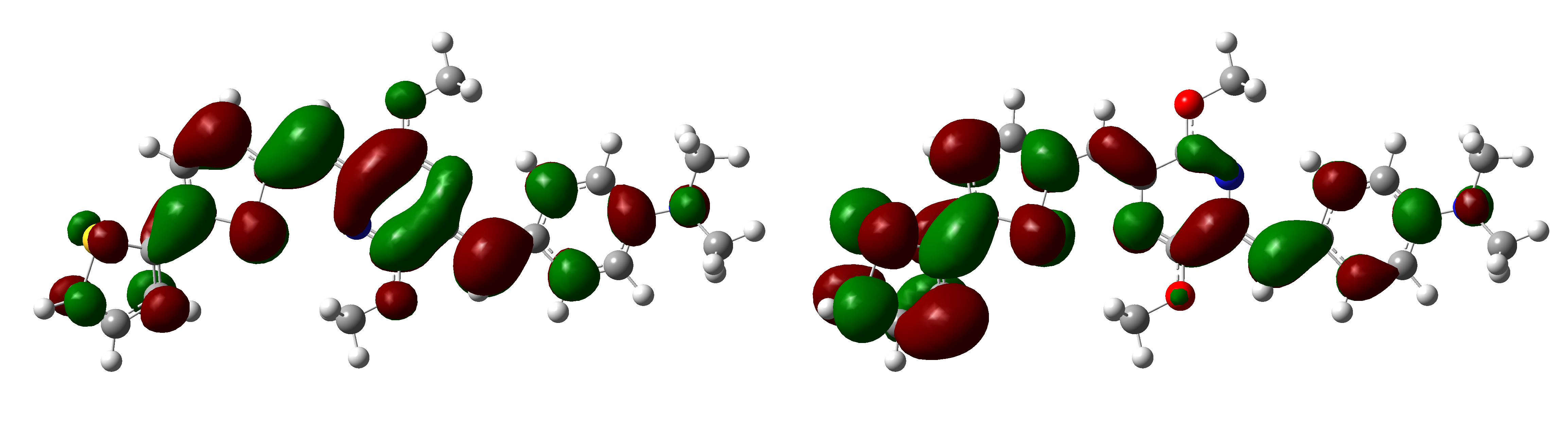
**

**πL πL+1**

**Figure S39** Frontier molecular orbitals of **AsNMe_2_**.


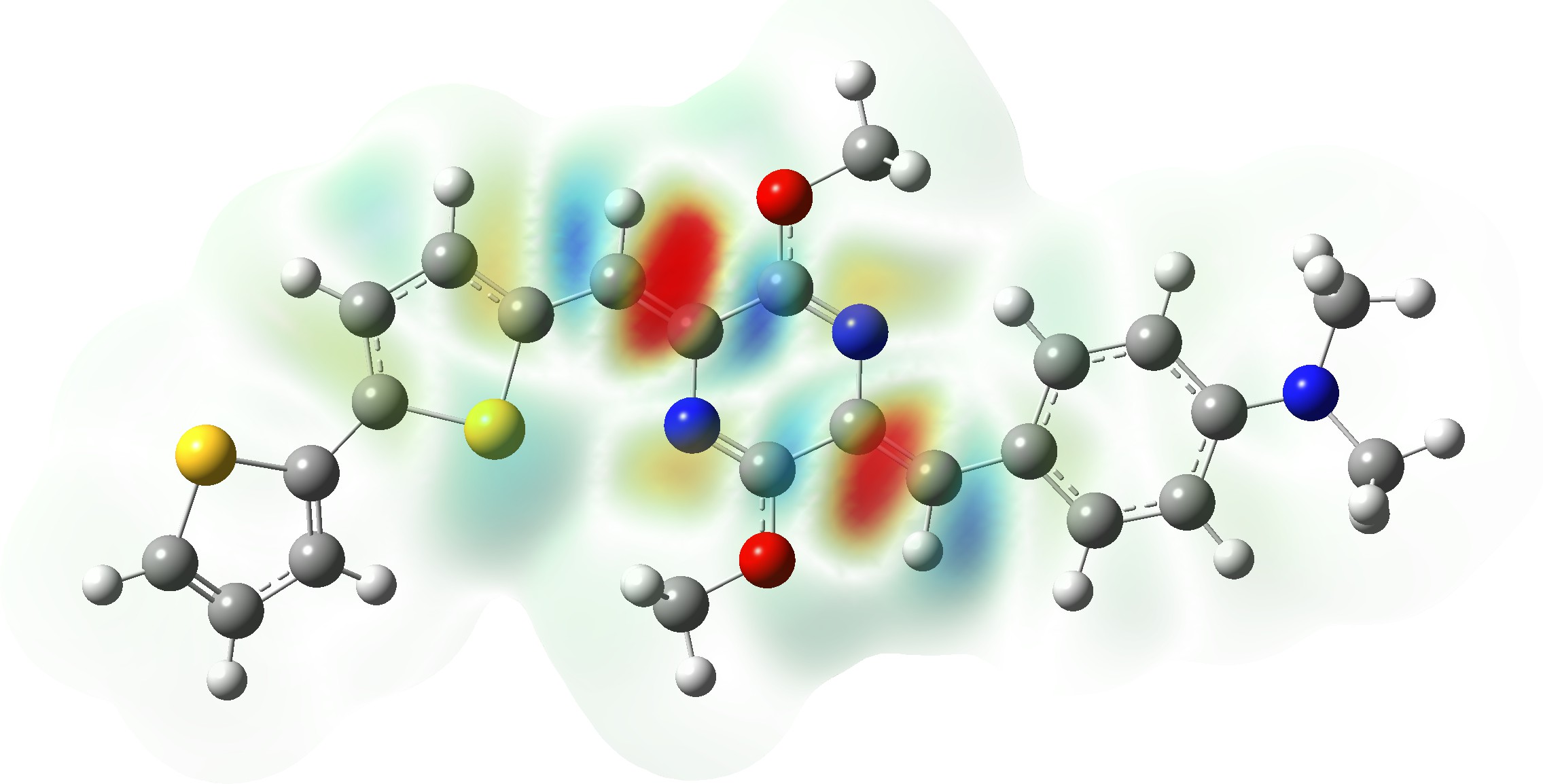


**Figure S40** Effect of the S_0_→S_1_ transition on the electron density of **AsNMe_2_**; increase and decrease of electron densities are represented by blue (+0.0001) and red (-0.0001), respectively.

**Table S12** Absorption wavelengths (λ), oscillator strength (f) and molecular orbitals of the T_1_ → Tn transitions of **AsNMe_2_** in toluene (CPCM) calculated by the wB97XD/6-31G*//wB97XD/6-31G* model, together with the experimental absorption maxima. SOMO and SOMO-1 are the highest and the second highest Singly Occupied Molecular Orbitals in the T_1_ state, respectively.

| **Transition** | **λth/nm** | **f** | **MO** | **%** | **λexp/nm** |
| --- | --- | --- | --- | --- | --- |
| **T1**→**T2** | 771 | 0.0796 | πSOMO-2→πSOMO-1 | 56 |  |
| **T1**→**T3** | 571 | 1.2856 | πSOMO→πSOMO+1 | 51 | 640 |
| **T1**→**T4** | 517 | 0.1494 | πSOMO→πSOMO+2 | 33 |  |
| **T1**→**T5** | 438 | 0.0203 | πSOMO-3→πSOMO-1 | 31 |  |
| **T1**→**T6** | 416 | 0.3957 | πSOMO-4→πSOMO-1 | 27 |  |
| **T1**→**T7** | 404 | 0.0005 | πSOMO-8→πSOMO-1 | 86 |  |
| **T1**→**T8** | 389 | 0.0325 | πSOMO→πSOMO+3 nSOMO-7→πSOMO-1 πSOMO-5→πSOMO-1 | 13  11  12 |  |
| **T1**→**T9** | 378 | 0.0359 | πSOMO→πSOMO+3 | 36 |  |
| **T1**→**T10** | 376 | 0.0740 | πSOMO-5→πSOMO-1 | 18 |  |
| **T1**→**T11** | 355 | 0.1500 | πSOMO-1→πSOMO+1 | 18 |  |

**Table S13** Predicted energies for the **AsNMe_2_** excited states in toluene (CPCM) calculated by TD wB97XD/6- 31G*//wB97XD/6-31G*; the lowest triplet energies were obtained by calculation on the T_1_ state optimised as ground state. ∆ = [E(S_1_)-2E(T_1_)] and Ω = [E(T_2_) - 2E(T_1_)] are the SF driving force and the triplet-triplet annihilation (TTA) possibility, respectively, thus the compounds should match the SF energy criteria (∆ ≥ 0) and TTA criteria (Ω ≥ 0).

| **BF** |  |  |  |  |  |  |  |  |
| --- | --- | --- | --- | --- | --- | --- | --- | --- |
| **Transition** | **λ/nm** | **ΔE/eV** | **E(wB97XD)/**  **Ha** | **ΔE(wB97XD)**  **/Ha** | **ΔE(wB97XD)/**  **eV** | **λ/ nm** | **State** | **E/eV** |
| S0 |  |  | -2039.317700 | 0.000000 | 0.000 |  | S0 | 0.000 |
| S0←T1,rel |  |  | -2039.277400 | 0.040300 | 1.097 | 1131 |  |  |
| S0←S1,rel |  |  | -2039.231800 | 0.085900 | 2.337 | 531 | T1,rel | **1.097** |
| S0→T1,FC | 1008 | 1.231 |  |  |  |  | T1,FC | 1.231 |
| S1,rel→S0 | 612 | 2.027 |  |  |  |  | S1,rel | **2.027** |
| S0→S1,FC | 461 | 2.691 |  |  |  |  | S1,FC | 2.691 |
| S0→T2,FC | 526 | 2.359 |  |  |  |  | T2,FC | 2.359 |
| T1,rel→T2 | 771 | 1.609 |  |  |  |  | T2@T1,rel | **2.706** |
| ∆ | 0.498 |  |  |  |  |  |  |  |
| ∆adiab | -0.166 |  |  |  |  |  |  |  |
| ∆FC | 0.230 |  |  |  |  |  |  |  |
| Ω | 0.513 |  |  |  |  |  |  |  |

## **4.3 TPh**

**
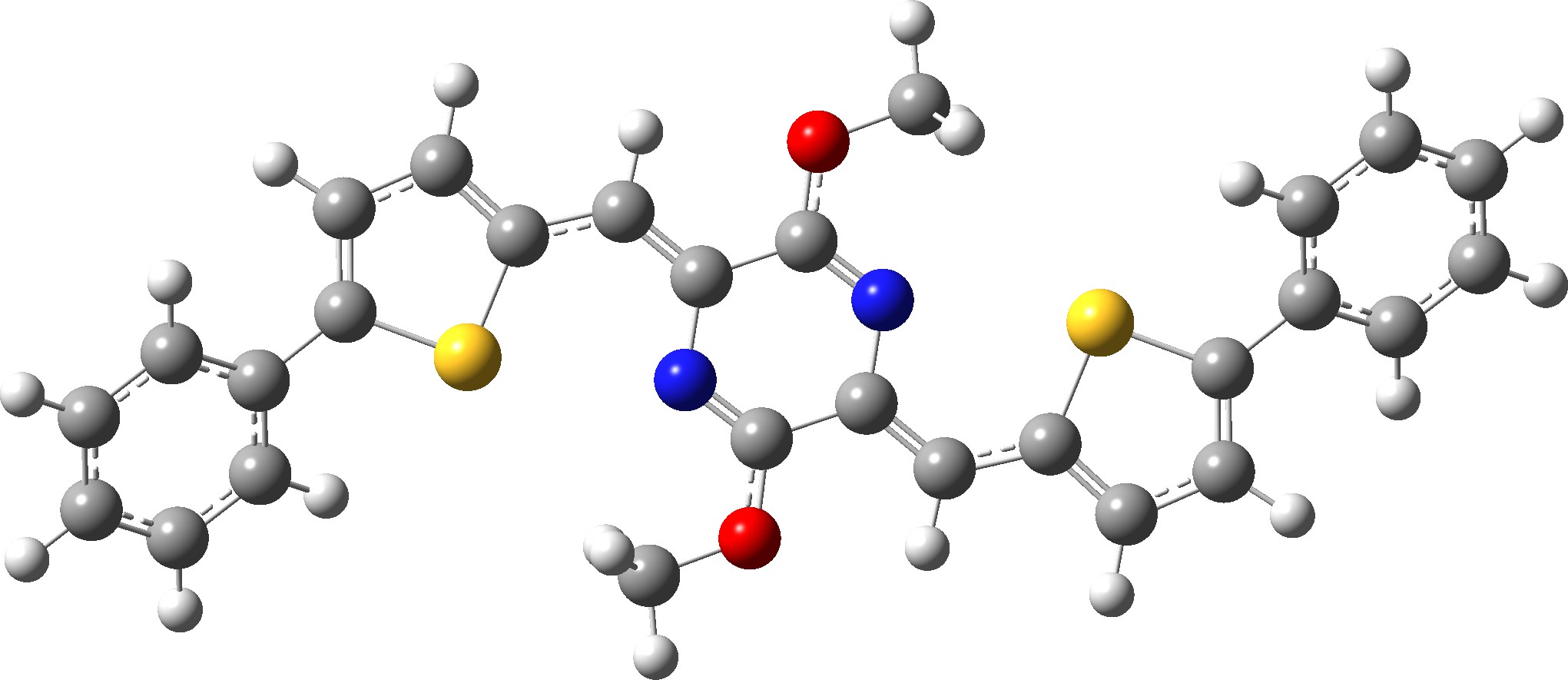
**

**Figure S41** Ground state optimized geometry of **TPh** by wB97XD/6-311+G(d) @ S_0_ in toluene.

**Table S14** Absorption wavelengths (λ), oscillator strength (f) and molecular orbitals of **TPh** in toluene (CPCM) calculated by the wB97XD/6-311+G(d)//wB97XD/6-311+G(d) model, together with the experimental absorption and emission maxima.

| **Transition** | **λth/nm** | **f** | **MO** | **%** | **λexp/nm** |
| --- | --- | --- | --- | --- | --- |
| **S0**→**T1** | 1087 | 0.0000 | πH→πL* | 96 |  |
| **S0**→**T2** | 544 | 0.0000 | πH-1→πL* πH→πL+1* | 49  33 |  |
| **S0**→**S1** | 464 | 2.0822 | πH→πL* | 93 | 503 |
| **S0**→**T3** | 401 | 0.0000 | πH-2→πL* πH-1→πL+1* πH→πL+2* | 21  27  23 |  |
| **S0**→**T4** | 362 | 0.0000 | πH-1→πL+2* | 20 |  |
| **S0**→**S2** | 335 | 0.0000 | πH-1→πL* | 70 |  |
| **S0**→**T5** | 330 | 0.0000 | πH→πL+6* | 28 |  |
| **S0**→**T6** | 320 | 0.0000 | πH-10→πL* πH-7→πL+4* | 12  12 |  |
| **S0**→**T7** | 314 | 0.0000 | nH-3→πL* | 52 |  |
| **S0**→**T8** | 314 | 0.0000 | πH-4→πL* | 56 |  |
| **S0**→**T9** | 304 | 0.0000 | nH-8→πL* | 75 |  |
| **S0**→**T10** | 301 | 0.0000 | πH-7→πL+3* πH→πL+6* | 10  10 |  |
| **S0**→**S3** | 293 | 0.0000 | nH-8→πL* | 74 |  |
| **S0**→**S4** | 285 | 0.0000 | πH→πL+1* | 60 |  |
| **S0**→**S5** | 267 | 0.2520 | πH-4→πL+2* πH→πL+2* | 33  28 |  |
| **S0**→**S6** | 265 | 0.0000 | πH-3→πL* | 59 |  |
| **S0**→**S7** | 258 | 0.7077 | πH-4→πL* | 38 |  |
| **S0**→**S8** | 256 | 0.0000 | πH→πL+6* | 37 |  |
| **S0**→**S9** | 249 | 0.0021 | πH→πL+4* | 28 |  |
| **S0**→**S10** | 249 | 0.0000 | πH→πL+3* | 25 |  |
| **S1**→**S0** | 625 | 2.1220 | πH→πL* | 95 | 558 |


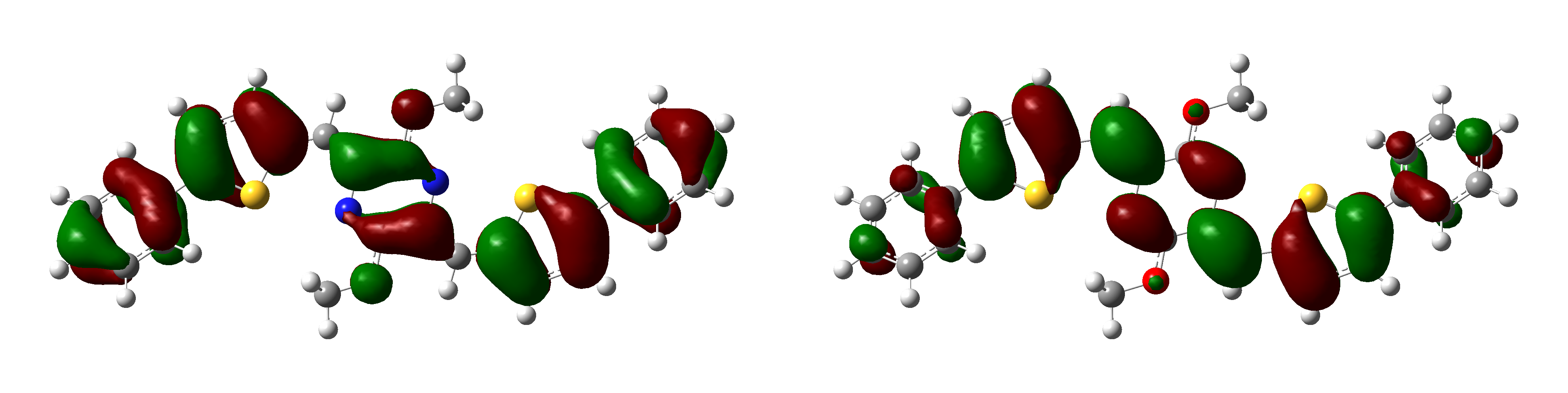


**πH-1 πH**

**
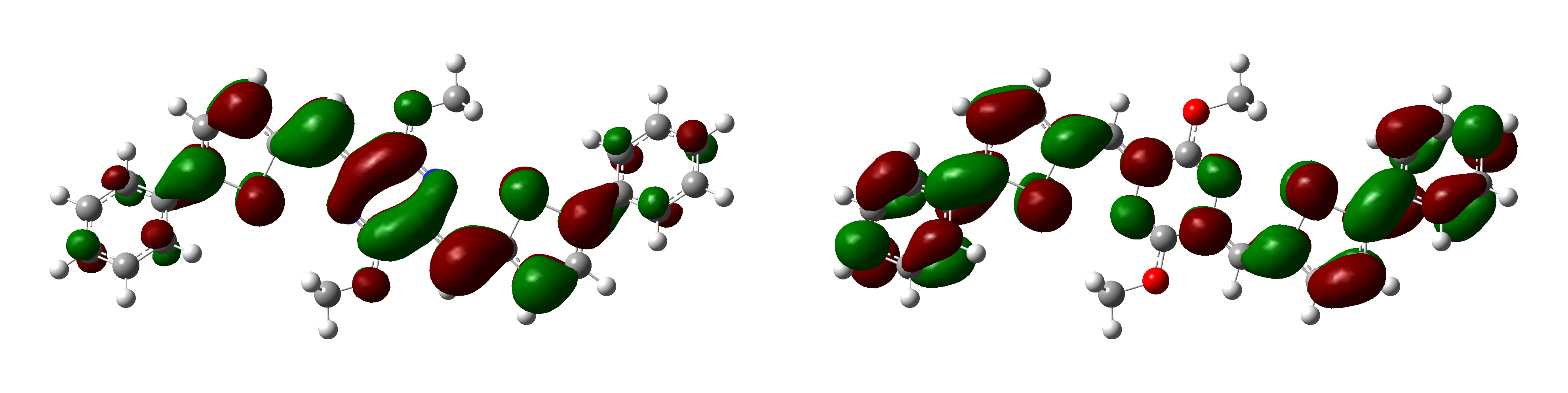
**

**πL πL+**

**Figure S42** Frontier molecular orbitals of **TPh**.


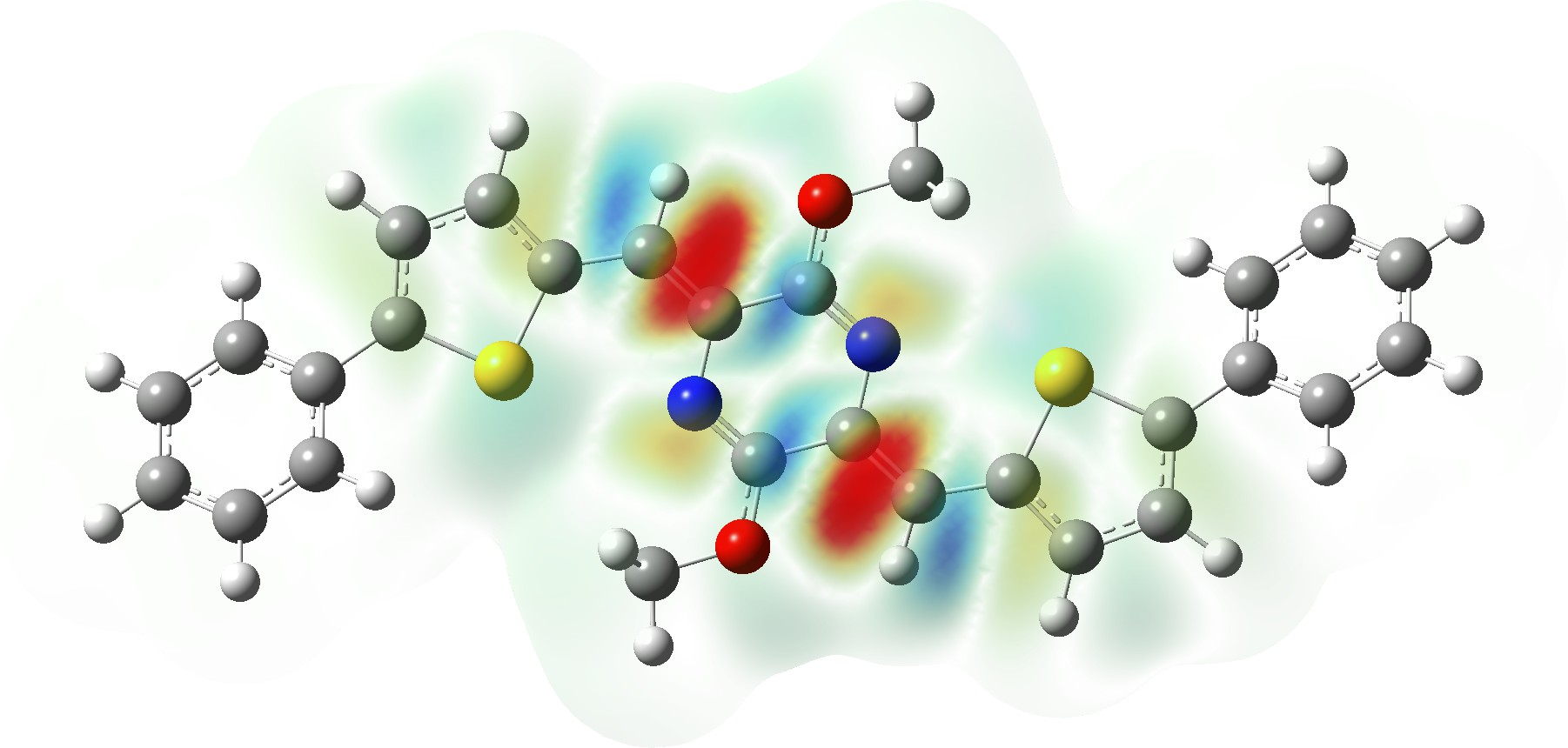


**Figure S43** Effect of the S_0_→S_1_ transition on the electron density of **TPh**; increase and decrease of electron densities are represented by blue (+0.0001 and red (-0.0001), respectively.

**Table S15** Absorption wavelengths (λ), oscillator strength (f) and molecular orbitals of the T_1_ → Tn transitions of **TPh** in toluene (CPCM) calculated by the wB97XD/6-31G*//wB97XD/6-31G* model, together with the experimental absorption maxima. SOMO and SOMO-1 are the highest and the second highest Singly Occupied Molecular Orbitals in the T_1_ state, respectively.

| **Transition** | **λth/nm** | **f** | **MO** | **%** | **λexp/nm** |
| --- | --- | --- | --- | --- | --- |
| **T1**→**T2** | 763 | 0.0528 | πSOMO-2→πSOMO-1 | 61 |  |
| **T1**→**T3** | 550 | 1.3527 | πSOMO→πSOMO+1 | 58 | 620 |
| **T1**→**T4** | 491 | 0.0000 | πSOMO-3→πSOMO-1 | 27 |  |
| **T1**→**T5** | 444 | 0.0145 | πSOMO→πSOMO+3 | 29 |  |
| **T1**→**T6** | 399 | 0.0636 | πSOMO-9→πSOMO-1 | 69 |  |
| **T1**→**T7** | 394 | 0.3853 | πSOMO-9→πSOMO-1 | 16 |  |
| **T1**→**T8** | 393 | 0.0000 | πSOMO-6→πSOMO-1 | 43 |  |
| **T1**→**T9** | 385 | 0.2061 | πSOMO-5→πSOMO-1 | 46 |  |
| **T1**→**T10** | 364 | 0.0000 | πSOMO-6→πSOMO-1 | 18 |  |
| **T1**→**T11** | 354 | 0.0617 | πSOMO-10→πSOMO-1 | 15 |  |

**Table S16** Predicted energies for the **TPh** excited states in toluene (CPCM) calculated by TD wB97XD/6-31G*//wB97XD/6-31G*; the lowest triplet energies were obtained by calculation on the T_1_ state optimised as ground state. ∆ = [E(S_1_)-2E(T_1_)] and Ω = [E(T_2_) - 2E(T_1_)] are the SF driving force and the triplet-triplet annihilation (TTA) possibility, respectively, thus the compounds should match the SF energy criteria (∆ ≥ 0) and TTA criteria (Ω ≥ 0).

| **BF** |  |  |  |  |  |  |  |  |
| --- | --- | --- | --- | --- | --- | --- | --- | --- |
| **Transition** | **λ/nm** | **ΔE/eV** | **E(wB97XD)/**  **Ha** | **ΔE(wB97XD)**  **/Ha** | **ΔE(wB97XD)/**  **eV** | **λ/ nm** | **State** | **E/eV** |
| S0 |  |  | -2136.388000 | 0.000000 | 0.000 |  | S0 | 0.000 |
| S0←T1,rel |  |  | -2136.350300 | 0.037700 | 1.026 | 1209 |  |  |
| S0←S1,rel |  |  | -2136.302400 | 0.085600 | 2.329 | 533 | T1,rel | **1.026** |
| S0→T1,FC | 1087 | 1.141 |  |  |  |  | T1,FC | 1.141 |
| S1,rel→S0 | 625 | 1.985 |  |  |  |  | S1,rel | **1.985** |
| S0→S1,FC | 464 | 2.674 |  |  |  |  | S1,FC | 2.674 |
| S0→T2,FC | 544 | 2.281 |  |  |  |  | T2,FC | 2.281 |
| T1,rel→T2 | 763 | 1.626 |  |  |  |  | T2@T1,rel | **2.652** |
| ∆ | 0.622 |  |  |  |  |  |  |  |
| ∆adiab | -0.067 |  |  |  |  |  |  |  |
| ∆FC | 0.391 |  |  |  |  |  |  |  |
| Ω | 0.600 |  |  |  |  |  |  |  |

## **4.4 TPhOMe**

**
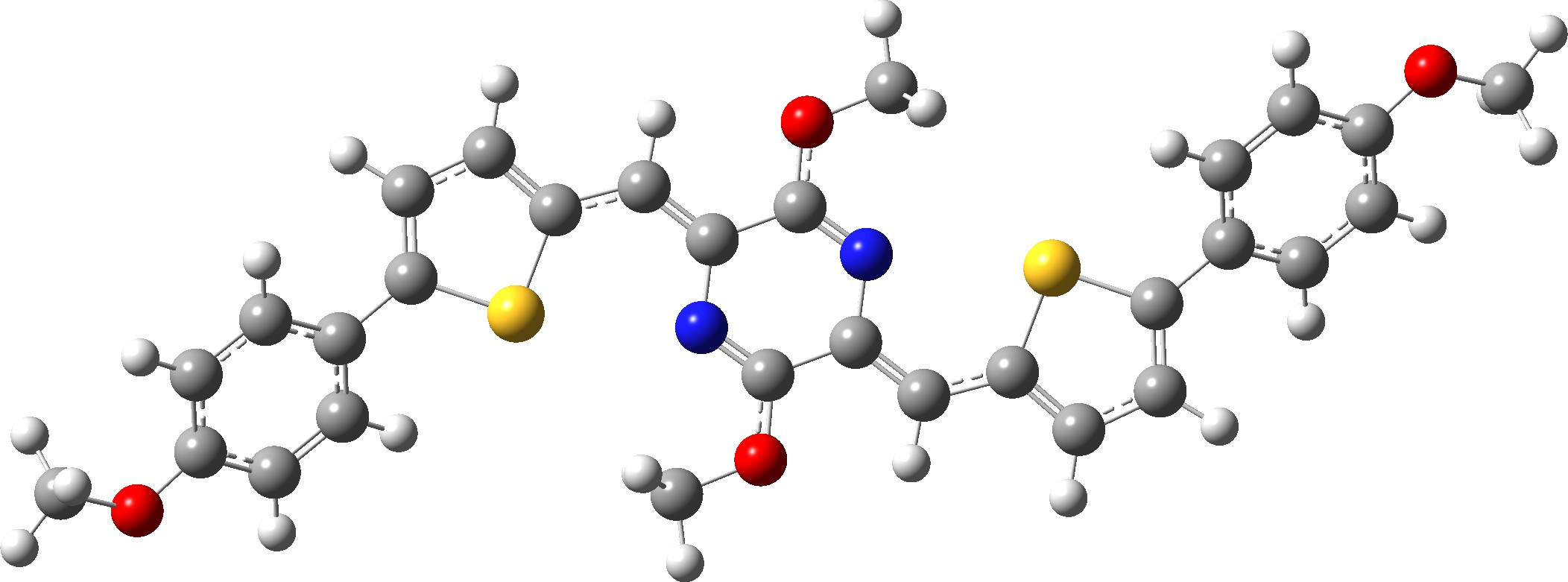
**

**Figure S44** Optimized geometry of **TPhOMe** by wB97XD/6-311+G(d) @ S_0_ in toluene.

**Table S17** Absorption wavelengths (λ), oscillator strength (f) and molecular orbitals of **TPhOMe** in toluene (CPCM) calculated by the wB97XD/6-311+G(d)//wB97XD/6-311+G(d) model, together with the experimental absorption and emission maxima.

| **Transition** | **λth/nm** | **f** | **MO** | **%** | **λexp/nm** |
| --- | --- | --- | --- | --- | --- |
| **S0**→**T1** | 1088 | 0.0000 | πH→πL* | 94 |  |
| **S0**→**T2** | 543 | 0.0000 | πH-1→πL* πH→πL+1* | 42  34 |  |
| **S0**→**S1** | 466 | 2.1807 | πH→πL* | 92 | 514 |
| **S0**→**T3** | 402 | 0.0000 | πH-1→πL+1* | 27 |  |
| **S0**→**T4** | 365 | 0.0000 | πH-3→πL* | 21 |  |
| **S0**→**S2** | 338 | 0.0000 | πH-1→πL* | 68 |  |
| **S0**→**T5** | 332 | 0.0000 | πH→πL+1* | 17 |  |
| **S0**→**T6** | 325 | 0.0000 | πH-10→πL* πH-2→πL+2* | 14  12 |  |
| **S0**→**T7** | 311 | 0.0000 | πH-4→πL* | 60 |  |
| **S0**→**T8** | 311 | 0.0000 | πH-5→πL* | 62 |  |
| **S0**→**T9** | 307 | 0.0000 | πH-2→πL+3* πH-1→πL+4* | 20  18 |  |
| **S0**→**T10** | 305 | 0.0000 | πH-1→πL+3* | 30 |  |
| **S0**→**S3** | 291 | 0.0000 | nH-8→πL* | 81 |  |
| **S0**→**S4** | 284 | 0.0000 | πH→πL+1* | 62 |  |
| **S0**→**S5** | 270 | 0.5803 | πH-2→πL* | 29 |  |
| **S0**→**S6** | 264 | 0.0000 | πH-4→πL* | 41 |  |
| **S0**→**S7** | 261 | 0.0000 | πH→πL+3* | 27 |  |
| **S0**→**S8** | 261 | 0.0691 | πH-1→πL+3* | 31 |  |
| **S0**→**S9** | 259 | 0.3835 | πH-5→πL* | 63 |  |
| **S0**→**S10** | 258 | 0.0000 | πH-3→πL* | 27 |  |
| **S1**→**S0** | 631 | 2.2447 | πH→πL* | 95 | 574 |


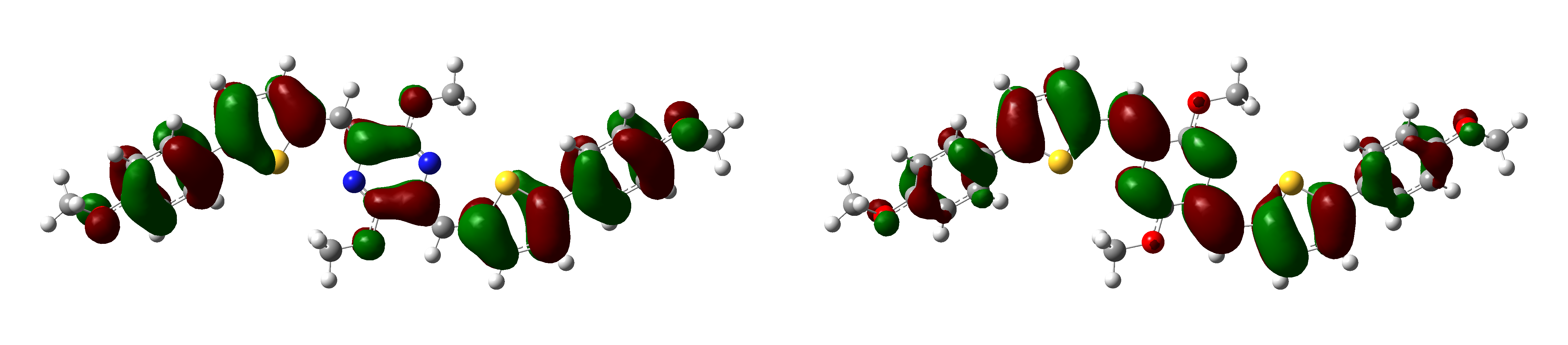


**πH-1 πH**

**
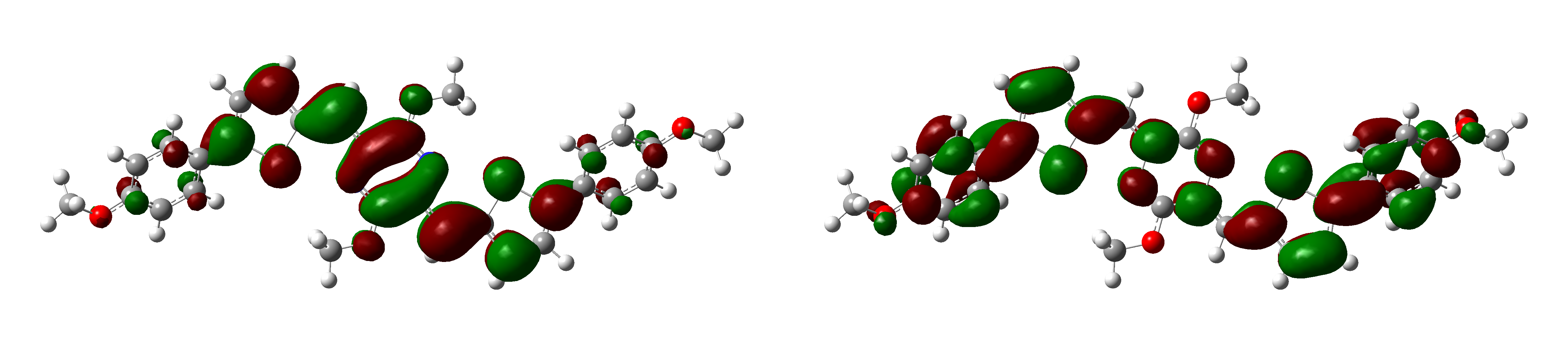
**

**πL πL+1**

**Figure S45** Frontier molecular orbitals of **TPhOMe**.


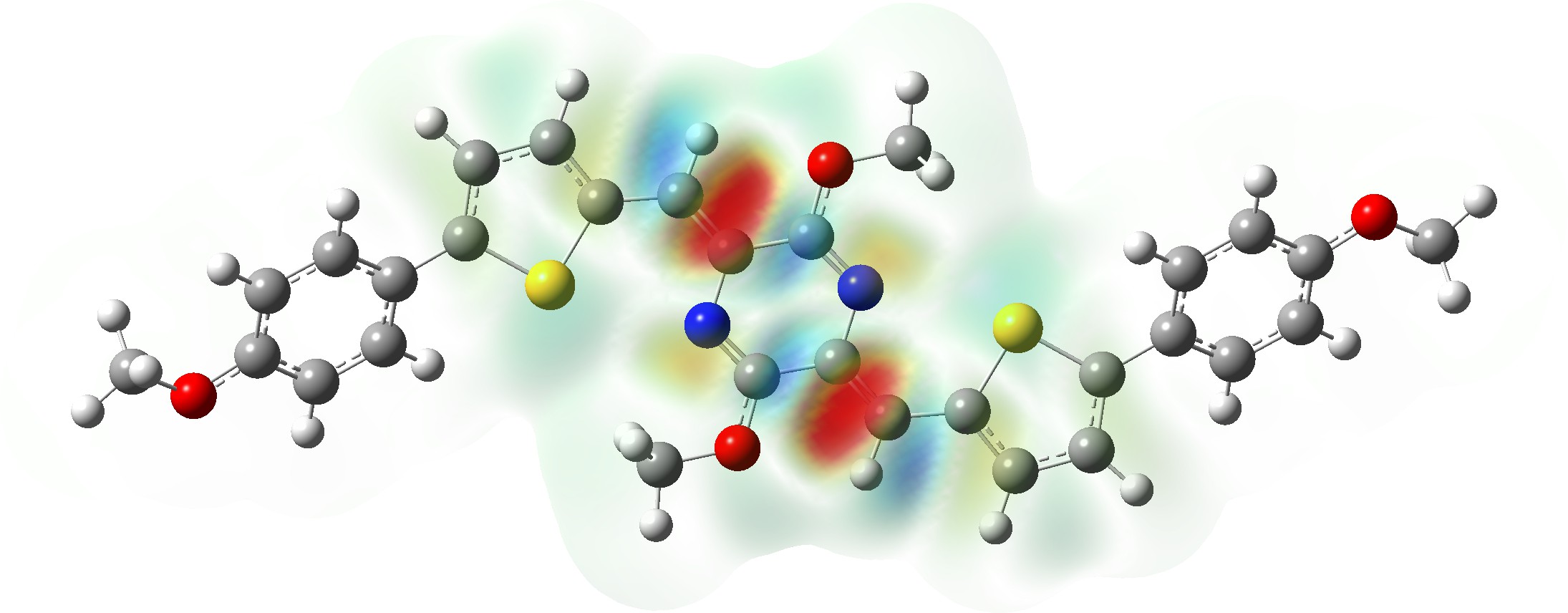


**Figure S46** Effect of the S_0_→S_1_ transition on the electron density of **TPhOMe**; increase and decrease of electron densities are represented by blue (+0.0001) and red (-0.0001), respectively.

**Table S18** Absorption wavelengths (λ), oscillator strength (f) and molecular orbitals of the T_1_ → Tn transitions of **TPhOMe** in toluene (CPCM) calculated by the wB97XD/6-31G*//wB97XD/6-31G* model, together with the experimental absorption maxima. SOMO and SOMO-1 are the highest and the second highest Singly Occupied Molecular Orbitals in the T_1_ state, respectively.

| **Transition** | **λth/nm** | **f** | **MO** | **%** | **λexp/nm** |
| --- | --- | --- | --- | --- | --- |
| **T1**→**T2** | 767 | 0.0703 | πSOMO-2→πSOMO-1 | 58 |  |
| **T1**→**T3** | 555 | 1.4542 | πSOMO→πSOMO+1 | 59 | 640 |
| **T1**→**T4** | 498 | 0.0000 | πSOMO-3→πSOMO-1 | 27 |  |
| **T1**→**T5** | 449 | 0.0078 | πSOMO-4→πSOMO-1 | 20 |  |
| **T1**→**T6** | 400 | 0.5791 | πSOMO→πSOMO+3 πSOMO-4→πSOMO-1 | 16  16 |  |
| **T1**→**T7** | 393 | 0.0538 | nSOMO-9→πSOMO-1 | 76 |  |
| **T1**→**T8** | 389 | 0.0000 | πSOMO-5→πSOMO-1 | 42 |  |
| **T1**→**T9** | 382 | 0.0433 | πSOMO-6→πSOMO-1 | 62 |  |
| **T1**→**T10** | 370 | 0.0000 | πSOMO-5→πSOMO-1 | 22 |  |
| **T1**→**T11** | 359 | 0.0761 | πSOMO-10→πSOMO-1 | 19 |  |

**Table S19** Predicted energies for the **TPhOMe** excited states in toluene (CPCM) calculated by TD wB97XD/ 6-31G*//wB97XD/6-31G*; the lowest triplet energies were obtained by calculation on the T_1_ state optimised as ground state. ∆ = [E(S_1_)-2E(T_1_)] and Ω = [E(T_2_) - 2E(T_1_)] are the SF driving force and the triplet-triplet annihilation (TTA) possibility, respectively, thus the compounds should match the SF energy criteria (∆ ≥ 0) and TTA criteria (Ω ≥ 0).

| **BF** |  |  |  |  |  |  |  |  |
| --- | --- | --- | --- | --- | --- | --- | --- | --- |
| **Transition** | **λ/nm** | **ΔE/eV** | **E(wB97XD)/**  **Ha** | **ΔE(wB97XD)**  **/Ha** | **ΔE(wB97XD)/**  **eV** | **λ/ nm** | **State** | **E/eV** |
| S0 |  |  | -2365.427723 | 0.000000 | 0.000 |  | S0 | 0.000 |
| S0←T1,rel |  |  | -2365.389994 | 0.037729 | 1.027 | 1208 |  |  |
| S0←S1,rel |  |  | -2365.343041 | 0.084682 | 2.304 | 538 | T1,rel | **1.027** |
| S0→T1,FC | 1088 | 1.140 |  |  |  |  | T1,FC | 1.140 |
| S1,rel→S0 | 631 | 1.966 |  |  |  |  | S1,rel | **1.966** |
| S0→S1,FC | 466 | 2.662 |  |  |  |  | S1,FC | 2.662 |
| S0→T2,FC | 543 | 2.285 |  |  |  |  | T2,FC | 2.285 |
| T1,rel→T2 | 767 | 1.618 |  |  |  |  | T2@T1,rel | **2.644** |
| ∆ | 0.609 |  |  |  |  |  |  |  |
| ∆adiab | -0.087 |  |  |  |  |  |  |  |
| ∆FC | 0.382 |  |  |  |  |  |  |  |
| Ω | 0.591 |  |  |  |  |  |  |  |


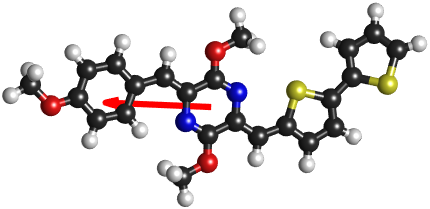


**Figure S47** Representation of the orientation of the S_0_→S_1_ transition dipole moment of **AsOMe** in the ground state optimized geometry.


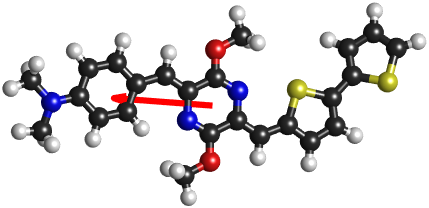


**Figure S48** Representation of the orientation of the S_0_→S_1_ transition dipole moment of **AsNMe_2_** in the ground state optimized geometry.


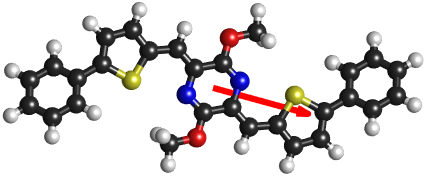


**Figure S49** Representation of the orientation of the S_0_→S_1_ transition dipole moment of **TPh** in the ground state optimized geometry.


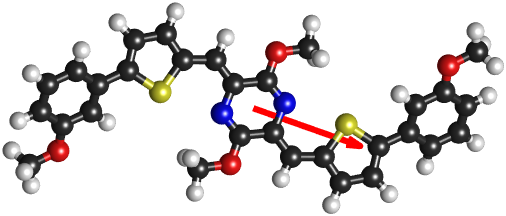


**Figure S50** Representation of the orientation of the S_0_→S_1_ transition dipole moment of **TPhOMe** in the ground state optimized geometry.

**
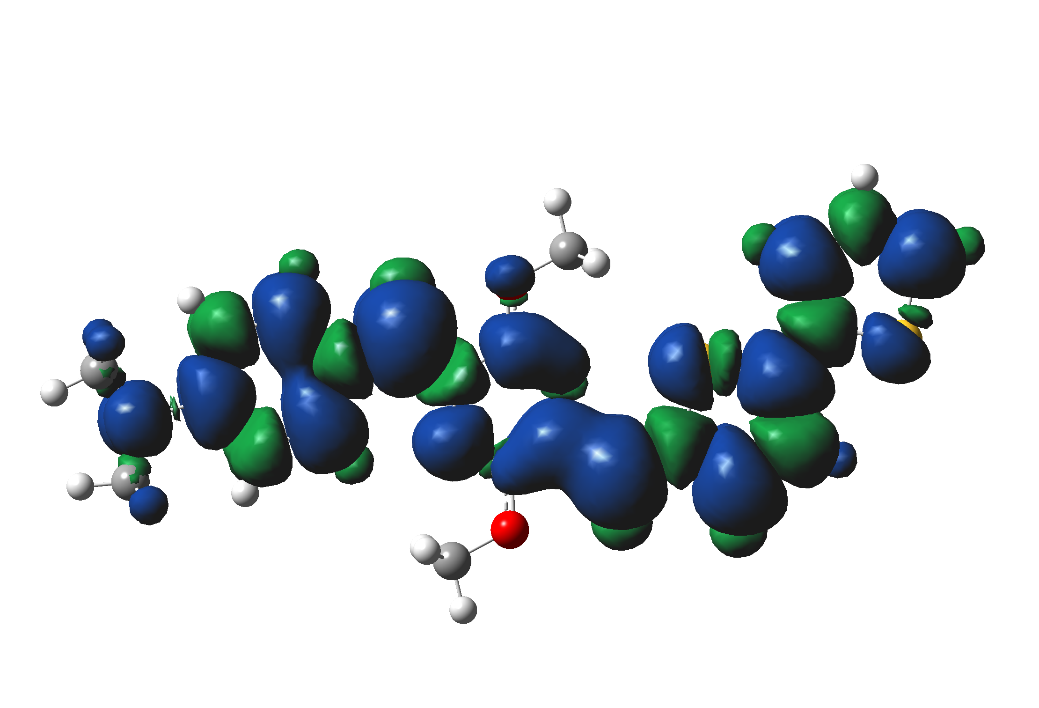
**

**AsNMe2**

**
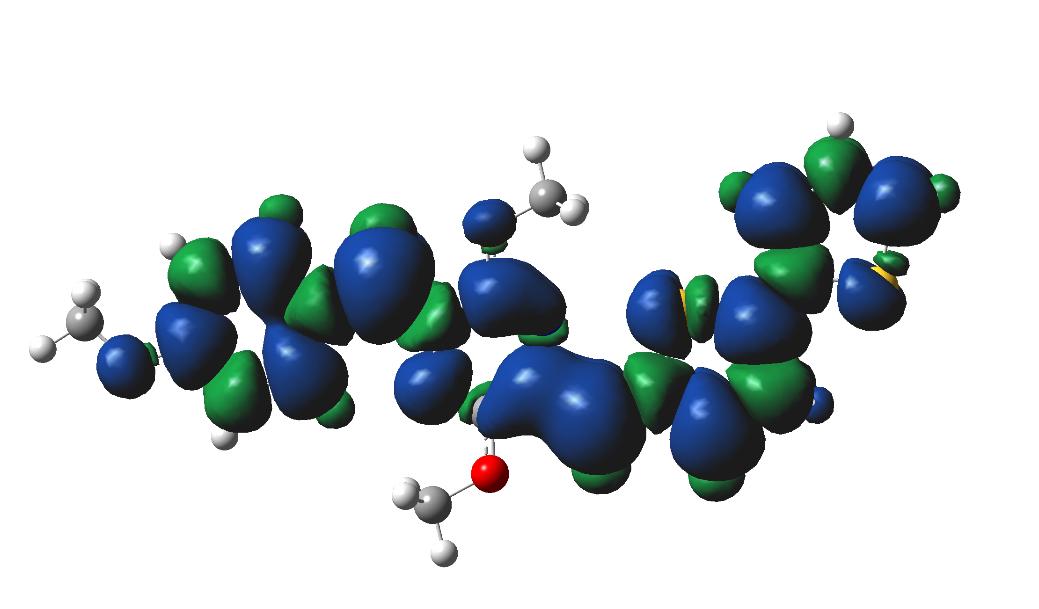
**

**AsOMe**

**
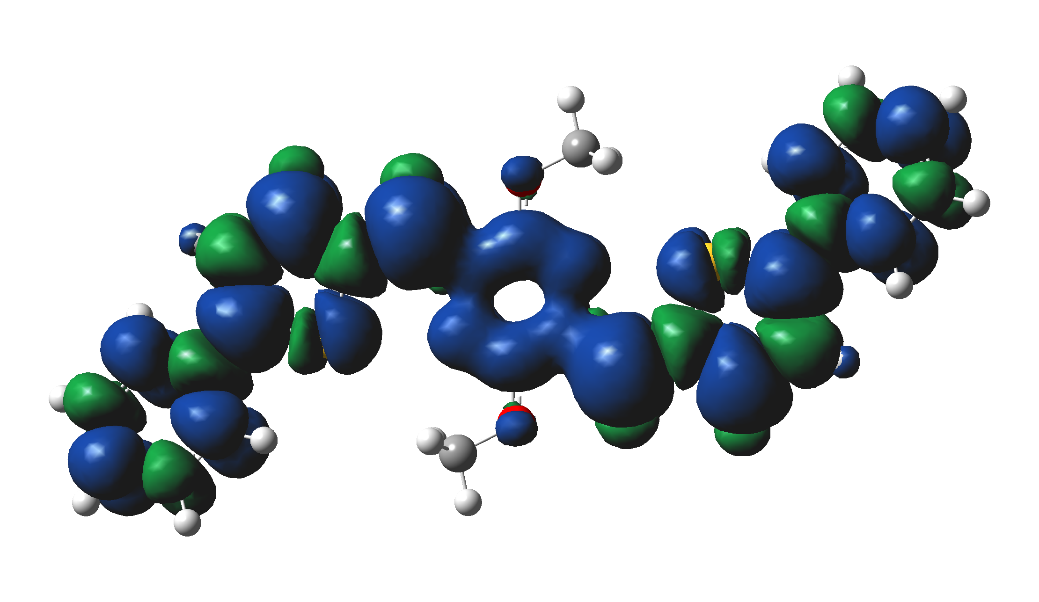
**

**TPh**

**
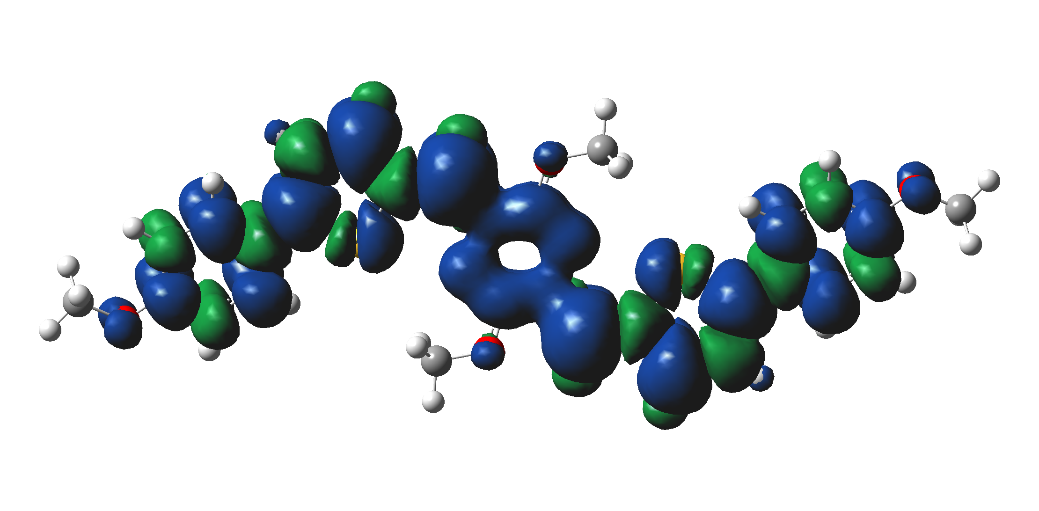
**

**TPhOMe**

**Figure S51** Spin Density @ relaxed T_1_ calculated by wB97XD/6-311+(G(d) in toluene.

# **Absorption and emission in thin film**

Wavelength/nm

Wavelength/nm

**Figure S52** Absorption, emission and excitation spectra of the thin films at different wavelengths of all the compounds.

**Figure S53** Comparison between absorption and emission spectra of the compounds in toluene solution (dashed line) and thin film (solid line).

**Figure S54** Comparison between photoluminescence kinetics recorded in solution (left) and in film (right) for the compounds.

# **Nanosecond Transient Absorption in solution**

**Figure S55** Triplet transient absorption matrix (a), spectra (c), aerated (b) and nitrogen-purged (d) kinetics of **AsOMe** in toluene obtained from Nanosecond Laser Flash Photolysis.

**Figure S56** Triplet transient absorption matrix (a), spectra (c), aerated (b) and nitrogen-purged (d) kinetics of **AsNMe_2_** in toluene obtained from Nanosecond Laser Flash Photolysis.

**Figure S57** Triplet transient absorption matrix (a), spectra (c), aerated (b) and nitrogen-purged (d) kinetics of **TPh** in toluene obtained from Nanosecond Laser Flash Photolysis.

**Figure S58** Triplet transient absorption matrix (a), spectra (c), aerated (b) and nitrogen-purged (d) kinetics of **TPhOMe** in toluene obtained from Nanosecond Laser Flash Photolysis.

**Table S20** Triplet properties of the investigated compounds in toluene. 𝜆_T_ : maximum wavelength of triplet absorption spectrum; 𝜏_T,air_ and 𝜏_T,N2_: triplet lifetime in air equilibrated and nitrogen purged solutions, respectively; ε_T_ : triplet molar absorption coeﬃcient; Φ_T_ : triplet quantum yield.

| **Compound** | **𝜆_T_/nm** | **τ_air_/ns** | **τ_N2_/ns** | **ε_T_/M^-1^ cm^-1^** | **Φ_T_ x ε_T_/M^-1^ cm^-1^** | **Φ_T_** |
| --- | --- | --- | --- | --- | --- | --- |
| AsOMe | 610 | 120 | 6900 | 42100 | 12800 | 0.30 |
| AsNMe_2_ | 640 | 130 | 6500 | 12900 | 470 | 0.036 |
| TPh | 620 | 150 | 8000 | 39800 | 7000 | 0.18 |
| TPhOMe | 640 | 130 | 11400 | 30700 | 5600 | 0.18 |

# **Nanosecond Transient Absorption in thin film**

**Figure S59** Triplet transient absorption matrix (a), spectra (b) and kinetic (c) of **AsOMe** in thin film obtained from Nanosecond Laser Flash Photolysis.

τ_1_=110 ns

τ_2_=3900 ns

**Figure S60** Triplet transient absorption matrix (a), spectra (b) and kinetic (c) of **AsNMe_2_** in thin film obtained from Nanosecond Laser Flash Photolysis.

τ_1_=120 ns

τ_2_=1800 ns

**Figure S61** Triplet transient absorption matrix (a), spectra (b) and kinetic (c) of **TPh** in thin film obtained from Nanosecond Laser Flash Photolysis.

τ_1_=40 ns

τ_2_=1100 ns

**Figure S62** Triplet transient absorption matrix (a), spectra (b) and kinetic (c) of **TPhOMe** in thin film obtained from Nanosecond Laser Flash Photolysis.

**Table S21** Triplet properties of the investigated compounds in thin film. 𝜆_T_ : maximum wavelength of triplet absorption spectrum; 𝜏_T_ : triplet lifetime.

| **Compound** | **𝜆_T_/nm** | **τ_T_/ns** |
| --- | --- | --- |
| AsOMe | 610 | 11000 |
| AsNMe_2_ | 650 | 3900 |
| TPh | 620 | 1800 |
| TPhOMe | 650 | 1100 |

# **Triplet energies sensitizations**

**Figure S63** Triplet-triplet sensitization experiment from **Anthracene** (E_T_ = 1.85 eV) to **AsOMe** in N_2_-purged CHCl_3_, obtained by laser flash photolysis at 355 nm.

**Figure S64** Triplet-triplet sensitization experiment from **AsOMe** to **Tetracene** (E_T_ = 1.27 eV) in N_2_-purged CHCl_3_, obtained by laser flash photolysis at 355 nm.

**Table S22** Results of triplet-triplet sensitization experiment employing Tetracene (E_T_ = 1.27 eV) together with **AsOMe** in N_2_-purged CHCl_3_(k_diff_ = 1.2×10^10^ M^−1^ s^−1^), obtained by laser flash photolysis at 355 nm.

| **AsOMe** | **τ_T_^0^/μs** | **τ_T_/μs** | **[Q] /10^-5^ M** | **k_q_ / 10^9^M^-1^ s^-1^** | **E_T,AsOMe_/ eV** |
| --- | --- | --- | --- | --- | --- |
| Tetracene | 30.5 | 10.4 | 1.3 | 5.1 | 1.29 |
|  | | | |  | **1.3** |

Sandros equation:

k_TET_ = k_diff_ / [1 + exp ( −(E_T,D_ − E_T,A_) ∕ k_B_T]

where k_TET_ is the rate constant for energy transfer (M^−1^ s^−1^), k_diff_ is the rate constant of energy transfer at the diffusion limit (M^−1^ s^−1^), E_T,D_ and E_T,A_ are the donor and acceptor T_1_ energies, respectively, k_B_ is the Boltzmann constant, and T is temperature. By utilising the Sandros equation for the donor –acceptor pair, the triplet energy has been estimated for **AsOMe** in CHCl_3_ at 298 K.

**Figure S65** Triplet-triplet sensitization experiment from **Tetracene** (E_T_ = 1.27 eV) to **AsNMe_2_** in N_2_-purged CHCl_3_, obtained by laser flash photolysis at 355 nm.

**Figure S66** Triplet-triplet sensitization experiment from **Rubrene** (E_T_ = 1.14 eV) to **AsNMe_2_** in N_2_-purged CHCl_3_, obtained by laser flash photolysis at 355 nm (not the best).

**Table S23** Results of triplet-triplet sensitization experiment employing Tetracene (E_T_ = 1.27 eV) and Rubrene (E_T_ = 1.14 eV) together with **AsNMe_2_** in N_2_-purged CHCl_3_(k_diff_ = 1.2×10^10^ M^−1^ s^−1^), obtained by laser flash photolysis at 355 nm.

| **AsNMe_2_** | **τ_T_^0^/μs** | **τ_T_/μs** | **[Q] /10^-5^ M** | **k_q_ / 10^9^M^-1^ s^-1^** | **E_T,AsNMe2_/ eV** |
| --- | --- | --- | --- | --- | --- |
| Tetracene | 64.8 | 2.7 | 1.7 | 21 | - |
| Rubrene | 48.1 | 2.5 | 5.0 | 7.6 | **1.13** |

**Figure S67** Triplet-triplet sensitization experiment from **Tetracene** (E_T_ = 1.27 eV) to **TPh** in N_2_-purged CHCl_3_, obtained by laser flash photolysis at 355 nm.

**Figure S68** Triplet-triplet sensitization experiment from **Rubrene** (E_T_ = 1.14 eV) to **TPh** in N_2_-purged CHCl_3_, obtained by laser flash photolysis at 355 nm.

**Table S24** Results of triplet-triplet sensitization experiments employing Tetracene (E_T_ = 1.27 eV) and Rubrene (E_T_ = 1.14 eV) together with **TPh** in N_2_-purged CHCl_3_ (k_diff_ = 1.2×10^10^ M^−1^ s^−1^), obtained by laser flash photolysis at 355 nm.

| **TPh** | **τ_T_^0^/μs** | **τ_T_/μs** | **[Q] /10^-5^ M** | **k_q_ / 10^9^M^-1^ s^-1^** | **E_T,TPh_/ eV** |
| --- | --- | --- | --- | --- | --- |
| Tetracene | 130 | 15.3 | 2.0 | 2.9 | 1.30 |
| Rubrene | 34.9 | 2.4 | 8.2 | 4.7 | 1.15 |
|  | | | | Average | **1.2** |

**Figure S69** Triplet-triplet sensitization experiment from **Tetracene** (E_T_ = 1.27 eV) to **TPhOMe** in N_2_-purged CHCl_3_, obtained by laser flash photolysis at 355 nm.

**Figure S70** Triplet-triplet sensitization experiment from **Rubrene** (E_T_ = 1.14 eV) to **TPhOMe** in N_2_-purged CHCl_3_, obtained by laser flash photolysis at 355 nm.

**Table S25** Results of triplet-triplet sensitization experiments employingTetracene (E_T_ = 1.27 eV) and Rubrene (E_T_ = 1.14 eV) together with **TPhOMe** in N_2_-purged CHCl_3_ (k_diff_ = 1.2×10^10^ M^−1^ s^−1^), obtained by laser flash photolysis at 355 nm.

| **TPhOMe** | **τ_T_^0^/μs** | **τ_T_/μs** | **[Q] /10^-5^ M** | **k_q_ / 10^9^M^-1^ s^-1^** | **E_T,TPhOMe_/ eV** |
| --- | --- | --- | --- | --- | --- |
| Tetracene | 109.8 | 13.5 | 1.3 | 5.3 | 1.28 |
| Rubrene | 84.6 | 3.1 | 3.1 | 6.4 | 1.14 |
|  | | | | Average | **1.2** |

# **Femtosecond Transient Absorption in solution**

**Figure S71** Femtosecond transient absorption matrix (a), spectra at different delay times (c), kinetics at different wavelengths (d), Evolution Associated Spectra (EAS) and lifetimes (b) obtained from Global Analysis for **AsOMe** in toluene.

Wavelength/nm

**Figure S72** Femtosecond transient absorption matrix (a) spectra at different delay times (c), kinetics at different wavelengths (d), Evolution Associated Spectra (EAS) and lifetimes (b) obtained from Global Analysis for **AsNMe_2_** in toluene.

**Figure S73** Femtosecond transient absorption matrix (a) spectra at different delay times (c), kinetics at different wavelengths (d), Evolution Associated Spectra (EAS) and lifetimes (b) obtained from Global Analysis for **TPh** in toluene.

**Figure S74** Femtosecond transient absorption matrix (a) spectra at different delay times (c), kinetics at different wavelengths (d), Evolution Associated Spectra (EAS) and lifetimes (b) obtained from Global Analysis for **TPhOMe** in toluene.

# **Femtosecond Fluorescence Up conversion in solution**

**Figure S75** Femtosecond fluorescence up conversion matrix (a) spectra at different delay times (c), kinetics at different wavelengths (d), Evolution Associated Spectra (EAS) and lifetimes (b) obtained from Global Analysis for **AsOMe** in toluene.

**Figure S76** Femtosecond fluorescence up conversion matrix (a) spectra at different delay times (c), kinetics at different wavelengths (d), Evolution Associated Spectra (EAS) and lifetimes (b) obtained from Global Analysis for **AsNMe_2_** in toluene.

**Figure S77** Femtosecond fluorescence up conversion matrix (a) spectra at different delay times (c), kinetics at different wavelengths (d), Evolution Associated Spectra (EAS) and lifetimes (b) obtained from Global Analysis for **TPh** in Tol.

**Figure S78** Femtosecond fluorescence up conversion matrix (a) spectra at different delay times (c), kinetics at different wavelengths (d), Evolution Associated Spectra (EAS) and lifetimes (b) obtained from Global Analysis for **TPhOMe** in Tol.

# **Femtosecond Transient Absorption in thin film**

Wavelength/nm

**Figure S79** Femtosecond transient absorption matrix (a) spectra at different delay times (c), kinetics at different wavelengths (d), Evolution Associated Spectra (EAS) and lifetimes (b) obtained from Global Analysis for **AsOMe** in thin film.

Wavelength/nm

**Figure S80** Femtosecond transient absorption matrix (a) spectra at different delay times (c), kinetics at different wavelengths (d), Evolution Associated Spectra (EAS) and lifetimes (b) obtained from Global Analysis for **AsNMe_2_** in thin film.

**Figure S81** Femtosecond transient absorption matrix (a) spectra at different delay times (c), kinetics at different wavelengths (d), Evolution Associated Spectra (EAS) and lifetimes (b) obtained from Global Analysis for **TPh** in thin film.

**Figure S82** Femtosecond transient absorption matrix (a) spectra at different delay times (c), kinetics at different wavelengths (d), Evolution Associated Spectra (EAS) and lifetimes (b) obtained from Global Analysis for **TPhOMe** in thin film.

# **Triplet Yield Calculation**

Triplet yields were evaluated from the temporal dynamics of the lowest excited singlet and triplet state populations, as obtained through analysis of the femtosecond transient absorption data, according to a procedure already described in the literature, and here detailed for the films of the investigated compounds.^[15–17]^

**AsOMe**

Global Fitting of the femtosecond transient absorption data was carried out using the Glotaran software which provided us with the Evolution Associated Spectra of the four exponential components and their temporal composition (Figure S83).

**Figure S83.** Evolution Associated Spectra (left) and composition in time (right) of the four exponential components resulting from global fitting of the femtosecond transient absorption data of **AsOMe** in film.

To obtain the spectral shapes of the excited singlet and triplet states the transient spectra at time delays of 0.23 and 2210 ps were selected, respectively (Figure S84). In fact, at these time delays abundances of the singlet and triplet transients were at the maxima ΔA value in the femtosecond transient absorption data (Figure S83, right side).

**Figure S84.** Transient absorption spectra at 0.234 and 2210 ps time delays from excitation recorded for **AsOMe** in film.

The spectra of the lowest singlet and triplet excited states must be related through the GSB they share in common. The ground state absorption spectrum was scaled and subtracted from the 0.234/2210 ps transient spectra in order to remove the GSB contribution. The ground state absorption spectrum was normalized to the transient absorption spectrum at the peak of the ground state bleaching, and then subtracted. The normalization was the method employed to determine the “right” amount of ground state absorption to be subtracted each time. The resulting spectra only show the S_1_/T_1_ ESA relative to a known amount of GSB (Figure S85).

**Figure S85.** Transient (black) and steady state (red) absorption spectra used to reconstruct the absorption spectra of the excited singlet (grey, left) and triplet (green, right) states.

The S_1_ and T_1_ spectra are then normalized to the GSB they share, resulting in two spectra that are quantitatively related (Figure S86).

**Figure S86.** Excited state absorption spectra, normalized to GSB, used to correct the composition profile in Figure S83 and to obtain population dynamics in Figure S87.

The quantitatively related spectra of S_1_ and T_1_ were used to correct the composition profiles previously shown. The temporal composition of the components resulting from the global fitting reported in Figure S83 indeed related to the differential absorbance measured during the ultrafast absorption experiments. Therefore, according to the Lambert−Beer law, it is dependent on both the absorption ability and the concentration of S_1_ and T_1_. From the quantitatively related S_1_ and T_1_ spectra reported in Figure S85, it is clear that the ratio between the triplet excited state absorption at its peak (0.627 at 623 nm) and the singlet excited state absorption at its peak (0.577 at 725 nm) is $\frac{\varepsilon_{T}}{\varepsilon_{S}}=1.09$. By scaling the S_1_ and T_1_ temporal compositions in Figure S83 (right side) for this factor (multiplying the singlet profile by 1.09), the correct concentration profiles were obtained: $\frac{c_{S}}{c_{T}}=\frac{{\Delta A}_{S}}{{\Delta A}_{T}}\times\frac{\varepsilon_{T}}{\varepsilon_{S}}$. From the population profiles normalized at the singlet population peak (Figure S87), a triplet quantum yield of **108%** was estimated for **AsOMe** in film.

**Figure S87.** Population dynamics of the excited singlet and triplet states for **AsOMe** in film.

**AsNMe_2_**

Global Fitting of the femtosecond transient absorption data was carried out using the Glotaran software which provided us with the Evolution Associated Spectra of the four exponential components and their temporal composition (Figure S88).

**Figure S88.** Evolution Associated Spectra (left) and composition in time (right) of the four exponential components resulting from global fitting of the femtosecond transient absorption data of **AsNMe_2_** in film.

To obtain the spectral shapes of the excited singlet and triplet states the transient spectra at time delays of 0.349 and 2750 ps were selected, respectively (Figure S89). In fact, at these time delays abundances of the singlet and triplet transients were at the maxima ΔA value in the femtosecond transient absorption data (Figure S88, right side).

**Figure S89.** Transient absorption spectra at 0.249 and 2750 ps time delays from excitation recorded for **AsNMe_2_** in film.

The spectra of the lowest singlet and triplet excited states must be related through the GSB they share in common. The ground state absorption spectrum was scaled and subtracted from the 0.249/2750 ps transient spectra in order to remove the GSB contribution. The ground state absorption spectrum was normalized to the transient absorption spectrum at the peak of the ground state bleaching, and then subtracted. The normalization was the method employed to determine the “right” amount of ground state absorption to be subtracted each time. The resulting spectra only show the S_1_/T_1_ ESA relative to a known amount of GSB (Figure S90).

**Figure S90.** Transient (black) and steady state (red) absorption spectra used to reconstruct the absorption spectra of the excited singlet (grey, left) and triplet (green, right) states.

The S_1_ and T_1_ spectra are then normalized to the GSB they share, resulting in two spectra that are quantitatively related (Figure S91).

**Figure S91.** Excited state absorption spectra, normalized to GSB, used to correct the composition profile in Figure S88 and to obtain population dynamics in Figure S92.

The quantitatively related spectra of S_1_ and T_1_ were used to correct the composition profiles previously shown. The temporal composition of the components resulting from the global fitting reported in Figure S88 indeed related to the differential absorbance measured during the ultrafast absorption experiments. Therefore, according to the Lambert−Beer law, it is dependent on both the absorption ability and the concentration of S_1_ and T_1_. From the quantitatively related S_1_ and T_1_spectra reported in Figure S90, it is clear that the ratio between the triplet excited state absorption at its peak (ca. 0.6 at 623 nm) and the singlet excited state absorption at its peak (1.05 at 730 nm) is $\frac{\varepsilon_{T}}{\varepsilon_{S}}=0.570$. By scaling the S_1_ and T_1_ temporal compositions in Figure S88 (right side) for this factor (multiplying the singlet profile by 0.570), the correct concentration profiles were obtained: $\frac{c_{S}}{c_{T}}=\frac{{\Delta A}_{S}}{{\Delta A}_{T}}\times\frac{\varepsilon_{T}}{\varepsilon_{S}}$. From the population profiles normalized at the singlet population peak (Figure S92), a triplet quantum yield of **209%** was estimated for **AsNMe_2_** in film.

**Figure S92.** Population dynamics of the excited singlet and triplet states for **AsNMe_2_** in film.

**TPh**

Global Fitting of the femtosecond transient absorption data was carried out using the Glotaran software which provided us with the Evolution Associated Spectra of the four exponential components and their temporal composition (Figure S93).

**Figure S93.** Evolution Associated Spectra (left) and composition in time (right) of the four exponential components resulting from global fitting of the femtosecond transient absorption data of **TPh** in film.

To obtain the spectral shapes of the excited singlet and triplet states the transient spectra at time delays of 0.217 and 3080 ps were selected, respectively (Figure S94). In fact, at these time delays abundances of the singlet and triplet transients were at the maxima ΔA value in the femtosecond transient absorption data (Figure S93, right side).

**Figure S94.** Transient absorption spectra at 0.217 and 3080 ps time delays from excitation recorded for **TPH** in film.

The spectra of the lowest singlet and triplet excited states must be related through the GSB they share in common. The ground state absorption spectrum was scaled and subtracted from the 0.217/3080 ps transient spectra in order to remove the GSB contribution. The ground state absorption spectrum was normalized to the transient absorption spectrum at the peak of the ground state bleaching, and then subtracted. The normalization was the method employed to determine the “right” amount of ground state absorption to be subtracted each time. The resulting spectra only show the S_1_/T_1_ ESA relative to a known amount of GSB (Figure S95).

**Figure S95.** Transient (black) and steady state (red) absorption spectra used to reconstruct the absorption spectra of the excited singlet (grey, left) and triplet (green, right) states.

The S_1_ and T_1_ spectra are then normalized to the GSB they share, resulting in two spectra that are quantitatively related (Figure S96).

**Figure S96.** Excited state absorption spectra, normalized to GSB, used to correct the composition profile in Figure S93 and to obtain population dynamics in Figure S97.

The quantitatively related spectra of S_1_ and T_1_ were used to correct the composition profiles previously shown. The temporal composition of the components resulting from the global fitting reported in Figure S93 indeed related to the differential absorbance measured during the ultrafast absorption experiments. Therefore, according to the Lambert−Beer law, it is dependent on both the absorption ability and the concentration of S_1_ and T_1_. From the quantitatively related S_1_ and T_1_ spectra reported in Figure S96, it is clear that the ratio between the triplet excited state absorption at its peak (0.568 at 670 nm) and the singlet excited state absorption at its peak (0.600 at 745 nm) is $\frac{\varepsilon_{T}}{\varepsilon_{S}}=0.947$. By scaling the S_1_ and T_1_ temporal compositions in Figure S93 (right side) for this factor (multiplying the singlet profile by 0.947), the correct concentration profiles were obtained: $\frac{c_{S}}{c_{T}}=\frac{{\Delta A}_{S}}{{\Delta A}_{T}}\times\frac{\varepsilon_{T}}{\varepsilon_{S}}$. From the population profiles normalized at the singlet population peak (Figure S97), a triplet quantum yield of **123%** was estimated for **TPh** in film.

**Figure S97.** Population dynamics of the excited singlet and triplet states for **TPh** in film.

**TPhOMe**

Global Fitting of the femtosecond transient absorption data was carried out using the Glotaran software which provided us with the Evolution Associated Spectra of the four exponential components and their temporal composition (Figure S98).

**Figure S98.** Evolution Associated Spectra (left) and composition in time (right) of the four exponential components resulting from global fitting of the femtosecond transient absorption data of **TPhOMe** in film.

To obtain the spectral shapes of the excited singlet and triplet states the transient spectra at time delays of 0.242 and 2900 ps were selected, respectively (Figure S99). In fact, at these time delays abundances of the singlet and triplet transients were at the maxima ΔA value in the femtosecond transient absorption data (Figure S98, right side).

**Figure S99.** Transient absorption spectra at 0.242 and 2900 ps time delays from excitation recorded for **TPhOMe** in film.

The spectra of the lowest singlet and triplet excited states must be related through the GSB they share in common. The ground state absorption spectrum was scaled and subtracted from the 0.242/2900 ps transient spectra in order to remove the GSB contribution. The ground state absorption spectrum was normalized to the transient absorption spectrum at the peak of the ground state bleaching, and then subtracted. The normalization was the method employed to determine the “right” amount of ground state absorption to be subtracted each time. The resulting spectra only show the S_1_/T_1_ ESA relative to a known amount of GSB (Figure S100).

**Figure S100.** Transient (black) and steady state (red) absorption spectra used to reconstruct the absorption spectra of the excited singlet (grey, left) and triplet (green, right) states.

The S_1_ and T_1_ spectra are then normalized to the GSB they share, resulting in two spectra that are quantitatively related (Figure S101).

**Figure S101.** Excited state absorption spectra, normalized to GSB, used to correct the composition profile in Figure S98 and to obtain population dynamics in Figure S102.

The quantitatively related spectra of S_1_ and T_1_ were used to correct the composition profiles previously shown. The temporal composition of the components resulting from the global fitting reported in Figure S98 indeed related to the differential absorbance measured during the ultrafast absorption experiments. Therefore, according to the Lambert−Beer law, it is dependent on both the absorption ability and the concentration of S_1_ and T_1_. From the quantitatively related S_1_ and T_1_spectra reported in Figure S101, it is clear that the ratio between the triplet excited state absorption at its peak (0.546 at 660 nm) and the singlet excited state absorption at its peak (0.470 at 735 nm) is $\frac{\varepsilon_{T}}{\varepsilon_{S}}=1.16$. By scaling the S_1_ and T_1_ temporal compositions in Figure S98 (right side) for this factor (multiplying the singlet profile by 1.16), the correct concentration profiles were obtained: $\frac{c_{S}}{c_{T}}=\frac{{\Delta A}_{S}}{{\Delta A}_{T}}\times\frac{\varepsilon_{T}}{\varepsilon_{S}}$. From the population profiles normalized at the singlet population peak (Figure S102), a triplet quantum yield of **102%** was estimated for **TPhOMe** in film.

**Figure S102.** Population dynamics of the excited singlet and triplet states for **TPhOMe** in film.

# **Ultrafast Spectroscopy experiments summary**

**Table S26** Lifetimes obtained from global analysis of the fs Transient Absorption and Fluorescence Up-Conversion data for all the investigated compounds in toluene and DMSO solution and thin film.

| **Name** |  | **TA τ /ps** | **FUC τ /ps** | **Assignment** |
| --- | --- | --- | --- | --- |
| **AsOMe** | Tol | 110 | 210 | **SR** |
|  |  | 1200 | 1300 | **S_1_** |
|  |  | Inf |  | **T_1_** |
|  | DMSO | 17 |  | **SR** |
|  |  | 310 |  | **S_1_** |
|  |  | Inf |  | **T_1_** |
|  | Film | 1.1 |  | **S_1_** |
|  |  | 12 |  | **^1^(TT)** |
|  |  | 490 |  | **^1^(T…T)** |
|  |  | Inf |  | **T_1_** |
| **AsNMe_2_** | Tol | 3.6 | 0.30 | **Solv.** |
|  |  | 87 | 84 | **S_1_** |
|  |  | Inf |  | **T_1_** |
|  | DMSO | 2.6 |  | **Solv.** |
|  |  | 75 |  | **S_1_** |
|  | Film | 0.70 |  | **S_1_** |
|  |  | 9.9 |  | **^1^(TT)** |
|  |  | 160 |  | **^1^(T…T)** |
|  |  | Inf |  | **T_1_** |
| **TPh** | Tol | 88 | 140 | **SR** |
|  |  | 1100 | 1440 | **S_1_** |
|  |  | Inf |  | **T_1_** |
|  | DMSO | 2.8 |  | **Solv.** |
|  |  | 110 |  | **SR** |
|  |  | 810 |  | **S_1_** |
|  |  | Inf |  | **T_1_** |
|  | Film | 1.7 |  | **S_1_** |
|  |  | 25 |  | **^1^(TT)** |
|  |  | 500 |  | **^1^(T…T)** |
|  |  | Inf |  | **T_1_** |
| **TPhOMe** | Tol | 3.2 | 11 | **Solv.** |
|  |  | 120 | 110 | **SR** |
|  |  | 1100 | 980 | **S_1_** |
|  |  | Inf |  | **T_1_** |
|  | DMSO | 2.2 |  | **Solv.** |
|  |  | 39 |  | **SR** |
|  |  | 700 |  | **S_1_** |
|  |  | Inf |  | **T_1_** |
|  | Film | 2.1 |  | **S_1_** |
|  |  | 26 |  | **^1^(TT)** |
|  |  | 490 |  | **^1^(T…T)** |
|  |  | Inf |  | **T_1_** |

# **TR-EPR Results**

## **TR-EPR in frozen solution**


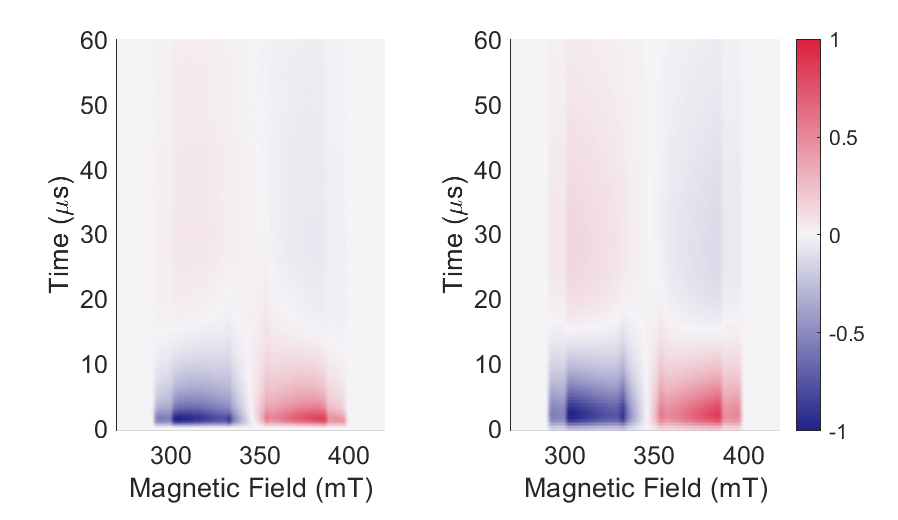


a)


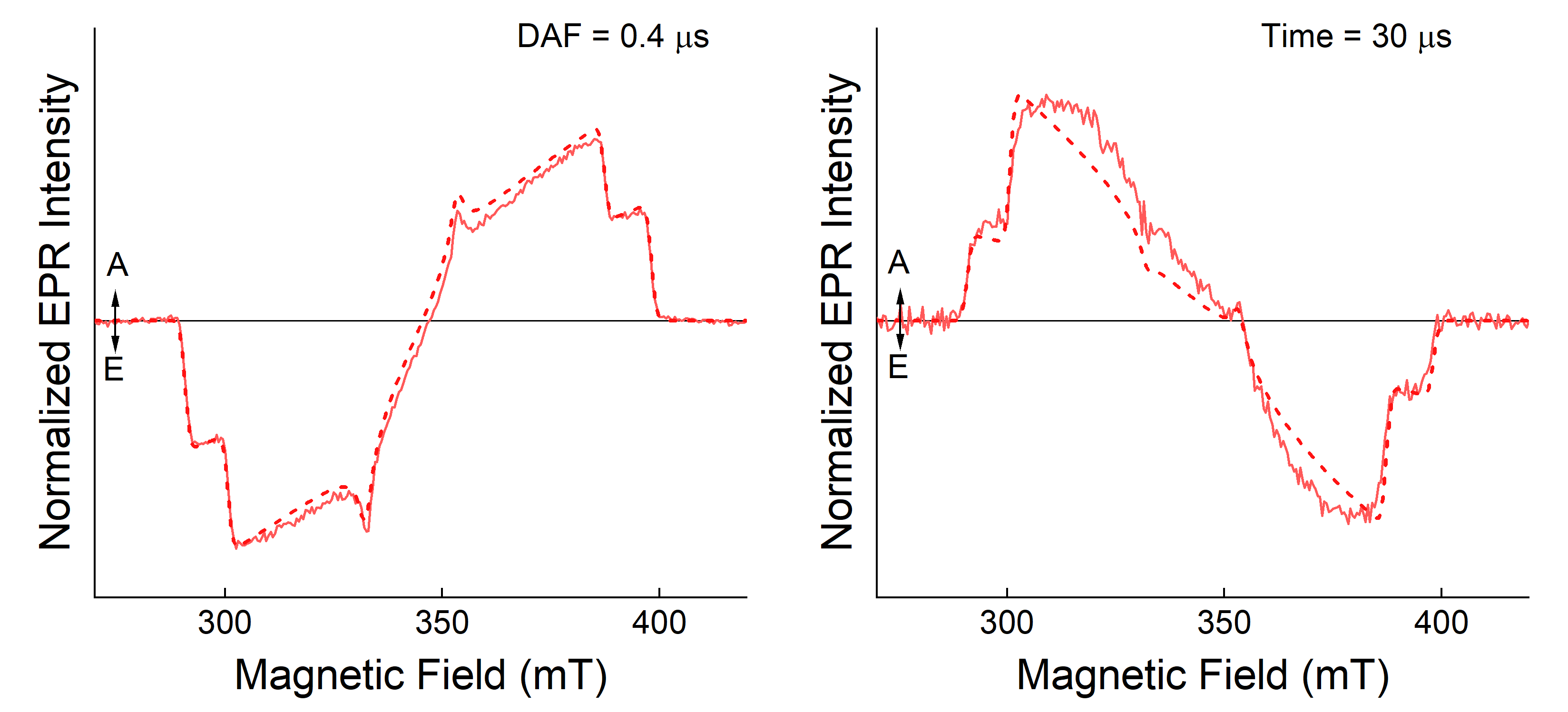


b)

**Figure S103** a) Experimental (left) and simulated (right) contour plots of the X-band isotropic TR-EPR data for the triplet state of **AsOMe**, in Tol/MeTHF 1:1 at 80 K, after photoexcitation with 2.5 mJ/pulse at 532 nm. b) Time evolution of the isotropic TR-EPR spectra and corresponding simulation (solid and dashed line respectively).


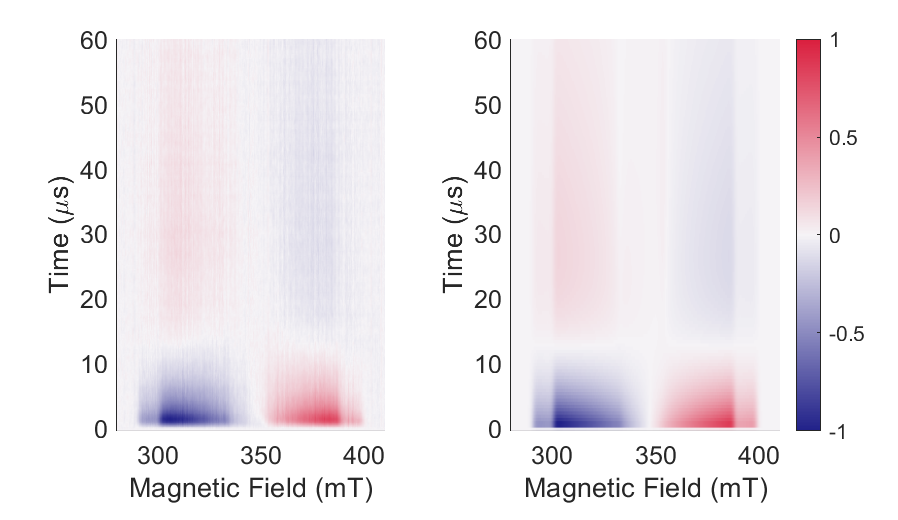


a)


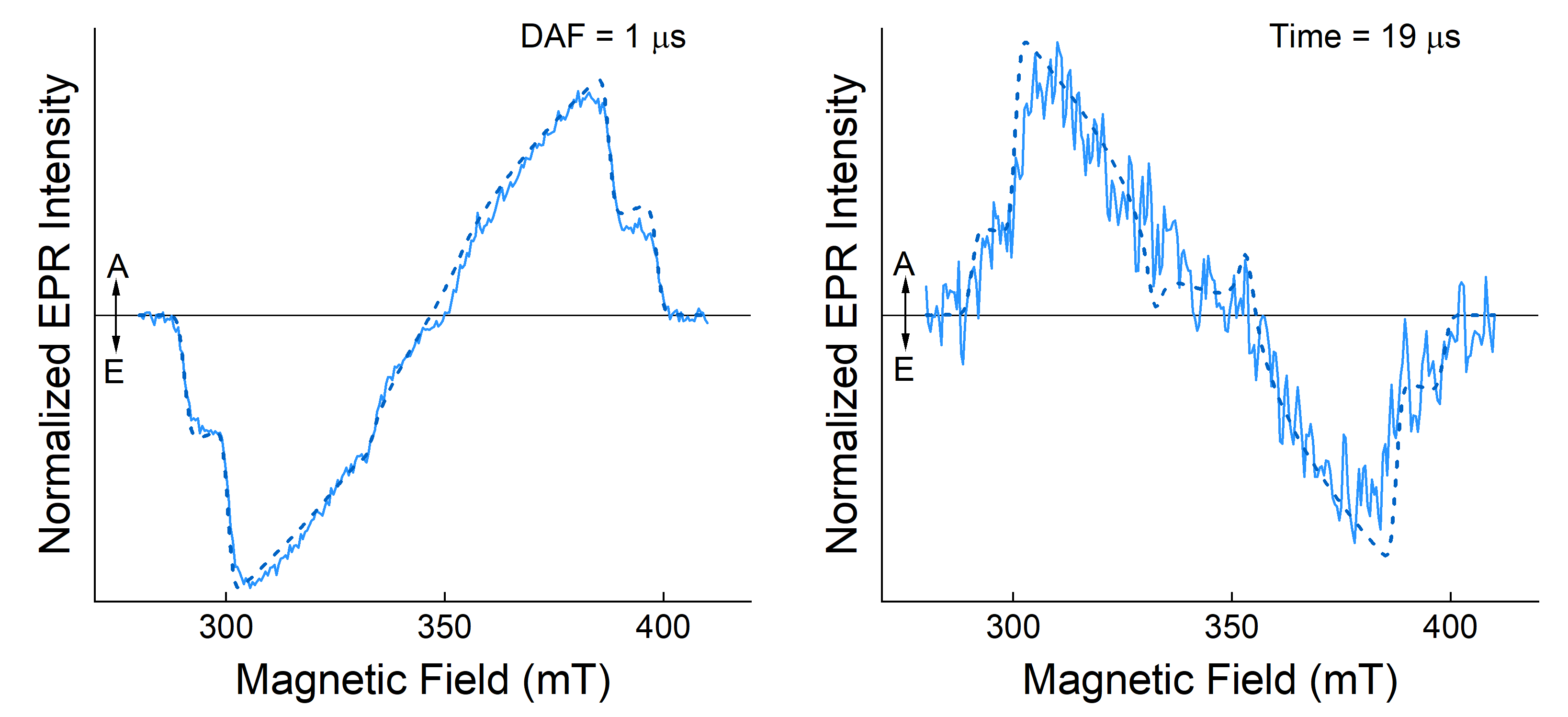


b)

**Figure S104** a) Experimental (left) and simulated (right) contour plots of the X-band isotropic TR-EPR data for **AsNMe_2_**, in Tol/DCM 1:1 at 80 K, after photoexcitation with 2.5 mJ/pulse at 532 nm. b) Time evolution of the isotropic TR-EPR spectra and corresponding simulation (solid and dashed line respectively).


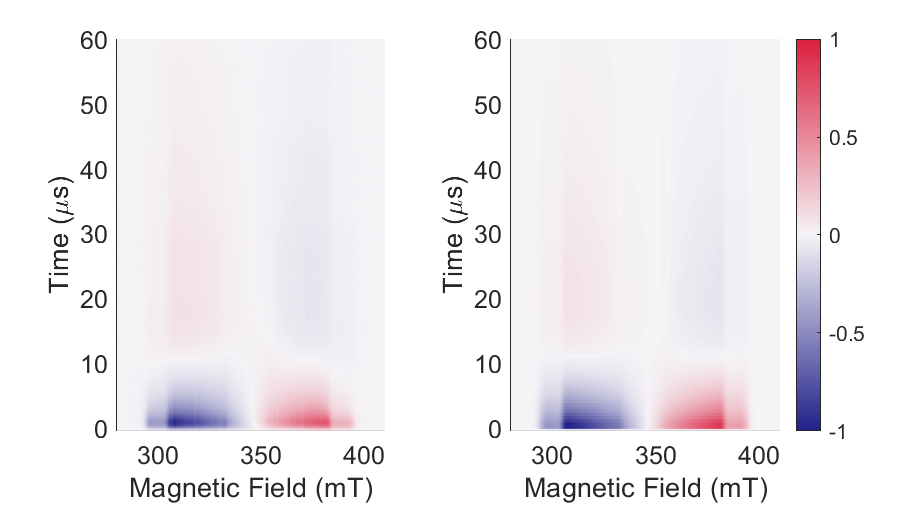


a)


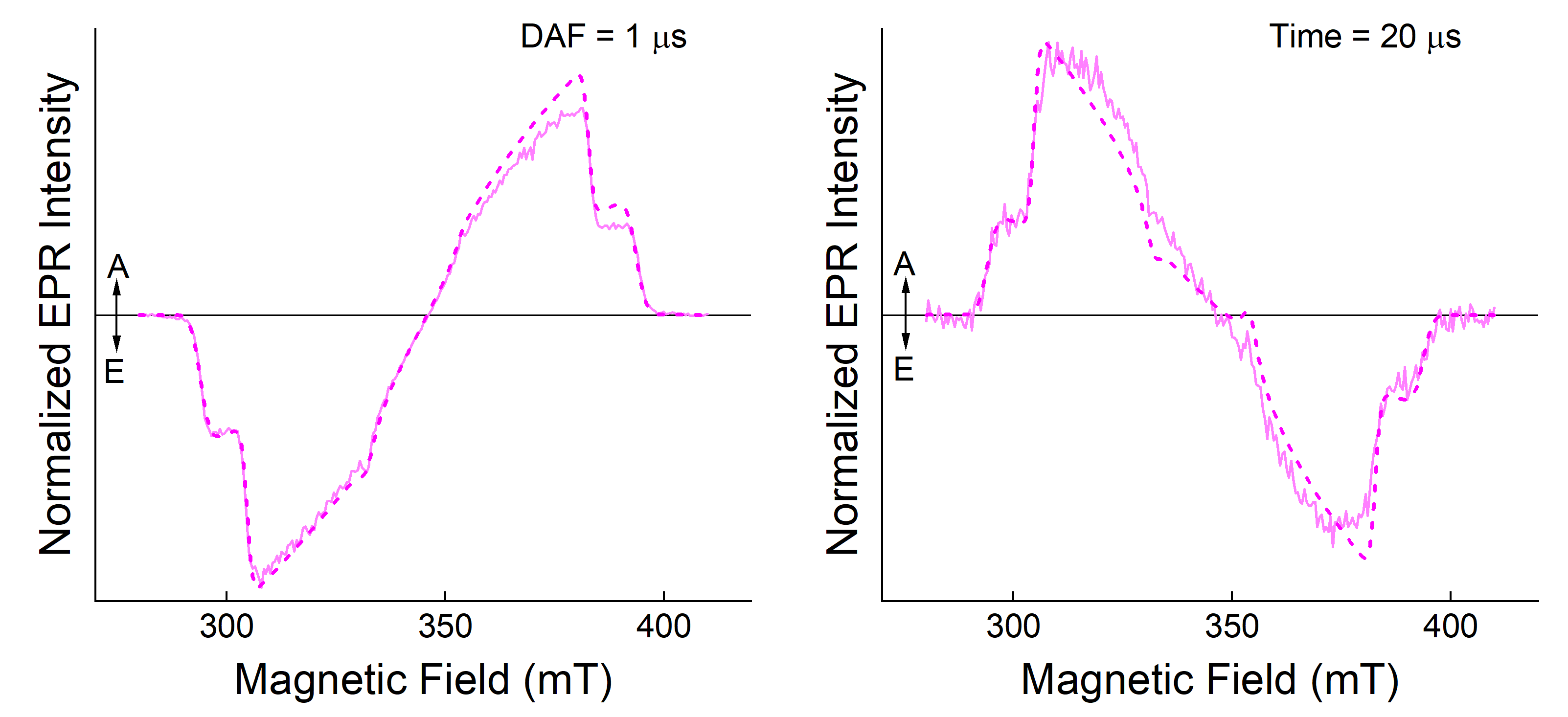


b)

**Figure S105** a) Experimental (left) and simulated (right) contour plots of the X-band isotropic TR-EPR data for **TPh**, in Tol/DCM 1:1 at 80 K, after photoexcitation with 2.5 mJ/pulse at 532 nm. b) Time evolution of the isotropic TR-EPR spectra and corresponding simulation (solid and dashed line respectively).


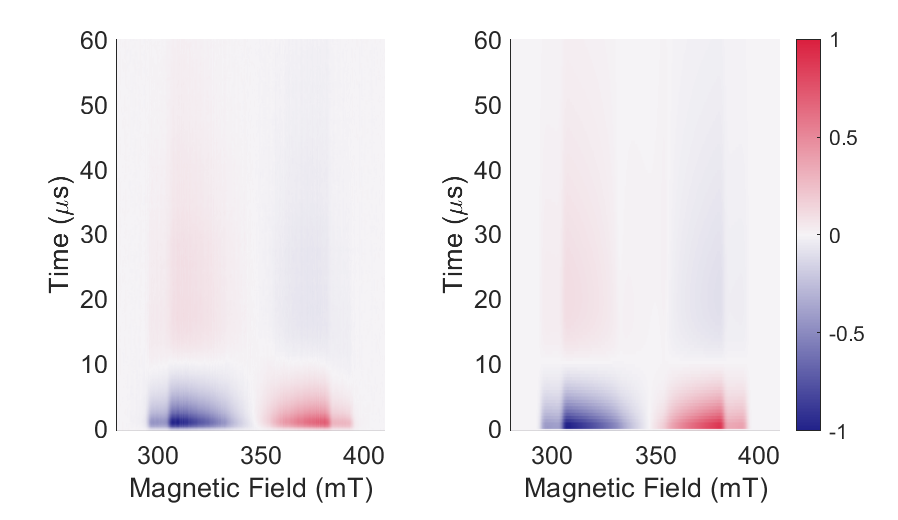


a)


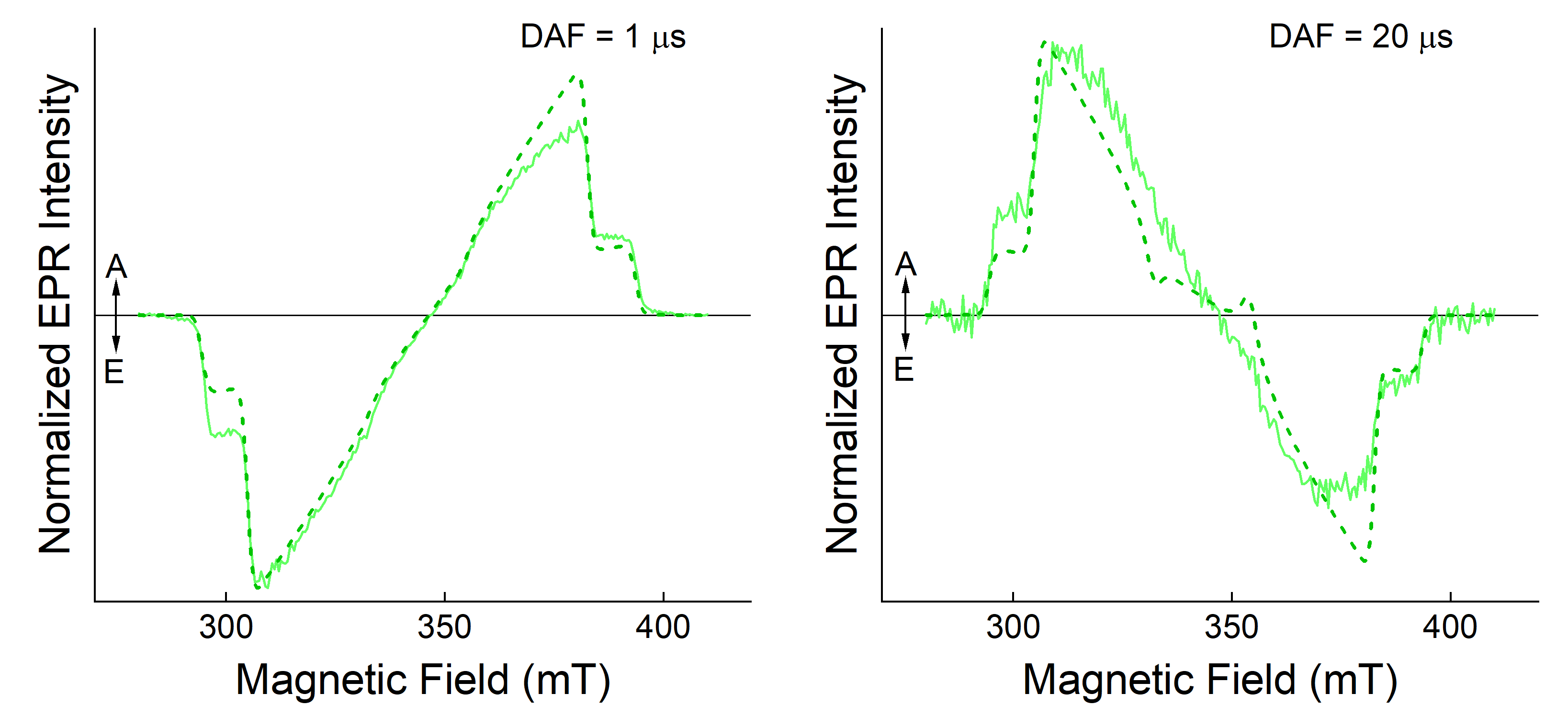


b)

**Figure S106** a) Experimental (left) and simulated (right) contour plots of the X-band isotropic TR-EPR data for **TPhOMe**, in Tol/DCM 1:1 at 80 K, after photoexcitation with 2.5 mJ/pulse at 532 nm. b) Time evolution of the isotropic TR-EPR spectra and corresponding simulation (solid and dashed line respectively).


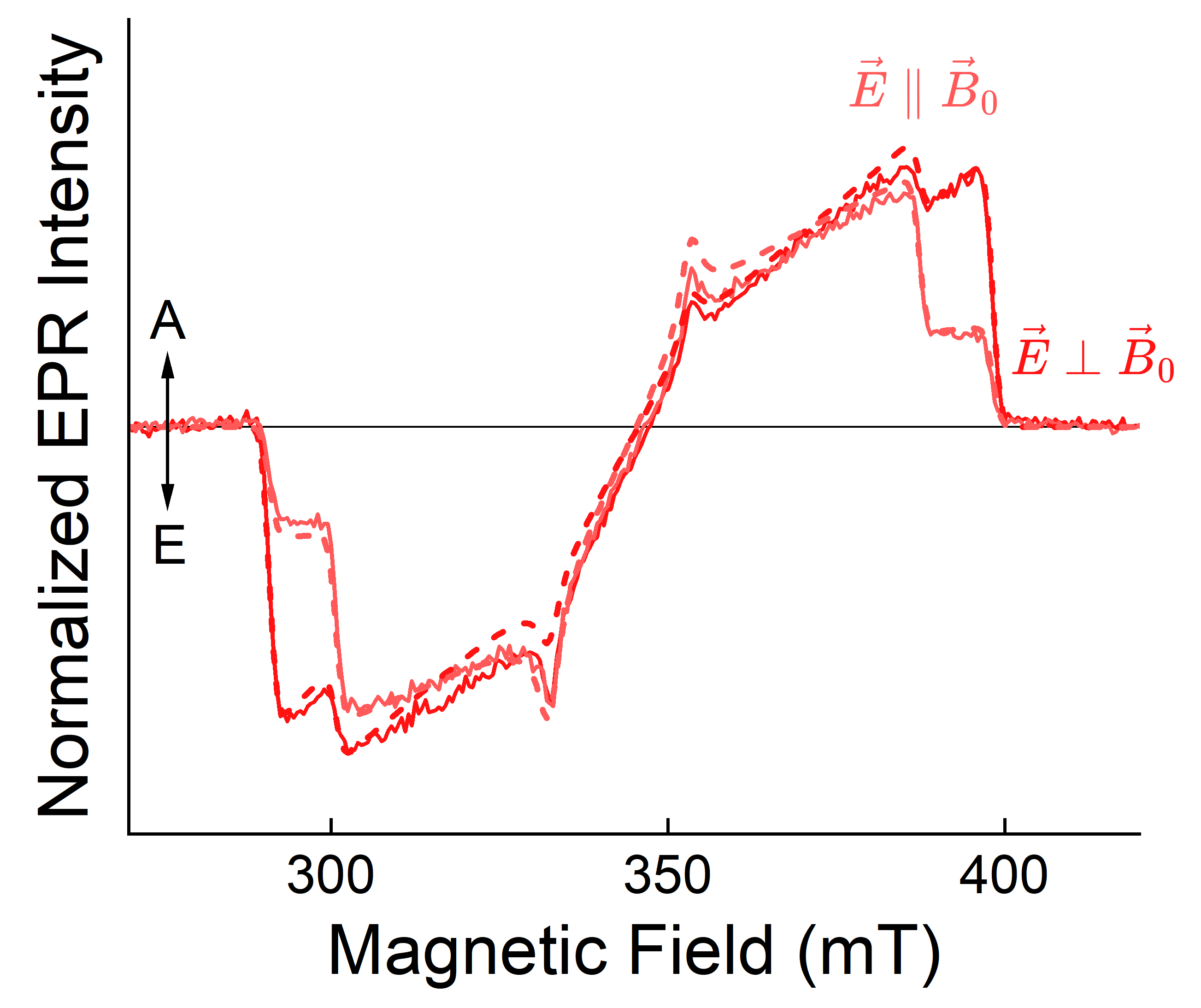

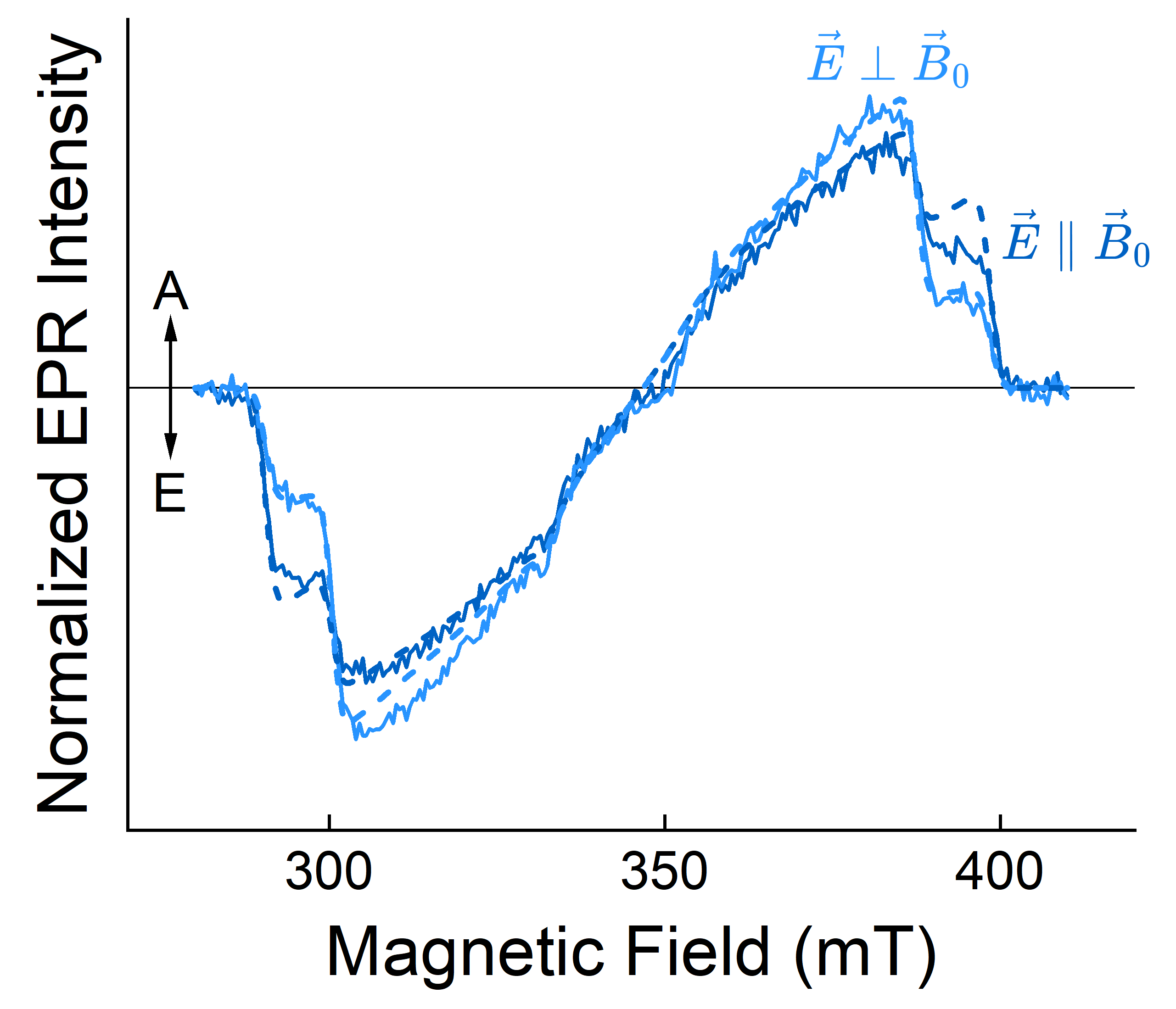

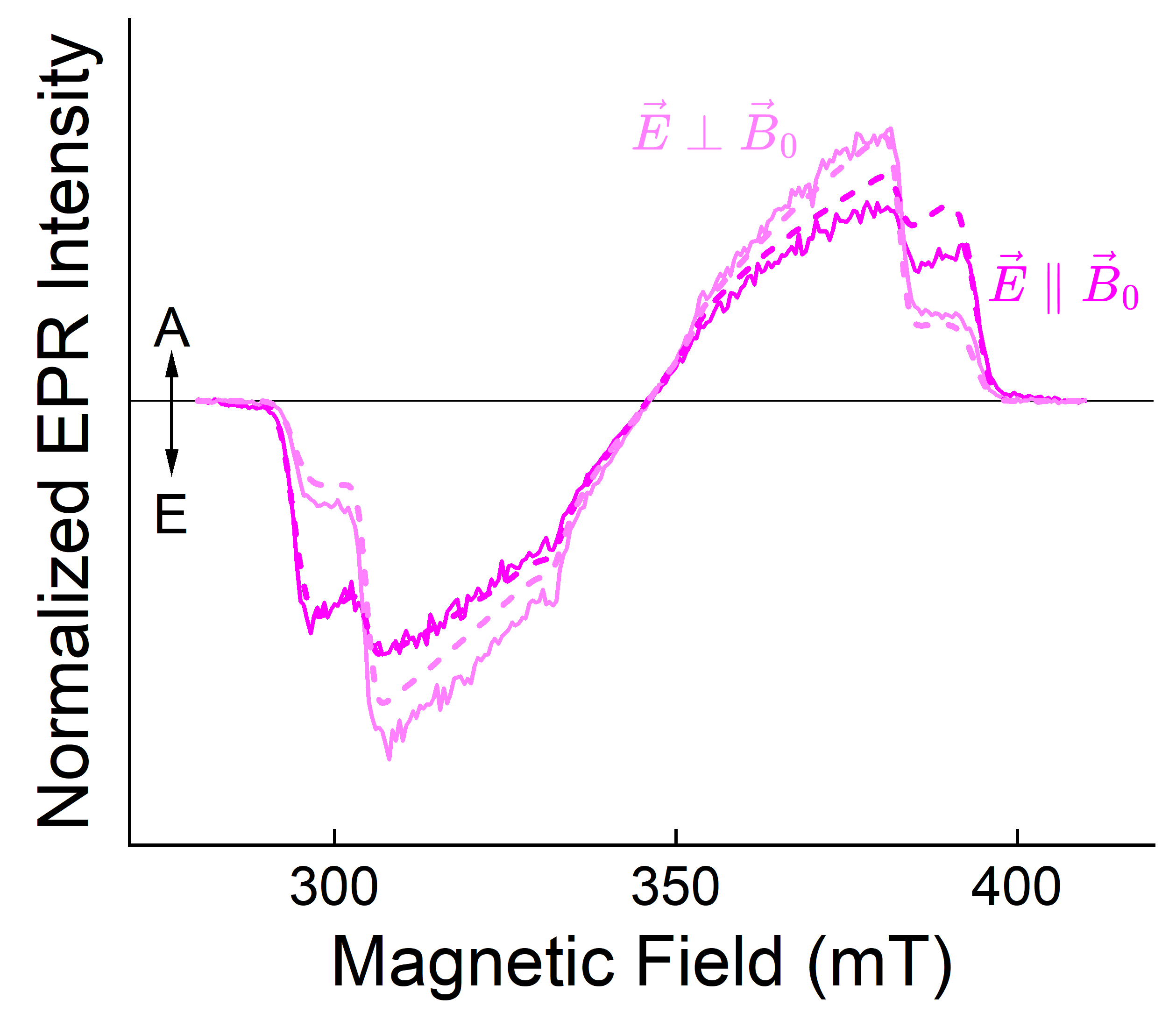

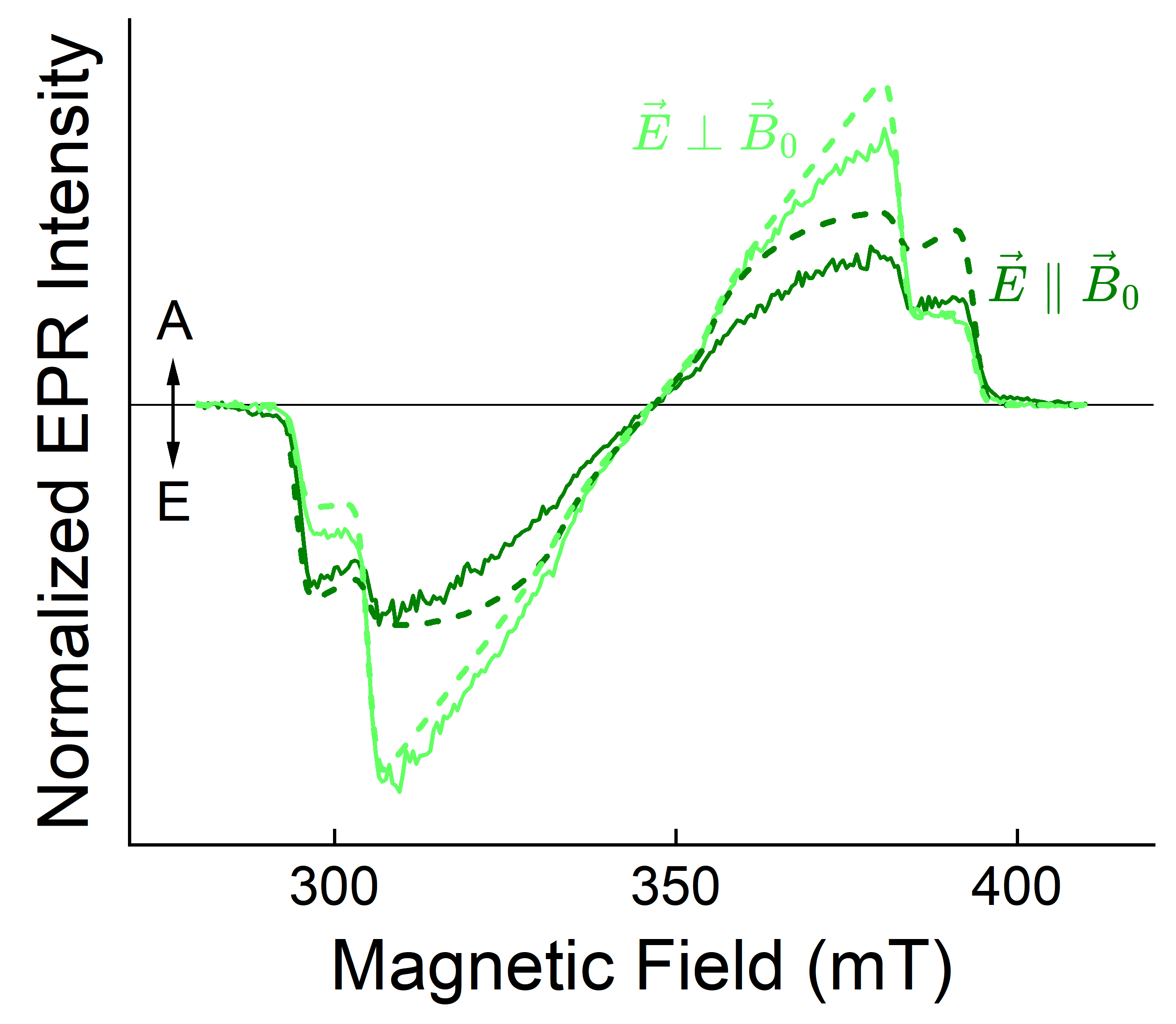


d)

c)

a)

b)

**Figure S107** X-band photoselected TR-EPR spectra of the triplet state of: a) **AsOMe**, b) **AsNMe_2_**, c) **TPh**, d) **TPhOMe** in Tol/DCM 1:1 at 80 K, **AsOMe** is in Tol/MeTHF 1:1, after photoexcitation at 532 nm using linearly polarized light parallel (dark color) and perpendicular (light color) to the external magnetic field. Simulations of the MPS effect are also shown (dashed lines).


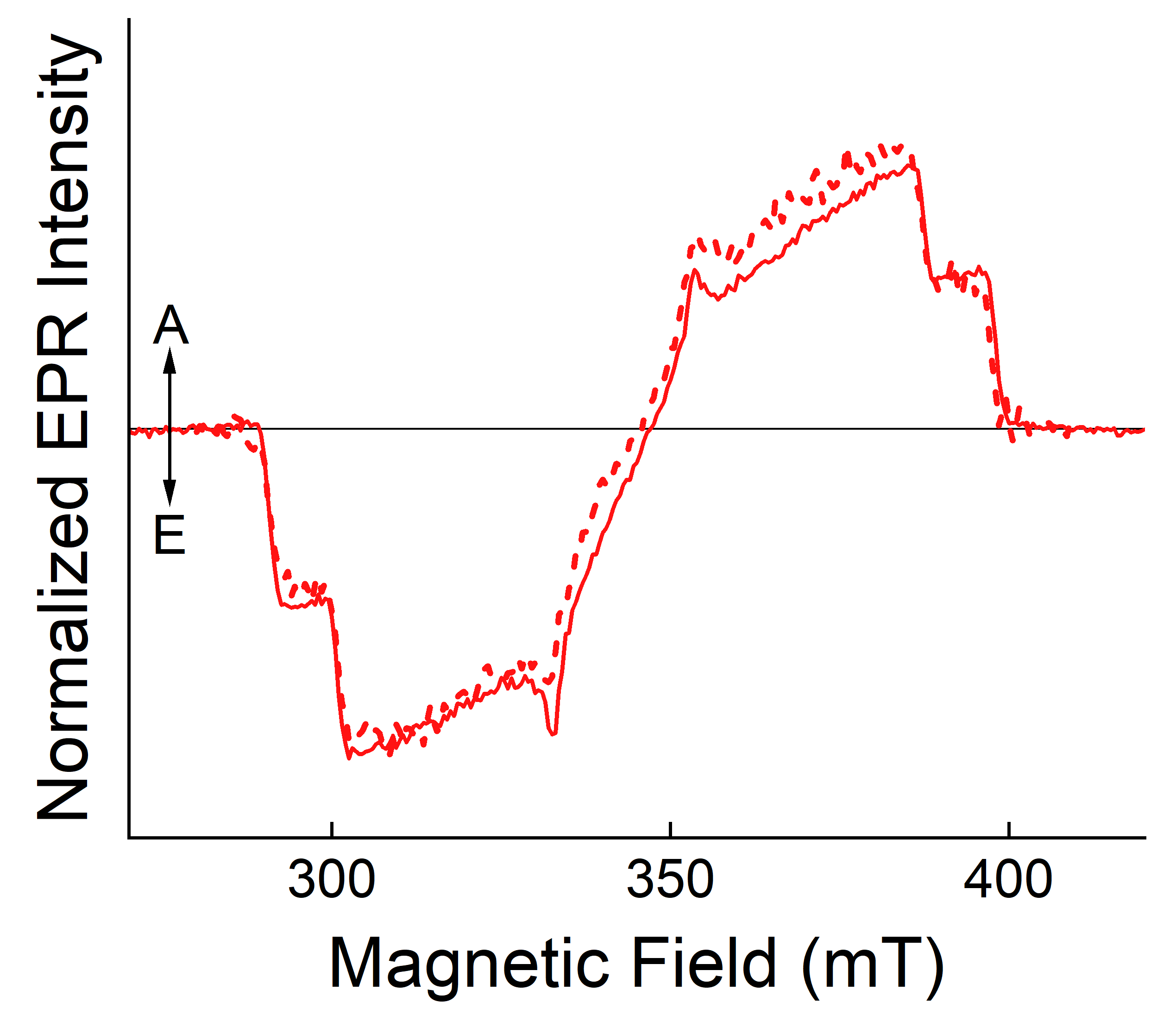

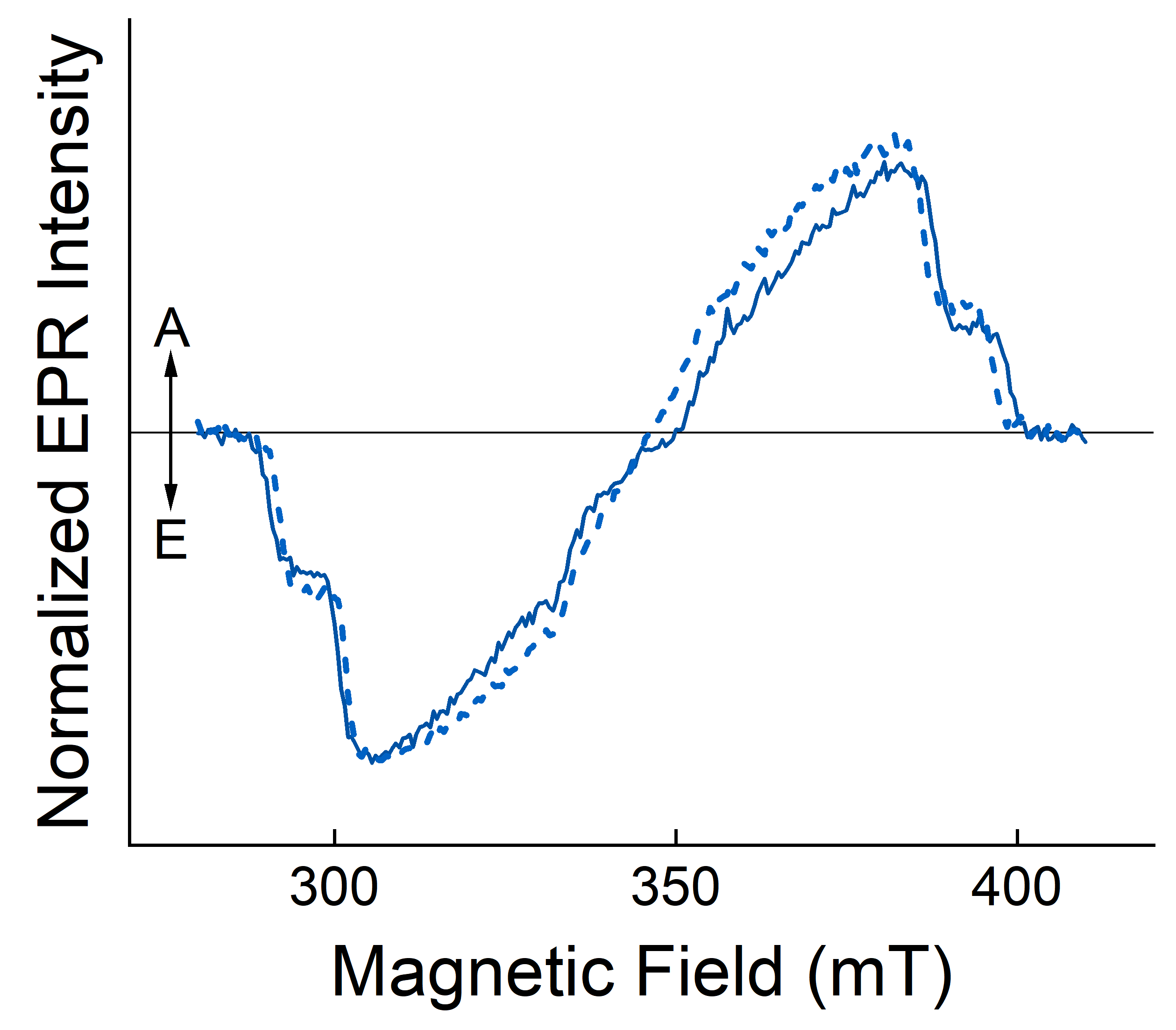


a)

b)

**Figure S108** X-band isotropic TR-EPR spectra of the triplet state of : a) **AsOMe** in Tol/MeTHF 1:1 (solid line) and in EtOH/MeOH 3:2 (dashed line), b) **AsNMe_2_** in Tol/DCM 1:1 (solid line) and in EtOH/MeOH 3:2 (dashed line) at 80 K after photoexcitation at 532 nm.


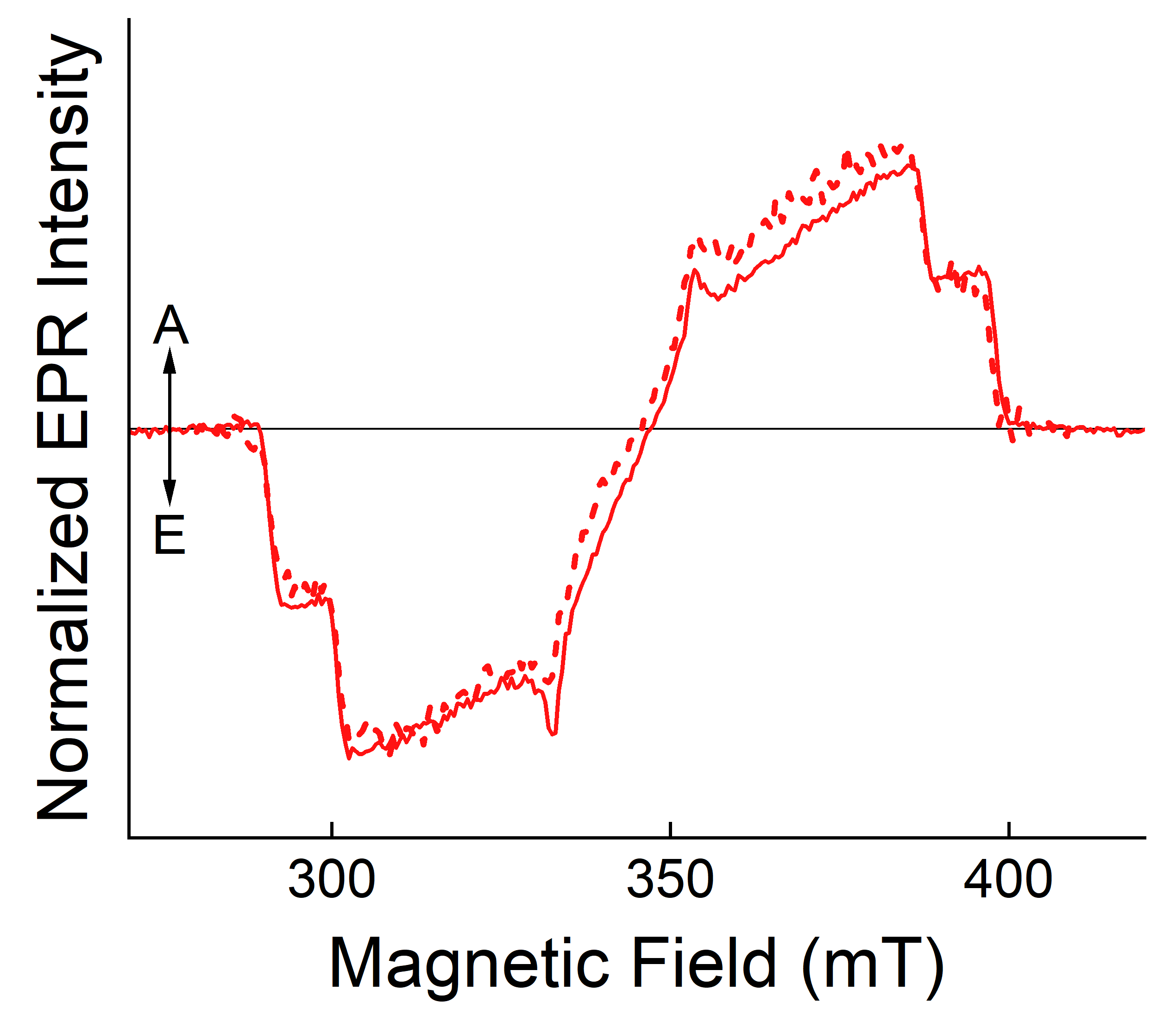

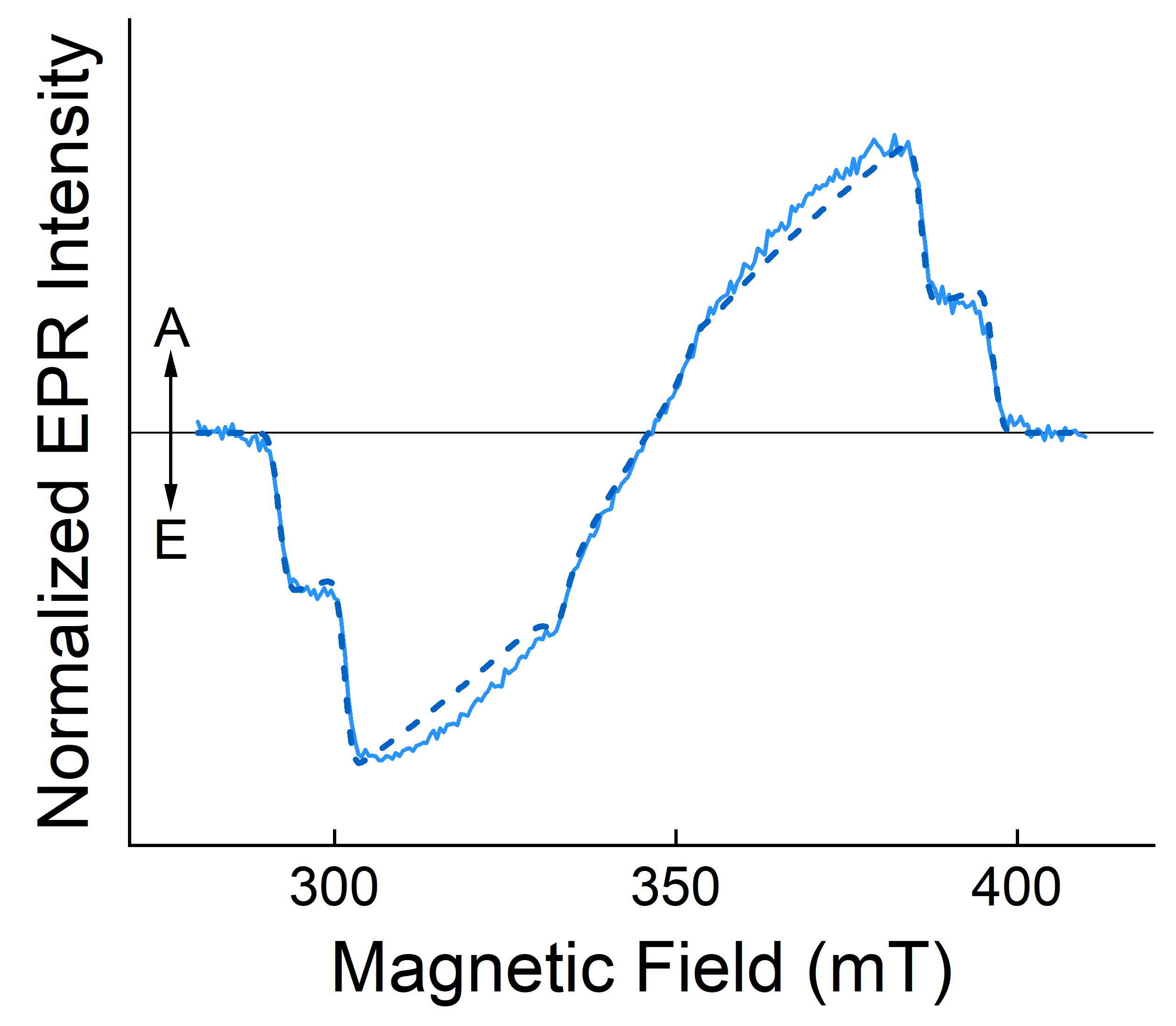


a)

b)


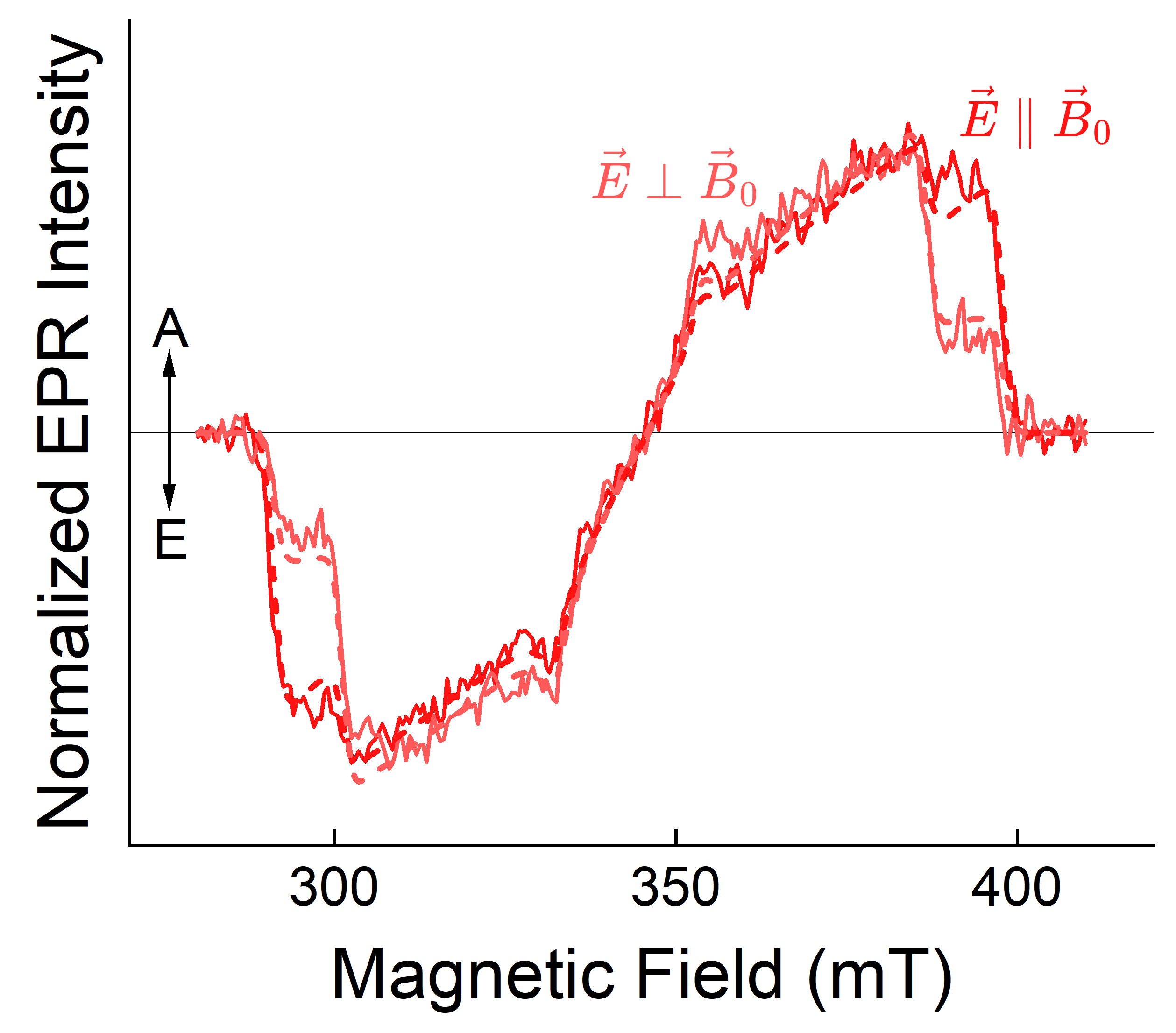

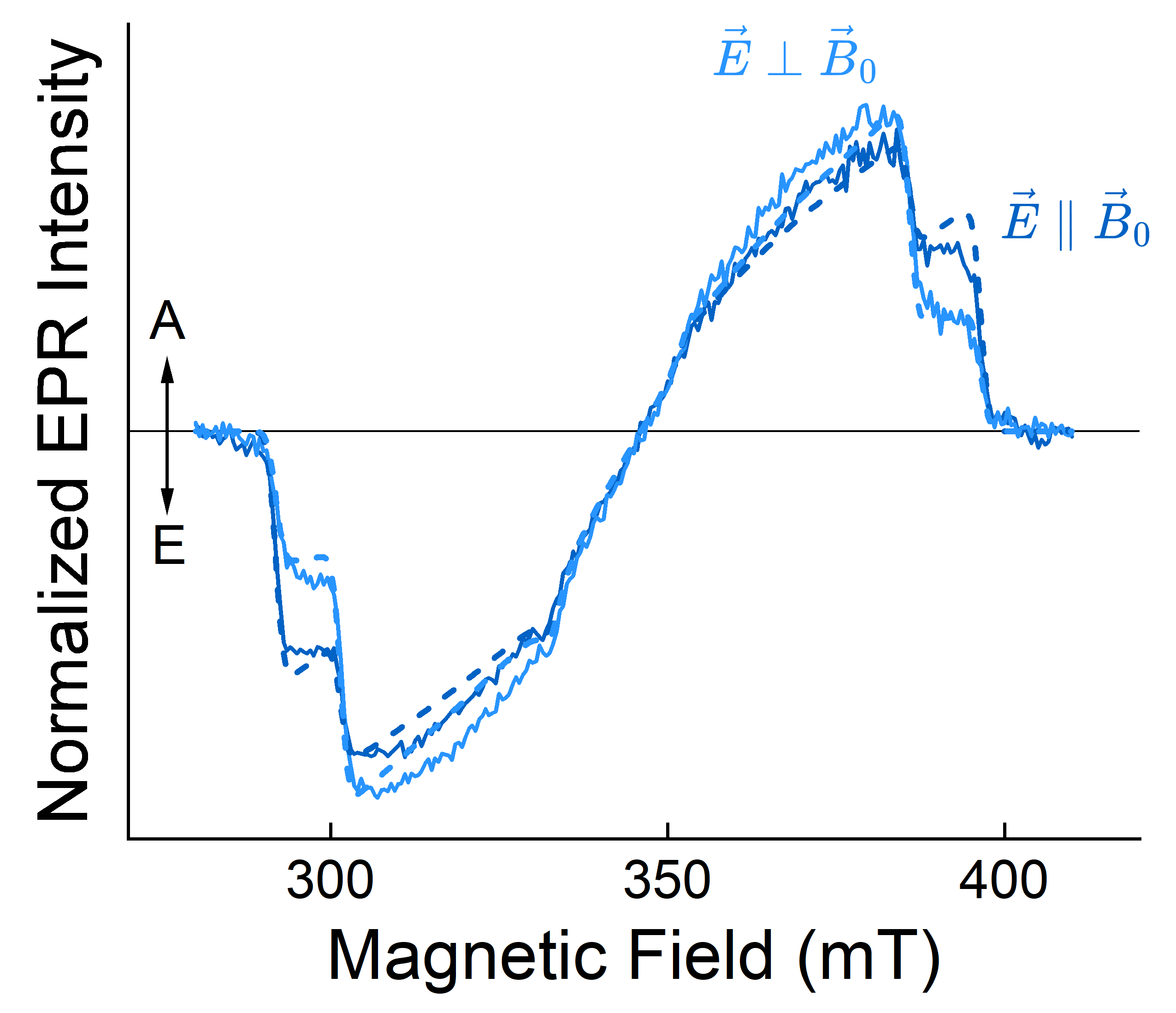


c)

d)

**Figure S109** Top: X-band isotropic TR-EPR spectra (solid line) and simulations (dashed line) of the triplet state of : a) **AsOMe** and b) **AsNMe_2_** in EtOH/MeOH 3:2 at 80 K after photoexcitation at 532 nm. Bottom: X-band photoselected TR-EPR spectra of the triplet state of: a) **AsOMe**, b) **AsNMe_2_**, in EtOH/MeOH 3:2 using linearly polarized light parallel (dark color) and perpendicular (light color) to the external magnetic field. Simulations of the MPS effect are also shown (dashed lines).

**Table S27** Simulation parameters of the TR-EPR spectra in frozen solution: ZFS parameters ($D\pm8$ MHz and $E\pm5$ MHz), relative population of the triplet sublevels ($p_{i}\pm0.02$), relative triplet sublevel time decay ($\frac{1}{k_{i}}\pm0.5 \mu s$), orientation of the TDM in the ZFS frame ($\omega\pm3^{\circ}$ and $\varphi\pm3^{\circ}$) as described in Toffoletti *et al.*^[18]^, and percentage of the isotropic contribution to the MPS effect ($c\pm2\%$) due to light scattering and other depolarization processes.

| **Compound** | $\boldsymbol{D}$ **(MHz)** | $\boldsymbol{E}$ **(MHz)** | $\boldsymbol{p}_{\boldsymbol{x}}\boldsymbol{:}\boldsymbol{p}_{\boldsymbol{y}}\boldsymbol{:}\boldsymbol{p}_{\boldsymbol{z}}$ | $\frac{\boldsymbol{1}}{\boldsymbol{k}_{\boldsymbol{x}}}\boldsymbol{:}\frac{\boldsymbol{1}}{\boldsymbol{k}_{\boldsymbol{y}}}\boldsymbol{:}\frac{\boldsymbol{1}}{\boldsymbol{k}_{\boldsymbol{z}}} \mathbf{(}\boldsymbol{\mu s)}$ | $\boldsymbol{(\omega,\varphi)}$ | **c (%)** |
| --- | --- | --- | --- | --- | --- | --- |
| **AsOMe** | -1495 | -304 | 0.29:0.26:0.45 | 13.3:16.1:10 | (11°, 4°) | 34 |
| **AsNMe_2_** | -1504 | -306 | 0.32:0.14:0.54 | 9.9:32:7.1 | (31°, 2°) | 32 |
| **TPh** | -1392 | -262 | 0.31:0.15:0.54 | 8.8:28:6.2 | (29°, 2°) | 20 |
| **TPhOMe** | -1384 | -260 | 0.34:0.12:0.54 | 7.3:23.3:5.2 | (43°, 10°) | 8 |
| **AsOMe**  **(EtOH/MeOH 3:2)** | -1488 | -298 | 0.28:0.18:0.54 | --- | (28°, 1°) | 30 |
| **AsNMe_2_**  **(EtOH/MeOH 3:2)** | -1459 | -297 | 0.30:0.16:0.54 | --- | (30°, 2°) | 35 |

## **TR-EPR in film**


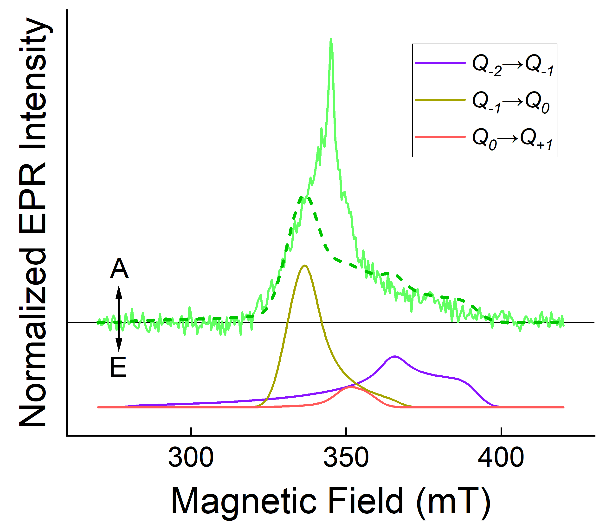

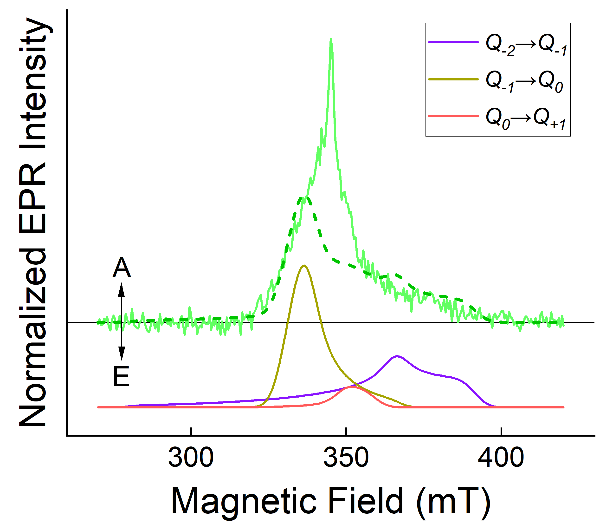


b)

a)


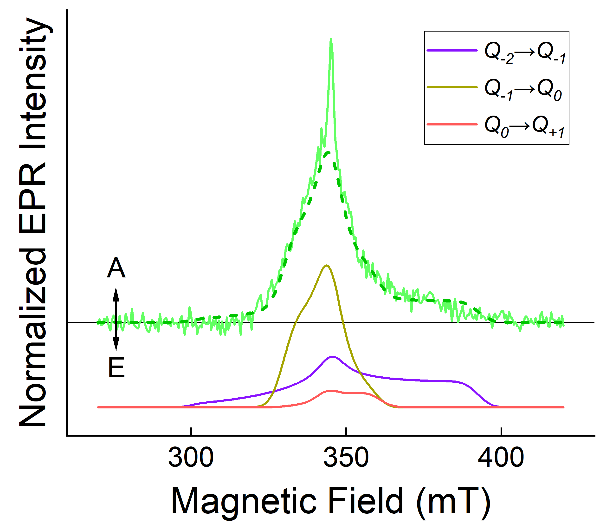

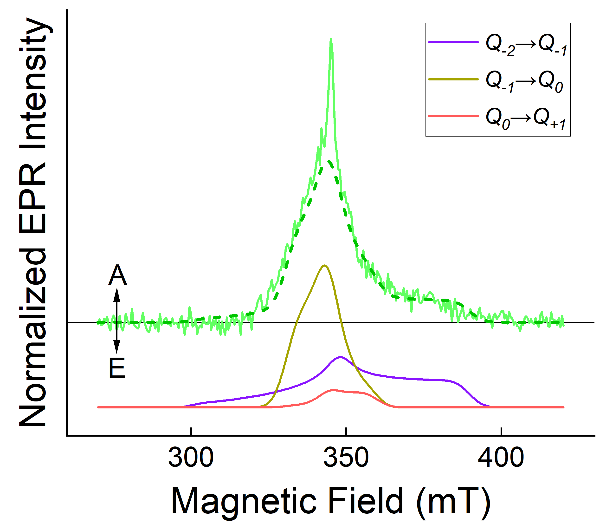

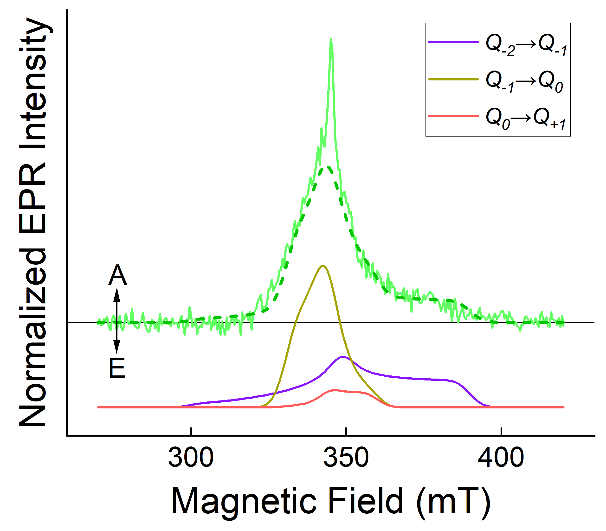

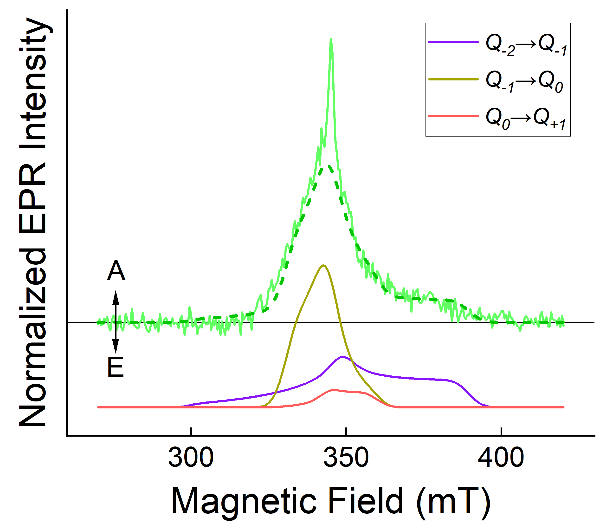


f)

e)

d)

c)

**Figure S110** Effect of the relative orientation of the two triplets on the simulation (dashed line) of the X-band TR-EPR spectrum of the quintet state of **TPhOMe** (solid line) at DAF=0.6 µs, different values of the Euler angles defining the relative orientation of the ZFS frames of the second triplet are used: a) collinear ZFS frames, b) $(\alpha,\beta,\gamma)=(20^{\circ},0^{\circ},0^{\circ})$, c) $(\alpha,\beta,\gamma)=(0^{\circ},60^{\circ},0^{\circ})$, d) $(\alpha,\beta,\gamma)=(20^{\circ},60^{\circ},0^{\circ})$, e) $(\alpha,\beta,\gamma)=(0^{\circ},60^{\circ},30^{\circ})$, f) $(\alpha,\beta,\gamma)=(20^{\circ},60^{\circ},20^{\circ})$. The contribution of the EPR transitions in the quintet state is also shown. The central radical signal has not been simulated.


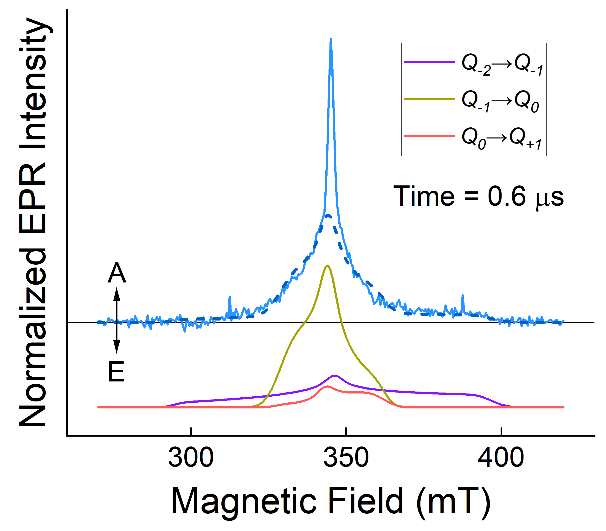

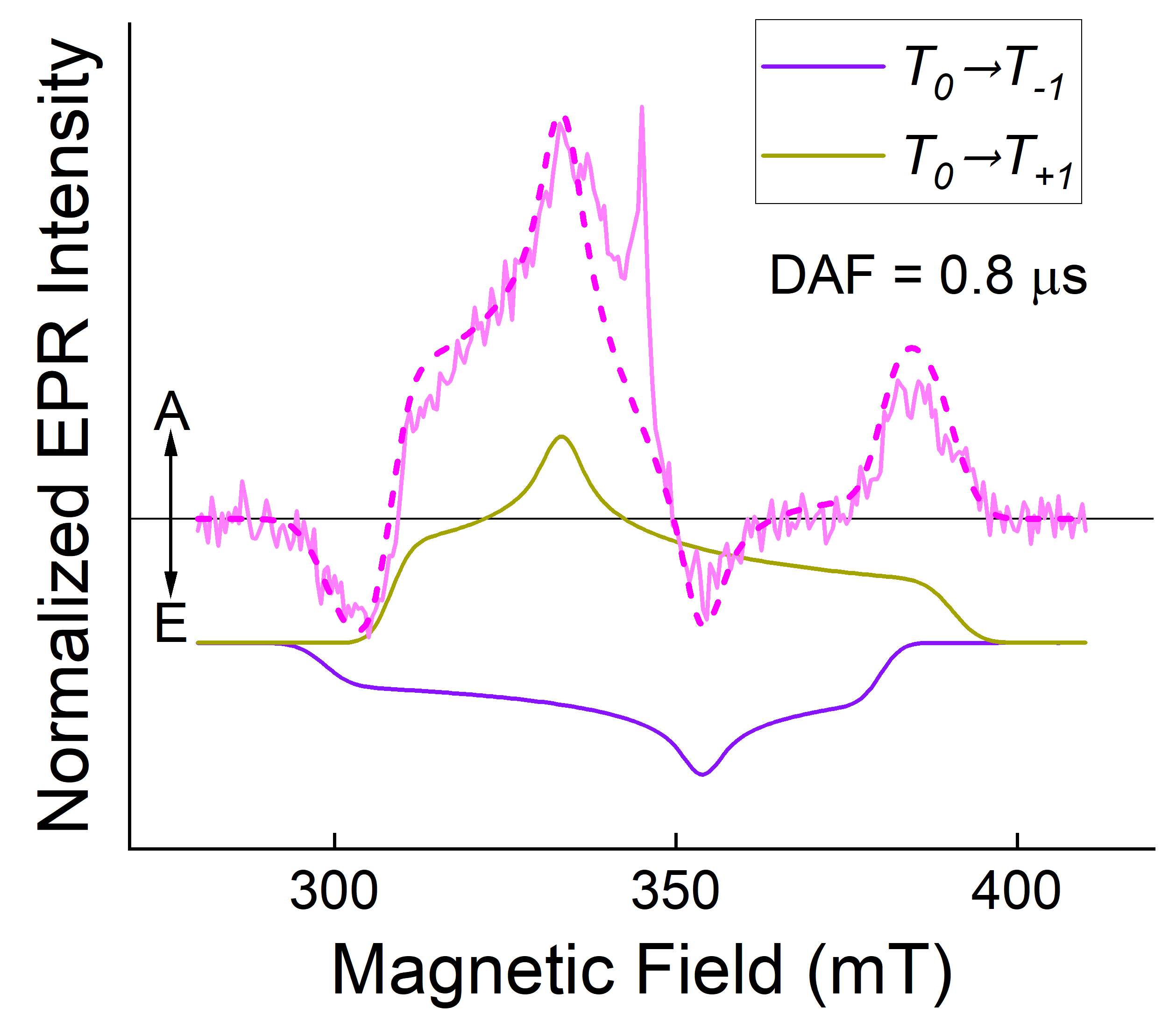


b)

a)


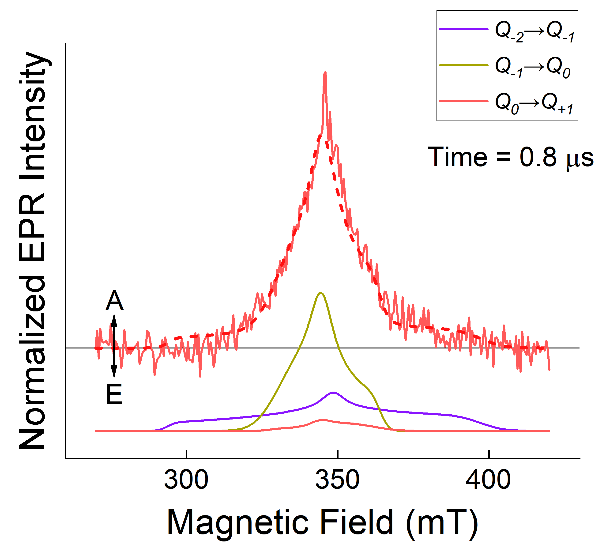


c)

**Figure S111** X-band TR-EPR spectra (full line) in film at 80 K and corresponding simulations (dashed line) of: a) the correlated triplet pair deriving from SF of **AsNMe_2_**, b) the uncorrelated triplet state deriving from SF of **TPh** after photoexcitation at 532 nm and c) the correlated triplet pair deriving from SF of **AsOMe** after photoexcitation at 450 nm. The contribution of the EPR transitions in the quintet state or triplet state are also shown. The central radical signal has not been simulated.

**Table S28** Simulation parameters for the triplet pair in the strong exchange coupling regime as detected in film for some of the compounds and in specific condition (as reported in the first column of the Table): ZFS parameters of triplet state ($D_{T}$ and $E_{T}$ in MHz from simulations of the TR-EPR spectra in frozen solution), relative population of the quintet sublevels ($p_{i}\pm0.03$), Euler angles ($\alpha,\beta,\gamma\pm3^{\circ}$) defining the relative orientation of the ZFS tensors of the second triplet and broadening of the spectral lines (HStrain in Easyspin). For all the systems $J=20$ GHz and $D_{AB}$=150 MHz are fixed parameters.

| **Compound** | $\boldsymbol{D}_{\boldsymbol{T}}$ **(MHz)** | $\boldsymbol{E}_{\boldsymbol{T}}$ **(MHz)** | $\boldsymbol{p}_{\boldsymbol{-}\boldsymbol{2}}\boldsymbol{:}\boldsymbol{p}_{\boldsymbol{-}\boldsymbol{1}}\boldsymbol{:}\boldsymbol{p}_{\boldsymbol{0}}\boldsymbol{:}\boldsymbol{p}_{\boldsymbol{+}\boldsymbol{1}}\boldsymbol{:}\boldsymbol{p}_{\boldsymbol{+2}}$ | **(**$\boldsymbol{\alpha,\beta,\gamma)}$ | **HStrain (MHz)** |
| --- | --- | --- | --- | --- | --- |
| **AsOMe (450 nm)** | -1495 | -304 | 0.50:0.26:0.24:0:0 | (0°,55°,0°) | (143, 289, 129) |
| **AsNMe_2_** | -1504 | -306 | 0.48:0.28:0.24:0:0 | (0°,58°,0°) | (119, 232, 177) |
| **AsNMe_2_ (250 K)** | -1504 | -306 | 0.48:0.28:0.24:0:0 | (0°,58°,0°) | (204, 211, 223) |
| **TPhOMe** | -1384 | -260 | 0.53:0.25:0.22:0:0 | (0°,62°,0°) | (183, 226, 173) |

**Table S29** Simulation parameters for the uncorrelated triplet state as detected in film for some of the compounds and in specific condition (as reported in the first column of the Table): ZFS parameters of the free triplets in film ($D_{T}\pm15$ MHz and $E_{T}\pm10$ MHz), relative population of the triplet sublevels ($p_{i}\pm0.03$) and broadening of the spectral lines (HStrain in Easyspin).

| **Compound** | $\boldsymbol{D}_{\boldsymbol{T}}$ **(MHz)** | $\boldsymbol{E}_{\boldsymbol{T}}$ **(MHz)** | $\boldsymbol{p}_{\boldsymbol{-}\boldsymbol{1}}\boldsymbol{:}\boldsymbol{p}_{\boldsymbol{0}}\boldsymbol{:}\boldsymbol{p}_{\boldsymbol{+}\boldsymbol{1}}$ | **HStrain (MHz)** |
| --- | --- | --- | --- | --- |
| **AsNMe_2_ (600 nm)** | -1393 | -247 | 0:1:0 | (137, 192, 121) |
| **TPh (532 nm) 80 K** | -1284 | -238 | 0.26:0.74:0 | (122, 147, 198) |
| **TPh (532 nm) 250 K** | -1284 | -227 | 0.28:0.72:0 | (112, 316, 229) |
| **TPh (600 nm)** | -1277 | -242 | 0.2:0.8:0 | (160, 300, 200) |


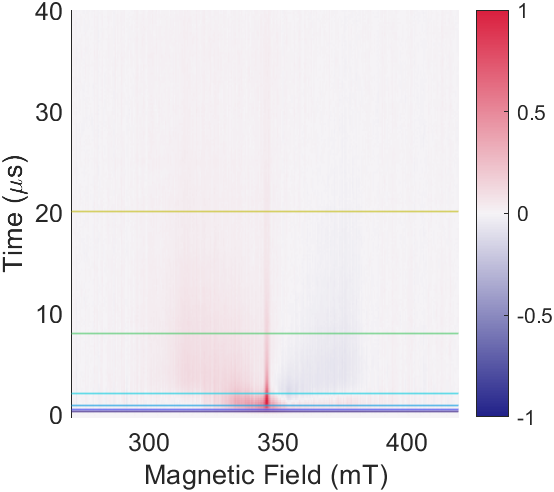

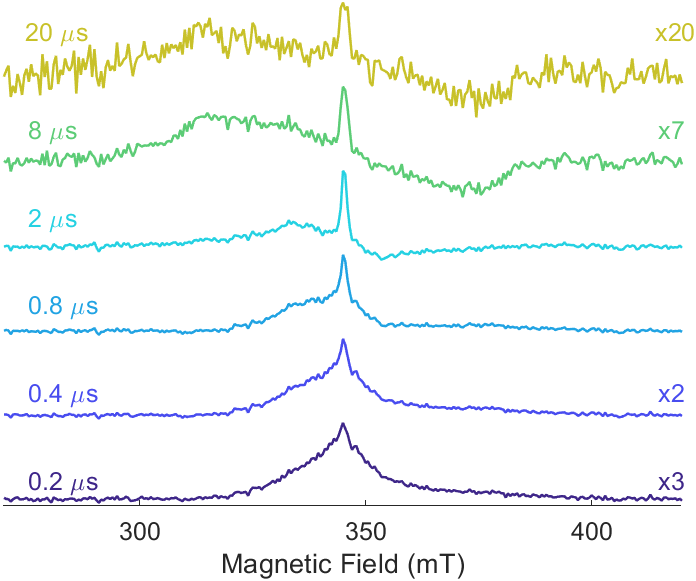


**Figure S112:** Experimental contour plot (left) and slices of the X-band isotropic TR-EPR data at selected DAF (right) for **TPhOMe** thin film at 80 K, after photoexcitation with 5 mJ/pulse at 532 nm.


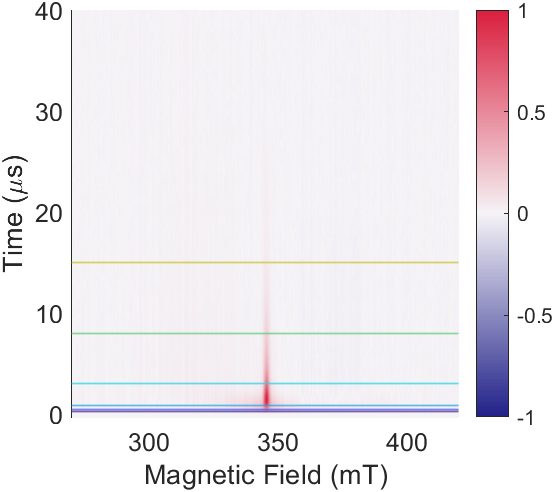

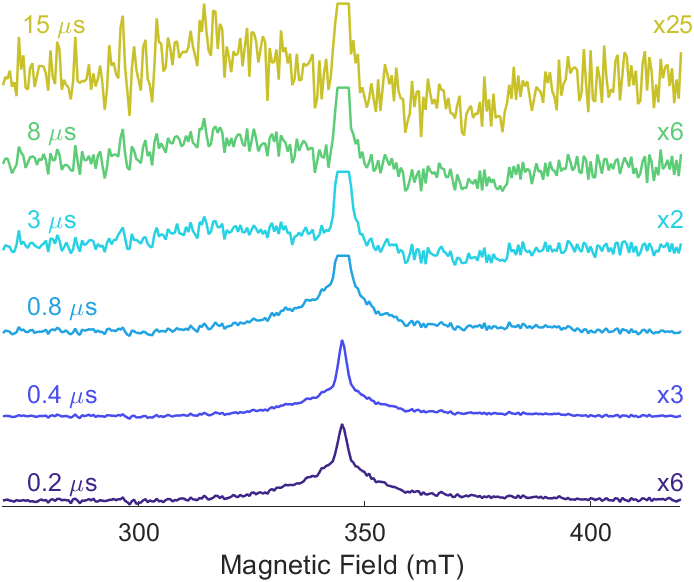


**Figure S113:** Experimental contour plot (left) and slices of the X-band isotropic TR-EPR data at selected DAF (right) for **AsNMe_2_** thin film at 80 K, after photoexcitation with 5 mJ/pulse at 532 nm. The central radical feature has been cut for proper visualization of the TR-EPR spectra at later times.


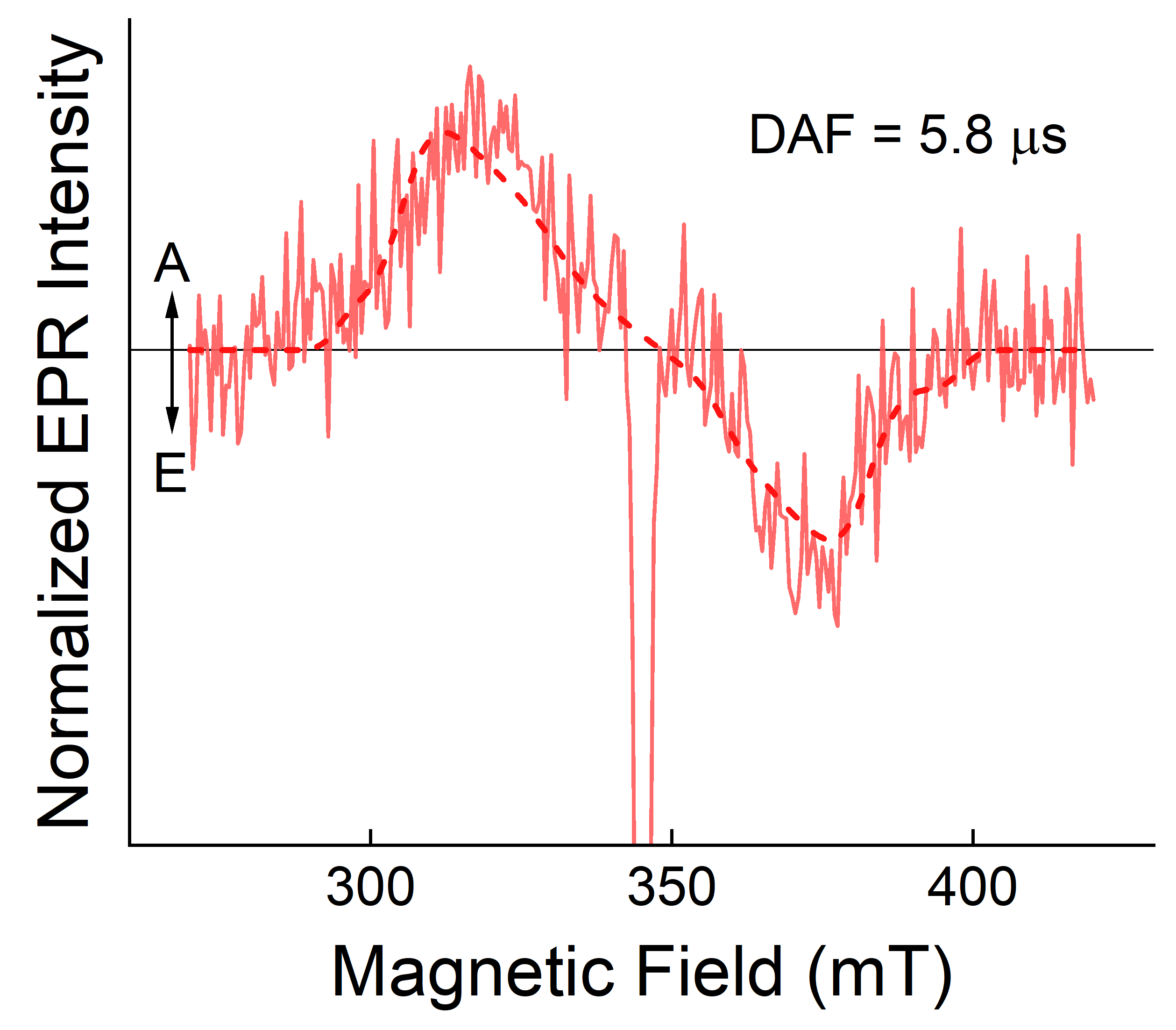

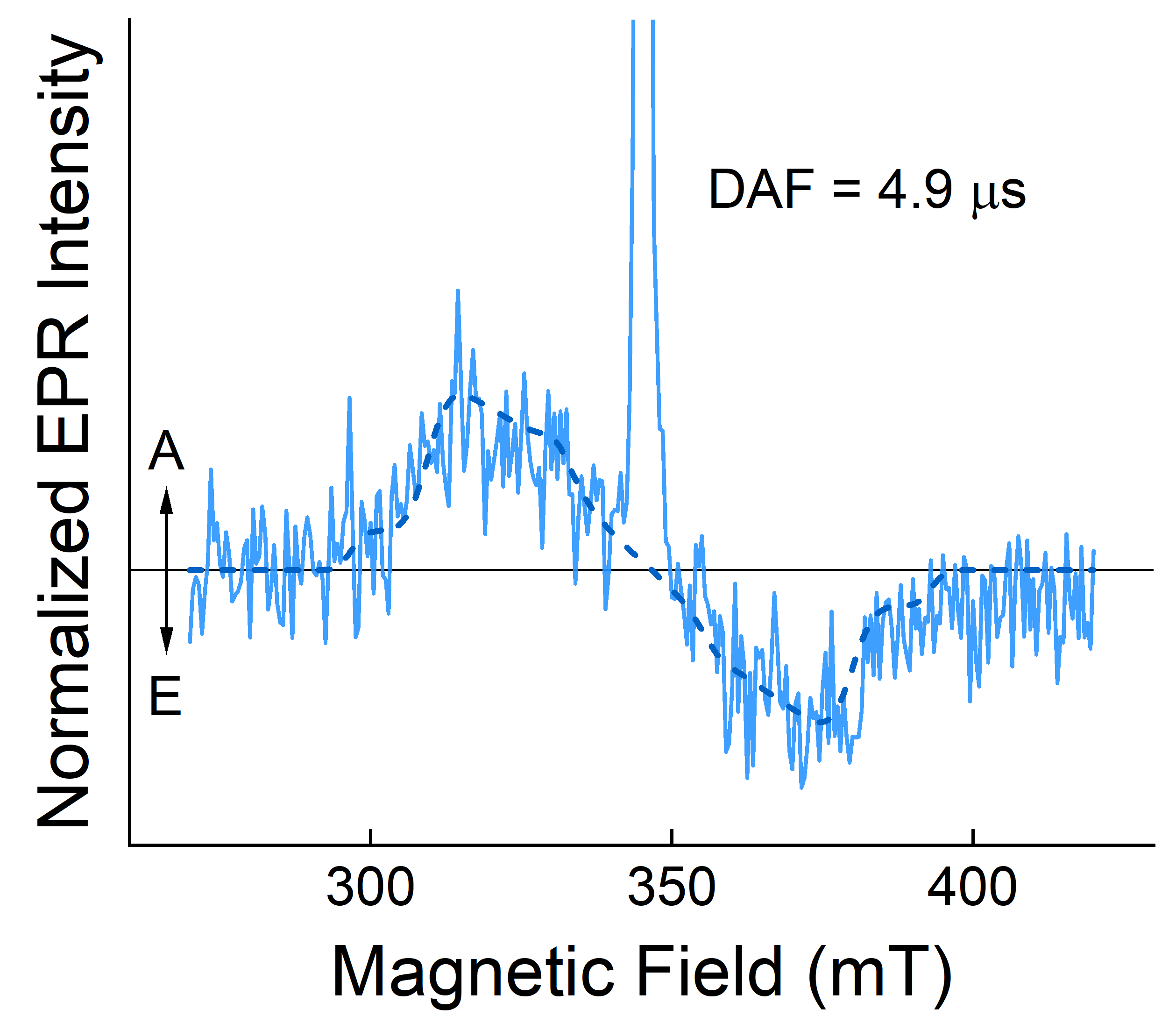


b)

d)

c)

a)


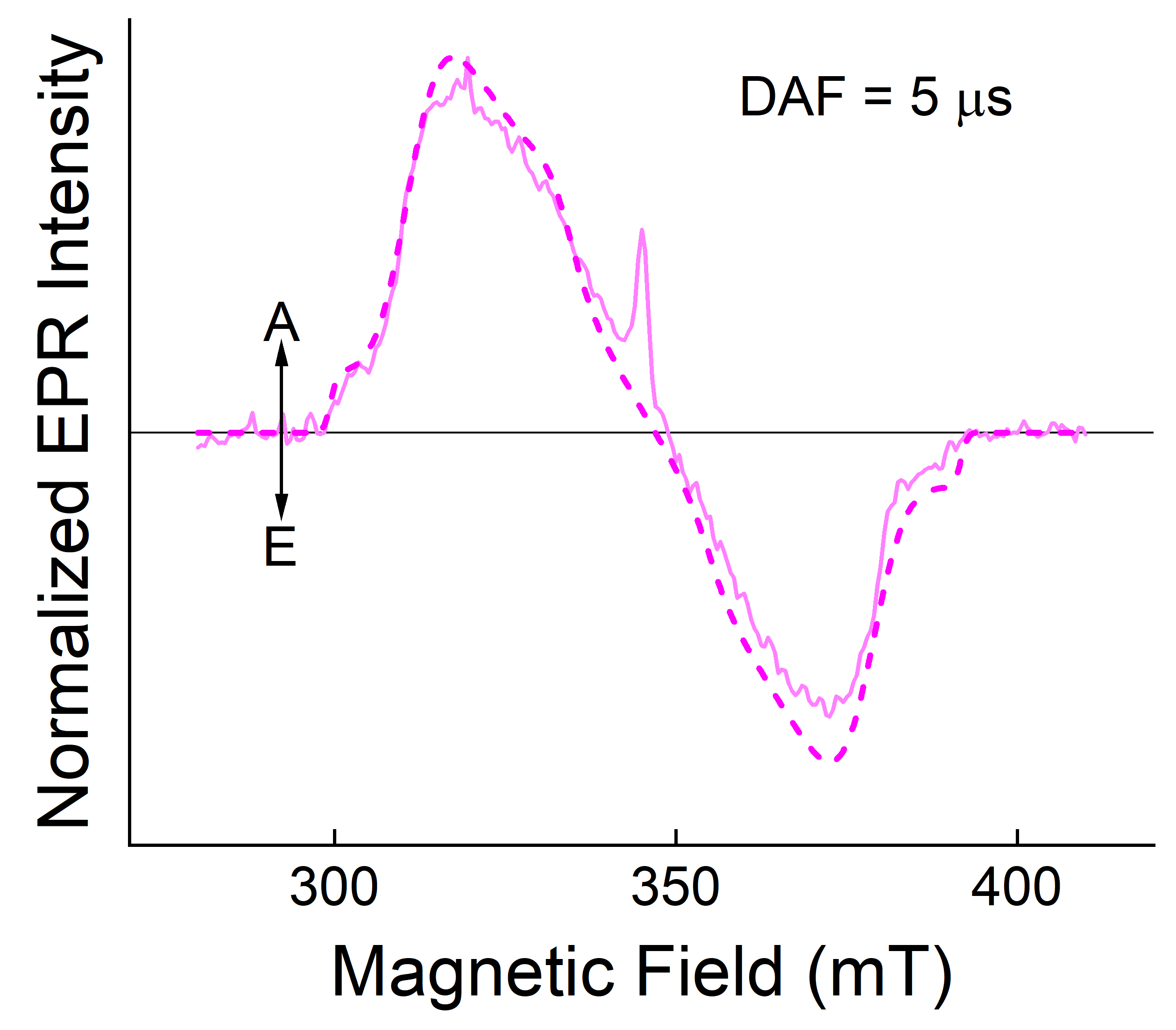

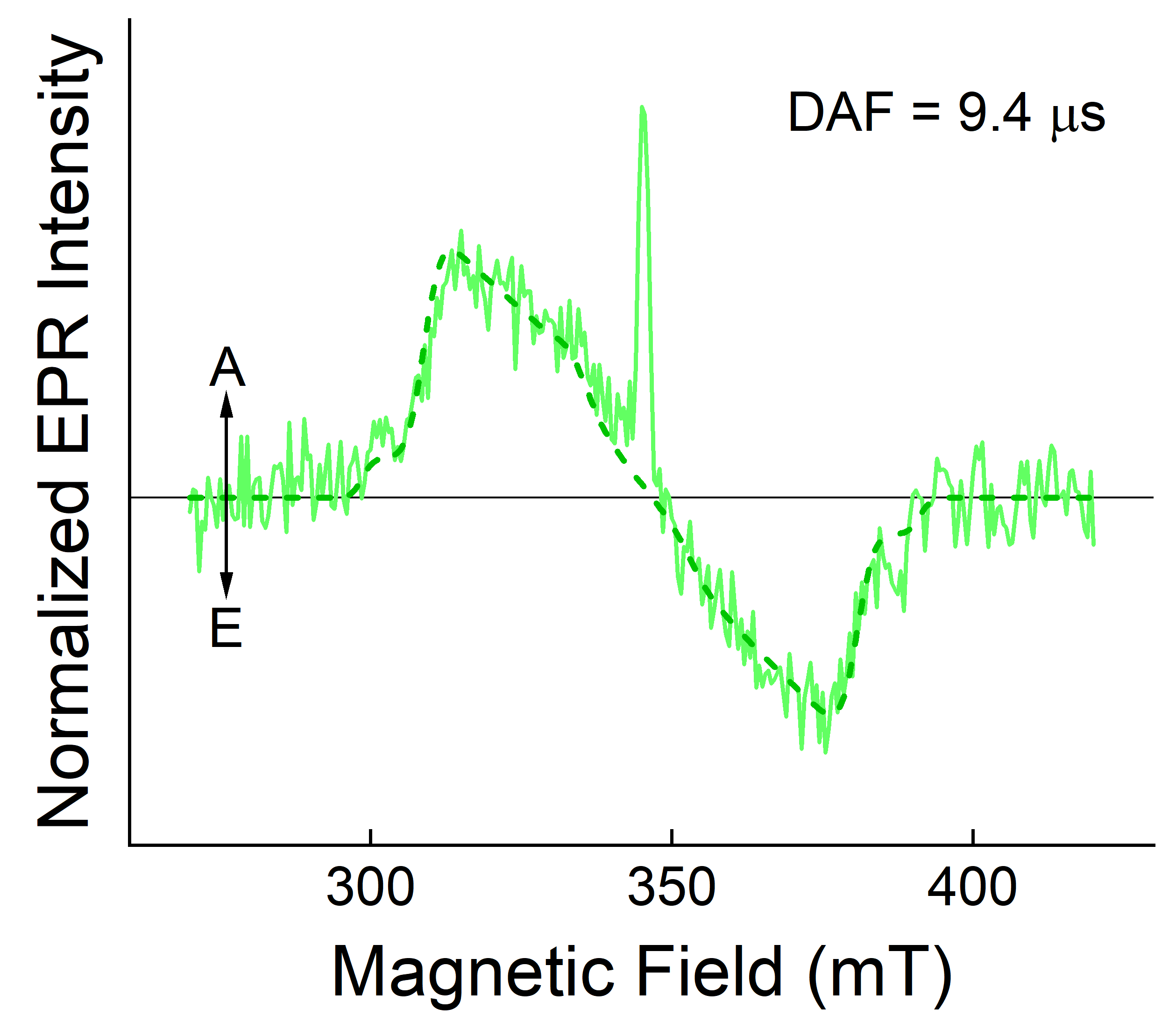


**Figure S114** X-band TR-EPR spectra (full line) in film at 80 K after photoexcitation at 532 nm at DAF as reported in the panels and corresponding triplet state simulations (dashed line) for: a) **AsOMe**, b) **AsNMe_2_**, c) **TPh**, d) **TPhOMe**. The central radical signal has not been simulated.


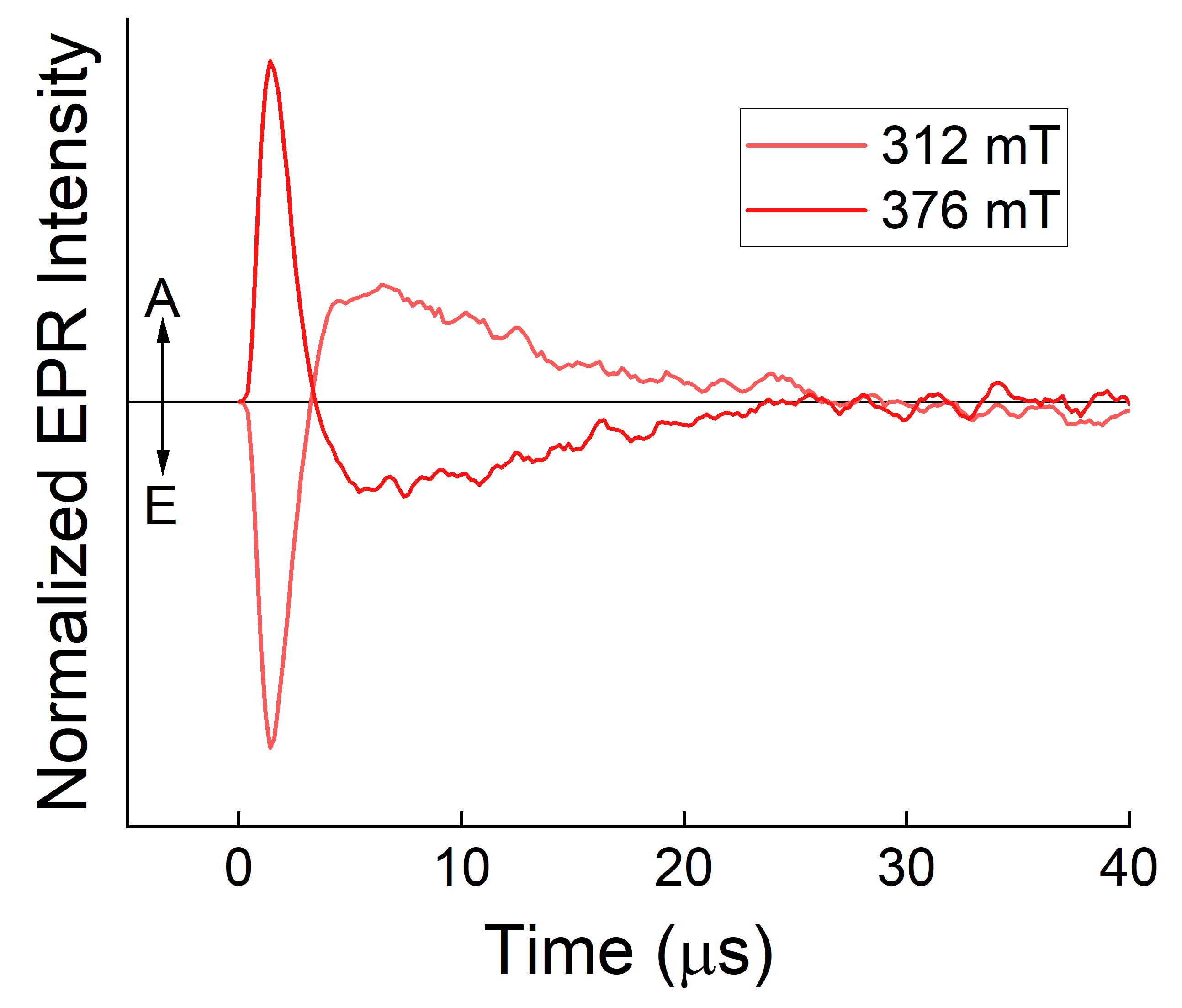

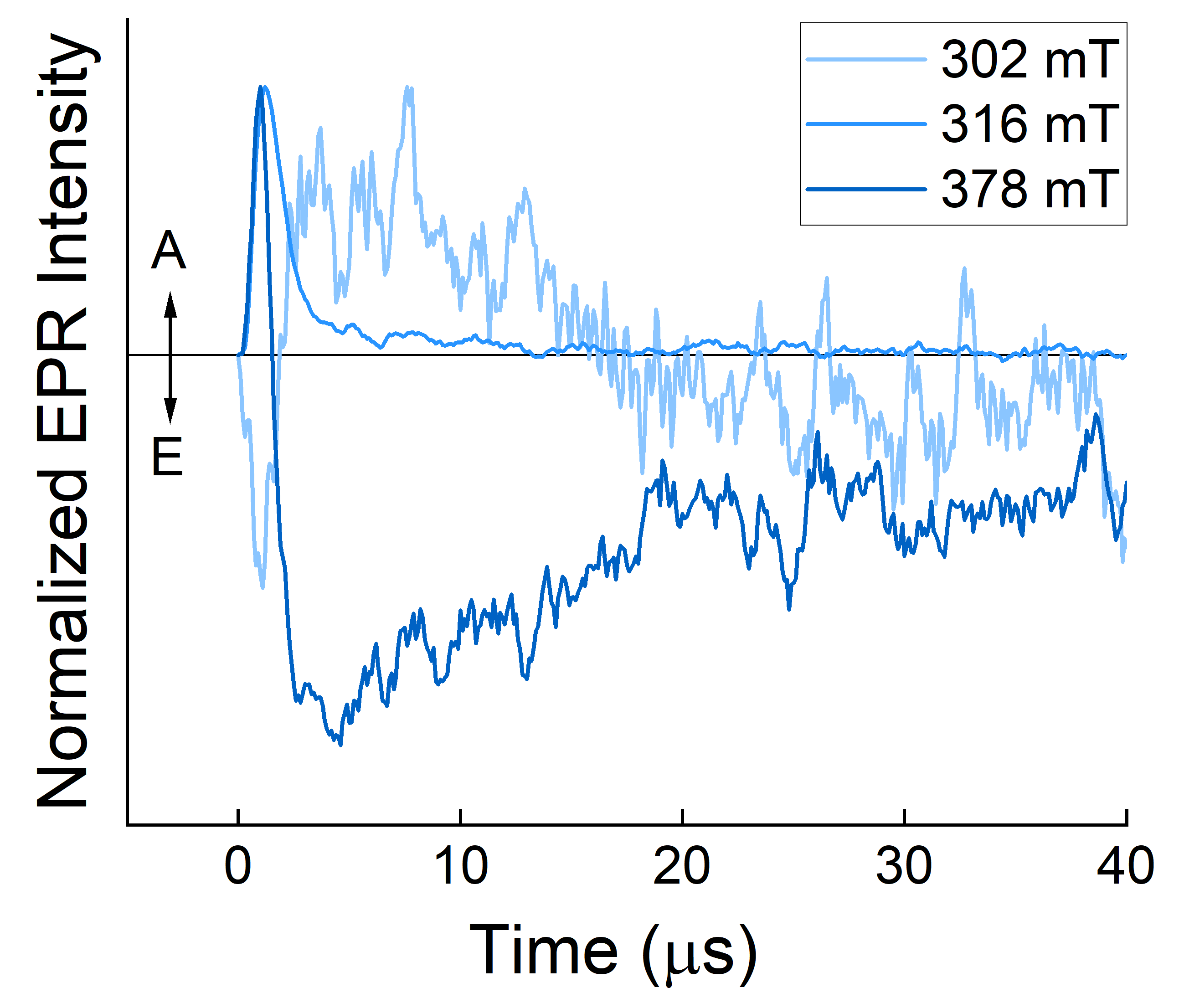


b)

d)

a)


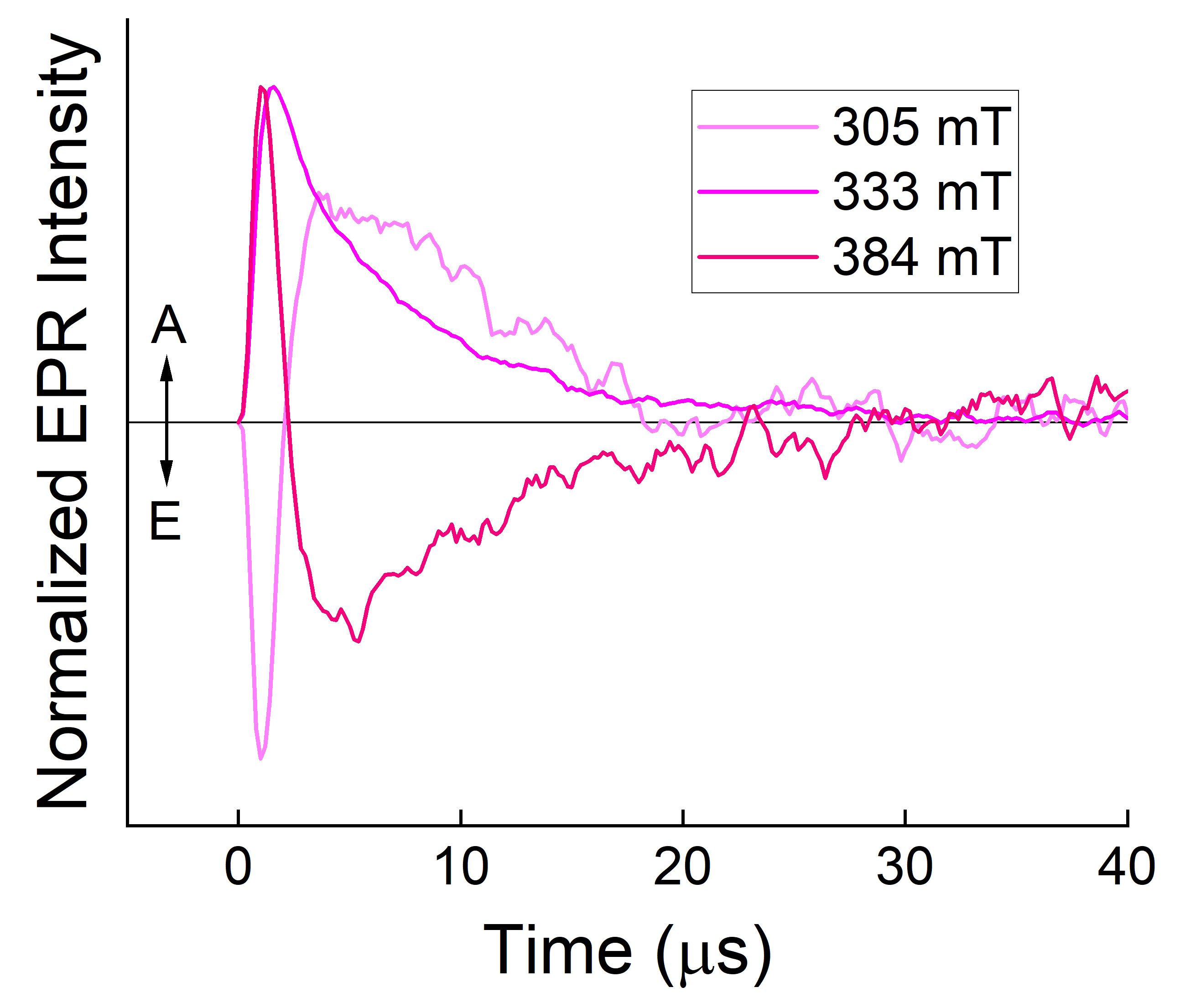

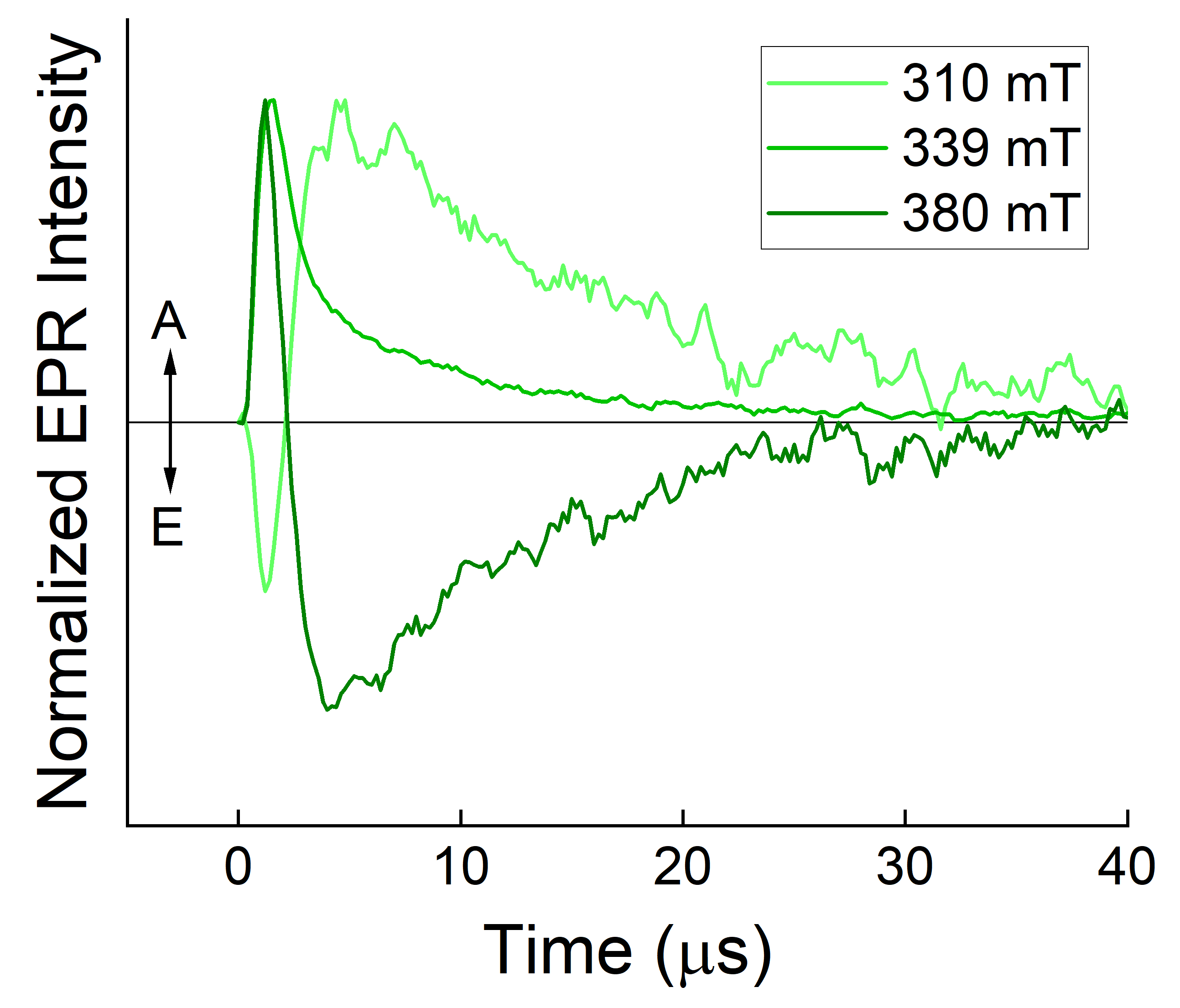


c)

**Figure S115** Time traces extracted from the 2D TR-EPR data in film at 80 K after photoexcitation at 532 nm for the following compounds: a) **AsOMe** b) **AsNMe_2_**, c) **TPh**, d) **TPhOMe**.

**Table S30** Simulation parameters of TR-EPR spectra in film at DAF>4 µs as reported in Fig. S112 for the four compounds under investigation: ZFS parameters ($D\pm15$ MHz and $E\pm10$ MHz), relative population of the triplet sublevels ($p_{i}\pm0.05$) and broadening of the spectral lines (HStrain in Easyspin).

| **Compound** | $\boldsymbol{D}$ **(MHz)** | $\boldsymbol{E}$ **(MHz)** | $\boldsymbol{p}_{\boldsymbol{X}}\boldsymbol{:}\boldsymbol{p}_{\boldsymbol{Y}}\boldsymbol{:}\boldsymbol{p}_{\boldsymbol{Z}}$ | **HStrain (MHz)** |
| --- | --- | --- | --- | --- |
| **AsOMe** | -1424 | -224 | 0.31:0.69:0 | (174, 247, 141) |
| **AsNMe_2_** | -1393 | -247 | 0.36:0.47:0.17 | (131, 183, 119) |
| **TPh** | -1294 | -204 | 0.35:0.47:0.18 | (144, 240, 20) |
| **TPhOMe** | -1301 | -237 | 0.34:0.39:0.27 | (137, 143, 96) |


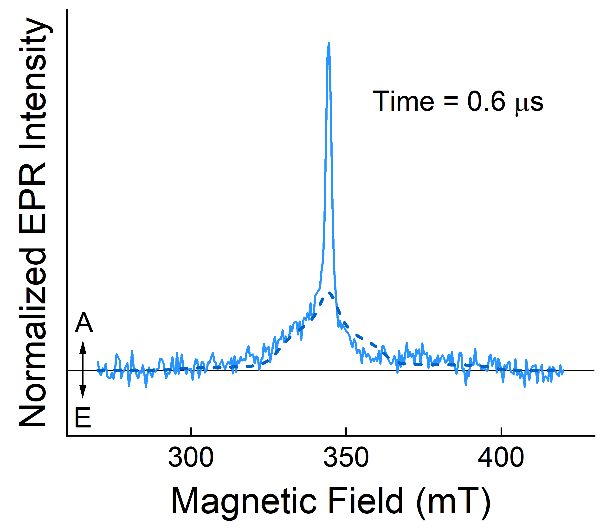

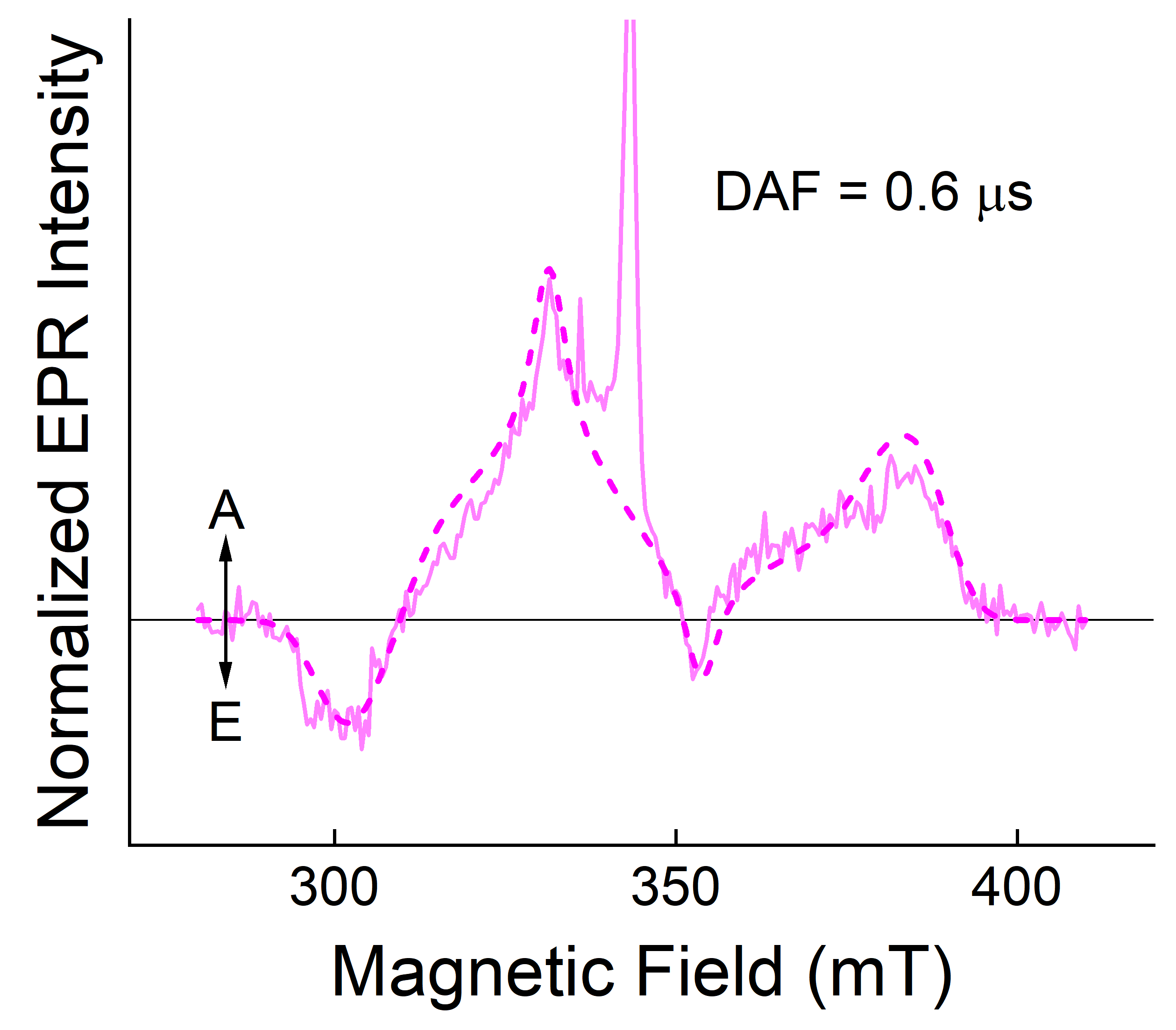


b)

a)

**Figure S116** X-band TR-EPR spectra (full line) and simulations (dashed line) in film at 250 K after photoexcitation at 532 nm of: a) the correlated triplet pair deriving from SF of **AsNMe_2_**, b) the uncorrelated triplet state deriving from SF of **TPh**. The central radical signal has not been simulated.


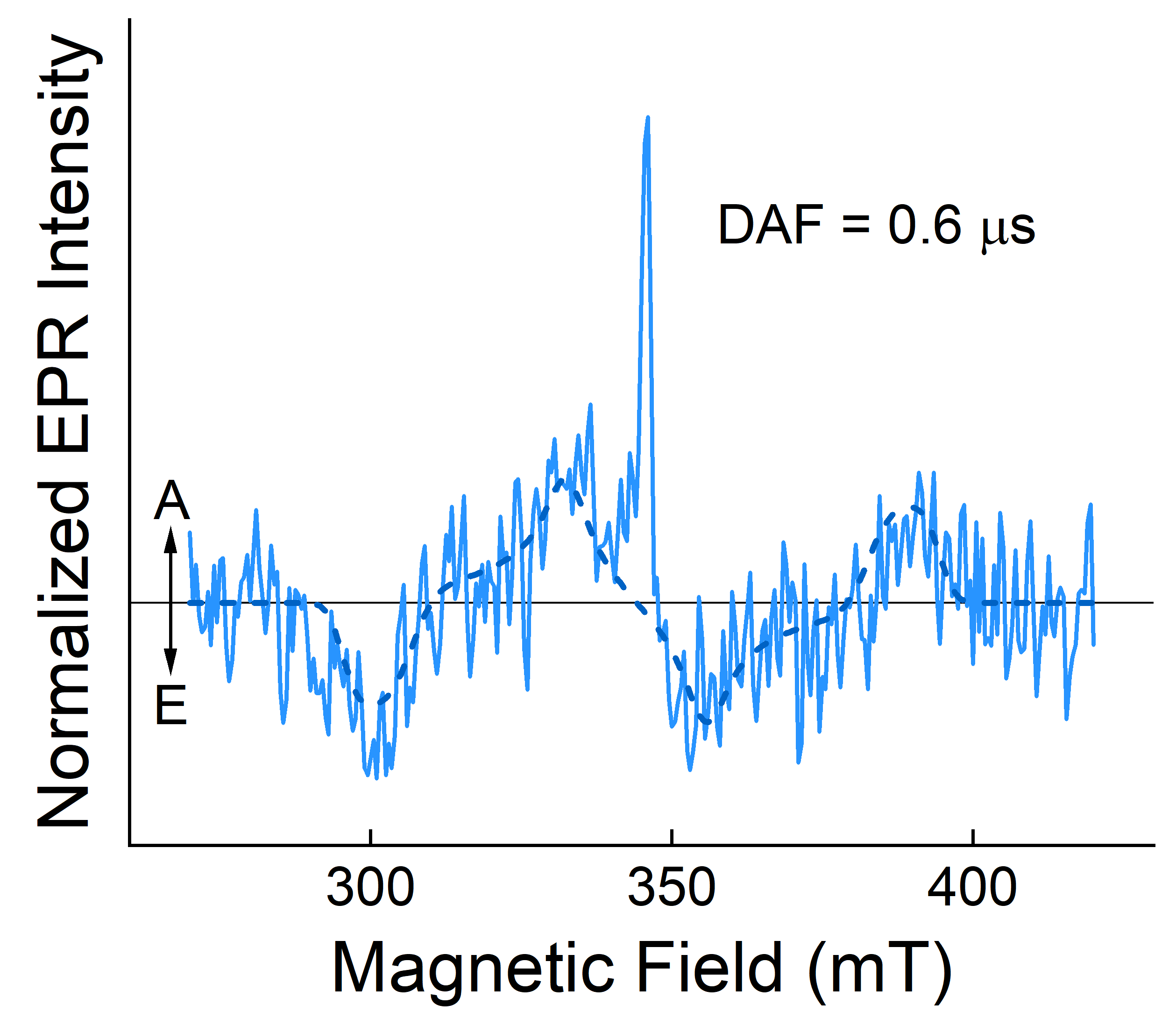

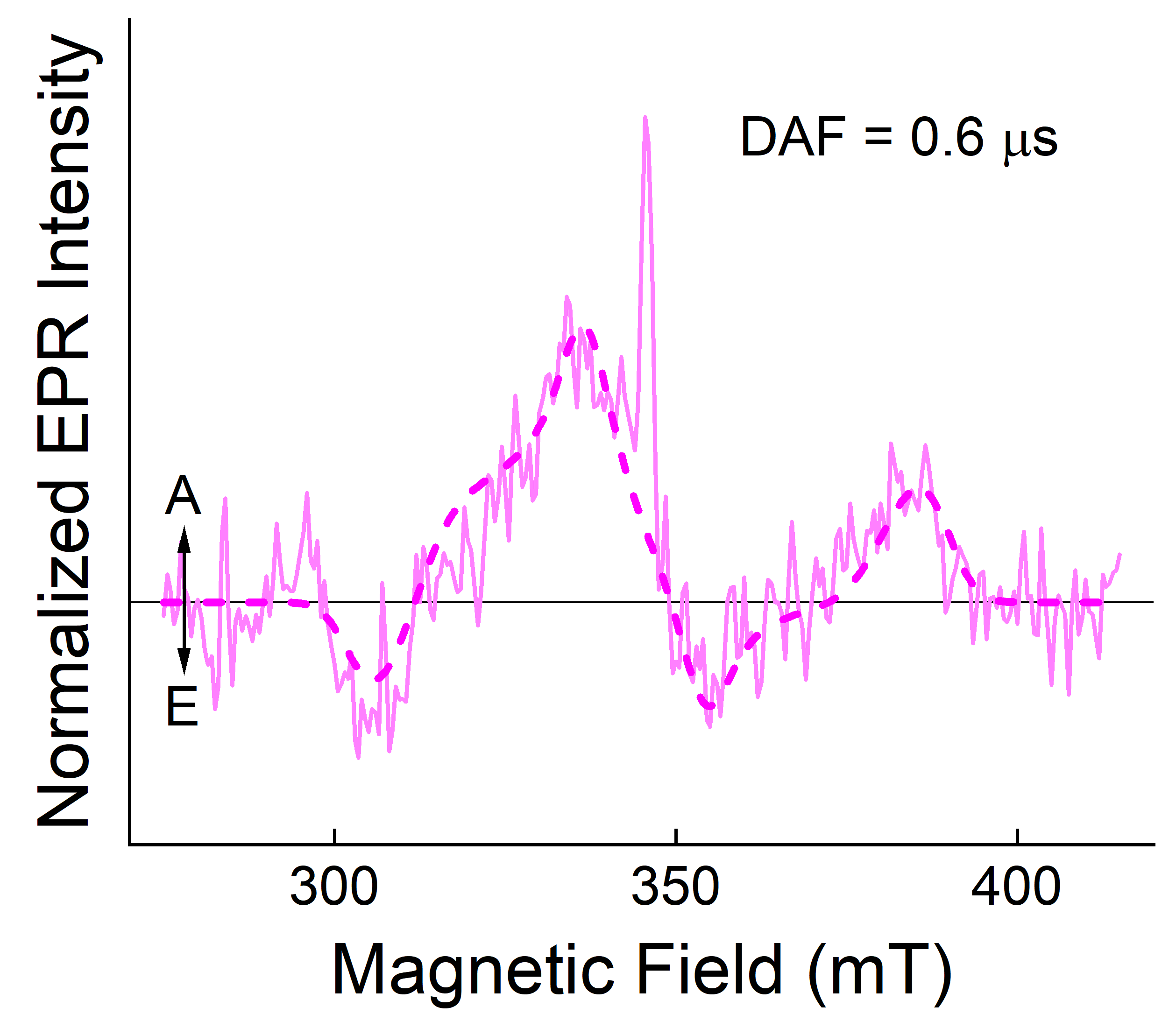


b)

a)

**Figure S117** X-band TR-EPR spectra (full line) in film at 80 K after photoexcitation at 600 nm and corresponding simulations (dashed line) of the uncorrelated triplet state deriving from SF for: a) **AsNMe_2_**, b) **TPh**. The central radical signal has not been simulated.

# **Scanning Electron Microscopy of Thin Films**


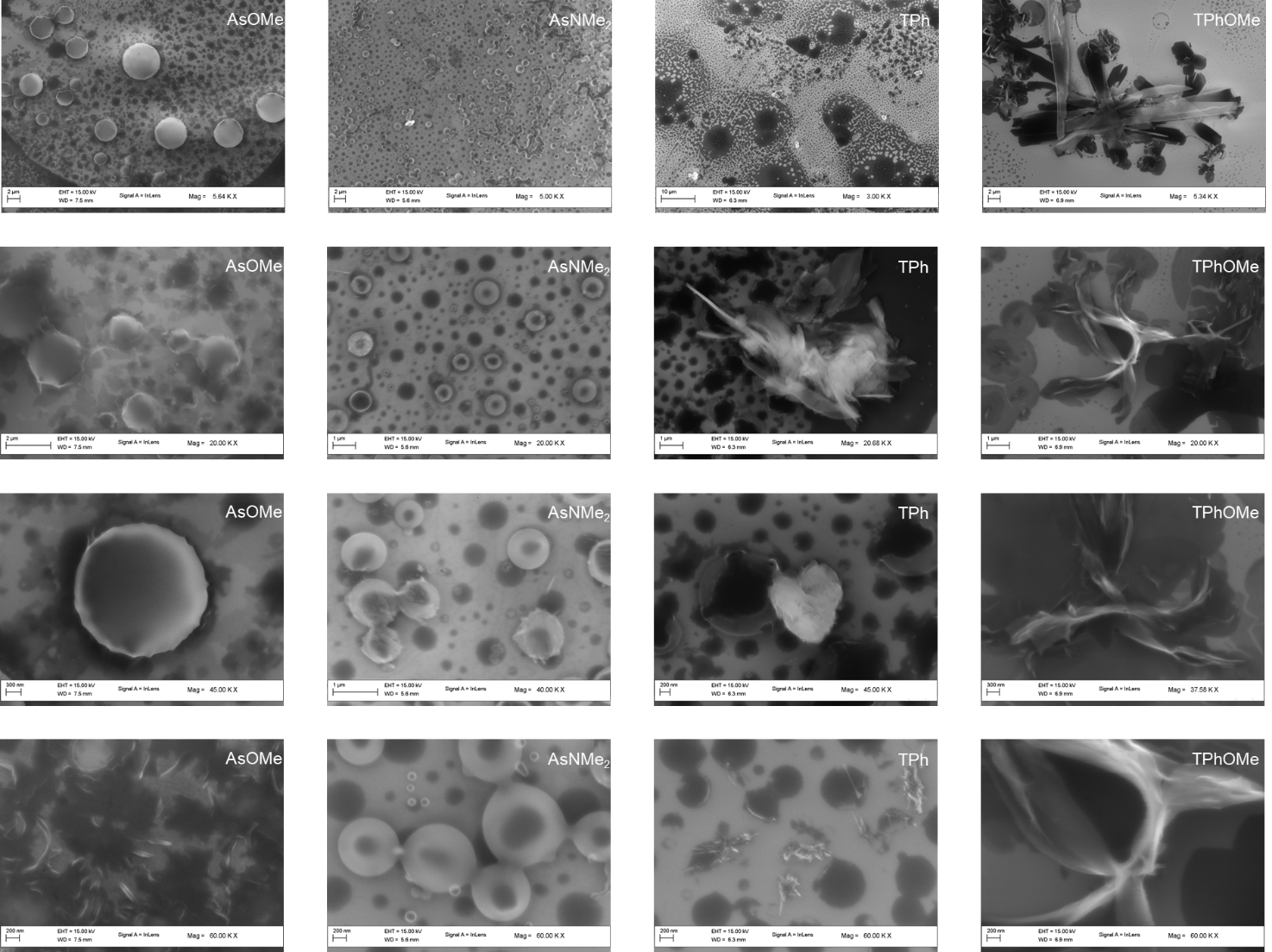


**Figure S118.** FE-SEM images of the **pAQM** spin-coated films deposited onto Si (100) substrate.

[1] Y. Cao, S. Xu, J. Liu, S. Zhao, J. Yan, “Rational construction and evaluation of a dual-functional near-infrared fluorescent probe for the imaging of Amyloid-β and mitochondrial viscosity” *Spectrochimica Acta Part A: Molecular and Biomolecular Spectroscopy* **2024**, *306*, 123564.

[2] M. Montalti, A. Credi, L. Prodi, M. T. Gandolfi, *Handbook of Photochemistry*, CRC Press, Boca Raton, **2006**.

[3] I. Carmichael, G. L. Hug, “Triplet–Triplet Absorption Spectra of Organic Molecules in Condensed Phases” *Journal of Physical and Chemical Reference Data* **1986**, *15*, 1–250.

[4] F. Ortica, A. Romani, G. Favaro, “Light-Induced Hydrogen Abstraction from Isobutanol by Thienyl Phenyl, Dithienyl, and Thienyl Pyridyl Ketones” *J. Phys. Chem. A* **1999**, *103*, 1335–1341.

[5] L. Mencaroni, B. Carlotti, F. Elisei, A. Marrocchi, A. Spalletti, “Exploring a new class of singlet fission fluorene derivatives with high-energy triplets” *Chem. Sci.* **2022**, *13*, 2071–2078.

[6] F. Ricci, F. Elisei, P. Foggi, A. Marrocchi, A. Spalletti, B. Carlotti, “Photobehavior and Nonlinear Optical Properties of Push–Pull, Symmetrical, and Highly Fluorescent Benzothiadiazole Derivatives” *J. Phys. Chem. C* **2016**, *120*, 23726–23739.

[7] S. Stoll, A. Schweiger, “EasySpin, a comprehensive software package for spectral simulation and analysis in EPR” *Journal of Magnetic Resonance* **2006**, *178*, 42–55.

[8] C. E. Tait, M. D. Krzyaniak, S. Stoll, “Computational tools for the simulation and analysis of spin-polarized EPR spectra” *Journal of Magnetic Resonance* **2023**, *349*, 107410.

[9] J. A. Weil, J. R. Bolton, *Electron paramagnetic resonance: elementary theory and practical applications*, Wiley-Interscience, Hoboken, N.J, **2007**.

[10] H. Benk, H. Sixl, “Theory of two coupled triplet states: Application to bicarbene structures” *Molecular Physics* **1981**, *42*, 779–801.

[11] M. Frisch, “Gaussian 09, Revision d. 01, Gaussian” *Inc, Wallingford CT* **2009**, *201*.

[12] J.-D. Chai, M. Head-Gordon, “Long-range corrected hybrid density functionals with damped atom-atom dispersion corrections” *Phys Chem Chem Phys* **2008**, *10*, 6615–6620.

[13] G. A. Petersson, A. Bennett, T. G. Tensfeldt, M. A. Al‐Laham, W. A. Shirley, J. Mantzaris, “A complete basis set model chemistry. I. The total energies of closed‐shell atoms and hydrides of the first‐row elements” *J. Chem. Phys.* **1988**, *89*, 2193–2218.

[14] V. Barone, M. Cossi, “Quantum Calculation of Molecular Energies and Energy Gradients in Solution by a Conductor Solvent Model” *J. Phys. Chem. A* **1998**, *102*, 1995–2001.

[15] C. Ramanan, A. L. Smeigh, J. E. Anthony, T. J. Marks, M. R. Wasielewski, “Competition between Singlet Fission and Charge Separation in Solution-Processed Blend Films of 6,13-Bis(triisopropylsilylethynyl)pentacene with Sterically-Encumbered Perylene-3,4:9,10-bis(dicarboximide)s” *J. Am. Chem. Soc.* **2012**, *134*, 386–397.

[16] E. A. Margulies, C. E. Miller, Y. Wu, L. Ma, G. C. Schatz, R. M. Young, M. R. Wasielewski, “Enabling singlet fission by controlling intramolecular charge transfer in π-stacked covalent terrylenediimide dimers” *Nature Chem* **2016**, *8*, 1120–1125.

[17] B. Carlotti, I. K. Madu, H. Kim, Zhengxu. Cai, H. Jiang, A. K. Muthike, L. Yu, P. M. Zimmerman, T. Goodson, “Activating intramolecular singlet exciton fission by altering π-bridge flexibility in perylene diimide trimers for organic solar cells” *Chem. Sci.* **2020**, *11*, 8757–8770.

[18] A. Toffoletti, Z. Wang, J. Zhao, M. Tommasini, A. Barbon, “Precise determination of the orientation of the transition dipole moment in a Bodipy derivative by analysis of the magnetophotoselection effect” *Physical Chemistry Chemical Physics* **2018**, *20*, 20497–20503.
